# Isoreticular Tuning of Conductive Metal–Organic Framework Nanocrystals for the Rapid Detection and Differentiation of Toxic Gases

**DOI:** 10.1021/acsnano.5c19929

**Published:** 2026-06-10

**Authors:** Elissa O. Shehayeb, Joseph Y. M. Chan, Doran L. Pennington, Christopher H. Hendon, Katherine A. Mirica

**Affiliations:** † Department of Chemistry, Burke Laboratory, 3728 Dartmouth College, Hanover, New Hampshire 03755, United States; ‡ Department of Chemistry and Biochemistry, 3265 University of Oregon, Eugene, Oregon 97403, United States

**Keywords:** metal−organic frameworks, metallotetrapyrazinoporphyrazine, chemiresistive sensors, analyte differentiation, DRIFTS

## Abstract

Despite advances in gas sensing technologies, achieving rapid detection and differentiation of toxic gases remains a critical challenge. Herein, three conductive metal–organic frameworks (cMOFs) based on metallotetrapyrazinoporphyrazine (MTPz) ligands are utilized to enable distinct chemiresistive sensing toward hazardous gases within seconds of exposure. This study focuses on harnessing variations in material–analyte interactions upon tuning the central metal within MTPz ligand (M = Co, Ni, Cu), to achieve discrete sensing responses capable of identifying hydrogen sulfide (H_2_S), ammonia (NH_3_), sulfur dioxide (SO_2_), and nitric oxide (NO), with detection limits as low as 0.7, 0.6, 2.5, and 0.03 ppm, respectively, within 6 seconds of gas exposure. The sensor array differentiates these gases at concentrations exceeding their permissible exposure limits within seconds. Complementary *in situ* and *ex situ* spectroscopic analyses reveal distinct redox processes governing the varied sensing responses, underscoring the critical role of molecular design in optimizing performance. This work establishes a framework for tailoring molecular design strategies toward next-generation gas sensing materials.

## Graphical abstract

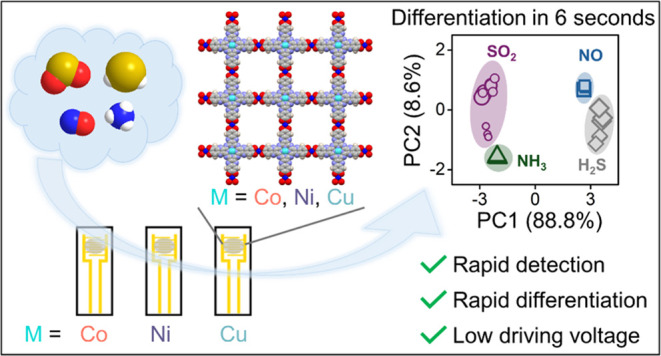

## 1 Introduction

The detection of gaseous pollutants, such as hydrogen sulfide (H_2_S), ammonia (NH_3_), sulfur dioxide (SO_2_), and nitric oxide (NO), is essential for ensuring environmental and industrial safety.^1,2^ Although these gases can be released into the environment from natural processes, such as volcanic eruptions, decomposition of organic matter, and atmospheric photochemical reactions,^3,4^ anthropogenic activities have contributed greatly to their proliferation, primarily through the combustion of fossil fuels for oil refineries, electrical power generation, mass transportation, or industrial operations.^5–7^ These gases threaten both human health^8^ and the environment,^9^ which creates an urgent need for their continuous, on-site, and rapid monitoring in susceptible locations. Hence, there is an increasing demand for the development of low-power, rapid, and reliable sensors capable of detecting the presence of H_2_S, NH_3_, SO_2_, and NO, particularly at concentrations above 20, 50, 5, and 25 ppm, their respective permissible exposure limits (PEL) set by the Occupational Safety and Health Administration (OSHA).^10,11^

Current technologies for the detection of toxic gases constituting electrochemical,^12^ colorimetric,^13^ catalytic,^14^ and infrared^15^ based sensors, are often limited by long analysis times, discontinuous monitoring capabilities, bulky instrumentation, high-power and/or temperature consumption, and the reliance on trained technicians, all of which compromises their portability and field utility.^16^ Chemiresistive sensing offers a practical alternative, enabling sensitive, selective, stable, portable, and low-power detection of H_2_S, NH_3_, SO_2_, and NO.^17^ Recent advancements in chemiresistive sensor technologies have established promising utility of semiconducting materials, including metal oxides,^18^ conducting polymers,^19^ carbon nanotubes,^20^ conductive metal–organic frameworks (cMOFs),^20^ and covalent organic frameworks (COFs),^21^ in delivering robust, rapid, and sensitive responses to toxic gases.^17,22^ Particularly, cMOFs allow the fabrication of sensing devices that require low-power consumption, sustain miniaturization, and provide tunable selectivity through simple nanostructural variations. Incorporating several cMOF devices into an array of chemiresistive sensors using multivariate analyses generates distinct recognition patterns of their collective responses,^23^ further enhancing their overall performance.^24,25^ Preceding reports have integrated cMOFs based on triphenylene,^26,27^ metallophthalocyanine (MPc),^28^ metallonaphthalocyanine (MNPc),^29^ and metalloporphyrazine (MPz) linkers,^30^ into chemiresistive sensing devices as well as arrays for the detection and discrimination of toxic gases and volatile organic compounds. However, these cMOFs often respond slowly, typically requiring at least several minutes of exposure for effective differentiation,^26–30^ thus constricting the ability of their corresponding arrays for rapid, real-time differentiation at relevant concentrations.

Herein, we harness the enhanced intramolecular charge transfer in metallotetrapyrazinoporphyrazine (MTPz) derivatives,^31^ and the rapid response rates and ultrasensitive detection of NO achieved with our previously reported DC-100, a NiTPz-based cMOF linked with copper ions,^32^ to design an array of analogs for differentiating a suite of toxic gases within seconds of exposure. We reasoned that combining the extended conjugation of MNPc-based frameworks^29^ with additional electronegative nitrogen atoms in the backbone structure of MPz-based frameworks^30^ can create (i) electron-deficient MTPz ligand units with enhanced charge transport across the framework and (ii) potential additional interactions with the ligand structure upon analyte exposure.^33^ Building on these findings, and owing to the ability of controlling the magnitude, rate, directionality, and recovery of these responses by strategically changing the metal center^34^ of the MTPz ligand, we herein introduce an array of three MOF analogs from the MTPz-based ligand family for the rapid differentiation of toxic gases. Taken together, the array comprising MTPz-based structures (M = Co, Ni, Cu) linked with copper ions, called DC-103, DC-100, and DC-104, respectively, can enable rapid detection and differentiation of H_2_S, NH_3_, SO_2_, and NO through principal component analysis (PCA). Within a 6-s time frame, the array demonstrates efficient differentiation and low limits of detection (LODs) of 0.7, 0.6, 2.5, and 0.03 ppm for the four gases, respectively, while extended exposures of 10 min can further improve differentiation and sensitivity, reaching ppb-level LODs of 0.1, 30, 172, and 0.3 ppb, respectively. Using diffuse reflectance Fourier transform infrared spectroscopy (DRIFTS), X-ray photoelectron spectroscopy (XPS) analyses, electrochemical impedance spectroscopy (EIS), and computational assessment of electronic properties, we highlight insights into the varying MOF–gas interactions observed for each MOF toward a certain gas, which pave the way to understanding the origin behind the differences in sensing responses. Hence, this work systematically studies how the atomic-level control of the metal ion center in MTPz complexes leads to cMOFs with unique sensing responses to multiple gaseous analytes, enabling low LODs and rapid differentiation of toxic gases for potential applications within air quality monitoring. In addition, this work provides fundamental insights into the electronic properties, nanoscale effects, and material–analyte interactions in this emerging class of materials.

## 2 Results and Discussion

### 2.1 Synthesis and Characterization of MTPz-Based MOFs

We followed a modular procedure, previously developed in our lab,^32^ for the preparation of octahydroxy-substituted MTPz monomers, denoted by MTPz­(OH)_8_ with M = Co, Ni, or Cu, detailed in Section S2 and Figures S1–S21. We synthesized the series of MTPz-Cu-MOFs using solvothermal means by the coordination of the three different monomers with copper­(II) ions in ethylene diamine (EDA) and anhydrous dimethyl sulfoxide (DMSO) to yield crystalline material (Figure 1a). Powder X-ray diffraction (PXRD) confirmed the crystallinity of the MOFs, showing significant diffraction patterns at 2θ values of 3.9°, 5.6°, 7.9°, and 27.5°, corresponding to the (100), (110), (200), and {001} facets, respectively. These peaks matched the simulated pattern for the eclipsed, rather than the staggered, stacking model of MTPz-Cu-MOFs (Figures 1b and S22).

**Figure 1:**
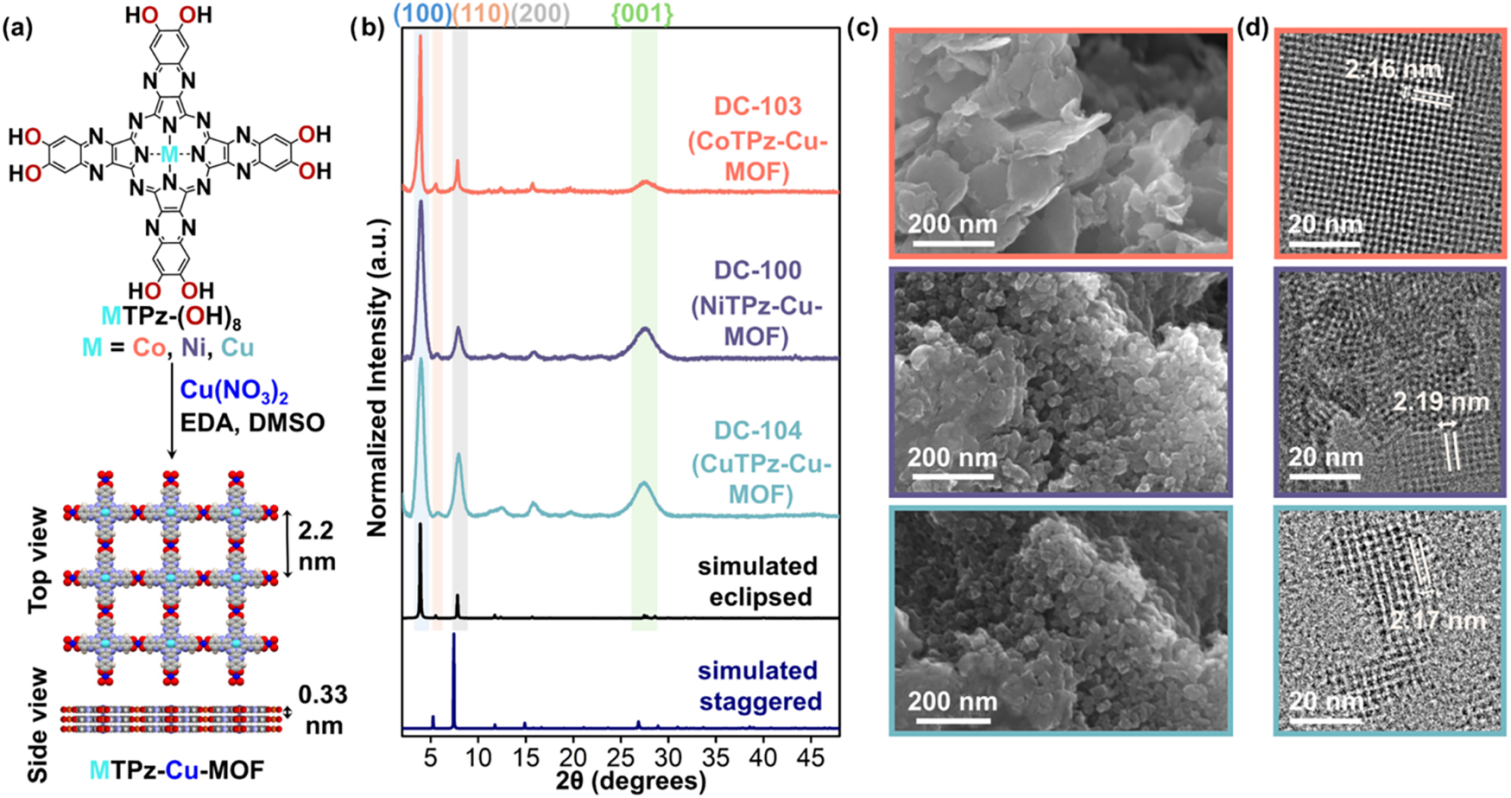
(a) Synthetic scheme for the synthesis of MTPz-Cu-MOFs where M = Co, Ni, or Cu. (b) Experimental PXRD diffraction patterns of the three MTPz-Cu-MOFs compared to the simulated eclipsed AA stacking and the staggered ABAB stacking. (c) SEM images and (d) TEM images of DC-103, DC-100, and DC-104, respectively.

We evaluated the nanoscale morphology, crystallinity, and long-range order of all MOFs using scanning electron microscopy (SEM) and transmission electron microscopy (TEM), Figures 1c,d, S23, and S24. Energy dispersive X-ray (EDX) spectroscopy confirmed uniform distribution of the expected elements in each analog (Figure S25). TEM images validated the experimental interspacing distances between the (100) planes of the square-like pores to be around 2.2 nm (Figure S26, eq S1, and Table S1), consistent with values calculated from PXRD using Bragg’s Law (eq S2, Figure S27, and Table S2). To further establish the chemical composition of the three frameworks, we performed CHN combustion analysis (Table S3), inductively coupled plasma mass spectrometry (ICP-MS), and thermogravimetric analysis (TGA) (Figure S28). Together, these results confirmed the incorporation of water molecules and residual DMSO and/or EDA molecules within the MOF unit cell (Table S4).

In addition, attenuated total reflectance–Fourier transform infrared (ATR-FTIR) spectra of the MTPz­(OH)_8_ monomers and their corresponding MOFs (Figure S29) and XPS spectra (Figures S30–S33) provided evidence for coordination bond formation, by revealing contributions from both C=O and C–O states in the O 1s region, indicative of semiquinoidal ligand configuration.^32^ We also noted the presence of C=N bonds, C=N–M bonds,^29^ and π–π* shake up satellite^35^ features from the TPz core in the N 1s region, as well as mixed-valence oxidation state of copper bridging ions with Cu­(I):Cu­(II) ratios of 54:46, 63:37, and 49:51 for DC-103, DC-100, and DC-104, respectively. The paramagnetic nature of the d^9^ Cu­(II) ions was affirmed by the electron paramagnetic resonance (EPR) spectra of the three MOFs at *g* = 2.06, as shown in Figure S34.^36^

Four-point probe measurements revealed semiconductive properties of the layered MOF structures, with electrical conductivity values of 7.6 × 10^–4^, 2.6 × 10^–6^, and 9.7 × 10^–7^ S cm^–1^ for DC-103, DC-100, and DC-104, respectively (eq S3, Figure S35, and Table S5). Proton conductivity measurements of the three MOFs, collected at 303–333 K and 98% relative humidity, demonstrated values in the range of 0.2–4.0 × 10^–5^ S cm^–1^ (Figure S36 and Table S5). Notably, DC-103 exhibited an activation energy of 0.61 eV, consistent with a slow vehicular proton transport mechanism, whereas DC-100 and DC-104 showed lower activation energy values of 0.41 and 0.40 eV, respectively, suggesting a Grotthuss hopping mechanism (Figure S36).^37^ Taken together, these results suggest that charge transport in DC-103 is predominantly electronic, as its electrical conductivity exceeds its proton conductivity by approximately 1 order of magnitude. In contrast, DC-100 and DC-104 exhibit higher proton conductivities relative to their electrical conductivities (by one and 2 orders of magnitude, respectively), suggesting that ionic species in the local chemical environment may play a more significant role in governing charge transport in these analogs.

Evaluating the porosity of the materials using nitrogen sorption isotherms displayed Brunauer–Emmett–Teller (BET) surface areas of 313, 396, and 347 m^2^ g^−1^ for DC-103, DC-100, and DC-104, respectively (Figure S37 and Table S6). UV–vis–NIR (ultraviolet–visible–near-infrared) absorption spectra and their corresponding Tauc plots revealed optical band gap values of 1.18, 1.32, and 1.21 eV for thin films of DC-103, DC-100, and DC-104, respectively (Figure S38). Density functional theory (DFT) band structure calculations provided qualitative support for the experimentally observed band gaps. Notably, computational examination of charge-neutral MOF structures predicted pronounced metallic character, while the incorporation of reduced pyrazine units within the MOF structure provided an improved alignment with experimental results (Figure S39). The redox activity of the pyrazine moiety is consistent with previous reports.^38–40^ Although we cannot definitively assign the precise oxidation state of the pyrazine subunits from the available spectroscopic and computational data, these results collectively support the presence of discrete, qualitatively similar semiconducting band gaps across all three analogs. With these structural and physicochemical characterizations of the three materials, we sought to investigate the influence of varying the central MTPz metal ion on their sensing responses toward toxic gases.

### 2.2 Chemiresistive Sensing Responses of MTPz-Cu-MOFs

We evaluated the chemiresistive sensing responses of the three MOFs toward H_2_S, NH_3_, SO_2_, and NO. While these gases are important to detect in real-life applications, they also serve as surface probes that can provide spectroscopic evidence for distinct material–analyte interactions. Briefly, we dropcasted aqueous suspensions of the MOFs onto gold interdigitated electrodes (see Section S5.1 and Figures S40 and S41) and tested the sensing response of these devices toward the different gases at concentrations of 40, 20, 10, 5, and 1 ppm in dry nitrogen (N_2_) atmosphere (see Section S5.2 and Figure S42). Each sensing cycle consisted of a 10 min N_2_ baseline, 10 min exposure to the specific concentration of analyte, followed by a 30 min recovery period under N_2_. The full sensing profiles, computed as −Δ*G*/*G*_0_, the normalized change in response (eq S4), are shown in Figures 2 and S43–S54.

**Figure 2:**
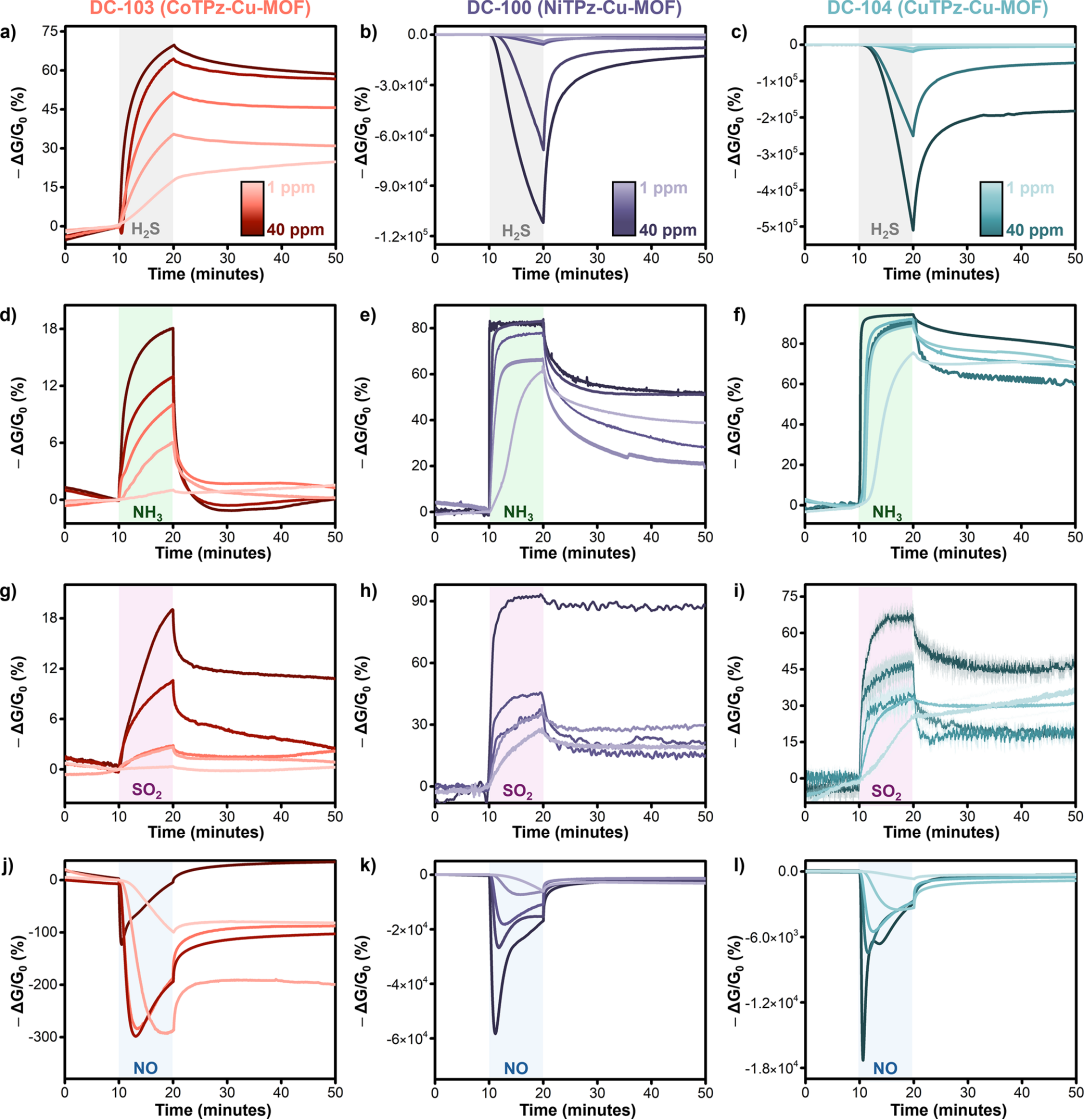
Average sensing responses of DC-103, DC-100, and DC-104 toward (a–c) H_2_S, (d–f) NH_3_, (g–i) SO_2_, and (j–l) NO at the different concentrations (40, 20, 10, 5, and 1 ppm) in N_2_. The shaded region indicates the time of exposure to each gaseous analyte. The sensing traces are representative averages of at least three replicate devices, which are provided in Section S5.

We observed distinct and gas-specific sensing responses across the three MOFs. Toward 40 ppm of H_2_S, which is a reducing gas, DC-100 and DC-104 showed remarkable decreases in the normalized response reaching values of −100,000% and −500,000%, respectively. In contrast, DC-103 showed an increase of 70% under the same conditions, highlighting different H_2_S-induced perturbations to the electronic properties of the MOF material dependent on the nature of the central MTPz metal ion. In the case of NH_3_, another reducing gas, all three MOFs displayed positive responses, albeit with varying rates and magnitudes. Unlike DC-103, which showed a relatively slow increase in −Δ*G*/*G*_0_ to a maximum of 18% after 10 min of 40 ppm NH_3_ exposure, we observed preeminent rapid responses for DC-100 and DC-104 that saturate at around 80% and 90%, respectively, within 1-to-3 min of exposure. Responses toward SO_2_ mirrored those for NH_3_, with DC-103 showing a maximum response of 18% toward 40 ppm of SO_2_ while DC-100 and DC-104 showed maximum percentages that saturated within 5 min of exposure, with values of 90% and 65%, respectively. Toward NO, an oxidizing gas, all three MOFs exhibited a decrease in response, characterized by an abrupt saturation within 1–2 min of exposure, followed by a gradual decrease in signal magnitude. This observation is attributed to competitive surface adsorption and subsequent bulk diffusion of NO within the TPz-based MOFs, which has been previously studied for zinc oxide materials upon hydrogen gas detection.^41^ To summarize these findings, Figure 3a–d displays the magnitude of response upon 10 min of exposure to 20 ppm of analyte gas in N_2_. These varying responses and magnitudes hint toward the possibility of enhanced differentiation of these toxic gases at concentrations that compromise human health and safety.

**Figure 3:**
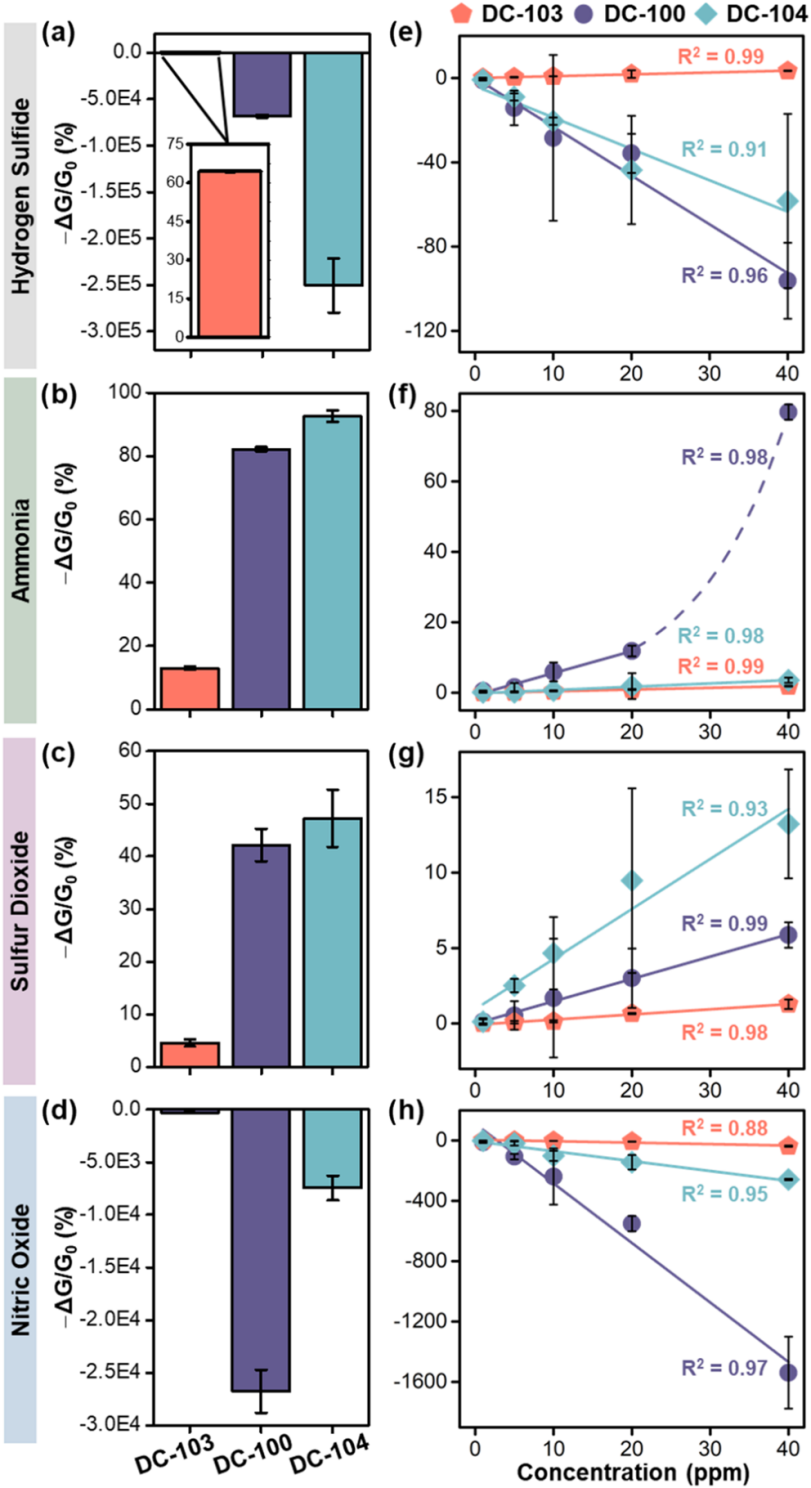
Magnitude of responses after 10 min of exposure toward 20 ppm of (a) H_2_S, (b) NH_3_, (c) SO_2_, and (d) NO in N_2_. Linear concentration-dependent responses of DC-103, DC-100, and DC-104 at 6 s of exposure toward (e) H_**2**_S, (f) NH_3_, (g) SO_2_, and (h) NO in N_2_.

We then investigated the sensitivity, response time, recyclability, and reversibility of the chemiresistive responses by extracting different sensing features. To assess sensitivity, we first evaluated the concentration-dependent responses at short exposure intervals of 6, 15, 30, and 60 s, as well as after the full 10 min exposure (Figures 3e–h and S55–S59). From empirically-derived linear relationships, we computed LODs (Table S7) as low as 0.7, 0.6, 2.5, and 0.03 ppm toward H_2_S, NH_3_, SO_2_, and NO, respectively, after only 6 s of exposure. Longer exposure times allowed for further improvements, ultimately reaching 0.1, 30, 172, and 0.3 ppb, respectively, upon the full 10 min exposure. Subsequently, we assessed initial rates of response (RoRs), which generally displayed a linear dependence on analyte concentration across all gases (Figures S60–S63). This feature captures the dynamic interaction between gas molecules and the MOF surface, as well as the fast charge transduction within the frameworks, providing useful parameters for rapid analyte differentiation.

To probe recyclability, we performed alternating 10 min exposure, followed by 10 min nitrogen purge experiments, repeated for up to 13 cycles (Figures S64–S77). In nearly all cases, the first exposure produced the strongest signal, followed by either (i) consistent responses in subsequent cycles, as observed for NH_3_, or (ii) progressively diminished responses, as observed for H_2_S, SO_2_, and NO in N_2_ atmosphere. We ascribed this decrease across succeeding cycles to partial device recovery, where some active sites remain occupied with preadsorbed analyte gas^42^ or with products resulting from the interaction of the analyte gas with the MOF (more details provided later in the Section 2.4). The enhanced recyclability over previously reported materials^23,29,30^ prompted our interest to quantitatively evaluate the recovery percentage (eq S5 and Figure S78). Briefly, the most significant results include 100% recovery of DC-103 toward NH_3_, and 80–90% for DC-100 and DC-104 toward 20 ppm of H_2_S and NO. All three analogs retain moderate recovery, namely 60–80%, after exposure to 20 ppm of SO_2_. Based on the differences in features extracted from sensing responses of the cMOFs toward the various gases, we aimed to study the differentiation ability of this array through PCA.

### 2.3 Principal Component Analysis

We performed PCA to determine the efficiency of the MTPz-Cu-MOF array in discerning the sensing responses from various analytes. To capture both rapid and long-term properties, we extracted features from data sets acquired at the full 10 min exposure, as well as at short time scales (as low as 6 s; details in Section S5). For analyses using full exposure, the chosen PCA features included (i) the initial RoR calculated as the slope of a fitted straight line during the first minute of exposure, (ii) the magnitude of response upon 10 min of exposure, and (iii) the area under the curve during the 10 min exposure time. PCA results of these values showed the ability of the array to distinguish gaseous analytes, particularly at their respective OSHA PEL values (Figures 4a, S79, and S80). Harnessing the unique rapid responses of MTPz-Cu-MOFs, we also performed postprocessing PCA analyses using data features extracted exclusively from the first 6 s of exposure within the full 10 min sensing experiment: (i) initial RoR as slope fitted within the data points of the first 6 s, (ii) magnitude of response at 6 s, and (iii) area under the curve between 0 and 6 s of exposure. Interestingly, the MTPz-Cu-MOF array is capable of fully differentiating sensing responses from H_2_S, NH_3_, SO_2_, and NO above their respective OSHA PEL concentrations^10,11^ during this very short time frame (Figures 4b and S81). The ability to achieve differentiated responses within just 6 s underscores the unique molecular design of this particular array compared to other cMOF-based sensor arrays,^26–29^ which typically require analyses of at least several minutes to achieve differentiation. Compared to other sensor platforms, the MTPz-Cu-MOF array not only surpasses the rapid differentiation capabilities of chemiresistive sensors based on metal oxides,^43,44^ graphene nanostructures,^45^ conductive polymers,^46^ and molecular complex semiconductors,^47^ which generally require at least 1–3 min of response and their respective recovery, but also outperforms other sensing modalities, including colorimetric^48^ and field-effect transistor^49^ sensors, which need 2–10 min of exposure to achieve effective analyte discrimination.

**Figure 4:**
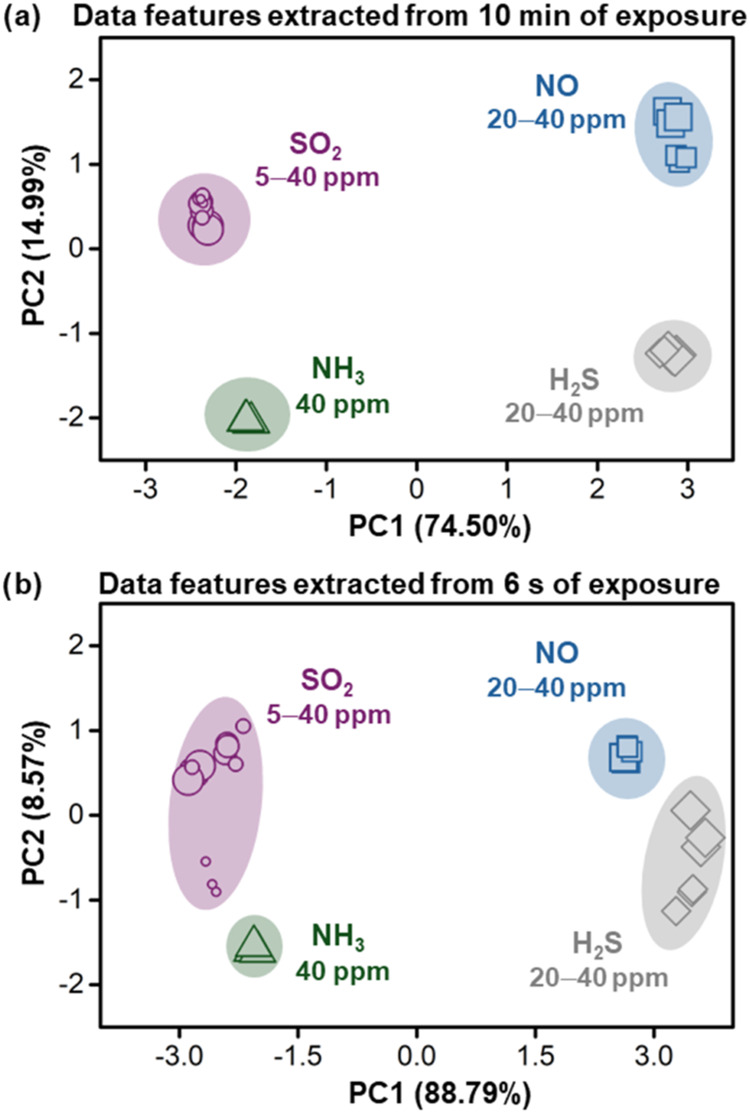
PCA plots of the extracted features (initial RoR, magnitude of response, and area under the curve during exposure) upon (a) 10 min and (b) 6 s of exposure of gaseous analytes around or above their corresponding OSHA PEL limits in N_2_ balance.

### 2.4 Spectroscopic Insights into Material–Analyte Interactions

To gain insight into the distinct responses of the MTPz-Cu-MOF analogs, we performed DRIFTS experiments (Figures 5 and S82–S93) and postexposure MOF characterization to probe the material-analyte interactions upon subjection to gas analytes (Figures S94–S109). While *ex situ* XPS measurements provide valuable insights into oxidation-state changes within the MOFs, it is important to note several limitations that may complicate direct correlation with the sensing results. These limitations include the use of high analyte concentrations (10,000 ppm) and prolonged exposure times (2 h) necessary to obtain high-quality, high-resolution spectra, as well as the vacuum conditions required during XPS data acquisition, which can promote analyte desorption and do not reflect ambient sensing environments. In contrast, DRIFTS enables the direct observation of analyte-induced perturbations. Notably, the DRIFTS spectra of DC-103 exhibit a decrease in the broad electronic absorbance (BEA) baseline upon exposure to all four gases, for which increasing peaks would appear even below the original baseline prior to exposure (Figures S82, S85, S87, and S91). We attribute these features to modifications in the electronic properties of the framework upon analyte interactions.^50–52^

**Figure 5:**
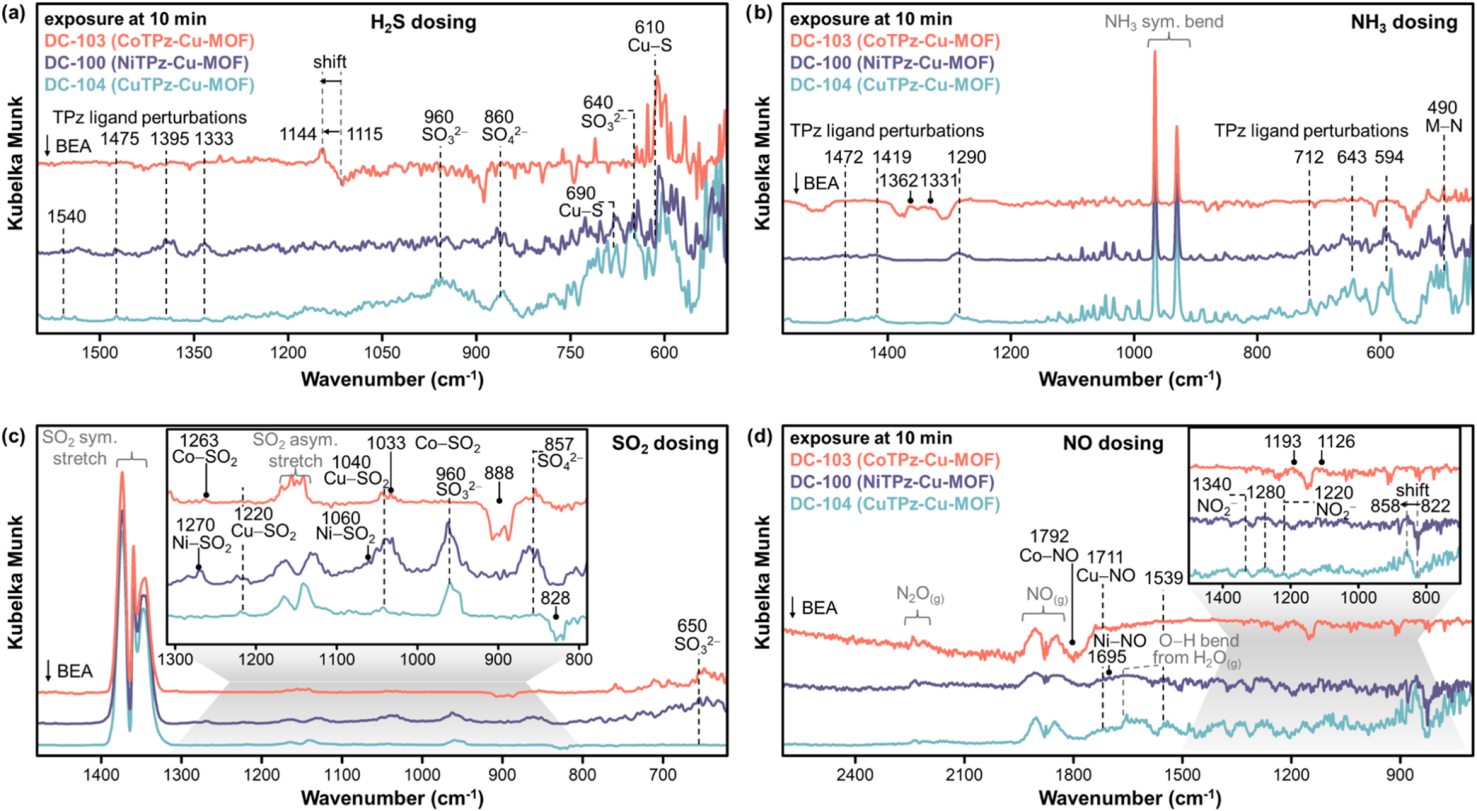
DRIFTS difference spectra recorded at 10 min of exposure for DC-103, DC-100, and DC-104 toward (a) 1000 ppm of H_2_S, (b) 1% of NH_3_, (c) 1% of SO_2_, and (d) 1% of NO in N_2_. The baselines in the plots are vertically offset for clarity.

Upon exposure to H_2_S, while DRIFTS spectra of the MOF series showed positive Kubelka–Munk peaks at around 1474, 1395, 1333, 960, 860, and 610 cm^–1^ for all three analogs, we observed positive peaks at 1540, 690, and 640 cm^–1^ only for DC-100 and DC-104 and a shift from 1115 to 1144 cm^–1^ for DC-103. These variations, attributed to perturbations in the MTPz ligand structure, correlate with the reversed directionalities observed in the sensing responses (Figure 2a–c). Particularly, we attributed the bands at 1540 and 1474 cm^–1^ to aromatic C–C stretching and those at 1395 and 1333 cm^–1^ to C–H bending vibrations (Figures 5a and S82–S84).^53^ In addition, we reasoned that the shift from 1115 to 1144 cm^–1^ resulted from deviations in the skeletal core of the central ligand structure of DC-103.^53^ However, we ascribed the other bands between 610 and 960 cm^–1^ to oxidized sulfurous species formed upon the transformation of H_2_S in the presence of the MOFs, specifically to S–O stretching vibrations of sulfite species (M−SO_3_^2–^ at 960 and 640 cm^–1^)^54^ and sulfate species (M−SO_4_^2–^ at 860 cm^–1^),^55^ in addition to the formation of metal sulfide bonds (M−S at 690 and 610 cm^–1^).^56^ The differences in interactions of DC-103 and the other analogs with H_2_S also appear in XPS analysis performed on the MOF powder upon exposure to 1% H_2_S in N_2_ for 2 h, where we observed newly developed S 2p peaks deconvoluted into varying species that are highly similar for DC-100 and DC-104 analogs, but of different composition for DC-103. The deconvolutions include combinations of hydrogen sulfite (HSO_3_^–^ at binding energy = 170.1 eV),^57^ SO_4_^2–^ (168.9 eV),^58^ SO_3_^2–^ (167.4 eV),^58^ H_2_S–M interactions (165.2 eV),^21^ elemental sulfur (S_0_, 164.1 eV),^59^ polysulfides (S_*x*_, 163.2 and 162.3 eV),^59^ as well as metal sulfide species (M–S at 161.4 eV),^57^ Figures S94–S96. To compensate for these oxidation state changes, we tried tracking the oxidation state of the ligand within the framework after exposure, as well as that of the copper bridging ions. While drawing conclusions from the former is inaccurate due to the overlap of peaks from binding energies of S–O and S=O over those of C–O and C=O between 530 and 533 eV,^60–62^ we noted the partial reduction of copper ions in DC-103 and DC-104 upon H_2_S exposure from a Cu­(I):Cu­(II) ratio of 54:46 to 63:37 and 49:51 to 56:44, respectively (Figures S94–S96). Hence, we attributed the differences in response directionalities to a variation in the electron transport within the framework of DC-103 when exposed to H_2_S, compared to that within DC-100 and DC-104, as confirmed by DRIFTS experiments which showed the different trends in MTPz perturbation upon H_2_S exposure.

Similarly, with exposure to NH_3_, we observed distinct differences in spectroscopic features for DC-100 and DC-104 in comparison with those of DC-103, which may pose as a plausible explanation for the significant difference in the magnitude and rate of the sensing responses, as well as the trends in recovery (Figures S85–S87). Though bands arising from gaseous or bound NH_3_ were present in all spectra, notably the shoulder at around 3220 cm^–1^ and bands at 1626, 966, 930, 650, 594, and 490 cm^–1^ corresponding to NH_3_ asymmetric stretching, symmetric bending,^50,56^ rocking,^63^ and M−N stretching of metal-bound NH_3_,^63^ we noted the presence of two extra bands at 1331 and 1362 cm^–1^ in the spectra of DC-103 additional to those at 1472, 1419, 1290, 712, 643, and 594 cm^–1^ appearing in the other analogs, which we attributed to vibrational modes of the TPz ligand.^53^ Complimenting the DRIFTS experiments, we noted minimal variations in oxidation states of DC-103 components, unlike the partial reduction of the ligand from C=O:C–O ratios of 54:41 to 47:39 in DC-100 and the partial shift of Cu­(I):Cu­(II) ratios of 63:37 to 55:45 in DC-100 and from 49:51 to 39:61 in DC-104 (Figures S97–S99).

In response to SO_2_, we distinguished the newly emerging bands at 1373, 1359, and 1344 cm^–1^ as gas-phase SO_2_ asymmetric stretching and those at 1160 and 1140 cm^–1^ as the symmetric stretching vibrations.^64^ For the DRIFTS spectra of all three MOFs (Figures S88–S90), we noticed bands relating to metal-bound SO_2_ (M−SO_2_) interactions, where the vibrations from Cu−SO_2_ appeared in the spectra of all analogs at around 1220 cm^–1^ (asymmetric S–O stretching) and 1040 cm^–1^ (symmetric S–O stretching).^65^ In addition to the latter bands, DRIFTS spectra of DC-100 contained S–O asymmetric and symmetric stretching vibrations of Ni−SO_2_ at 1270 and 1060 cm^–1^, whereas that of DC-103 contained the corresponding vibrations for Co-bound SO_2_ at 1263 and 1033 cm^–1^, respectively.^66^ Furthermore, for the three analogs, we noted the formation of SO_3_^2–^ and SO_4_^2–^ species, confirmed by their corresponding S–O stretching bands at 963, 857, and 655 cm^–1^,^54,55^ respectively, as well as by analyzing their XPS spectra after exposure in which peaks at binding energies of 168.8 and 167.6 eV emerged (Figures S100–S102).^58^ We hypothesize that the oxidation of the sulfurous species is complemented by the partial reduction of the TPz ligand, which cannot be fully confirmed due to the overlap of S–O and S=O from the SO_3_^2–^ and SO_4_^2–^ species in the O 1s region of the postexposure XPS analyses,^60–62^ mainly due to the constant or very slight variation in the oxidation states of the metal ions and the TPz ligand structure.

Upon exposure to NO, the common series of peaks emerging from the presence of gaseous analyte include 1907, 1875, and 1847 cm^–1^ from gaseous NO,^56^ and 2240 and 2209 cm^–1^, corresponding to nitrous oxide (N_2_O) formed in situ (Figures S91–S93).^67^ The observed formation of N_2_O under N_2_ may arise from reductive coupling of NO at the redox-active metal centers of the MOF, consistent with previous reports of NO disproportionation catalyzed by transition-metal sites.^68,69^ In addition, we discerned a broad absorption at around 1700 cm^–1^ consisting of two band combinations: one at 1717 cm^–1^ for Cu­(I)-NO interactions, common for all three cMOFs, and the other at 1792 cm^–1^ for Co−NO interactions and at 1694 cm^–1^ for Ni−NO interactions in the DC-103 and DC-100 spectra, respectively.^32,56,70^ These spectroscopic observations are consistent with previously reported results.^32^ We also noted the formation of nitrite (NO_2_^–^) ions in DC-100, as well as DC-104, as indicated by bands at wavenumbers 1340 and 1220 cm^–1^ in the DRIFTS spectra and peaks at a binding energy of 403.5 eV in their N 1s XPS spectra (Figures S103–S105).^71–73^ Potential sources of oxygen (O_2_) required to enable this oxidation under our experimental conditions include trace residual O_2_ in the house N_2_ background stream and/or O_2_ adsorbed on the surface of the TPz-MOFs.^21^ We allocated the other bands at 1539, 1282, and 856 cm^–1^ observed for the three MOF analogs to stretching or bending vibrations resulting from electronic alterations within the TPz framework upon the interaction with NO. These spectroscopic analyses provide insight into the chemical speciation and oxidation-state changes of the MOF components upon gas exposure, which govern the observed distinct chemiresistive responses.

To probe the origin of the differing electrical response directionalities toward the studied reducing gases (H_2_S, NH_3_, and SO_2_), we collected UV–vis−NIR spectra upon gas exposure, from which we extracted optical band gaps. Upon exposure to H_2_S, DC-103 exhibited an increase in resistance (Figure 2a), which was accompanied by an increase in the optical band gap from 1.18 to 1.24 eV (Figure S107a). In contrast, DC-100 and DC-104, which displayed a decrease in resistance upon H_2_S exposure (Figure 2b,c), showed slight decreases in their optical band gap values (Figure S107b,c). For NH_3_ and SO_2_, all three MOFs exhibited increases in resistance (Figure 2d–i), which correlated well with the slight increases in optical band gaps upon exposure to each of these gases (Figures S108 and S109). These trends indicate a correlation between changes in the optical band gap and the directionality of the chemiresistive response, consistent with prior reports on gas-induced band gap modulation in metal oxide nanomaterials.^74^

To further decouple possible electronic and ionic contributions to the observed responses, we performed EIS measurements upon gas exposure under alternating current conditions. The EIS results confirmed the two general trends observed through chronoamperometry. First, DC-103 exhibited an increase in resistance upon H_2_S exposure within the same order of magnitude of impedance values (10–60 kΩ), Figures S110 and S111. In contrast, DC-100 and DC-104 showed a substantial decrease in resistance spanning over 2 orders of magnitude (50–0.2 MΩ for DC-100 and 120–0.2 MΩ for DC-104), Figures S112 and S113. Second, the three MOF analogs displayed similar increase in resistance within the same order of magnitude upon SO_2_ exposure (Figures S114–S116). Moreover, analysis of characteristic frequencies revealed notable differences across three materials. While the impedance analysis of DC-103 demonstrated peak frequencies at ∼10^4^–10^5^ Hz, similar peaks for DC-100 and DC-104 emerged at lower frequency values of ∼10^1^–10^3^ Hz (Figure S117). Taken together, these results suggested possible differences in charge transport mechanisms across these materials, where responses in DC-103 may be dominated by electronic effects, while ionic contributions exert a more significant influence in responses of DC-100 and DC-104 for Brønsted acid analytes, such as H_2_S.

To probe the effect on the nanoscale, we examined the zeta potential and crystallite sizes of the MOF analogs in their aqueous suspension. DC-100 and DC-104 exhibited more negative average zeta potentials (−30.8 and –41.4 mV, respectively) than DC-103 (−15.2 mV), indicating higher surface charge density on the former two analogs (Figures S118 and S119). Dynamic light scattering analysis revealed larger crystallite sizes for DC-103 (mean radius 139.8 nm) relative to DC-100 (81.8 nm) and DC-104 (68.1 nm), Figure S120. These findings suggested that the larger, less charged crystallites of DC-103, combined with its higher electronic conductivity and limited proton transport, render its sensing response less sensitive to ionic effects. In contrast, the smaller, more charged particles of DC-100 and DC-104, characterized by higher protonic conductivities over electrical conductivities, were more susceptible to ion-mediated interactions. We conclude that, in sensor architectures comprising randomly oriented crystallites, where interparticle junctions dominate charge transport, nanoscale factors, such as crystallite size and surface charge, may introduce significant ionic contributions that compete with or override intrinsic electronic properties. This effect is particularly pronounced for Brønsted acidic analytes capable of introducing mobile protons into the system. Collectively, these results highlight the distinct interactions occurring between the three MOF analogs and the four different gases, corroborating similar mechanisms for similar sensing performances and underscoring varying mechanisms for those of reversed directionalities of responses.

## 3 Conclusion

In summary, we have developed a chemiresistive sensor array based on MTPz-based cMOFs with varying central TPz metal centers (M = Co, Ni, and Cu), capable of distinctly detecting four toxic gases, namely H_2_S, NH_3_, SO_2_, and NO at a low driving voltage of 0.1 V. The exceptionally rapid and reliable sensing responses enable detection and differentiation of the toxic gases above their OSHA PEL values with low detection limits of 0.7, 0.6, 2.5, and 0.03 ppm within just 6 s of exposure, highlighting the uniqueness of this cMOF combination. We attribute the ability to achieve reliable rapid response signals to the more electron-deficient nature,^75^ as well as the enhanced intramolecular charge transfer^31^ of MTPz structures compared to MPz, MPc, and MNPc-based structures (Table S8), owing to the combined effects of extended conjugation and electron-withdrawing ability of the additional nitrogen atoms in MTPz-based MOFs.^76^ By probing the underlying material–analyte interactions, we demonstrate the critical role of targeted molecular design in achieving fast and distinct responses.

This work emphasizes the investigation of the fundamental properties of MTPz-based MOFs in response to toxic gases. From an applied perspective, the current demonstrations have two limitations. First, while TPz-based MOFs provide significant advantages for rapid sensing, complete differentiation across all gas concentrations was not achieved with this particular array (Figure S79). We attribute this limitation to the similarity in response features across different MOF/gas concentration pairings, such as the case of DC-103 toward 40 ppm of NH_3_ with DC-100 toward 1 ppm of SO_2_ (Figure 2d,h). Previous reports have shown full concentration-resolved discrimination in other sensor systems, suggesting that further optimization, such as tuning the bridging metal ion,^34^ herein fixed as copper, may enhance the performance. Second, another important parameter to consider is the performance of the sensors under complex environments including interference from air and humidity. Additional work focused on chemical sensing in complex environments and in the presence of complex mixtures and interferents would be necessary for achieving practical implementation. Overall, this work establishes a foundation for future efforts aimed at advancing MOF-based arrays toward comprehensive, ultrafast discrimination of toxic gases across varying concentrations within a short exposure time frame.

## 4 Experimental Section

A complete account of materials, methods, monomer syntheses, and supporting experimental details can be found in the Supporting Information.

### 4.1 Synthesis of DC-103

CoTPz­(OH)_8_ (10 mg, 0.011 mmol, 1 equiv) was charged into a high-pressure Schlenk flask and subjected to three vacuum-nitrogen cycles. Under nitrogen, 24 mL of anhydrous DMSO were added, then the tube was sealed and sonicated for 5 min. Under argon, a solution of Cu­(NO_3_)_2_·2.5H_2_O, (5.38 mg, 0.023 mmol, 2.1 equiv) in 1 mL of anhydrous DMSO was added, followed by EDA (0.29 mL, 4.4 mmol, 400 equiv). The reaction vessel was sealed and placed in a preheated oven at 85 °C for 15 h. After cooling to room temperature, the light brown solution with black precipitate was transferred to a centrifuge tube. The resulting solid was washed and decanted successively with 25 mL of DMF, water, and acetone, and dried in a vacuum oven overnight.

### 4.2 Synthesis of DC-100

NiTPz­(OH)_8_ (10 mg, 0.011 mmol, 1 equiv) was charged into a high-pressure Schlenk flask and subjected to three vacuum-nitrogen cycles. Under nitrogen, 24 mL of anhydrous DMSO were added, then the tube was sealed and sonicated for 5 min. Under argon, a solution of Cu­(NO_3_)_2_·2.5H_2_O, (5.38 mg, 0.023 mmol, 2.1 equiv) in 1 mL of anhydrous DMSO was added, followed by EDA (1.18 mL, 17.6 mmol, 1600 equiv). The reaction vessel was sealed and placed in a preheated oven at 85 °C for 15 h. After cooling to room temperature, the light brown solution with black precipitate was transferred to a centrifuge tube. The resulting solid was washed and decanted successively with 25 mL of DMF, water, and acetone, and dried in a vacuum oven overnight.

### 4.3 Synthesis of DC-104

CuTPz­(OH)_8_ (10 mg, 0.011 mmol, 1 equiv) was charged into a high-pressure Schlenk flask and subjected to three vacuum-nitrogen cycles. Under nitrogen, 24 mL of anhydrous DMSO were added, then the tube was sealed and sonicated for 5 min. Under argon, a solution of Cu­(NO_3_)_2_·2.5H_2_O, (5.38 mg, 0.023 mmol, 2.1 equiv) in 1 mL of anhydrous DMSO was added, followed by EDA (1.18 mL, 17.6 mmol, 1600 equiv). The reaction vessel was sealed and placed in a preheated oven at 85 °C for 15 h. After cooling to room temperature, the light brown solution with black precipitate was transferred to a centrifuge tube. The resulting solid was washed and decanted successively with 25 mL of DMF, water, and acetone, and dried in a vacuum oven overnight.

### 4.4 Sensing Experiments

Chemiresistive devices were fabricated by dropcasting 25 μL of a 1 mg mL^–1^ aqueous MOF suspension onto interdigitated electrodes. The devices were electrically connected to a potentiostat via a breadboard and enclosed within a custom-built Teflon chamber to control gas in- and outflow. Analyte gases, supplied as 1% analyte/N_2_ mixtures, were introduced through mass flow controllers and diluted with N_2_ to achieve concentrations ranging from 2 to 40 ppm. Chemiresistive responses were monitored as time-dependent changes in current upon analyte exposure and are reported as the negative normalized conductance change (−Δ*G*/*G*_0_).

### 4.5 DRIFTS Experiments

MOF/KBr composites were loaded into an airtight steel chamber equipped with gas inlet/outlet ports and KBr windows. Samples were activated at 110 °C under N_2_ flow for 1 h prior to analysis. Spectra were collected from 400–4000 cm^–1^ with 32 scans per spectrum, and difference spectra (Kubelka–Munk) were generated relative to the initial spectrum. Time-dependent spectra were recorded during analyte exposure and subsequent N_2_ purging. To improve signal intensity, higher analyte concentrations were used for DRIFTS experiments, including 0.1% H_2_S and 1% NH_3_, SO_2_, and NO in N_2_.

## Supplementary Material



## Notes

Additional experimental methods, synthesis and characterization details, gas sensing experiments, *in situ* DRIFTS and postexposure XPS spectra. (PDF)
