# Supplementary material for: Isoreticular Tuning of Conductive Metal–Organic Framework Nanocrystals for the Rapid Detection and Differentiation of Toxic Gases
Source: ACS Nano. 2026 Jun 10;20(24):17233–43. doi: 10.1021/acsnano.5c19929 (PMC13296508; doi:10.1021/acsnano.5c19929)
Supplement: Supplementary file 1 [file nn5c19929_si_001.pdf]

Supporting Information

Isoreticular Tuning of Conductive Metal–Organic Framework Nanocrystals for the Rapid Detection and Differentiation of Toxic Gases

Elissa O. Shehayeb,[a] Joseph Y. M. Chan,[a] Doran L. Pennington,[b] Christopher H. Hendon,[b] and Katherine A. Mirica[a]*

a. Department of Chemistry, Burke Laboratory, Dartmouth College, Hanover, New Hampshire, 03755, United States

b. Department of Chemistry and Biochemistry, University of Oregon, Eugene, Oregon 97403, United States

*Email: Katherine.A.Mirica@dartmouth.edu

Table of Contents

S1. Materials and Methods 2

S2. MOF synthesis 3

S3. Characterization of MTPz-Cu-MOFs 20

S3.1. Microscopy images of MTPz-Cu-MOF 20

S3.2. Elemental Analysis of MTPz-Cu-MOFs 25

S3.3. ATR-FTIR spectra 28

S3.4. XPS spectra 29

S3.5. EPR plots 31

S3.6. Conductivity measurements 31

S3.7. BET isotherms and surface areas 34

S3.8. Optical band gaps of pristine MOFs 35

S4. Computational studies 36

S5. Sensing experiments of MTPz-Cu-MOFs 38

S5.1. Preparation of sensing devices 38

S5.2. Sensing experiment setup 39

S5.3. H_2S gas sensing 41

S5.4. NH_3 gas sensing 44

S5.5. SO_2 gas sensing 47

S5.6. NO gas sensing 50

S5.7. Limits of Detection (LODs) 53

S5.8. Initial Rates 58

S5.9. Recyclability Tests 63
S5.10. Recovery Calculations 71
S5.11. Comparison to MPz, MPc, and MNPc MOFs 73
S6. Principal Component Analysis 73
S7. Diffuse Reflectance Infrared Fourier Transform Spectroscopy (DRIFTS) 78
S8. Characterization after exposure to gases 91
S9. Optical Band Gaps Upon Gas Exposure 102
S10. Electrochemical impedance spectroscopy 103
S11. Suspension characterization 107
S12. References 109

S1. Materials and Methods

Unless otherwise specified, all materials were purchased from commercial sources (Millipore Sigma, Thermo Fischer Scientific, Ambeed, or Beantown Chemicals) and used without further purification. NMR spectra were collected on a Bruker 600 MHz NMR spectrometer. Mass Spectra were carried out on a Waters Synapt G2-Si Electron Spray Ionization (ESI) Mass Spectrometer or a Bruker Autoflex Speed LRF Matrix-Assisted Laser Desorption Ionization (MALDI) spectrometer. Powder X-ray Diffraction (PXRD) diffraction patterns were recorded on a Rigaku sixth generation MiniFlex X-ray diffractometer with a Cu Kα (600 W, 40 kV, 15 mA, α = 1.54 Å) radiation source. Scanning Electron Microscopy (SEM) images were taken on a Thermo Scientific™ Helios™ 5 CX DualBeam microscope. Energy Dispersive X-ray (EDX) spectra were recorded using an Ultim Extreme 100 Oxford Instrument X-ray detector. Transmission Electron Microscopy (TEM) images were collected on a Thermo Scientific Talos F200i (S)TEM microscope. CHN elemental analysis data were performed using combustion by Atlantic Microlab, Inc. Fourier Transform – Infrared (FTIR) spectra and Diffuse Reflectance Infrared Fourier Transform Spectroscopy (DRIFTS) data were acquired using a Nicolet 6700 FT-IR spectrometer. Nitrogen adsorption experiments for BET isotherms were conducted on an ASAP Plus 2020 3Flex (Micrometrics, Norcross, Georgia) instrument at 77K. Thermogravimetric Analysis (TGA) were recorded on a TGA 55 instrument from 25 to 900 °C with a ramp rate of 5 °C/min under nitrogen or air as purge gases. Bulk conductivity measurements were collected on pressed pellets of the materials using a Signatone tungsten carbide four-point linear probe. Proton conductivity and electrochemical impedance spectroscopy measurements were carried out on a Gamry Potentiostat Interface 1010E. Inductively Coupled Plasma – Mass Spectrometry (ICP-MS) experiments were performed on 8900 Triple Quadrupole ICP-QQQ-MS. X-ray photoelectron spectroscopy (XPS) experiments were performed on a Physical Electronics 5000 VersaProbe II Scanning ESCA Microprobe X-ray/Ultraviolet Photoelectron Spectrometer. Electron Paramagnetic Resonance (EPR) spectra were acquired using a Bruker BioSpin Gmbh spectrometer equipped with a standard mode cavity. UV–Vis–NIR spectra were performed on a JASCO V-570 S13 spectrophotometer between 400 and 2000 nm at the scan rate of 400 nm/min under ambient conditions. Dynamic light scattering measurements were performed on a DynaPro NanoStar™, while zeta potential measurements were collected on a Malvern ZetaSizer Nano. Sensing experiments were performed using suspensions of the materials in water dropcasted on interdigitated gold electrodes with 5 μm or 10 μm gaps purchased from Metrohm (G-IDEAU5 or

G-IDEAU10). Details of the sensing setup are described in **Section S5**, where analyte gases (hydrogen sulfide, nitric oxide, sulfur dioxide, ammonia, and carbon monoxide) are purchased from Airgas as tanks constituting 10,000 ppm gas analyte in nitrogen, and Micro-Trak and Smart-Trak mass flow controllers are acquired from Sierra Instruments, Inc. Material Studio, VESTA, and ImageJ softwares were used to acquire the molecular structure and analysis images.

S2. MOF synthesis

The metallo-tetrapyrazinoporphyrazine (MTPz) ligands were synthesized similar to our previously reported procedure for DC-100.[1]

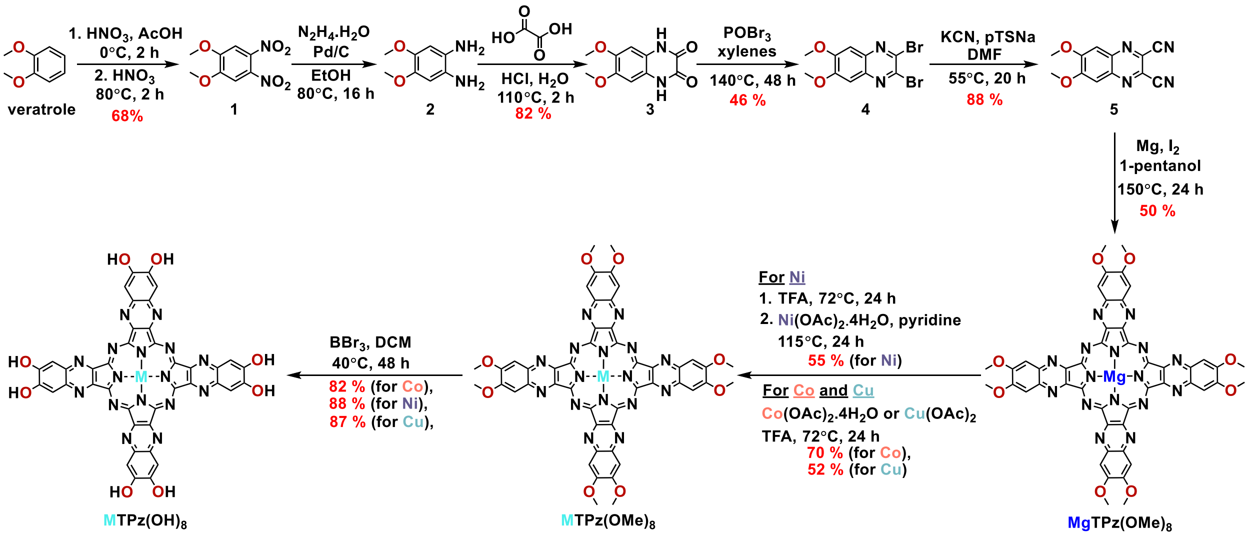

Scheme S1. Synthesis of $MTPz(OH)_8$.

1,2-dimethoxy-4,5-dinitrobenzene (1). The title compound was synthesized according to a modified literature procedure.[2] Veratrole (25 mL, 0.195 mol) and acetic acid (AcOH, 25 mL) were stirred in a round-bottom flask in an ice bath. Concentrated nitric acid (10 mL) was added dropwise using an addition funnel for a period of around 10 minutes, and the reaction mixture was allowed to stir in the ice bath for 2 hours. Then, it was poured over a beaker containing ice-water using acetone to transfer all the product. Excess distilled water was added to crash out the product, which was collected by suction filtration. After drying, the solid was transferred into a new round-bottom flask to which 25 mL of concentrated nitric acid were added. The reaction mixture was heated at 80°C for 2 hours (notice evolution of NO_2 gas which should be neutralized in a base

bath, as well as precipitation of yellow solid). The reaction mixture was cooled to room temperature and poured over a beaker containing ice-water similar to the procedure described above. The precipitate was collected by suction filtration and allowed to dry under vacuum overnight (30.2 g, 68%). ^{1}H-NMR (600 MHz, $CDCl_3$, ppm): 7.34 (s, 2H) and 4.02 (s, 6H).

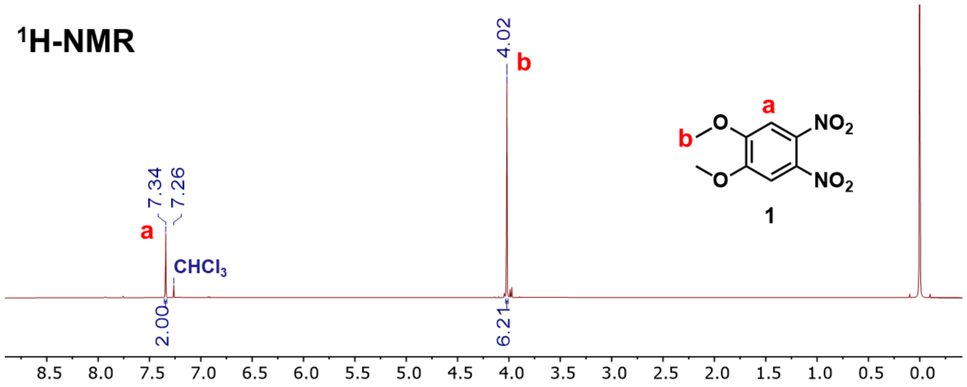

Figure S1. ^{1}H-NMR spectrum of **1** in $CDCl_3$ at 600 MHz.

4,5-dimethoxybenzene-1,2-diamine (2). The title compound was synthesized according to a modified literature procedure.[2] In a 250 mL round-bottom flask, **1** (6 g, 26.3 mmol) and 200 mg of 10 wt% palladium on activated carbon were suspended in 50 mL of ethanol. A reflux setup with continuous nitrogen gas flow was assembled directly upon the addition of 10 mL of hydrazine monohydrate, and the reaction mixture was stirred and heated at 80°C overnight (notice change in color from red to dark yellow to colorless). The reaction was cooled to room temperature while still under nitrogen and then filtered under vacuum in a fine-sintered funnel over 6 mL of concentrated hydrochloric acid. The filtrate was evaporated under reduced pressure until a very concentrated suspension remains. The product was directly used in the next step without further purification or characterization.

6,7-dimethoxy-1,4-dihydroquinoxaline-2,3-dione (3). The title compound was synthesized according to a previously reported literature procedure.[3] To the round-bottom flask containing **2**, oxalic acid dihydrate (9 g, 100 mmol), 50 mL of distilled water, and 2 mL of hydrochloric acid were

added. The reaction mixture was stirred and refluxed at 110°C for 2 hours during which a wine-red solid was precipitated out of the solution. After cooling to room temperature, the product was collected by suction filtration and dried under vacuum overnight (4.77 g, 82%). ^{1}H-NMR (600 MHz, DMSO-d_6, ppm): 11.72 (s, 2H), 6.73 (s, 2H), and 3.72 (s, 6H). ^{13}C-NMR (150 MHz, DMSO-d_6, ppm): 155.39, 145.60, 119.12, 99.95, 56.27.

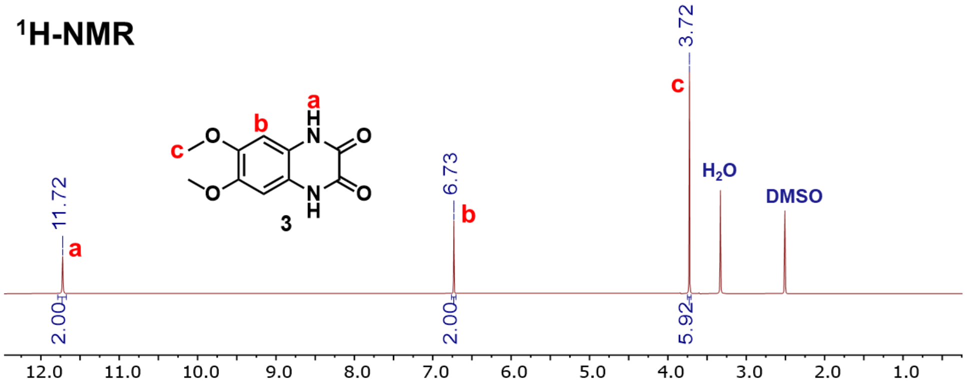

Figure S2. ^{1}H-NMR spectrum of **3** in DMSO-d_6 at 600 MHz.

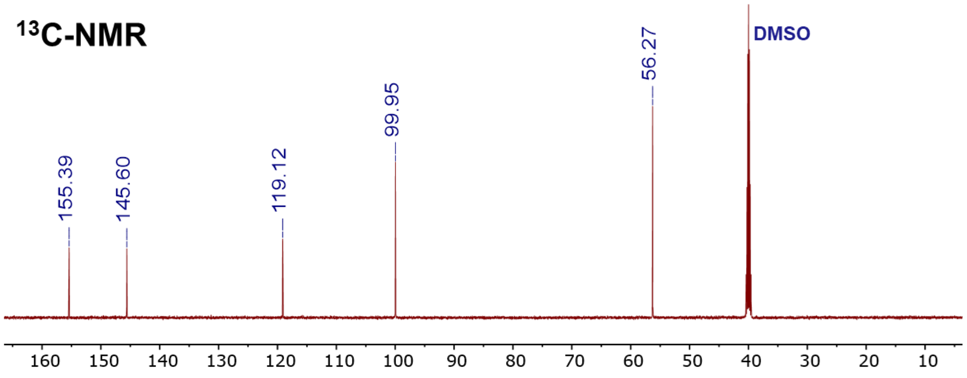

Figure S3. ^{13}C-NMR spectrum of **3** in DMSO-d_6 at 150 MHz.

2,3-dibromo-6,7-dimethoxyquinoxaline (4). A round-bottom flask was charged with **3** (1.11 g, 5.00 mmol, 1 eq), phosphoryl bromide (3.3 g, 11.5 mmol, 2.3 eq), and 25 mL of xylenes mixture. It was assembled into a reflux setup with a nitrogen gas flow input and an outflow of gas into a base bath (to neutralize the exerted $HBr_{(g)}$). The reaction was stirred and refluxed at 140°C for 48 hours. The reaction mixture was then cooled to room temperature and poured over a beaker

containing ice. A sodium hydroxide solution was added to make the medium basic. The product was dissolved and extracted from water with dichloromethane (4 × 100 mL). The organic layers were then combined, dried over magnesium sulfate, and evaporated under reduced pressure. The resulting sticky solid was washed with a minimal amount of hexane and filtered to afford the pure product (0.8 g, 46%). ^{1}H-NMR (600 MHz, $CDCl_3$, ppm): 7.29 (s, 2H) and 4.04 (s, 2H). ^{13}C-NMR (150 MHz, $CDCl_3$, ppm): 153.82, 138.64, 137.72, 105.79, 56.57. HRMS (ESI): $C_{10}H_9Br_2N_2O_2$, $[M+H]^+$ m/z calculated: 348.9010, found: 348.9012.

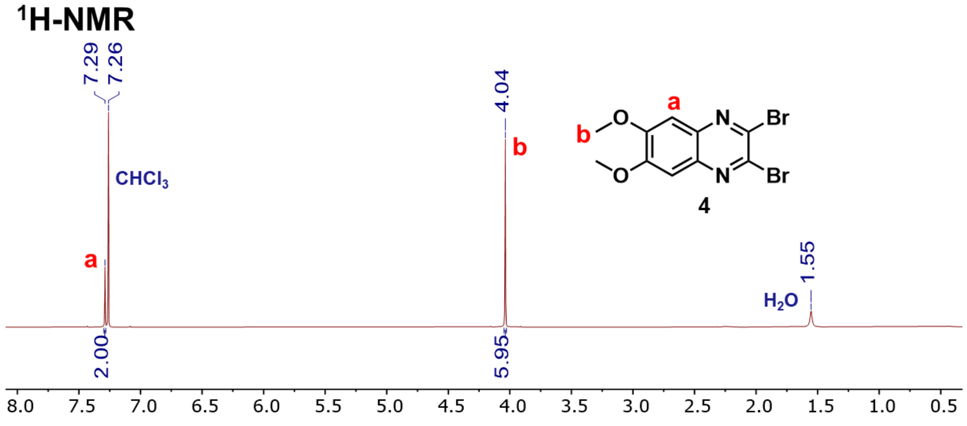

Figure S4. ^{1}H-NMR spectrum of **4** in $CDCl_3$ at 600 MHz.

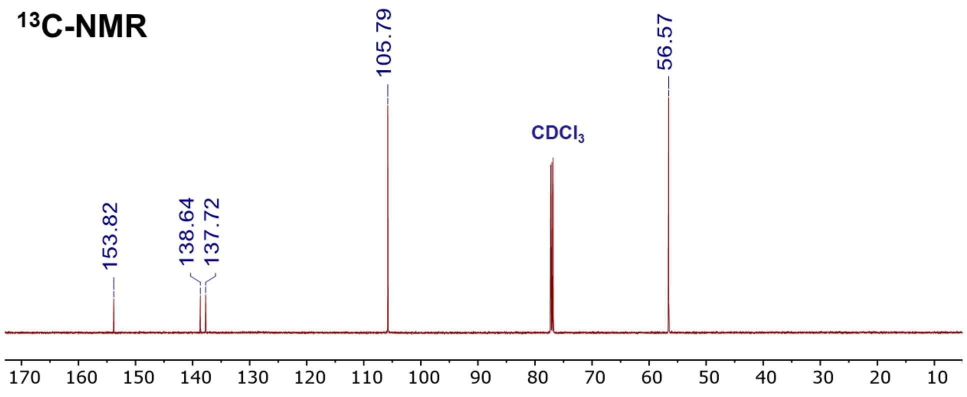

Figure S5. ^{13}C-NMR spectrum of **4** in $CDCl_3$ at 150 MHz.

6,7-dimethoxyquinoxaline-2,3-dicarbonitrile (5). The solid reactants: **4** (0.5 g, 1.44 mmol, 1 eq), potassium cyanide (0.428 g, 6.58 mmol, 4.6 eq), and *p*-toluene sulfinic acid sodium salt

(0.254 g, 1.43 mmol, 1 eq) were added into a Schlenk tube with a stir bar and degassed by three vacuum-nitrogen cycles. Under nitrogen, 1 mL of anhydrous *N*,*N*-dimethylformamide (DMF) was added using a syringe. The reaction mixture was heated at 55 °C under nitrogen for 20 hours. Then, the reaction was quenched with distilled water, the solid was collected by suction filtration. The pure product was obtained after drying the solid in a vacuum oven overnight (0.30 g, 88%). ^{1}H-NMR (600 MHz, $CDCl_3$, ppm): 7.41 (s, 2H) and 4.13 (s, 2H). ^{13}C-NMR (150 MHz, $CDCl_3$, ppm): 157.18, 140.48, 128.23, 114.02, 106.36, 57.16. HRMS (ESI): $C_{12}H_9N_4O_2$, $[M+H]^+$ m/z calculated: 241.0681, found: 241.0721.

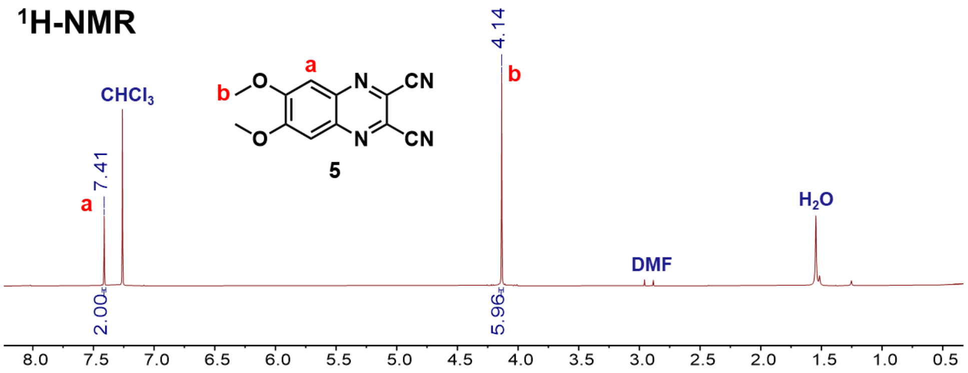

Figure S6. ^{1}H-NMR spectrum of **5** in $CDCl_3$ at 600 MHz.

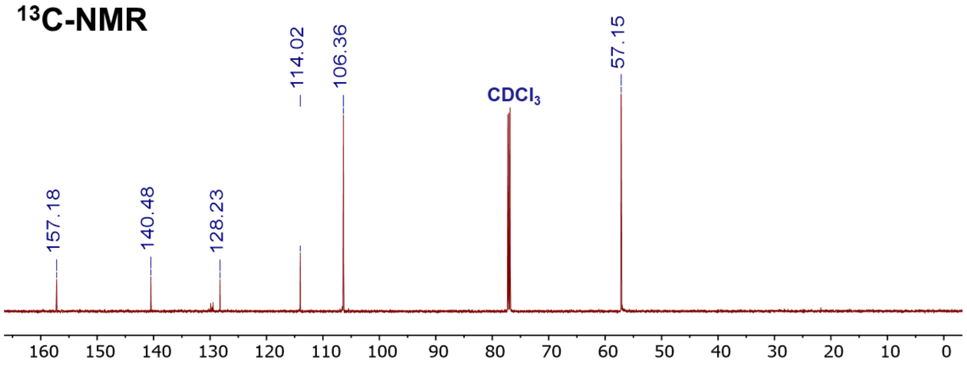

Figure S7. ^{13}C-NMR spectrum of **5** in $CDCl_3$ at 150 MHz.

MgTPz(OMe)₈. In a 3-neck round-bottom flask, magnesium turnings (82 mg, 3.37 mmol, 4 eq) previously activated by a dilute hydrochloric acid solution were added with a stir bar. After degassing with three vacuum-nitrogen cycles, 6 mL of 1-pentanol and a crystal of sublimed iodine were added, and the reaction mixture was heated at 130 °C under nitrogen for at least 10 hours until a thick suspension of colorless crystals is observed. Under nitrogen, **5** (0.2 g, 0.832 mmol, 1 eq) was added, and the reaction mixture was further heated to 150 °C for 18 hours. It was then cooled to room temperature and suspended in ethyl acetate, and the solid was washed with methanol and acetone. The remaining precipitate was dried in a vacuum oven overnight to afford the pure product (101 mg, 49%). ^{1}H-NMR (600 MHz, CF_3COOD, ppm): 8.41 (s, 8H) and 4.48 (s, 24H). ^{13}C-NMR spectrum could not be obtained due to poor solubility in common solvents. HRMS (MALDI): $C_{48}H_{34}NaN_{16}O_8$, $[M]^+$ m/z calculated: 985.2643, found: 985.2489.

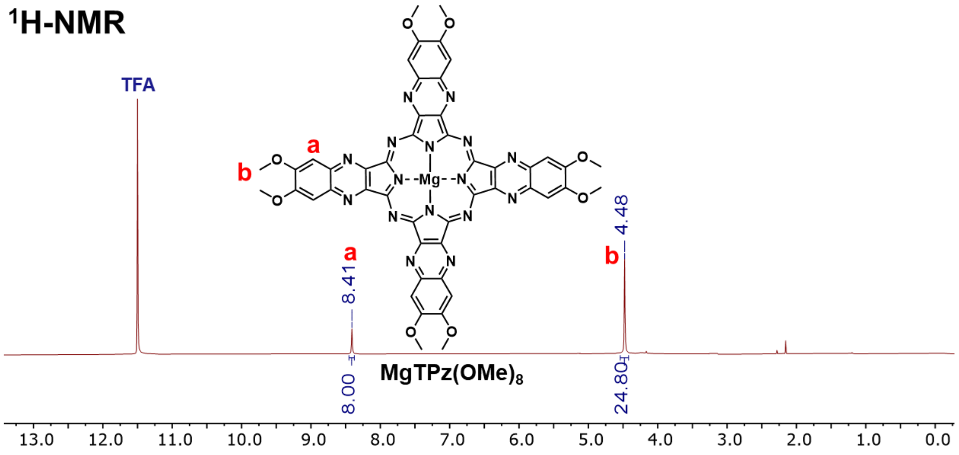

Figure S8. ^{1}H-NMR spectrum of **MgTPz(OMe)₈** in CF_3COOD at 600 MHz.

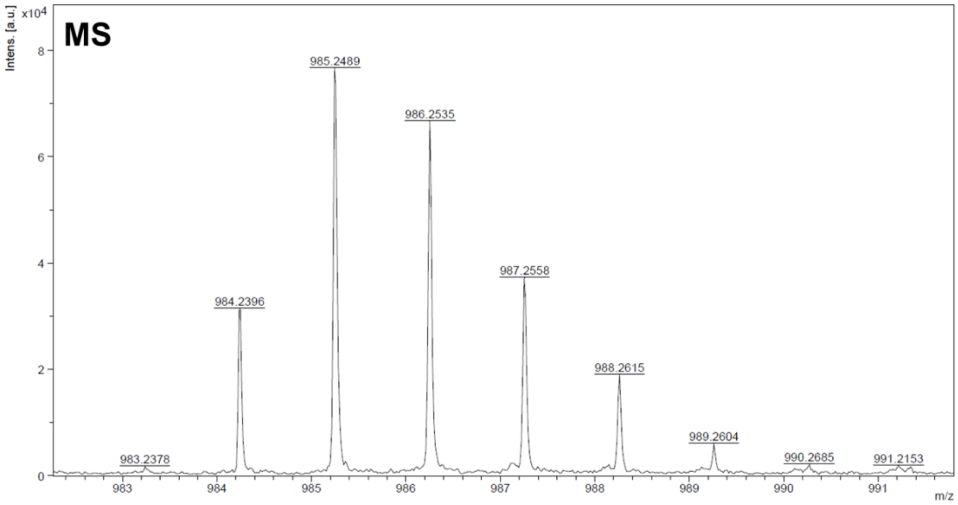

Figure S9. High-resolution mass spectrum (MALDI) of **MgTPz(OMe)$_8$**.

CoTPz(OMe)$_8$. In a 3-neck round-bottom flask was charged with **MgTPz(OMe)$_8$** (100 mg, 0.101 mmol, 1 eq), cobalt (II) acetate tetrahydrate (100 mg, 0.40 mmol, 4 eq), and a stir bar. After its assembly in a reflux setup, the solids were degassed by three vacuum-nitrogen cycles. Trifluoroacetic acid (8 mL) was added under nitrogen, and the reaction mixture was refluxed at 72 °C overnight. The resulting solution was evaporated, and the remaining solid was washed with water and acetone in a centrifuge tube. The precipitate was dried in a vacuum oven to afford the pure dark blue product (73 mg, 70%). ^{1}H-NMR (600 MHz, CF_3COOD, ppm): 8.59 (s, 8H) and 3.92 (s, 24H). ^{13}C-NMR spectrum could not be obtained due to poor solubility in common solvents. HRMS (MALDI): $C_{48}H_{32}CoN_{16}O_8$, $[M]^+$ m/z calculated = 1019.1921, found = 1019.1915.

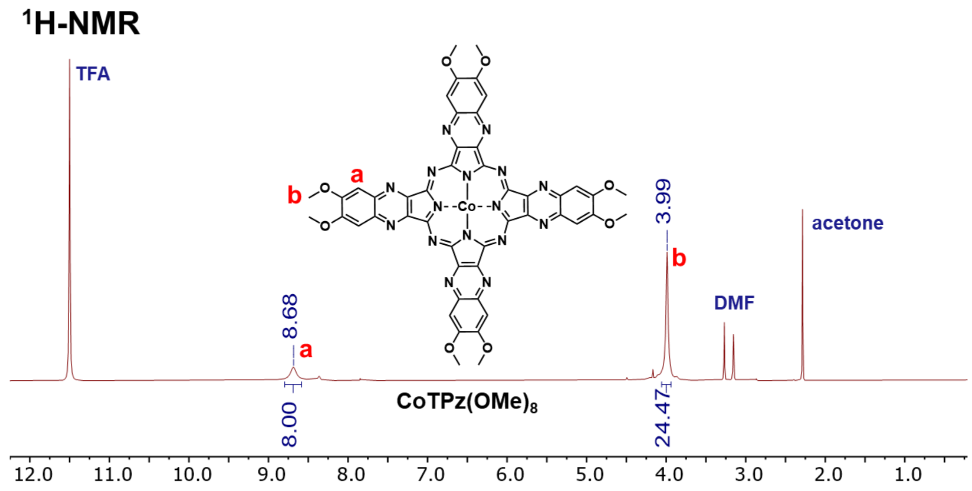

Figure S10. ^{1}H-NMR spectrum of **CoTPz(OMe)$_8$** in CF_3COOD at 600 MHz.

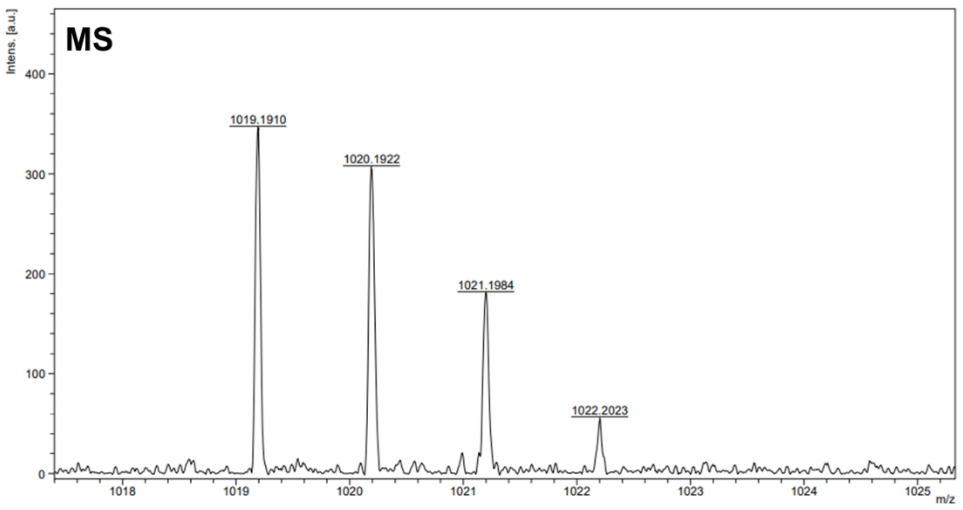

Figure S11. High-resolution mass spectrum (MALDI) of **CoTPz(OMe)$_8$**.

CoTPz(OH)$_8$. In a 3-neck round-bottom flask fitted into a reflux setup and connected to the Schlenk line, **CoTPz(OMe)$_8$** (220 mg, 0.216 mmol, 1 eq) was added with a magnetic stirrer. The solid was degassed with three vacuum-nitrogen cycles, and under nitrogen, 4.5 mL of anhydrous dichloromethane (DCM) were added followed by the dropwise addition of boron tribromide (1.03 mL, 10.8 mmol, 50 eq). The reaction mixture was stirred at reflux under nitrogen for 40 hours. After cooling to room temperature, the reaction mixture was poured onto ice with methanol, and

the resulting suspension was centrifuged and decanted. The obtained solid was then washed, centrifuged, and decanted successively with methanol and acetone. The solid was then dissolved in 15 mL of DMF, followed by its precipitation in 400 mL of water. The precipitate was collected by suction filtration, washed with acetone, and dried under vacuum to obtain the pure dark bluish-green powder (160 mg, 82%). ^{1}H-NMR (600 MHz, DMSO-d_6, ppm): 11.67 (s, 8H) and 8.61 (s, 8H). ^{13}C-NMR spectrum could not be obtained due to poor solubility in common solvents. HRMS (MALDI): $C_{40}H_{17}CoN_{16}O_8$, $[M+H]^+$ m/z calculated = 908.0703, found = 908.0754.

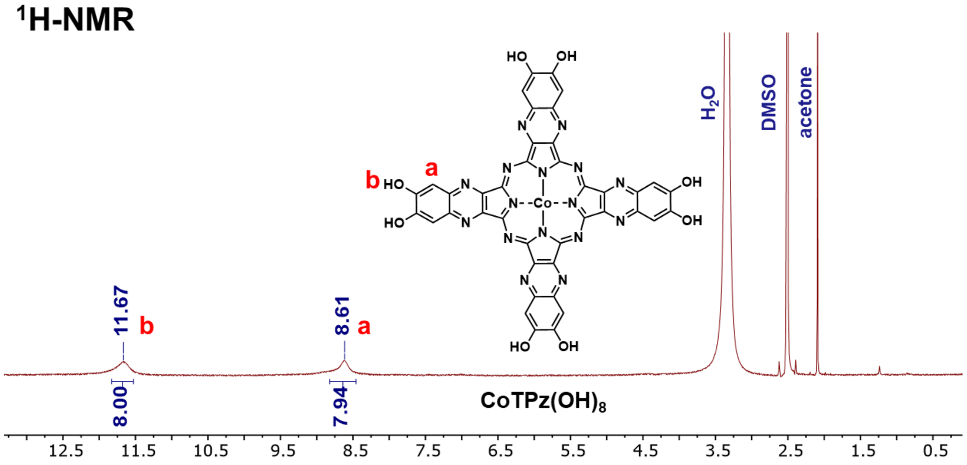

Figure S12. ^{1}H-NMR spectrum of **CoTPz(OH)$_8$** in DMSO-d_6 at 600 MHz.

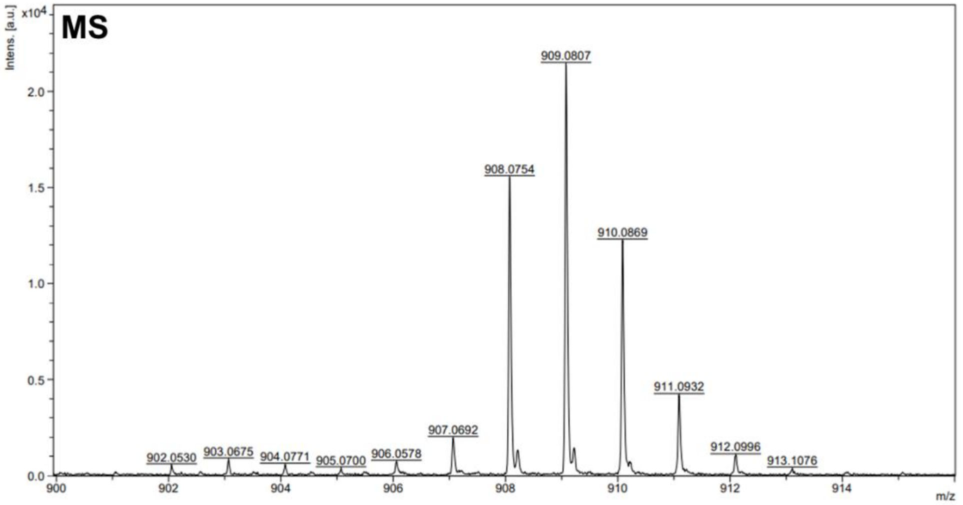

Figure S13. High-resolution mass spectrum (MALDI) of **CoTPz(OH)$_8$**.

NiTPz(OMe)₈. A 3-neck round-bottom flask was charged with **MgTPz(OMe)₈** (200 mg, 0.20 mmol, 1 eq) and a stir bar. After its assembly in a reflux setup, the solids were degassed by three vacuum-nitrogen cycles. Trifluoroacetic acid (10 mL) was added under nitrogen, and the reaction mixture was refluxed at 72 °C overnight. The solvent was evaporated under reduced pressure, and the resulting solid was directly reacted with nickel (II) acetate tetrahydrate (200 mg, 0.80 mmol, 4 eq) in pyridine (10 mL) and heated at reflux overnight. Then, the solvent was evaporated under reduced pressure, and the obtained solid was washed, centrifuged, and decanted with methanol (3 × 15 mL) then acetone (3 × 15 mL) and dried in a vacuum oven overnight to afford the pure dark green solid (114 mg, 55%). ^{1}H-NMR (600 MHz, CF_3COOD, ppm): 8.36 (s, 8H) and 4.48 (s, 24H). ^{13}C-NMR spectrum could not be obtained due to poor solubility in common solvents. HRMS (MALDI): $C_{48}H_{33}N_{16}NiO_8$, $[M+H]^+$ m/z calculated = 1019.1976, found = 1019.1977.

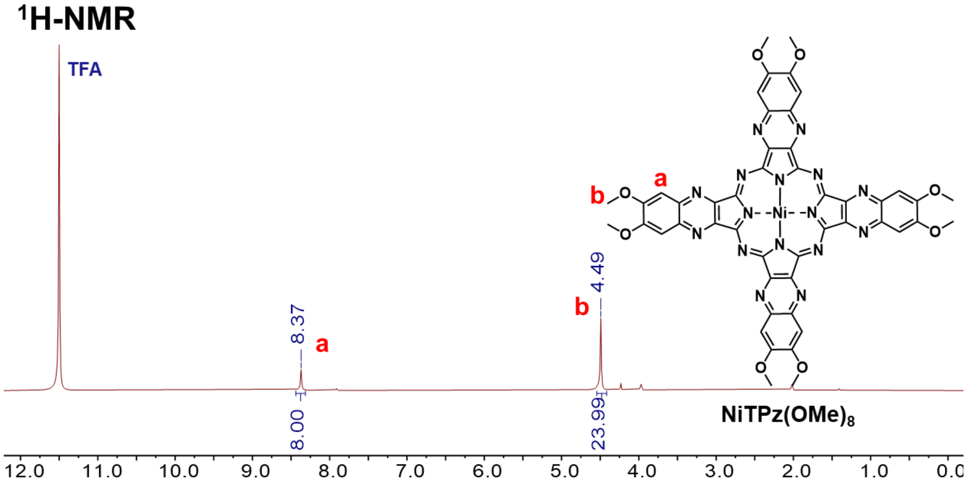

Figure S14. ^{1}H-NMR spectrum of **NiTPz(OMe)₈** in CF_3COOD at 600 MHz.

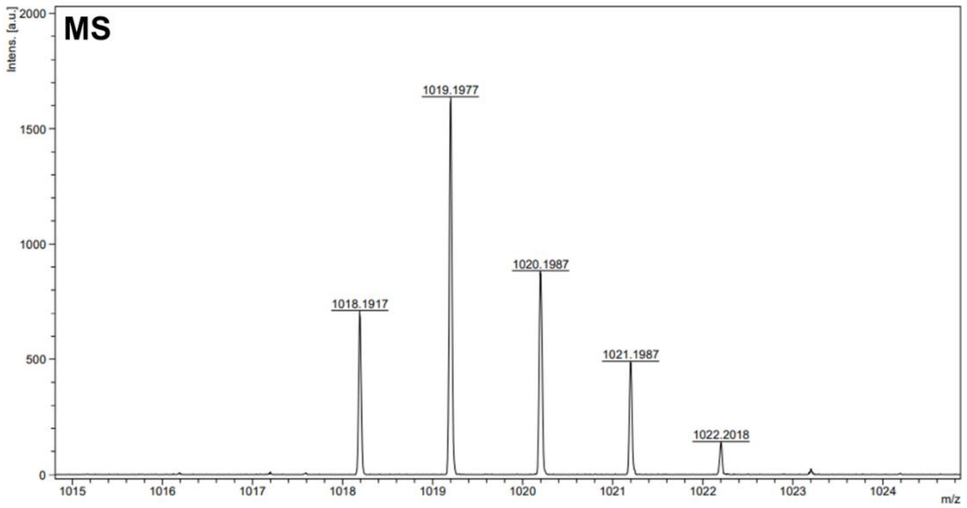

Figure S15. High-resolution mass spectrum (MALDI) of **NiTPz(OMe)$_8$**.

NiTPz(OH)$_8$. In a 3-neck round- bottom flask fitted into a reflux setup and connected to the Schlenk line, **NiTPz(OMe)$_8$** (100 mg, 0.098 mmol, 1 eq) was added with a magnetic stirrer. The solid was degassed with three vacuum-nitrogen cycles, and under nitrogen, 6.5 mL of anhydrous DCM were added followed by the dropwise addition of boron tribromide (0.75 mL, 7.85 mmol, 80 eq). The reaction mixture was stirred at reflux under nitrogen for 40 hours. After cooling to room temperature, the reaction mixture was poured onto ice with methanol, and the resulting suspension was centrifuged and decanted. The obtained solid was then washed, centrifuged, and decanted with water (3 × 15 mL) and acetone (3 × 25 mL), successively, and dried under vacuum to obtain the pure dark green powder (75 mg, 88%). ^{1}H-NMR (600 MHz, D_2SO_4, ppm): 9.32 (s, 8H). ^{13}C-NMR spectrum could not be obtained due to poor solubility in common solvents. HRMS (MALDI): $C_{40}H_{17}N_{16}NiO_8$, [M+H]$^+$ m/z calculated = 907.0724, found = 907.0762.

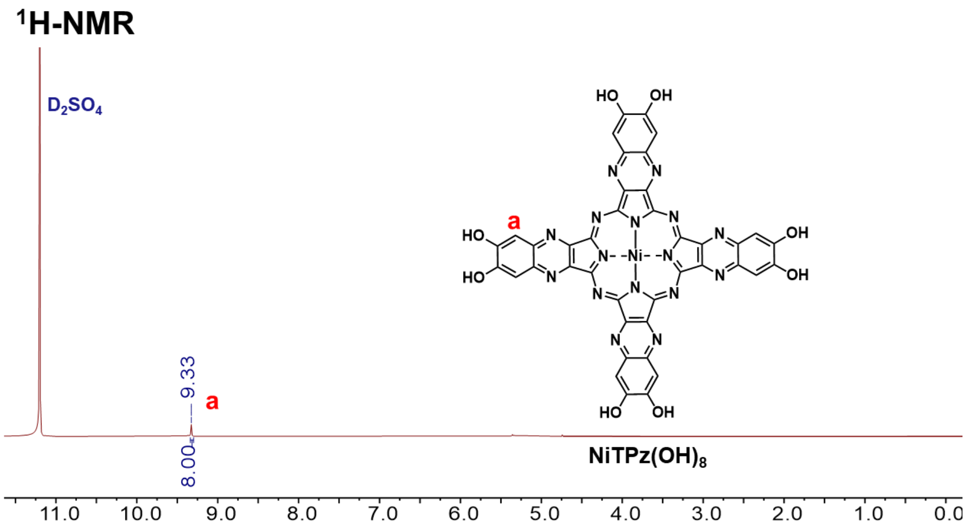

Figure S16. ¹H-NMR spectrum of **NiTPz(OH)₈** in D_2SO_4 at 600 MHz.

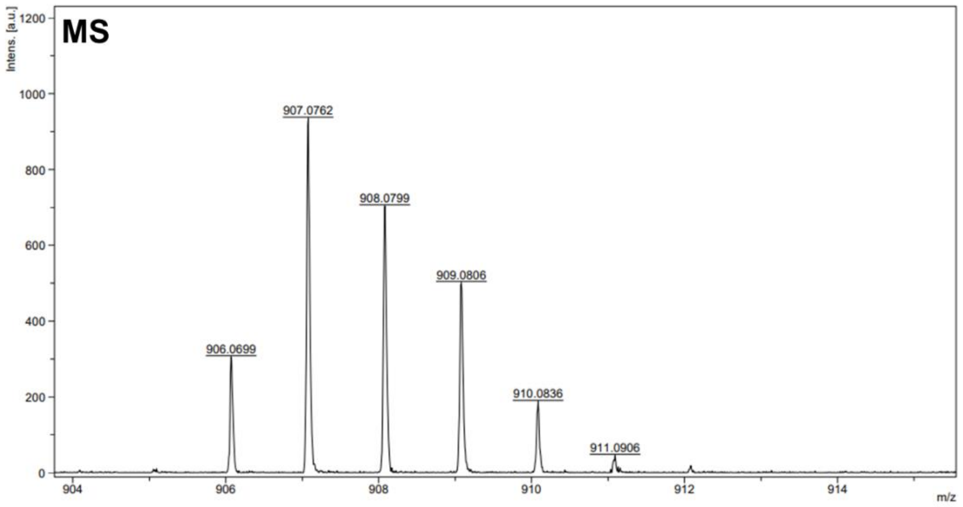

Figure S17. High-resolution mass spectrum (MALDI) of **NiTPz(OH)₈**.

CuTPz(OMe)₈. A 3-neck round-bottom flask was charged with **MgTPz(OMe)₈** (200 mg, 0.2 mmol, 1 eq), copper (II) acetate (200 mg, 0.40 mmol, 4 eq), and a stir bar. After its assembly in a reflux setup, the solids were degassed by three vacuum-nitrogen cycles. Trifluoroacetic acid (10 mL) was added under nitrogen, and the reaction mixture was refluxed at 72 °C overnight. The resulting solution was evaporated, and the remaining solid was washed with i) water (3 × 25 mL), ii) acetone (3 × 25 mL), and iii) tetrahydrofuran (3 × 25 mL) in a centrifuge tube. The precipitate was dried in

a vacuum oven to afford the pure dark green product (108 mg, 52 %). ^{1}H-NMR (600 MHz, CF_3COOD, ppm): 8.66 (s, 8H) and 4.23 (s, 24H). ^{13}C-NMR spectrum could not be obtained due to poor solubility in common solvents. HRMS (MALDI): $C_{48}H_{32}CuN_{16}O_8$ $[M+H]^+$ m/z calculated = 1024.1963, found = 1024.1928.

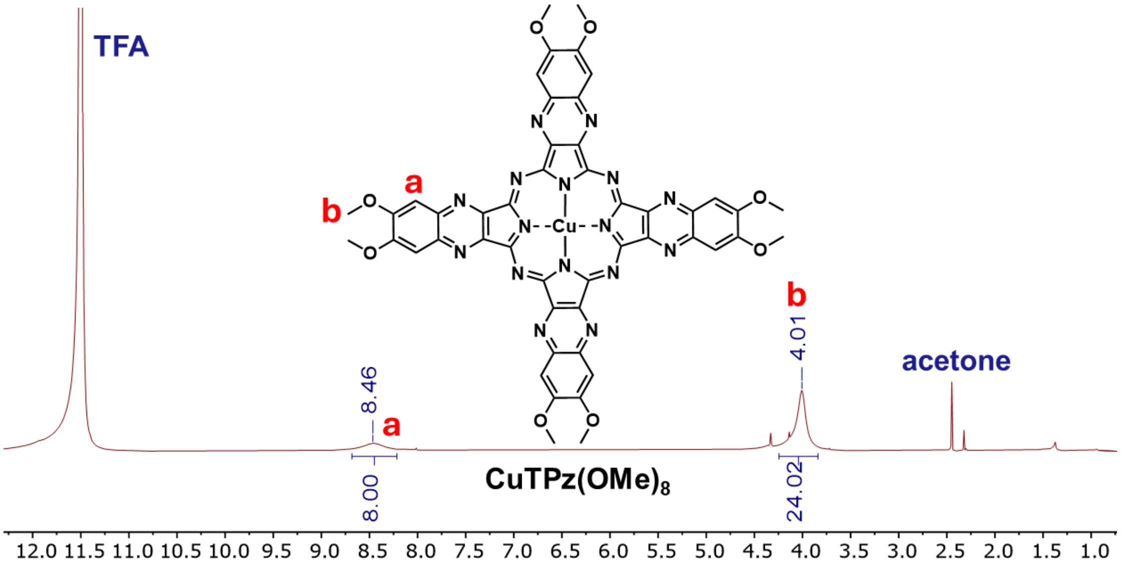

Figure S18. ^{1}H-NMR spectrum of $CuTPz(OMe)_8$ in CF_3COOD at 600 MHz.

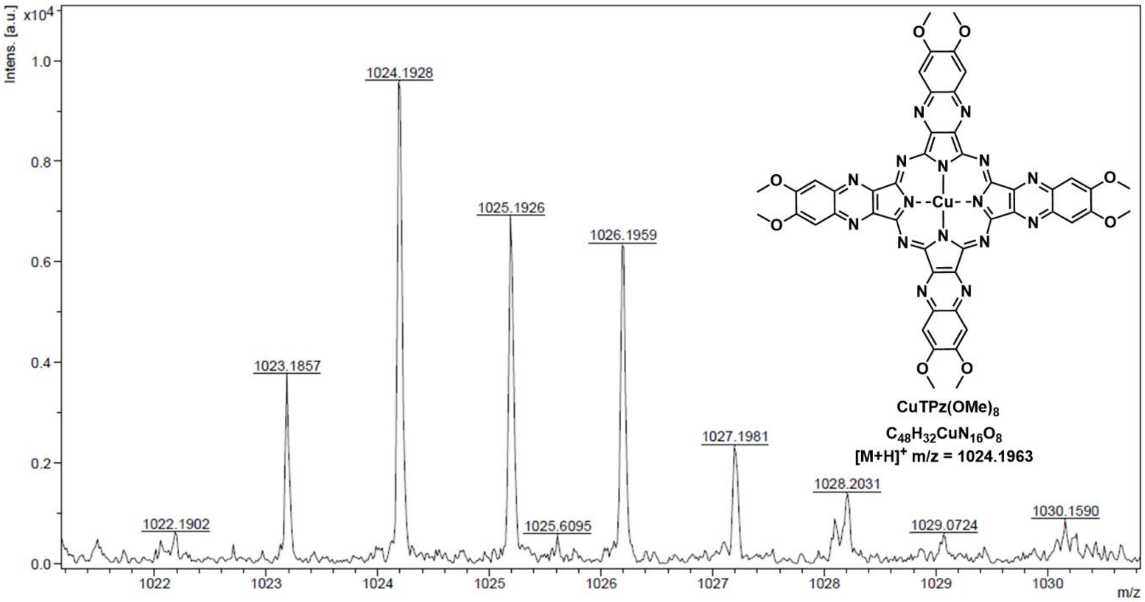

Figure S19. High-resolution mass spectrum (MALDI) of $CuTPz(OMe)_8$.

Synthesis of CuTPz(OH)$_8$. In a three-neck round bottom flask fitted into a reflux setup and connected to the Schlenk line, **CuTPz(OMe)$_8$** (100 mg, 0.098 mmol, 1 eq) was added with a magnetic stirrer. The solid was degassed with three vacuum-nitrogen cycles, and under nitrogen, 6.5 mL of anhydrous DCM were added followed by the dropwise addition of boron tribromide (0.75 mL, 7.85 mmol, 80 eq). The reaction mixture was stirred at reflux under nitrogen for 40 hours. After cooling to room temperature, the reaction mixture was poured onto ice with methanol, and the resulting suspension was centrifuged and decanted. The obtained solid was then washed, centrifuged, and decanted successively with water (3 × 15 mL) and acetone (3 × 25 mL), successively, and dried under vacuum to obtain the pure dark green powder (74 mg, 87 %). ^{1}H-NMR and ^{13}C-NMR spectra could not be obtained due to the paramagnetic nature of Cu^{2+} ions. HRMS (MALDI): $C_{40}H_{17}CuN_{16}O_8$ $[M+H]^+$ m/z calculated = 912.0711, found = 912.0688.

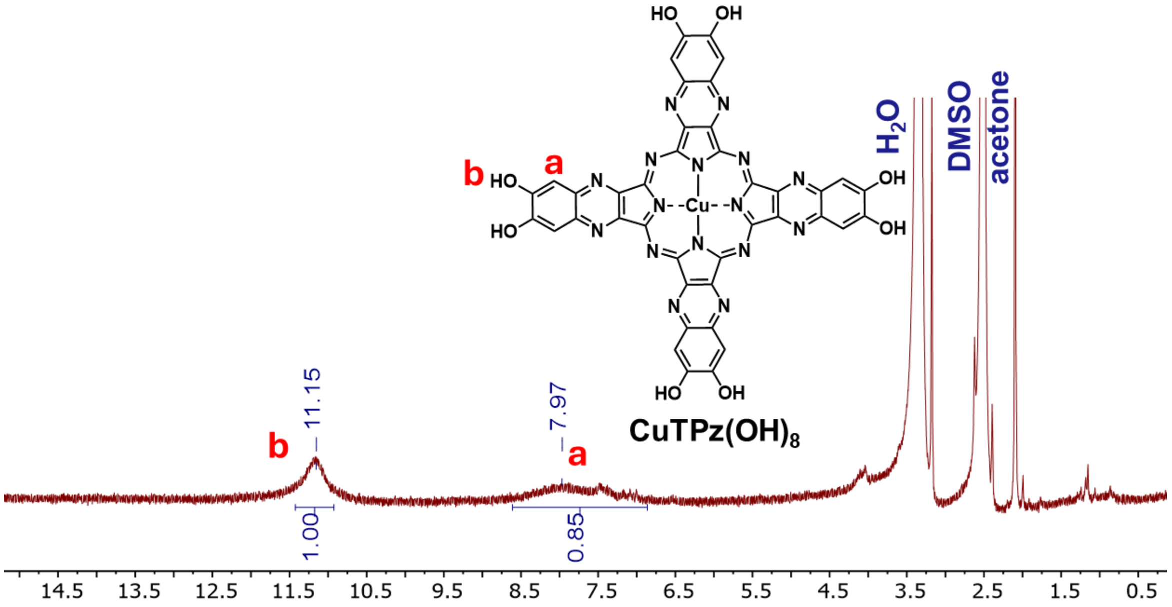

Figure S20. ^{1}H-NMR spectrum of CuTPz(OH)$_8$ in DMSO-d_6 at 600 MHz.

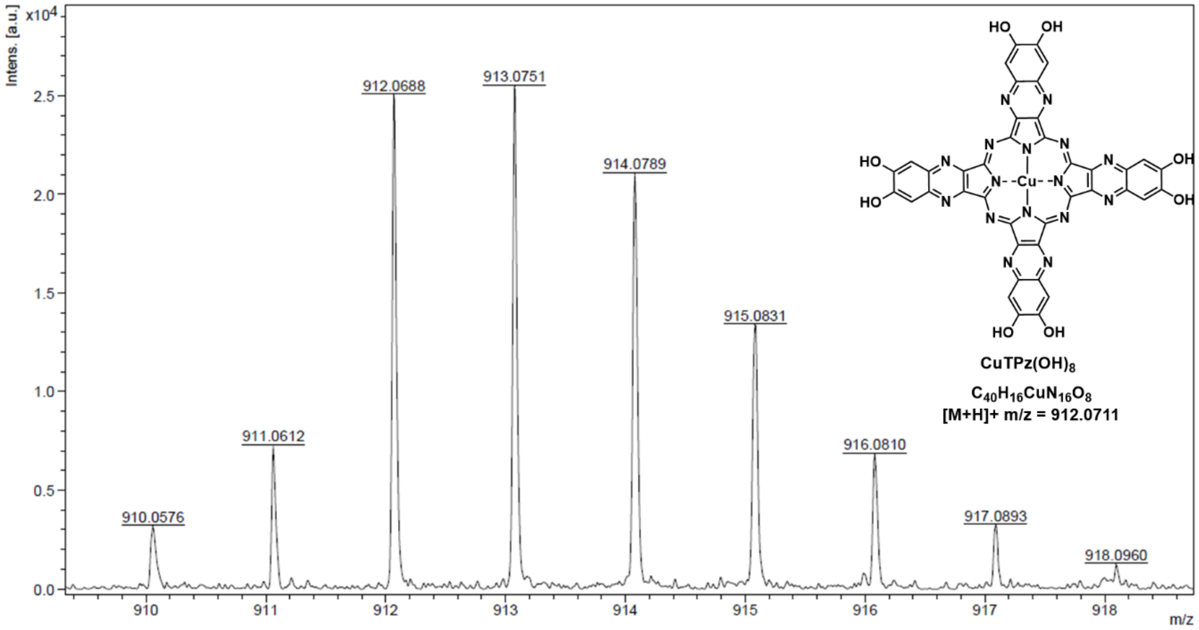

Figure S21. High-resolution mass spectrum (MALDI) of CuTPz$(OH)_8$.

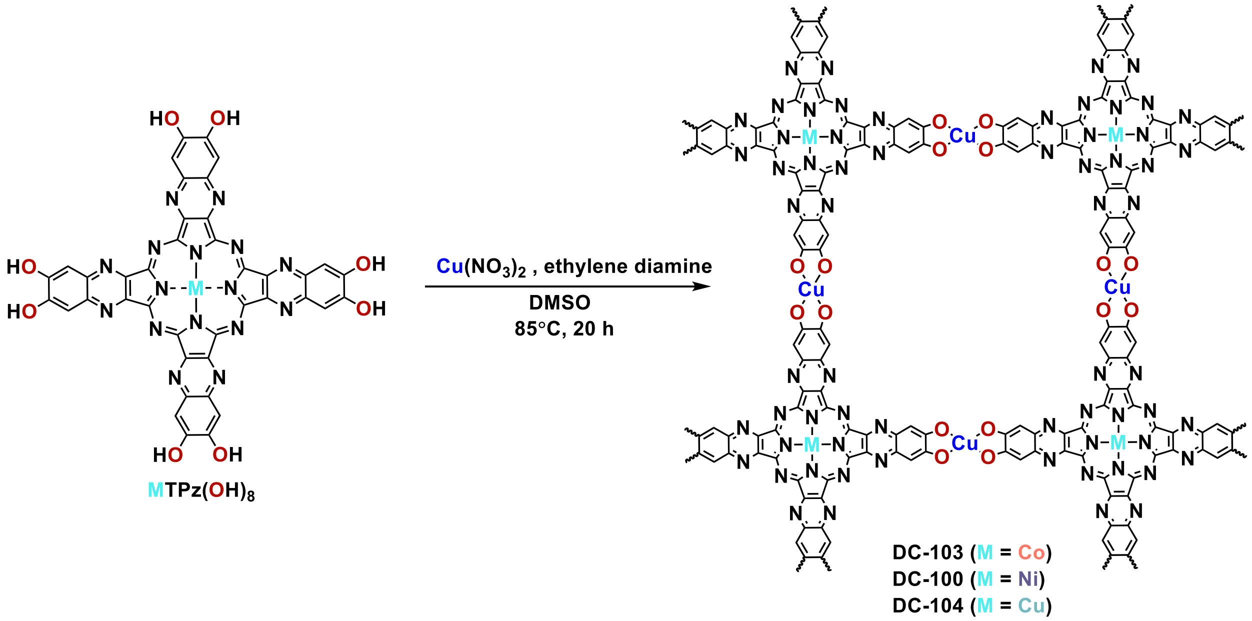

Scheme S2. Synthesis of MTPz-Cu-MOF analogues.

MTPz-Cu-MOF (General Synthesis). MTPz$(OH)_8$ (10 mg, 0.011 mmol, 1 eq) was charged into a high-pressure Schlenk flask and subjected to three vacuum-nitrogen cycles. Under nitrogen, 24

mL of anhydrous dimethylsulfoxide (DMSO) were added, then the tube was sealed and sonicated for 5 minutes. Meanwhile, a 23 mM solution of copper(II) nitrate hemi(pentahydrate), $Cu(NO_3)_2{\cdot}2.5H_2O$, (5.38 mg, 0.023 mmol, 2.1 eq) in 1 mL of anhydrous DMSO was prepared. The reaction vessel was reconnected to argon, and it was opened to add the salt solution followed by ethylene diamine (variable amount for each analogue, see below). The Schlenk tube was sealed and placed in a preheated oven at 85 °C for 15 hours. After cooling to room temperature, the light brown solution with black precipitate was transferred to a centrifuge tube, and the resulting solid was washed and decanted successively with 25 mL of DMF, water, and acetone. The MOF bulk powder was dried under vacuum overnight, and their crystallinities were confirmed by their PXRD diffraction patterns, shown in **Figure 1b**. The MOFs were activated by soaking them for 24 hours in each of DMSO (2 × 20 mL) and THF (1 × 20 mL), followed by drying in a vacuum oven at 60 °C overnight.

Synthesis of DC-103. CoTPz(OH)$_8$ (10 mg, 0.011 mmol, 1 eq), $Cu(NO_3)_2{\bullet}2.5H_2O$ (5.38 mg, 0.023 mmol, 2.1 eq), ethylene diamine (0.29 mL, 4.4 mmol, 400 eq).

Synthesis of DC-100. NiTPz(OH)$_8$ (10 mg, 0.011 mmol, 1 eq), $Cu(NO_3)_2{\bullet}2.5H_2O$ (5.38 mg, 0.023 mmol, 2.1 eq), ethylene diamine (1.18 mL, 17.6 mmol, 1600 eq).

Synthesis of DC-104. CuTPz(OH)$_8$ (10 mg, 0.011 mmol, 1 eq), $Cu(NO_3)_2{\bullet}2.5H_2O$ (5.38 mg, 0.023 mmol, 2.1 eq), ethylene diamine (1.18 mL, 17.6 mmol, 1600 eq).

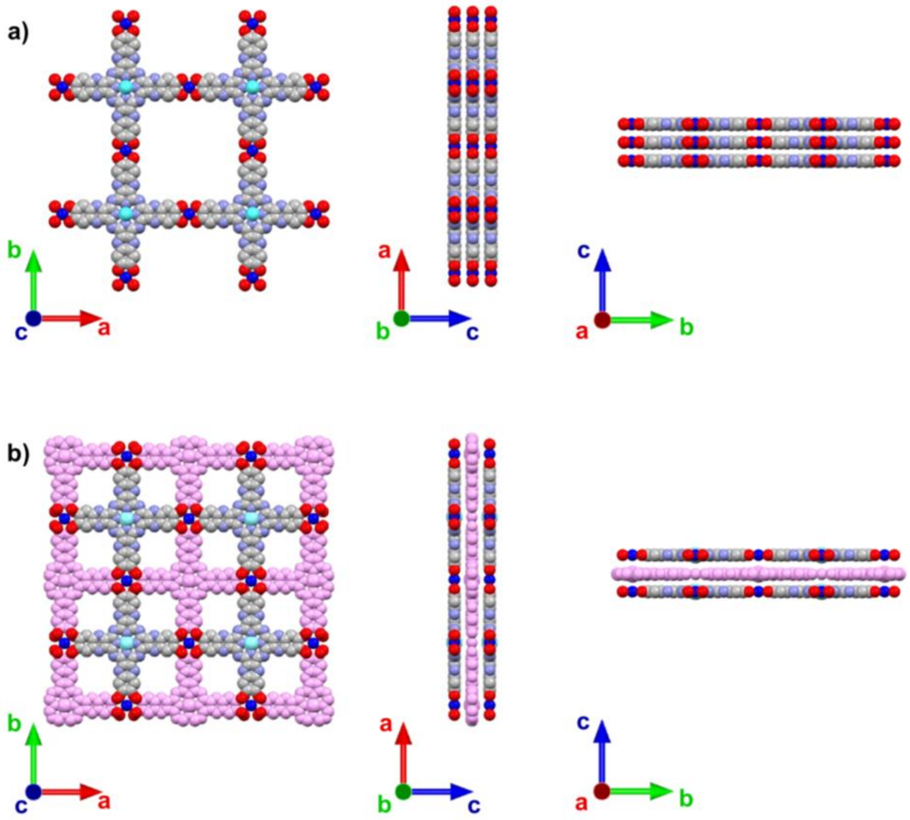

Figure S22. Front and side views of the a) eclipsed and b) staggered packing of MTPz-Cu-MOFs.

S3. Characterization of MTPz-Cu-MOFs

S3.1. Microscopy images of MTPz-Cu-MOF

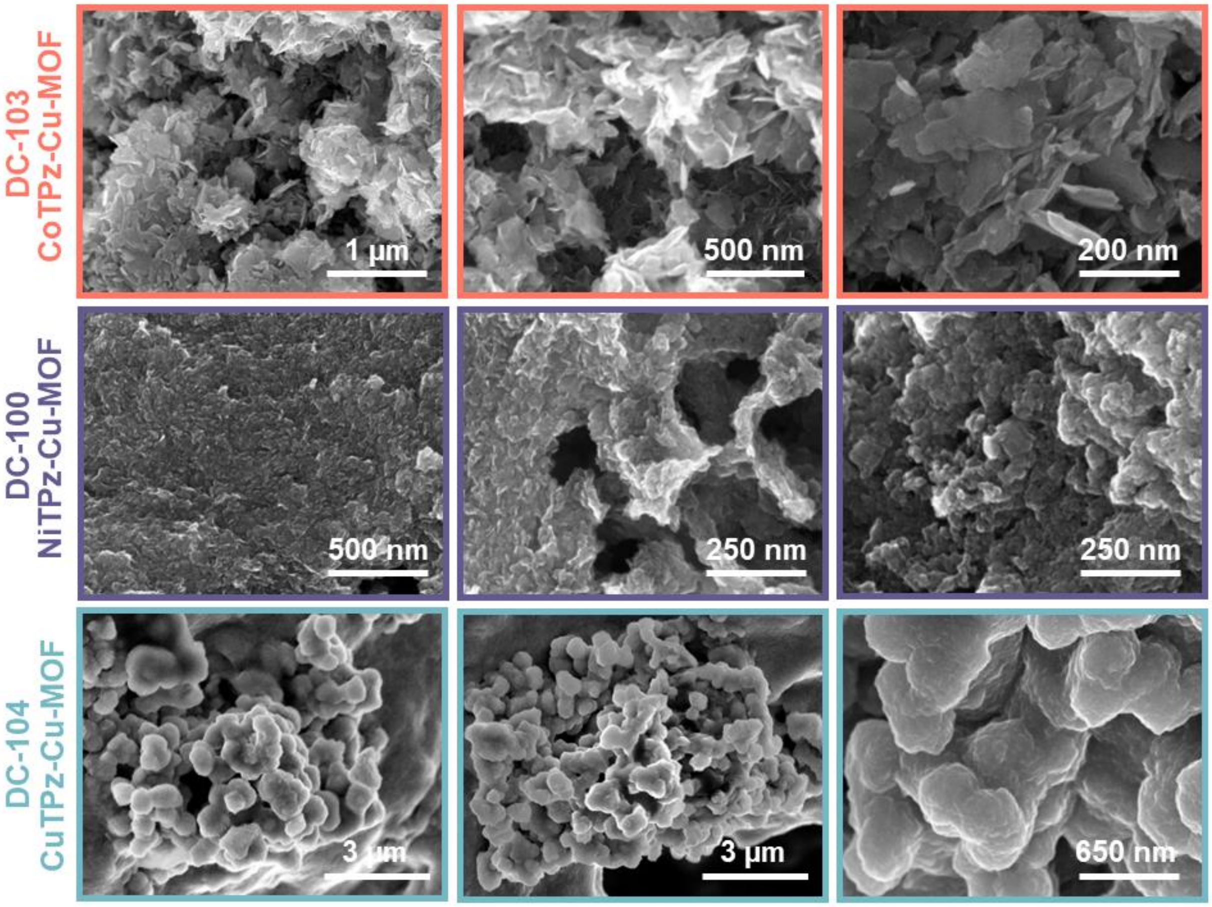

Figure S23. SEM images of the same batches of MTPz-Cu-MOFs taken at different magnifications.

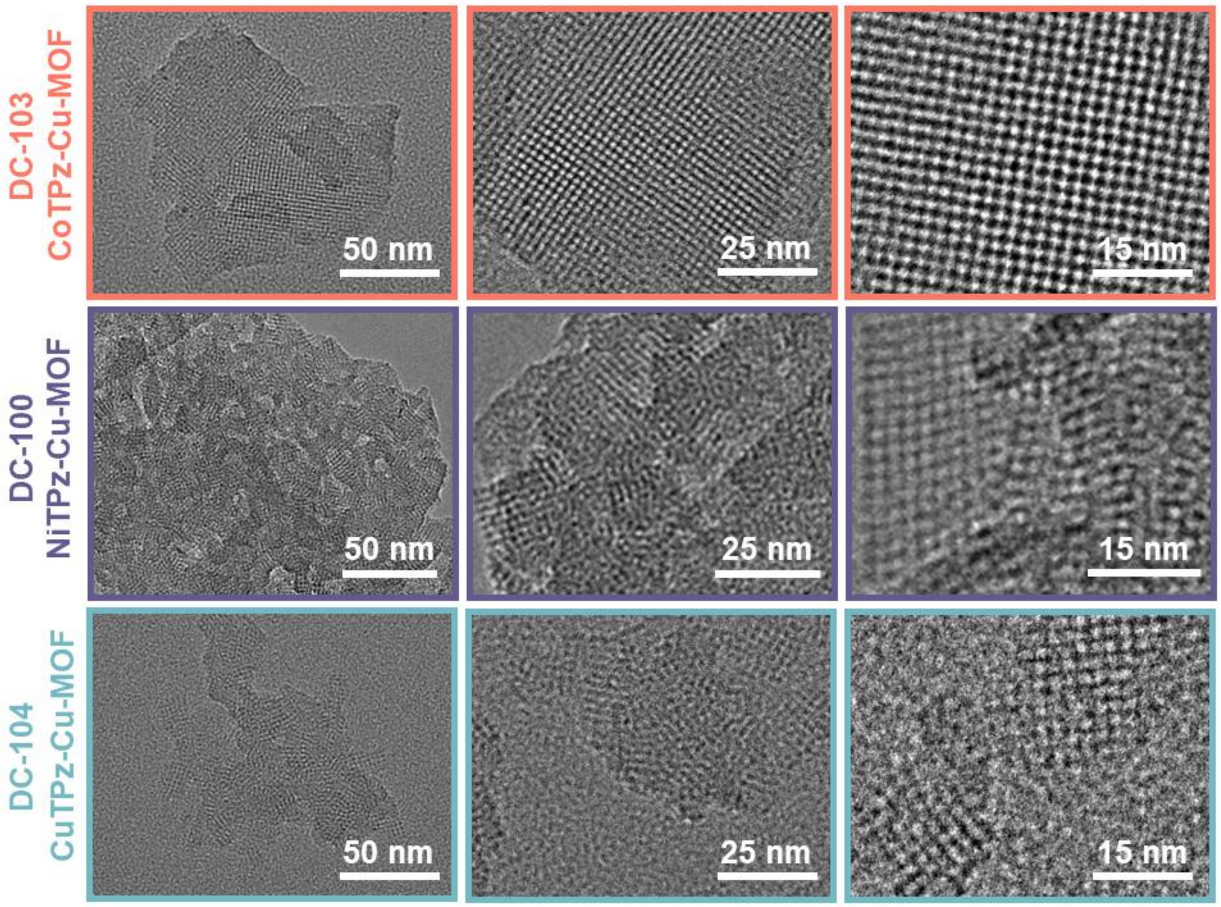

Figure S24. TEM images of the same batches of MTPz-Cu-MOFs taken at different magnifications.

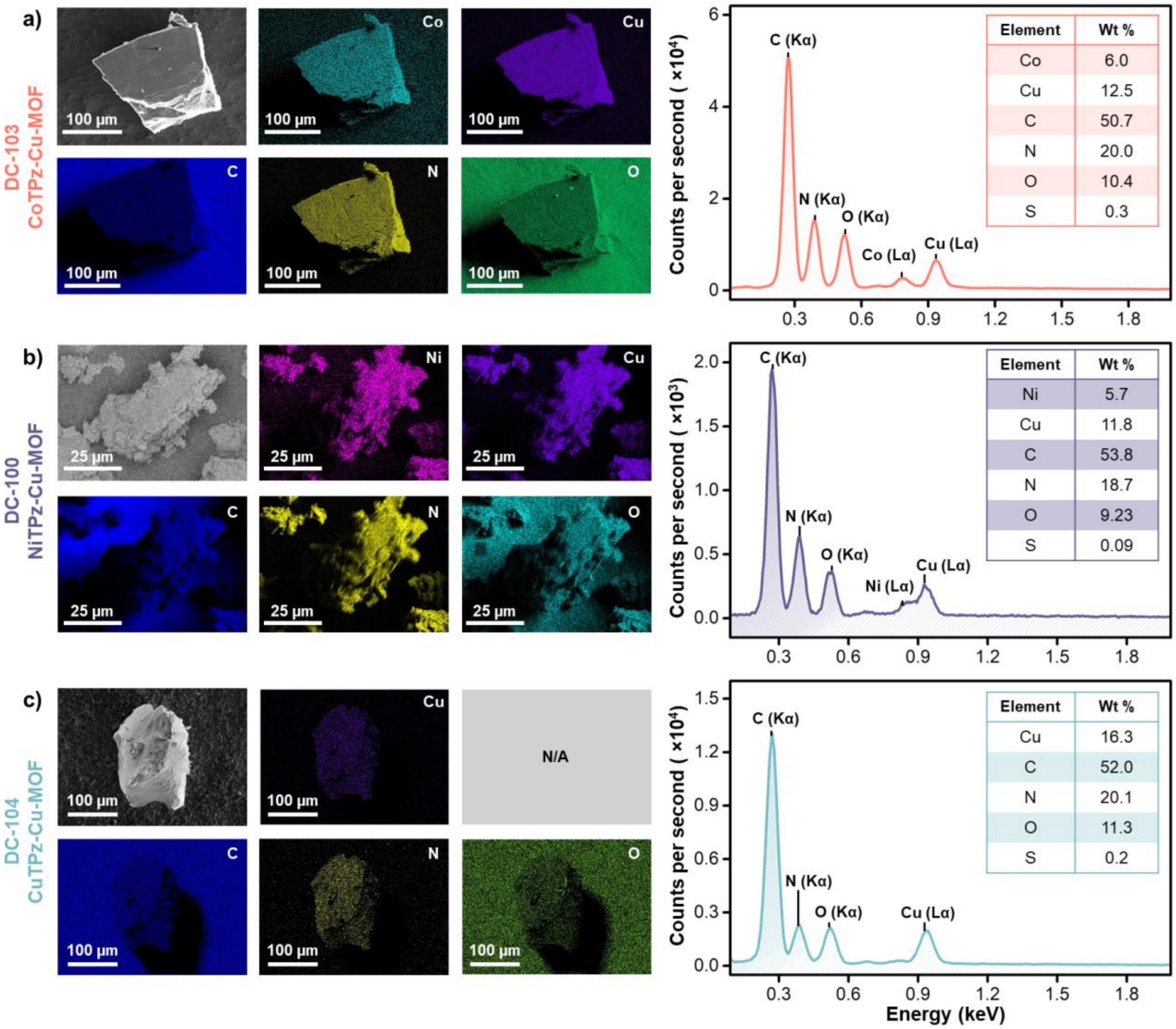

Figure S25. EDX mapping of a) DC-103, b) DC-100, and c) DC-104 .

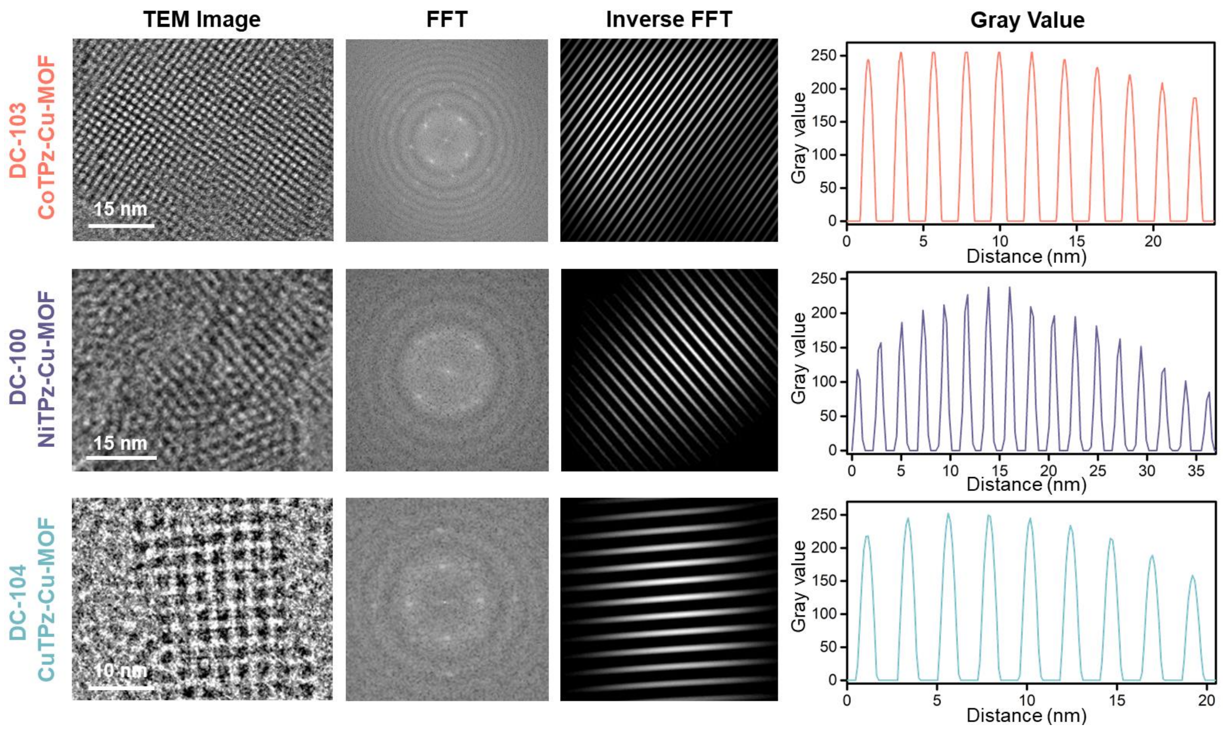

Figure S26. Determination of the interlayer spacing distances of the three MTPz-Cu-MOFs from their respective TEM images with FFT and inverse FFT analyses on ImageJ software. The gray value plots were determined from the inverse FFT for each MOF.

The interplanar spacing distance (d) between the (100) planes was calculated by dividing the total analyzed distance (D) for each divided by the number of observed peaks (# of peaks) within this distance, **Equation S1**.

Equation S1: $d = \frac{D}{\#\ of\ peaks}$

Table S1. Table showing the interplanar distances from experimental TEM images.

Planes	MOF	D (nm)	# of peaks	d (nm)
**(100)**	CoTPz-Cu-MOF	24.2	11	2.200
	NiTPz-Cu-MOF	37.7	17	2.218
	CuTPz-Cu-MOF	20.0	9	2.222

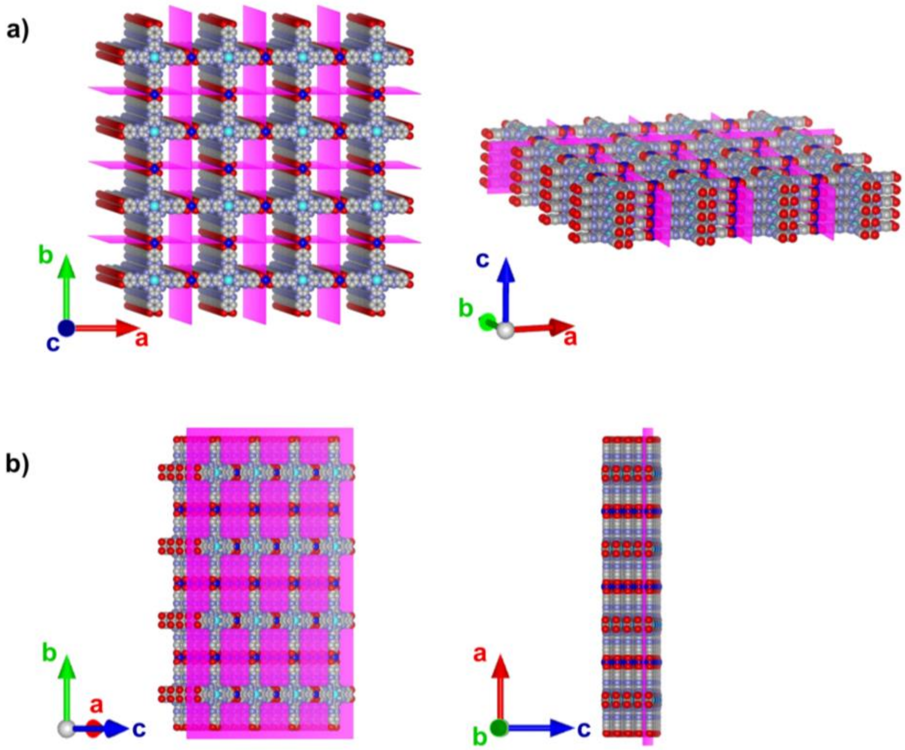

Figure S27. Illustration of the a) (100) and b) (001) families of planes, shown in pink, along the MTPz-Cu-MOF structure.

Equation S2 (Bragg's Law): $n\,\lambda = 2\,d\,\sin\theta$

where n is an integer (here considered as 1), λ is the wavelength of the incident rays (here equals to 1.54 Å or 0.154 nm from the Cu Kα source), d is the interlayer distance, and θ is the angle at which the crystals diffract to form the peaks.

Table S2. Table showing the interplanar distances from calculations according to Bragg's law.

Planes	MOF	2θ (°)	θ (°)	d (nm)
(100)	CoTPz-Cu-MOF	3.92	1.96	2.25
	NiTPz-Cu-MOF	3.94	1.97	2.24
	CuTPz-Cu-MOF	3.98	1.99	2.22
(001)	CoTPz-Cu-MOF	27.64	13.82	0.322
	NiTPz-Cu-MOF	27.52	13.76	0.324
	CuTPz-Cu-MOF	27.40	13.70	0.325

S3.2. Elemental Analysis of MTPz-Cu-MOFs

To quantify the metal content of the MOFs, we performed inductively-coupled plasma mass spectrometry (ICP-MS) on a diluted sample of MOF, digested in a 9:1 solution of concentrated sulfuric acid and hydrogen peroxide (6%).

Table S3. Table summarizing the CHNS combustion and the ICP-MS analysis results of the three MOFs. (theo % = theoretical percentage based on the simulated unit cell, exp % = average of two experimental percentage values)

		**DC-103**		**DC-100**		**DC-104**	
		theo (%)	exp (%)	theo (%)	exp (%)	theo (%)	exp (%)
**CHNS combustion**	C	46.80	41.13	46.81	44.53	46.59	41.36
	H	0.79	3.56	0.79	3.73	0.78	3.30
	N	21.83	18.49	21.83	21.83	21.73	19.74
	S	0.00	0.35	0.00	1.07	0.00	0.00
**ICP-MS**	M	(M = Co) 5.74	5.44	(M = Ni) 5.72	4.44	-	-
	Cu	12.38	13.79	12.38	9.98	18.49	17.1

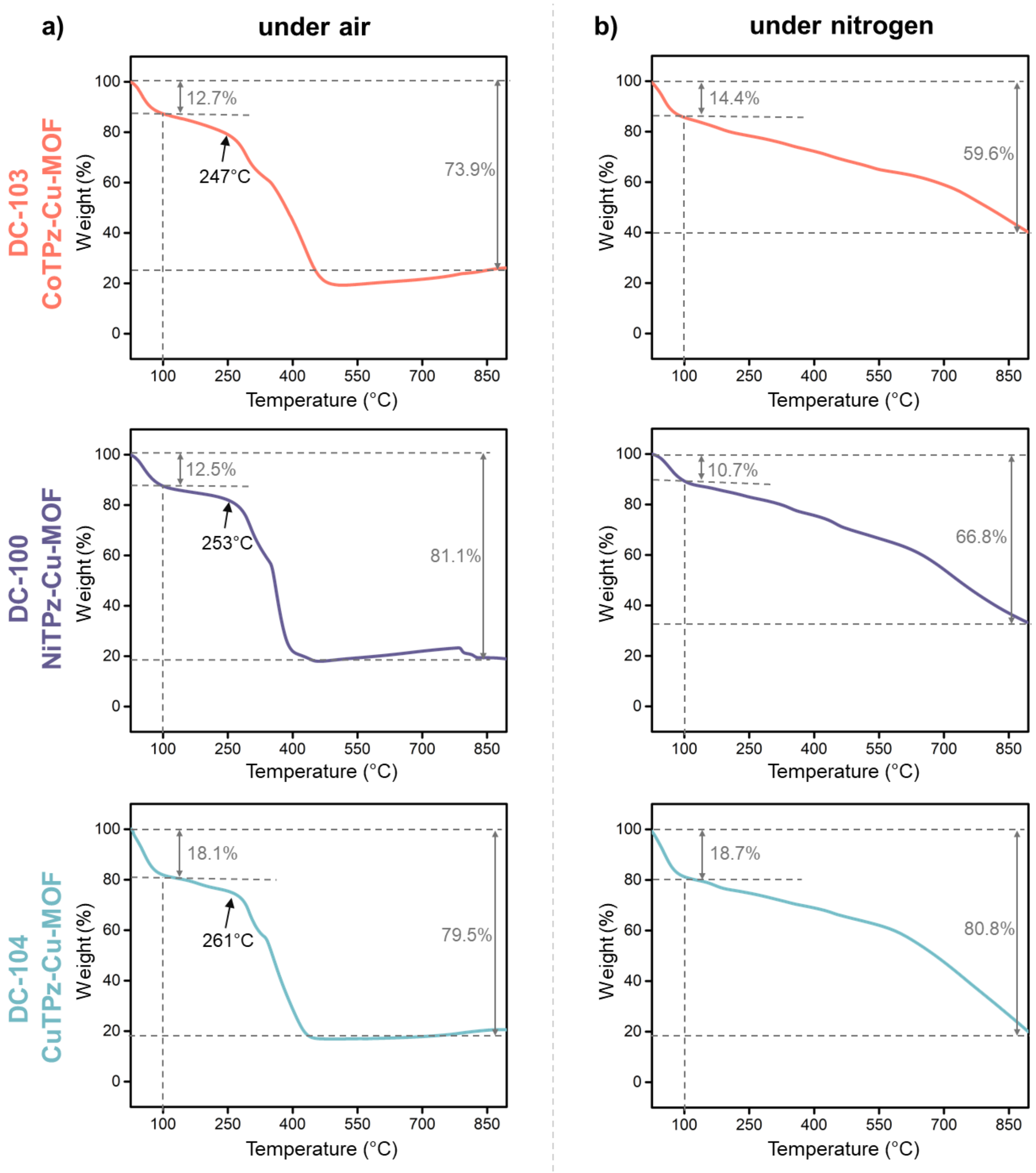

Figure S28. TGA traces of the MTPz-Cu-MOF analogues a) in air and b) in nitrogen. Plots were collected from 25 to 900 °C at a ramp rate of 5 °C/min.

Table S4. Table summarizing the deduced molecular formulas of the three MOFs. (theo % = theoretical percentage, exp % = experimental percentage values)

	DC-103		DC-100		DC-104	
	$CoTPzCu_2(H_2O)_3(DMSO)_{0.1}$		$NiTPzCu_2(EDA)_2(H_2O)_3(DMSO)_{0.5}$		$CuTPzCu_2(H_2O)_4$	
	theo (%)	exp (%)	theo (%)	exp (%)	theo (%)	exp (%)
**C**	44.34	41.13	43.60	44.53	43.53	41.36
**H**	1.34	3.56	2.68	3.73	1.45	3.30
**N**	20.59	18.49	22.60	21.83	20.31	19.74
**S**	0.29	0.34	1.30	1.07	0.00	0.00
**M**	(M = Co) 5.41	5.44	(M = Ni) 4.73	4.44	-	-
**Cu**	11.68	13.79	10.25	9.98	17.29	17.1
**O**	16.33	calc. 17.25	14.84	calc. 14.42	17.41	calc. 17.51

S3.3. ATR-FTIR spectra

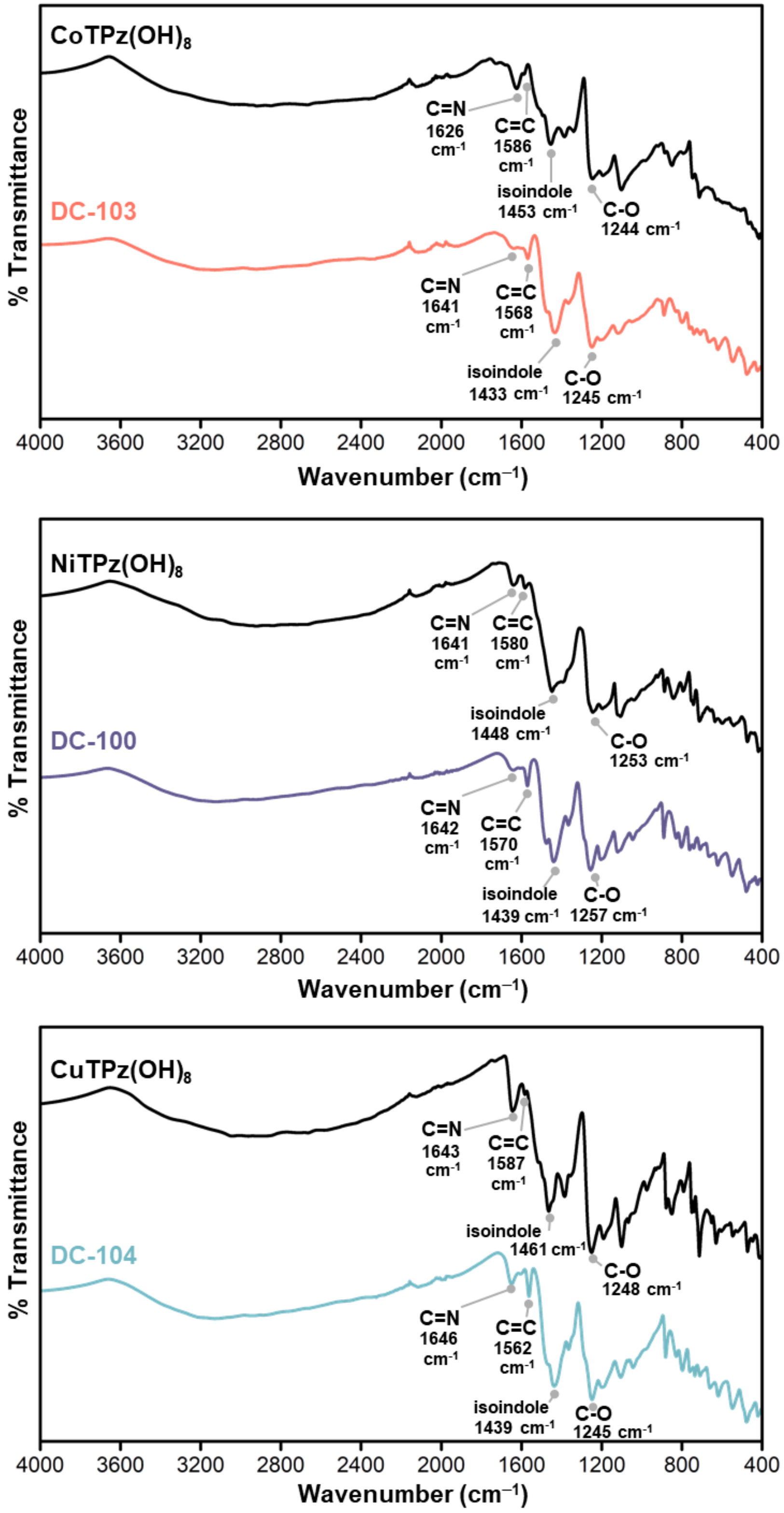

Figure S29. ATR-FTIR spectra of the $MTPz(OH)_8$ monomers and the MTPz-Cu-MOF analogues.

S3.4. XPS spectra

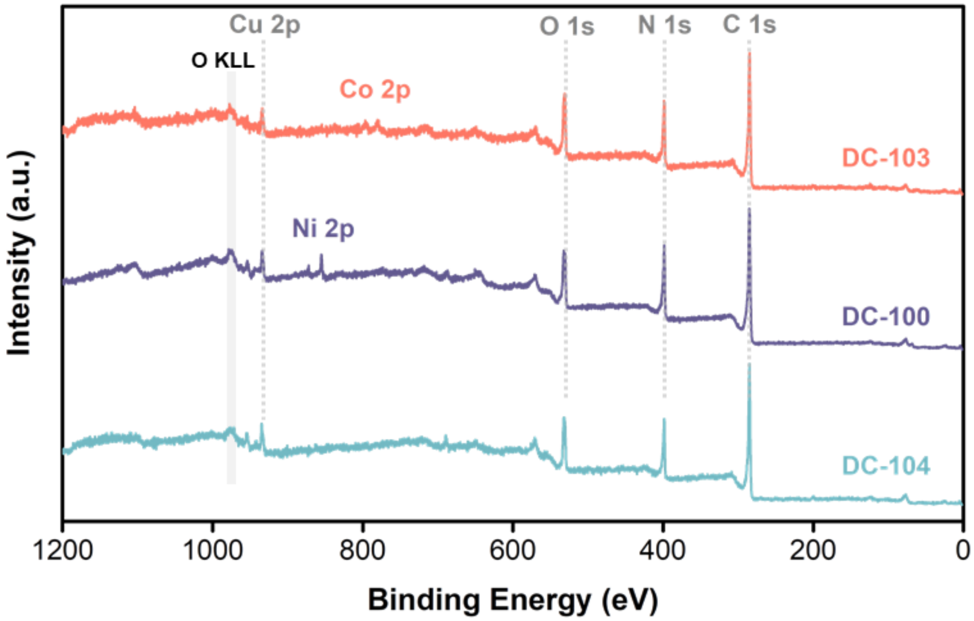

Figure S30. XPS survey spectra of the three MOF analogues.

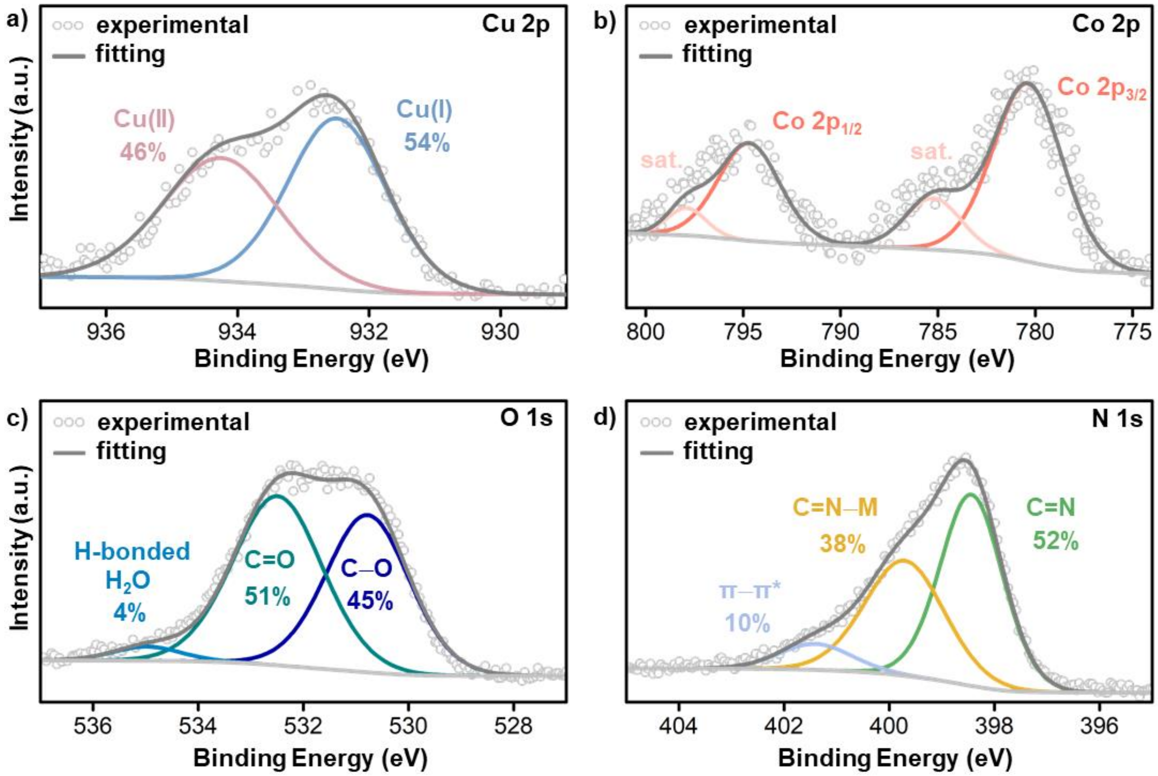

Figure S31. High-resolution XPS spectra of CoTPz-Cu-MOF at the binding energies of a) Cu 2p, b) Co 2p, c) O 1s, and d) N 1s.

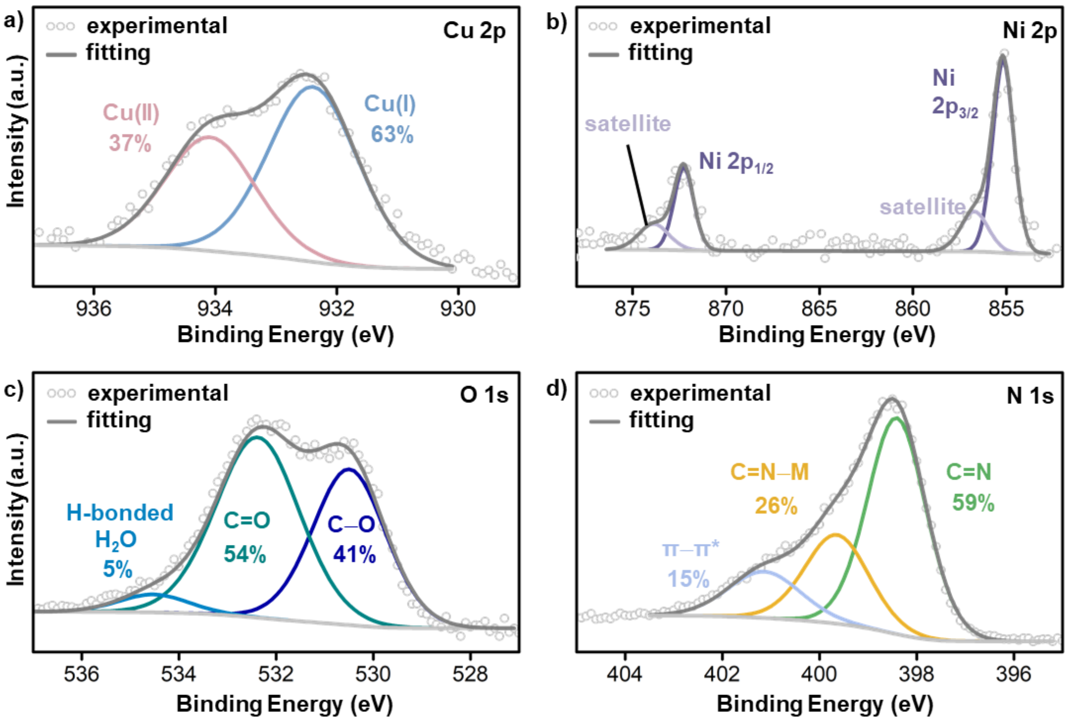

Figure S32. High-resolution XPS spectra of NiTPz-Cu-MOF at the binding energies of a) Cu 2p, b) Ni 2p, c) O 1s, and d) N 1s.

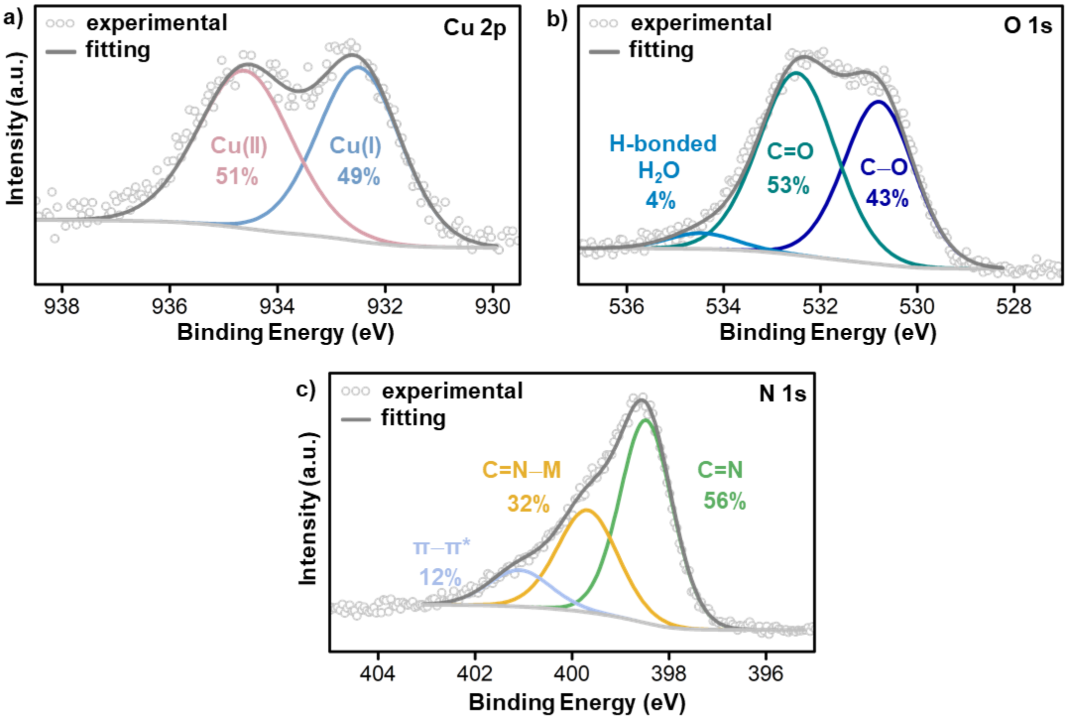

Figure S33. High-resolution XPS spectra of CuTPz-Cu-MOF at the binding energies of a) Cu 2p, b) O 1s, and c) N 1s.

S3.5. EPR plots

2 mg of each sample were placed in quartz EPR tubes and analyzed at room temperature. $CoTPz(OH)_8$ shows a weak signal with g = 2.257, which we attributed to the low-spin d^7 configuration of Co(II) constituting the core of the TPz ligand (**Figure S33a**). Accordingly, we assigned the EPR signal at g = 2.068 in DC-103 to that of paramagnetic Co^{2+} or Cu^{2+} of the MOF, consistent with previous reports.[4] The g-value of 1.961 for $NiTPz(OH)_8$ corresponds to partially oxidized monomer species or adsorbed oxygen molecules, whereas that of DC-100 at 2.058 is a metal-centered EPR signal resulting from the paramagnetic Cu(II) ions of the MOF (**Figure S33b**).[5] $CuTPz(OH)_8$ and DC-104 in **Figure S17c**, however, showed a slight deviation in the g-value from 2.019 to 2.058, both of which result from paramagnetic d^9 Cu(II) ions.[6]

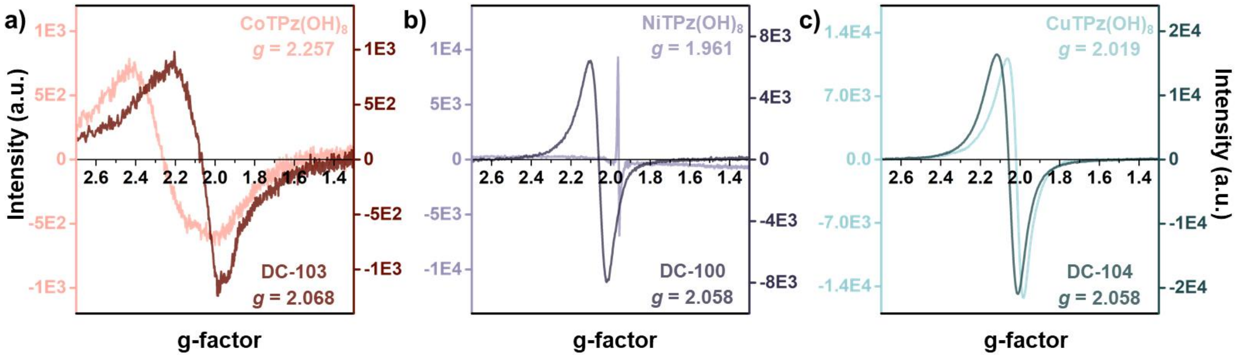

Figure S34. EPR plots taken at room temperature of a) $CoTPz(OH)_8$ monomer and DC-103, b) $NiTPz(OH)_8$ monomer and DC-100, c) $CuTPz(OH)_8$ monomer and DC-104.

S3.6. Conductivity measurements

Conductivity measurements were collected on separate pressed pellets of each MOF. Pellets (diameter = 6 mm, thickness = 75 μm) were prepared upon pressing ~ 20 mg of each MOF with a pellet presser at 1000 psi for 10 minutes in a 6 mm disc-shaped die set mold.

The electrical conductivities were determined by a four-point linear probe, as illustrated in **Figure S35**, according to **Equation S3**.[7] The obtained average results of 9 to 11 measurements for each MOF are summarized in **Table S5**.

Equation S3: $$\sigma = \frac{I}{V} \times \frac{1}{2\pi sF}$$

where σ is the electrical conductivity (in S cm^{-1}), I is the current (in A), V is the voltage (in V), s is the spacing between the probes (here 1.25 mm), and F is a unitless correction factor for the diameter and thickness of the pellet.

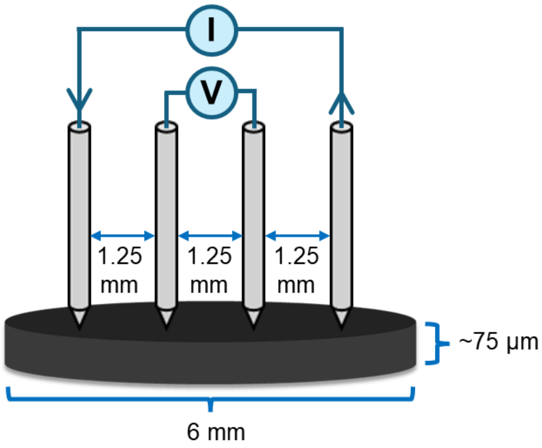

Figure S35. Schematic representation of the four-point probe conductivity measurements.

Proton conductivity measurements were collected, using a two-point probe, in a saturated (98% RH) humidity home-made chamber, at different temperatures ranging from 303 to 333 K. **Figure S36** shows the obtained Nyquist plots as well as the temperature-dependence change in proton conductivity for the three MOF analogs and their deduced activation energy (E_A). The resulting proton conductivity at the studied temperature ranges are summarized in **Table S5**.

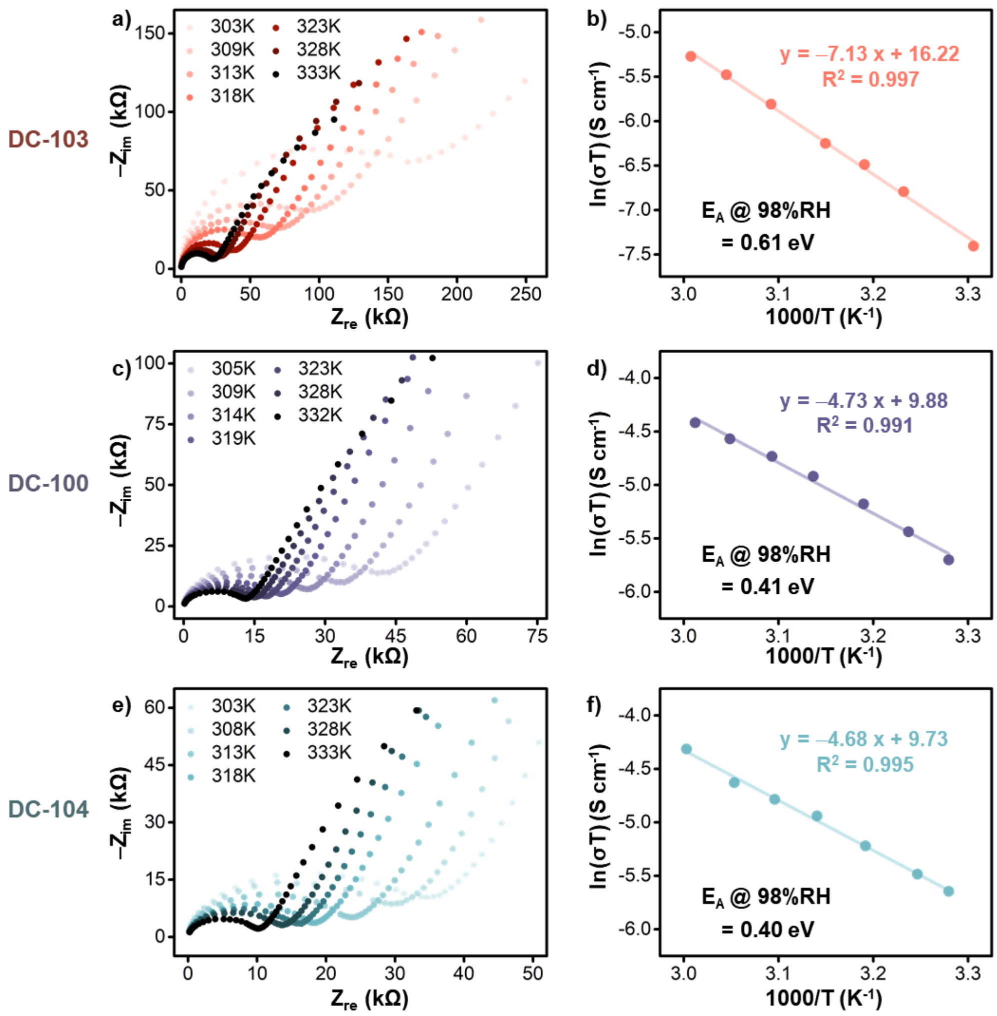

Figure S36. Nyquist plot at temperatures ranging between 303 and 333K and temperature-dependent conductivity calculations and deduced activation energy values at saturated (98% RH) humidity of a,b) DC-103, c,d) DC-100, and e,f) DC-104. These measurements allowed for the determination of the proton conductivity values shown in **Table S5**.

Table S5. Table summarizing the electrical conductivities (at ambient conditions) and proton conductivities (at 303–333K and 98%RH) of MTPz-Cu-MOF analogues.

	DC-103	DC-100	DC-104
**Electrical conductivity ($S\ cm^{-1}$)**	$7.59 \pm 2.62 \times 10^{-4}$	$2.59 \pm 1.27 \times 10^{-6}$	$9.66 \pm 1.50 \times 10^{-7}$
**Proton conductivity ($S\ cm^{-1}$)**	$0.2 – 1.5 \times 10^{-5}$	$1.1 – 3.6 \times 10^{-5}$	$1.2 – 4.0 \times 10^{-5}$

S3.7. BET isotherms and surface areas

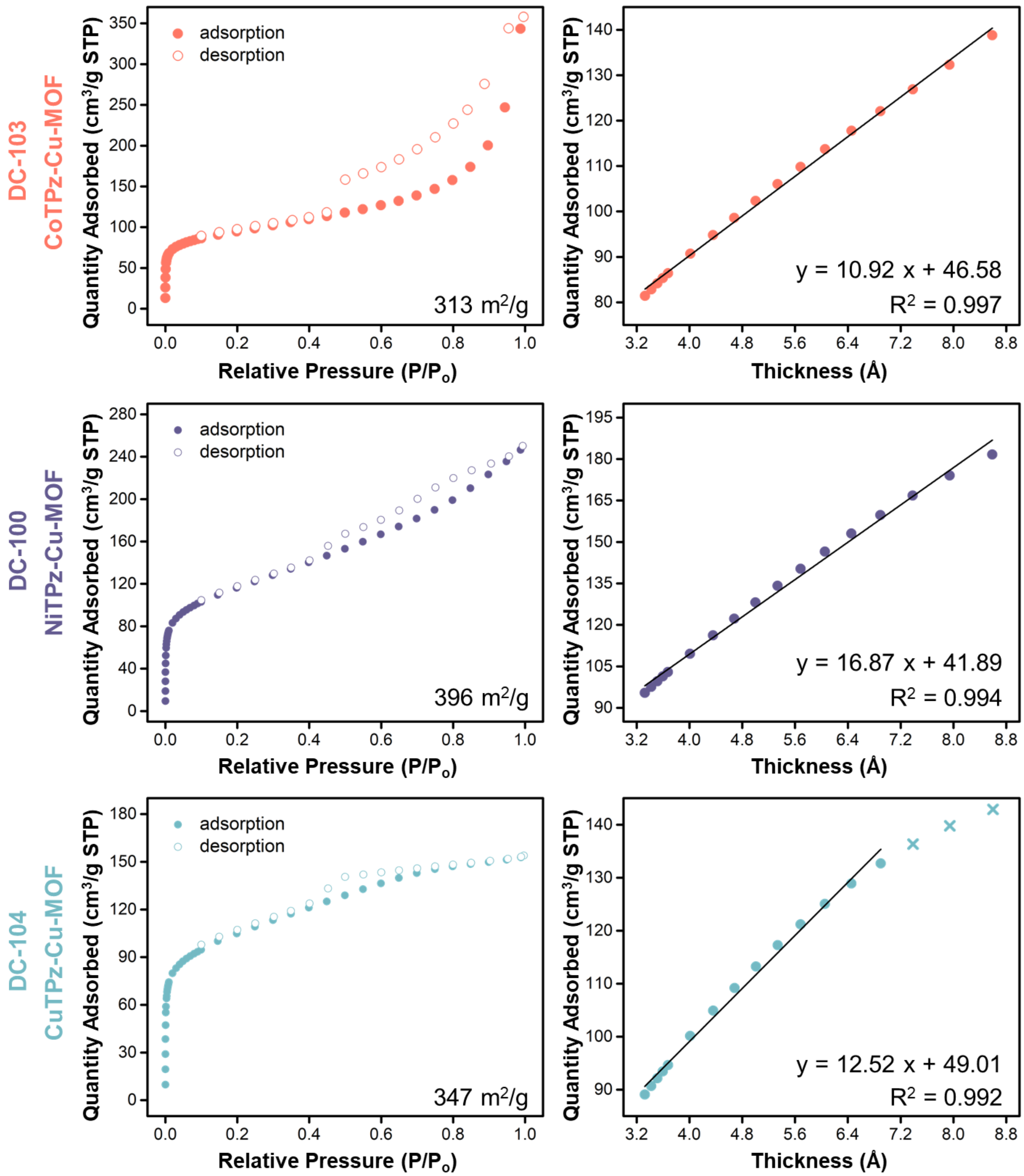

Figure S37. BET N_2 adsorption and desorption isotherms for the three MTPz-Cu-MOF analogues (left) and their respective t-Plots (right). Points designated by × were not fitted into the equation.

Table S6. Table summarizing the BET surface areas and the t-plot micropore areas of MTPz-Cu-MOF analogues.

	DC-103	DC-100	DC-104
**BET surface area (m^2 g^{-1})**	313.1	395.7	347.2
**Micropore area (m^2 g^{-1})**	121.7	94.8	120.2
**External surface area (m^2 g^{-1})**	191.4	300.9	227.01

S3.8. Optical band gaps of pristine MOFs

UV-Vis-NIR absorption spectra of MTPz-MOF thin films were collected upon dropcasting MOF suspensions on quartz glass slides. Briefly, 250 µL of MOF suspensions (with concentrations of 2.5 mg mL^{-1} in H_2O) were dropcasted on the quartz slides and dried in an oven at 50 °C for 2 hours. A blank quartz baseline and a zero background correction, were collected prior to the sample measurement. Absorption spectra were collected between 400 and 2000 nm at a rate of 400 nm min^{-1} under ambient conditions, with representative shown in **Figure S38**. We determined the optical band gaps using the Tauc plots, which showed values in the range of 1.18–1.33 eV, consistent with band gap magnitudes of semiconducting materials.[8]

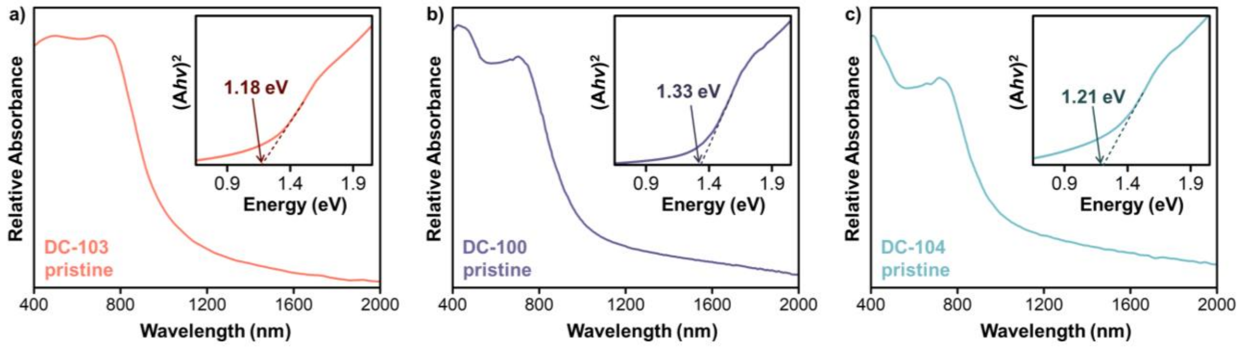

Figure S38. Representative UV-Vis-NIR absorption spectra and inset graphs of the Tauc plots with the determined optical band gaps of pristine a) DC-103, b) DC-100, and c) DC-104.

S4. Computational studies

All calculations were performed using density functional theory as implemented in the Vienna Ab initio Simulation Package (VASP 6.5.1) using a plane wave basis set and projector augmented-wave pseudopotentials.[9-11] The primitive cells of the periodic framework materials were structurally optimized on a 1 × 1 × 8 Gamma-centered *k*-mesh with a cutoff energy of 520 eV using the PBEsol generalized gradient approximation functional.[12] Interlayer van der Waals dispersion interactions were modeled using the DFT+D3 formalism.[13] The transition metal *d* orbitals were localized with the DFT+U formalism using effective Hubbard U values (Co: 2.0 eV, Ni: 6.4 eV, Cu: 4.0 eV) taken from fits to experimental oxidation energies for transition metal oxides.[14, 15] The ionic and electronic convergence criteria were 10 meV/Å and 10^{-6} eV, respectively. After structural relaxation, the electronic band structures were computed using the HSEsol functional along a truncated k-path for the *P*4/*mmm* space group to sample in-plane and out-of-plane interactions with an electronic convergence criterion of 10^{-6} eV.

In the charge-neutral structures containing TPz units, the electronic band structures obtained for DC-103, DC-100, and DC-104 showed out-of-plane conductivity with band gaps in the covalent direction of approximately 0.5 eV, well below the experimentally-observed optical gaps. However, reducing each of the pyrazine nitrogen atoms in the structure to yield a charge-neutral octahydro-TPz moiety widened the electronic gaps in the covalent direction to 1.24 eV, 1.53 eV, and 1.51 eV for DC-103, DC-100, and DC-104, respectively **(Figure S39)**. These calculated electronic gaps are more consistent with the experimental optical gaps.

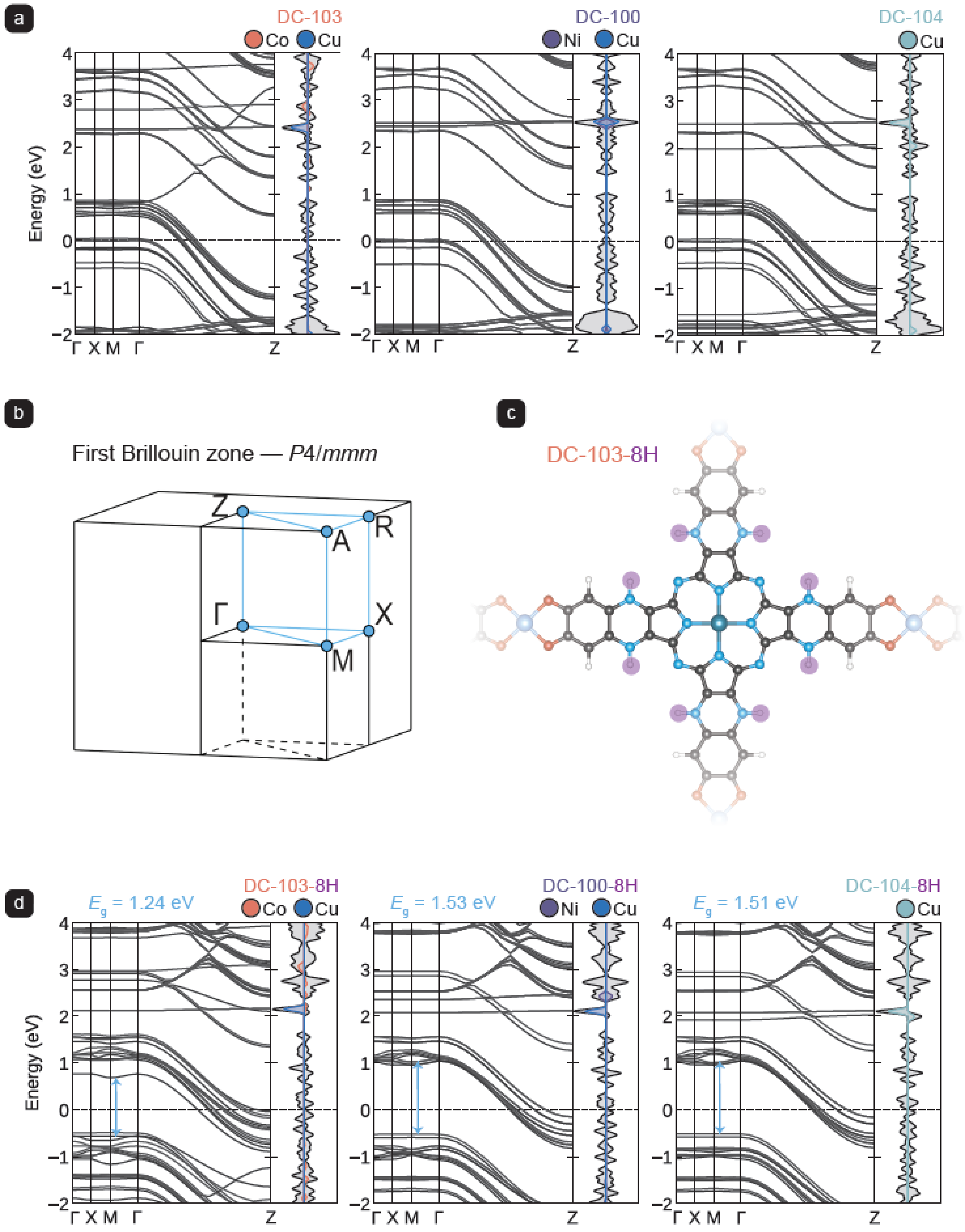

Figure S39. a) Hybrid band structure diagrams and projected density-of-states for the periodic framework materials in the charge-neutral TPz configuration, showing electronic gaps in the covalent direction on the order of ~0.5 eV. b) First Brillouin zone of the periodic framework materials. The Γ-X-M-Γ path samples the covalent plane of the material, and the Γ-Z path samples the out-of-plane stacking direction. c) A representative example of the reduced framework, resulting in a charge-neutral octahydro-TPz moiety, with the additional H atoms highlighted in purple. d) Hybrid band structure diagrams and projected density-of-states for the periodic framework materials in the charge-neutral octahydro-TPz configuration, showing electronic gaps in the covalent direction that are in close agreement with experimental optical gaps.

S5. Sensing experiments of MTPz-Cu-MOFs

S5.1. Preparation of sensing devices

Suspensions of each of the MOFs were prepared by sonicating 1 mg of the MOF in 1 mL of Milli-Q water for around 1.5 to 2 hours. PXRD spectra of the sonicated MOFs were recorded to confirm the retainment on their crystallinity, as shown in **Figure S40**.

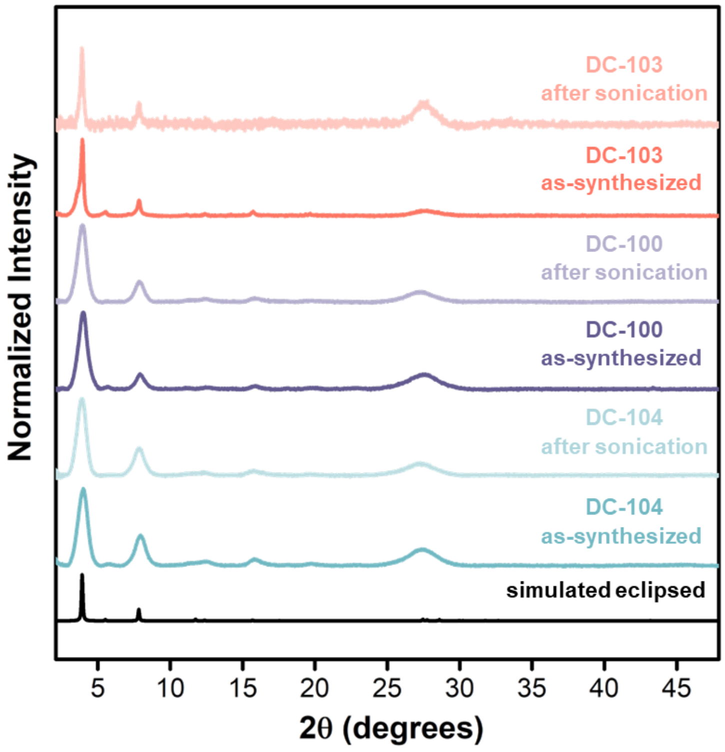

Figure S40. PXRD traces of the MOF analogues as-synthesized and after sonication for around 2 hours.

Of each suspension, 25 μL were drop-casted onto 10-μm gold interdigitated electrodes for DC-103 and on 5-μm gold interdigitated electrodes for DC-100 and DC-104. The electrodes were allowed to dry in the oven at 80-85 °C for 30 minutes to get resistance values in the range of 40 kΩ to 0.2 MΩ. Images of the electrodes are shown in **Figure S41**.

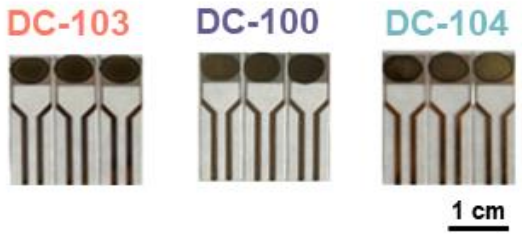

Figure S41. Representative photographs of the electrodes after dropcasting and drying the MOF suspensions overnight.

S5.2. Sensing experiment setup

A home-built setup, illustrated in **Figure S42**, is used for the sensing experiments. Analyte gases were purchased in 10,000 ppm concentrations (1% analyte gas in dry N_2) and diluted in dry nitrogen gas to allow the elution of the desired concentration of gas analyte. Briefly, a low-flow mass flow controller connected to the analyte gas tank would deliver the desired amount needed, say 2 mL/min, and the latter will then get premixed with nitrogen gas delivered through a high-flow mass flow controller, say at 0.5 L/min, before reaching the Teflon chamber, where our electrodes are placed, with a resulting diluted concentration of 40 ppm. The Teflon gas chamber creates a good seal around the electrodes with an inlet of the diluted gas and an outlet when ready for measurement.

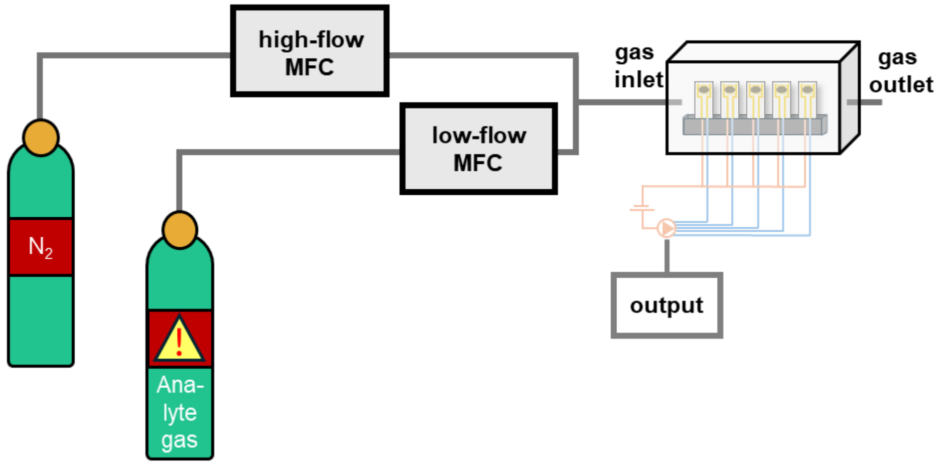

Figure S42. A schematic illustration of the chemiresistive sensing setup.

The electrodes are first equilibrated with a stream of nitrogen gas for around 15 minutes to ensure a straight baseline prior to recording. Each recorded measurement includes 10 minutes of nitrogen gas flow, followed by 10 minutes exposure to the analyte gas at the desired concentration, and a 30-minute recovery period where the analyte mass flow controller is switched off, and the electrodes are only subjected to nitrogen gas. All measurements were performed at a constant driving voltage of 0.1 V. The output obtained from the software is in the form of changes in current with time.

The change in current of different electrodes was normalized according to **Equation S4**, where -$\Delta G/G_0$ (in %) is the normalized response, I (in μA) represents the current at a certain time, and I_0 (in μA) is the initial current at 10 min directly before gas exposure.

Equation S4: $$-\Delta G/G_0 = -\frac{I - I_0}{I_0} \times 100$$

S5.3. H_2S gas sensing

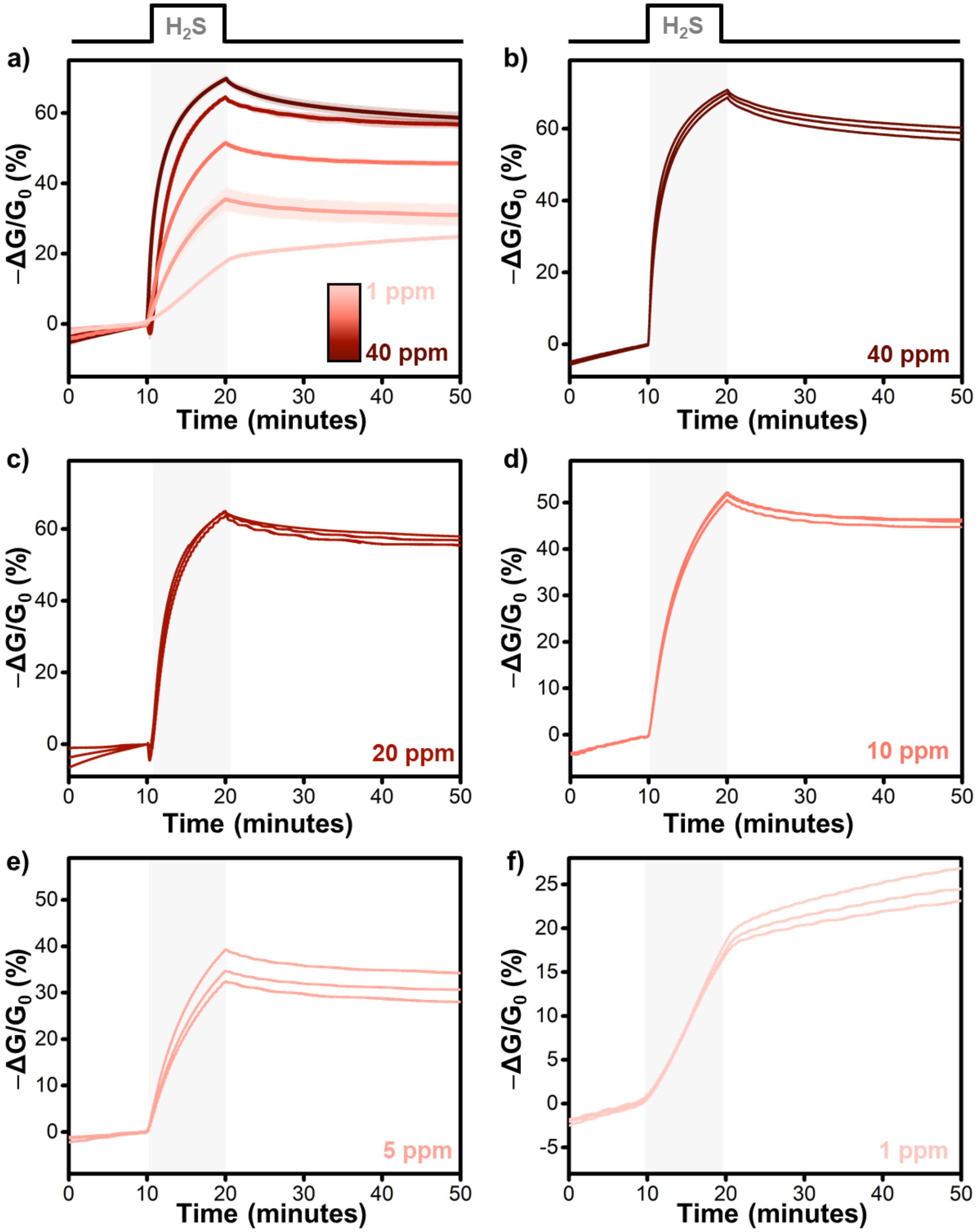

Figure S43. a) Averaged sensing responses of DC-103 towards H_2S at different concentrations. The orange shaded area represents the standard deviation of sensing responses from 3 devices. Sensing responses of at least 3 devices of DC-103 towards b) 40 ppm, c) 20 ppm, d) 10 ppm, e) 5 ppm, and f) 1 ppm of H_2S. The grey shaded area represents the time of exposure of the devices to H_2S.

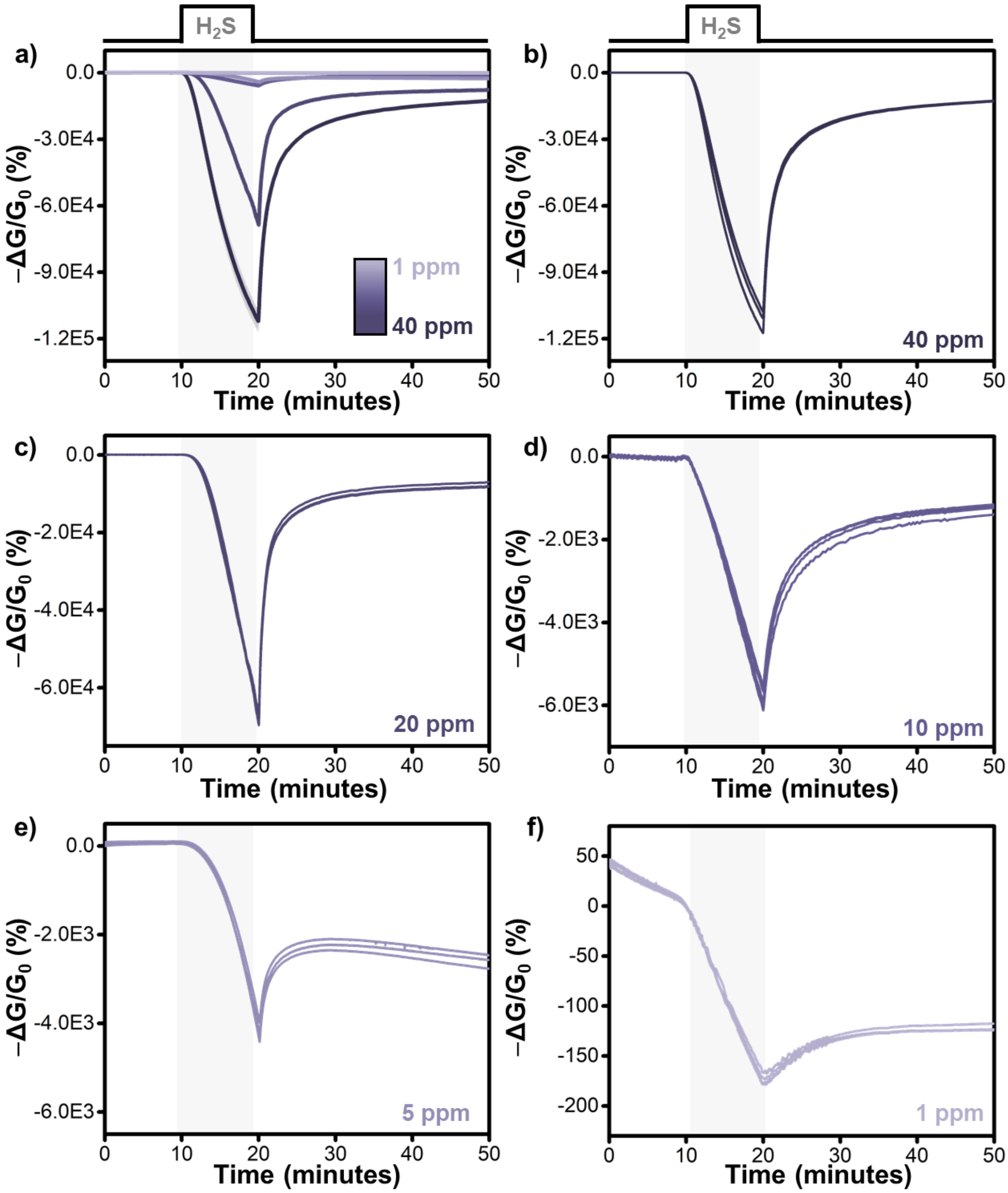

Figure S44. a) Averaged sensing responses of DC-100 towards H_2S at different concentrations. The purple shaded area represents the standard deviation of sensing responses from 3 devices. Sensing responses of at least 3 devices of DC-100 towards b) 40 ppm, c) 20 ppm, d) 10 ppm, e) 5 ppm, and f) 1 ppm of H_2S. The grey shaded area represents the time of exposure of the devices to H_2S.

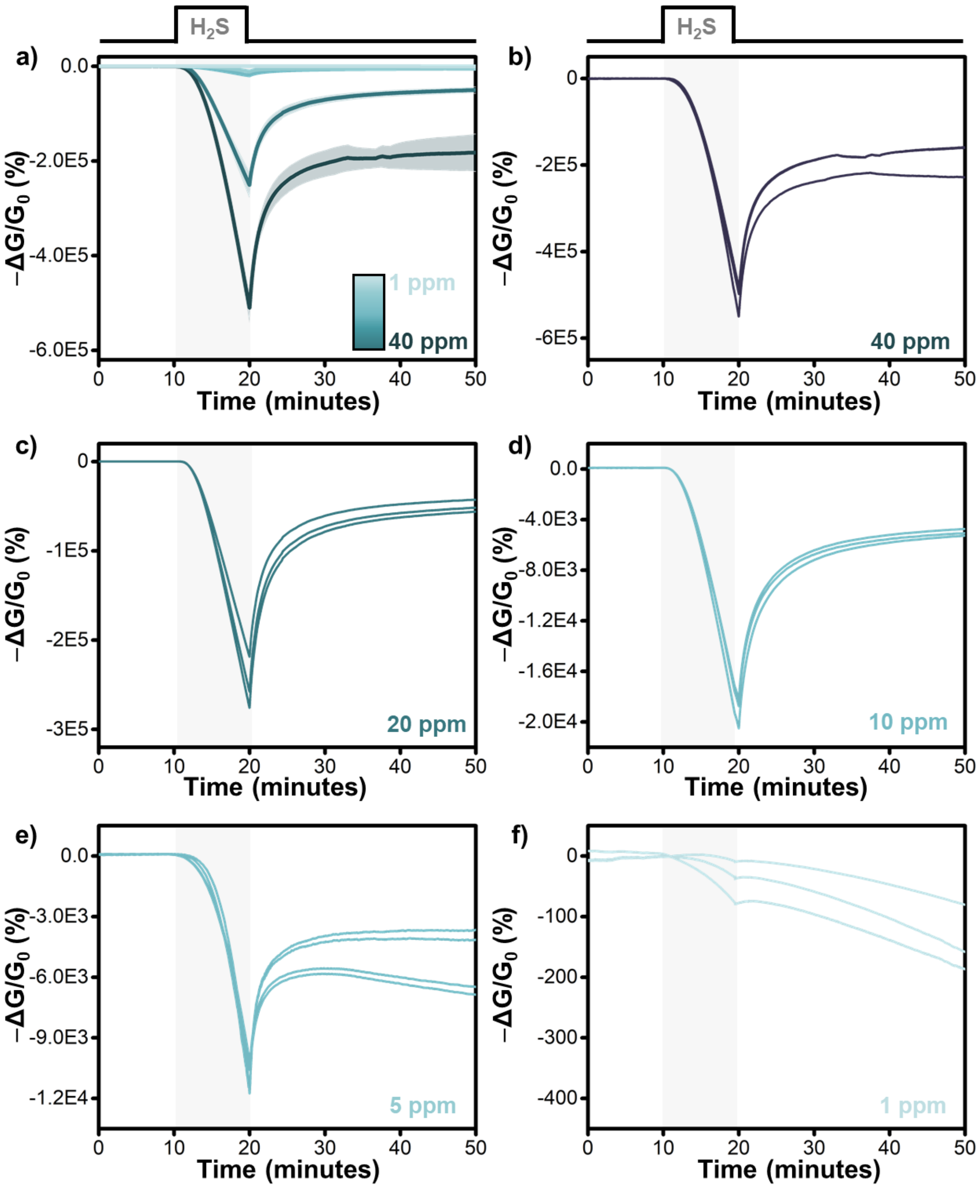

Figure S45. a) Averaged sensing responses of DC-104 towards H_2S at different concentrations. The teal shaded area represents the standard deviation of sensing responses from 3 devices. Sensing responses of at least 3 devices of DC-104 towards b) 40 ppm, c) 20 ppm, d) 10 ppm, e) 5 ppm, and f) 1 ppm of H_2S. The grey shaded area represents the time of exposure of the devices to H_2S.

S5.4. NH_3 gas sensing

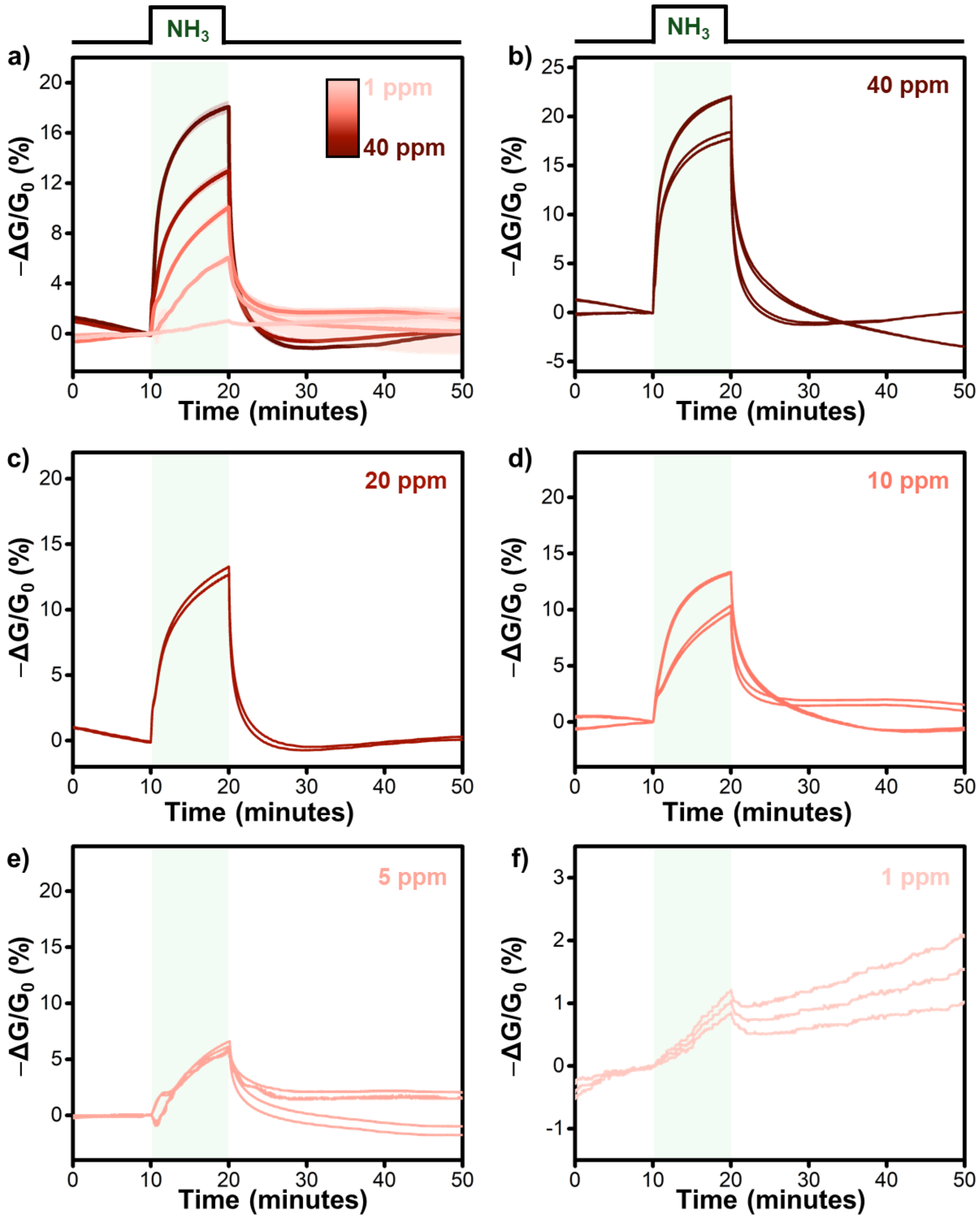

Figure S46. a) Averaged sensing responses of DC-103 towards NH_3 at different concentrations. The orange shaded area represents the standard deviation of sensing responses from 3 devices. Sensing responses of at least 3 devices of DC-103 towards b) 40 ppm, c) 20 ppm, d) 10 ppm, e) 5 ppm, and f) 1 ppm of NH_3. The green shaded area represents the time of exposure of the devices to NH_3.

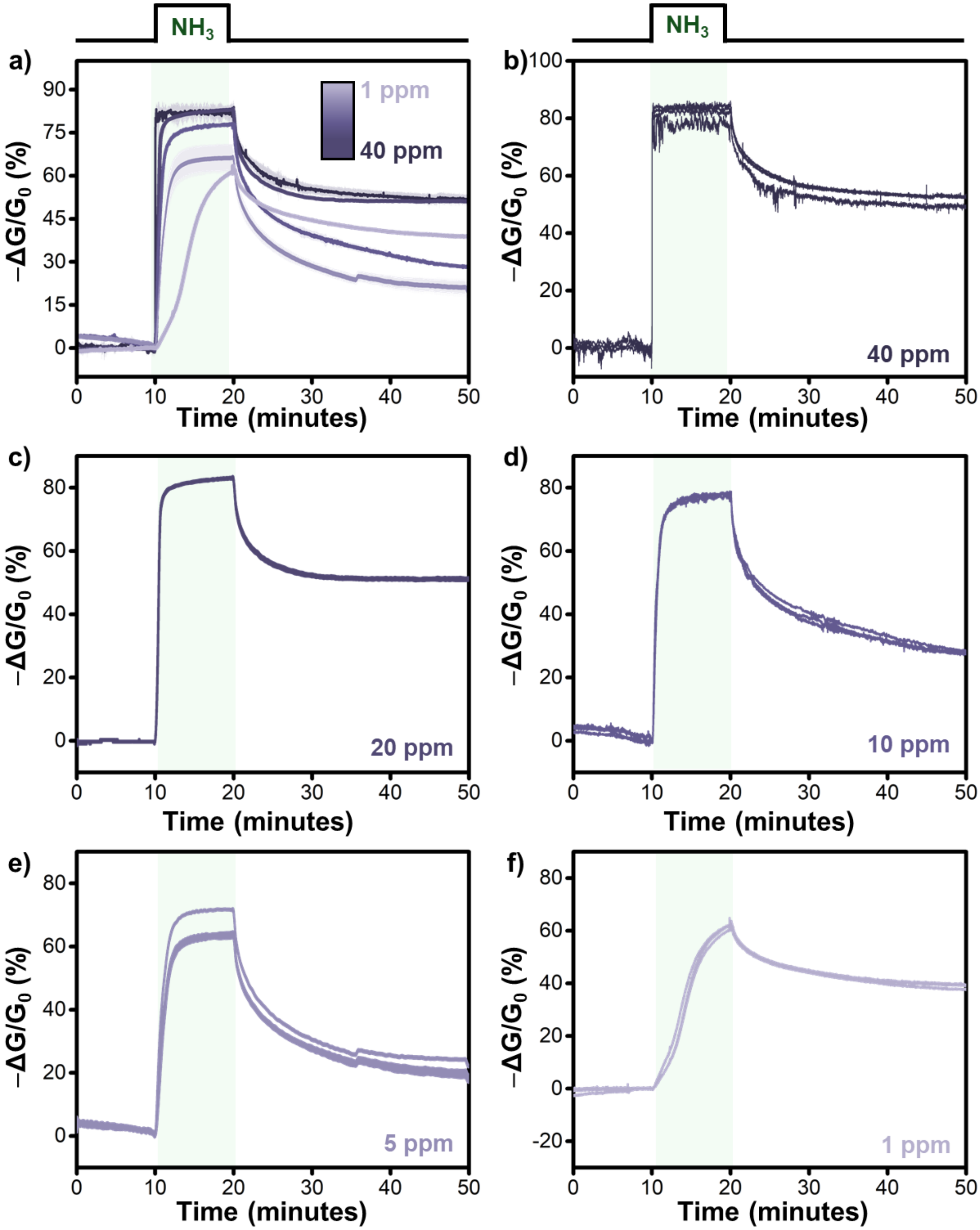

Figure S47. a) Averaged sensing responses of DC-100 towards NH_3 at different concentrations. The purple shaded area represents the standard deviation of sensing responses from 3 devices. Sensing responses of at least 3 devices of DC-100 towards b) 40 ppm, c) 20 ppm, d) 10 ppm, e) 5 ppm, and f) 1 ppm of NH_3. The green shaded area represents the time of exposure of the devices to NH_3.

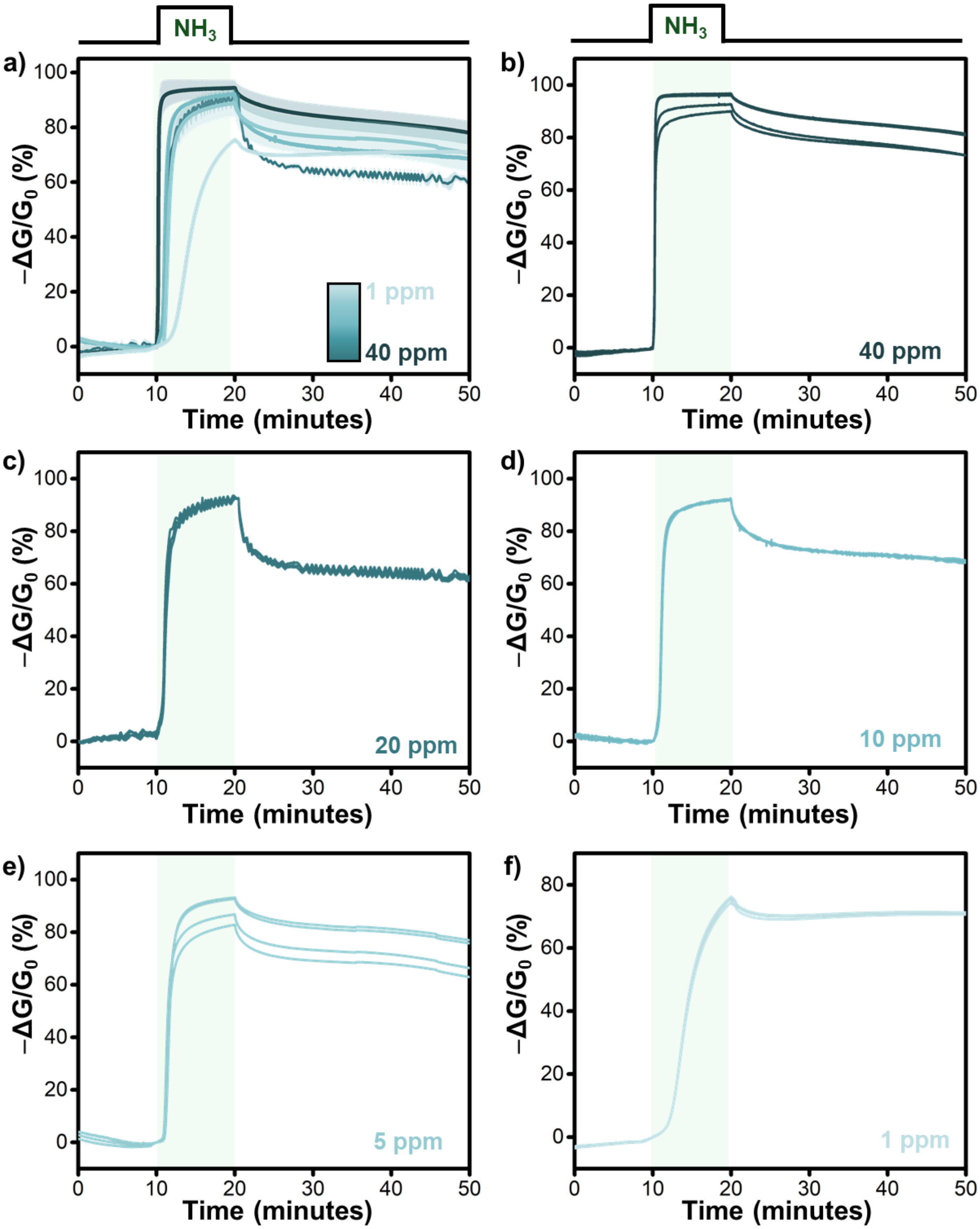

Figure S48. a) Averaged sensing responses of DC-104 towards NH_3 at different concentrations. The teal shaded area represents the standard deviation of sensing responses from 3 devices. Sensing responses of at least 3 devices of DC-104 towards b) 40 ppm, c) 20 ppm, d) 10 ppm, e) 5 ppm, and f) 1 ppm of NH_3. The green shaded area represents the time of exposure of the devices to NH_3.

S5.5. SO_2 gas sensing

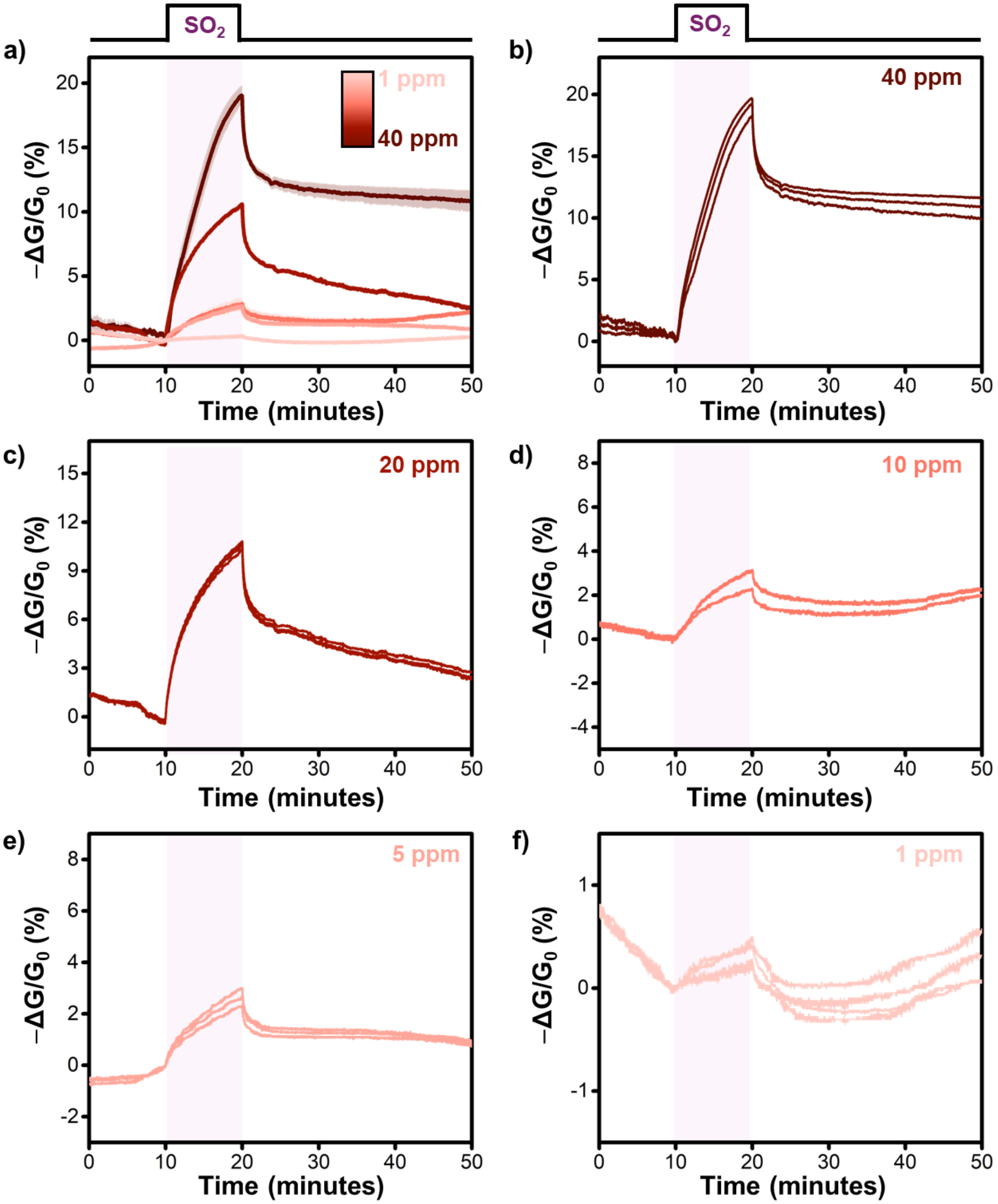

Figure S49. a) Averaged sensing responses of DC-103 towards SO_2 at different concentrations. The orange shaded area represents the standard deviation of sensing responses from 3 devices. Sensing responses of at least 3 devices of DC-103 towards b) 40 ppm, c) 20 ppm, d) 10 ppm, e) 5 ppm, and f) 1 ppm of SO_2. The pink shaded area represents the time of exposure of the devices to SO_2.

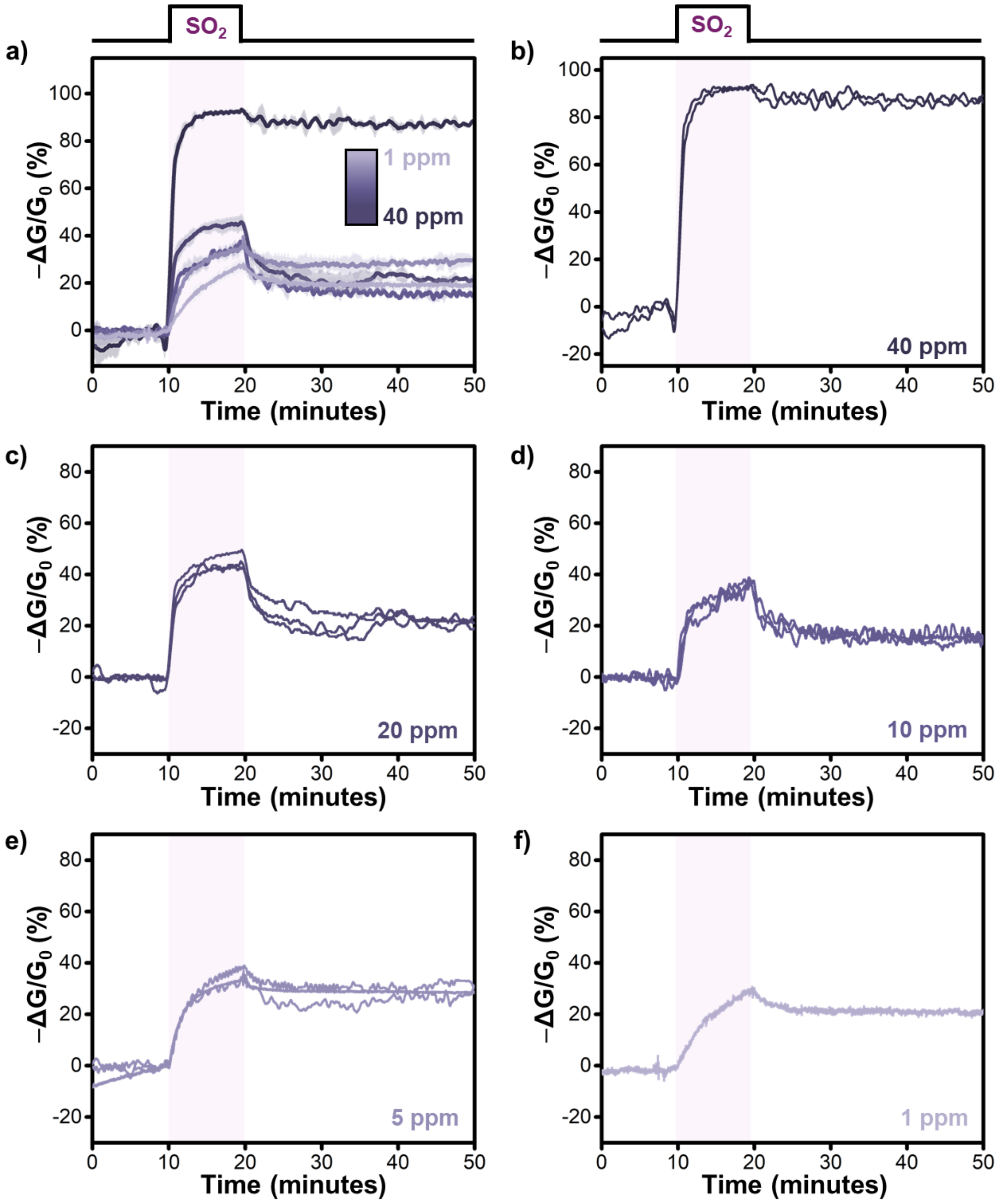

Figure S50. a) Averaged sensing responses of DC-100 towards SO_2 at different concentrations. The purple shaded area represents the standard deviation of sensing responses from 3 devices. Sensing responses of at least 3 devices of DC-100 towards b) 40 ppm, c) 20 ppm, d) 10 ppm, e) 5 ppm, and f) 1 ppm of SO_2. The pink shaded area represents the time of exposure of the devices to SO_2.

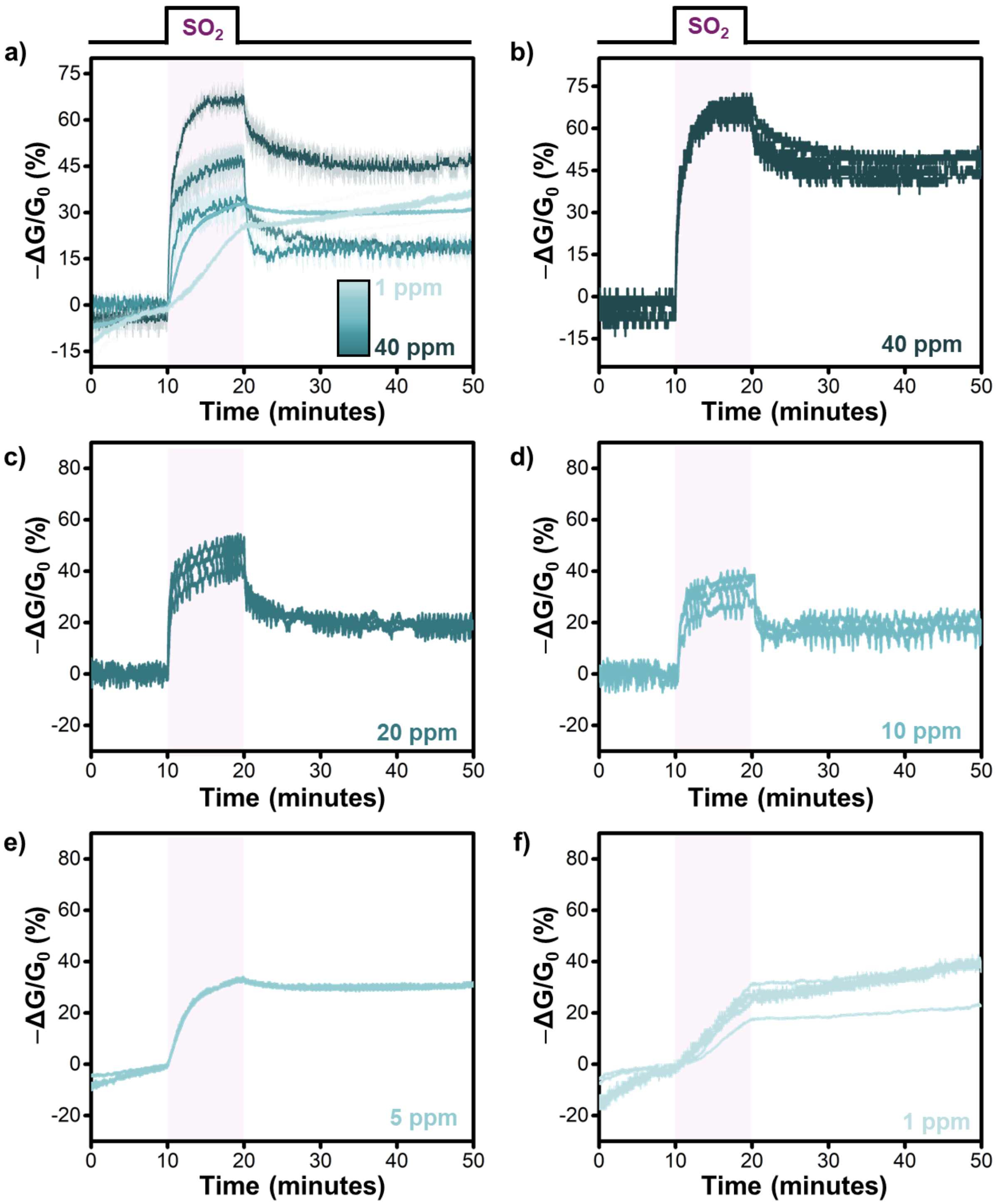

Figure S51. a) Averaged sensing responses of DC-104 towards SO_2 at different concentrations. The teal shaded area represents the standard deviation of sensing responses from 3 devices. Sensing responses of at least 3 devices of DC-104 towards b) 40 ppm, c) 20 ppm, d) 10 ppm, e) 5 ppm, and f) 1 ppm of SO_2. The pink shaded area represents the time of exposure of the devices to SO_2.

S5.6. NO gas sensing

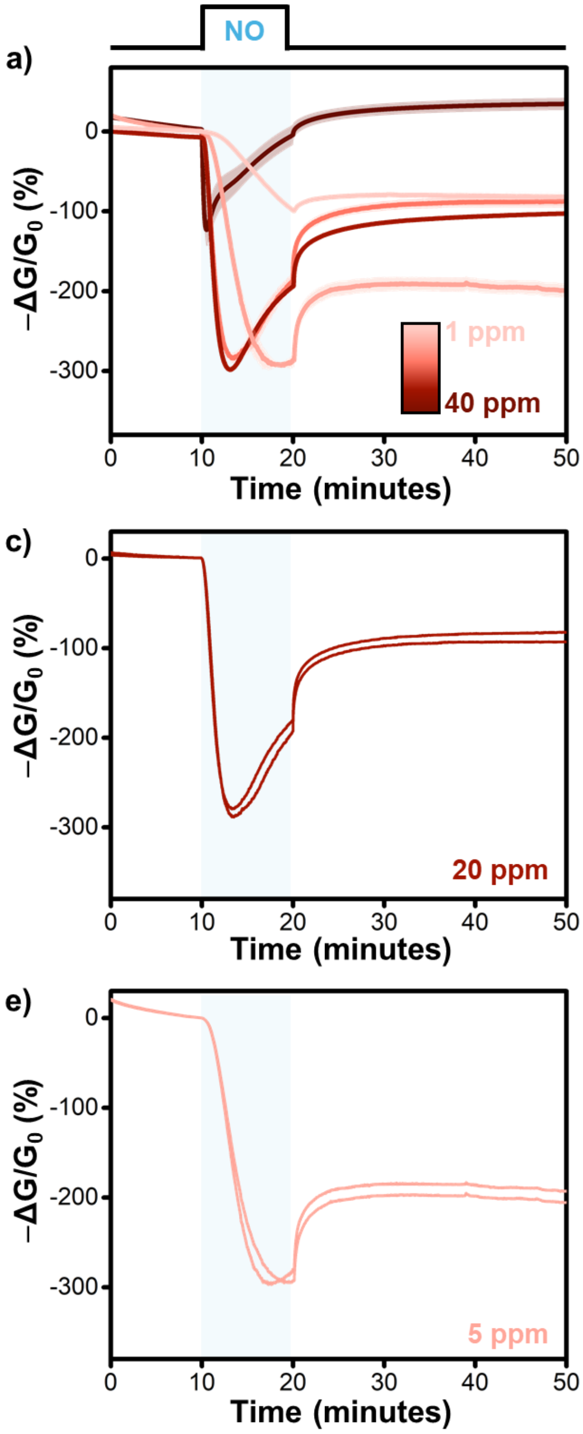

Figure S52. a) Averaged sensing responses of DC-103 towards NO at different concentrations. The orange shaded area represents the standard deviation of sensing responses from 3 devices. Sensing responses of at least 3 devices of DC-103 towards b) 40 ppm, c) 20 ppm, d) 10 ppm, e) 5 ppm, and f) 1 ppm of NO. The blue shaded area represents the time of exposure of the devices to NO.

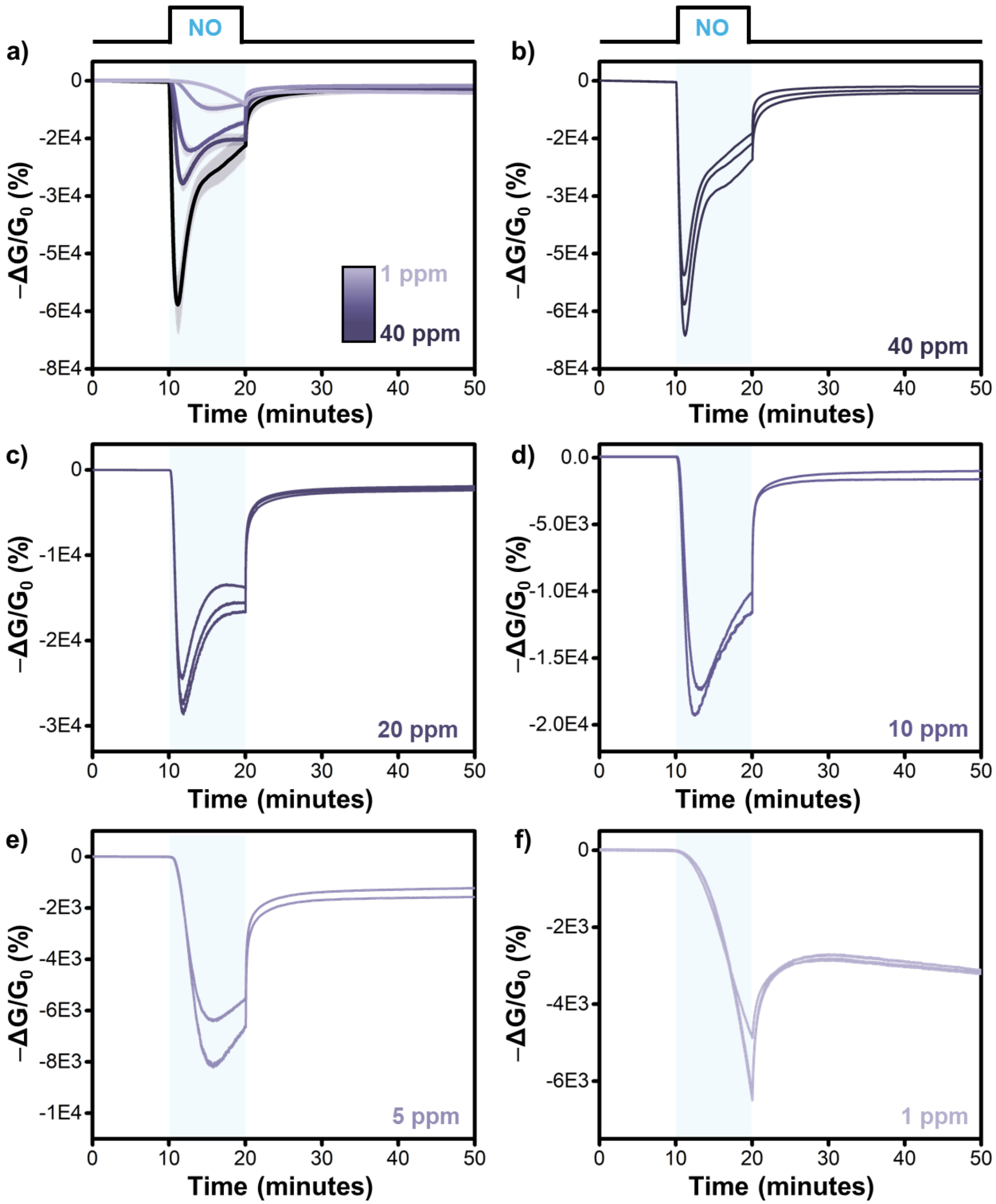

Figure S53. a) Averaged sensing responses of DC-100 towards NO at different concentrations. The purple shaded area represents the standard deviation of sensing responses from 3 devices. Sensing responses of at least 3 devices of DC-100 towards b) 40 ppm, c) 20 ppm, d) 10 ppm, e) 5 ppm, and f) 1 ppm of NO. The blue shaded area represents the time of exposure of the devices to NO.

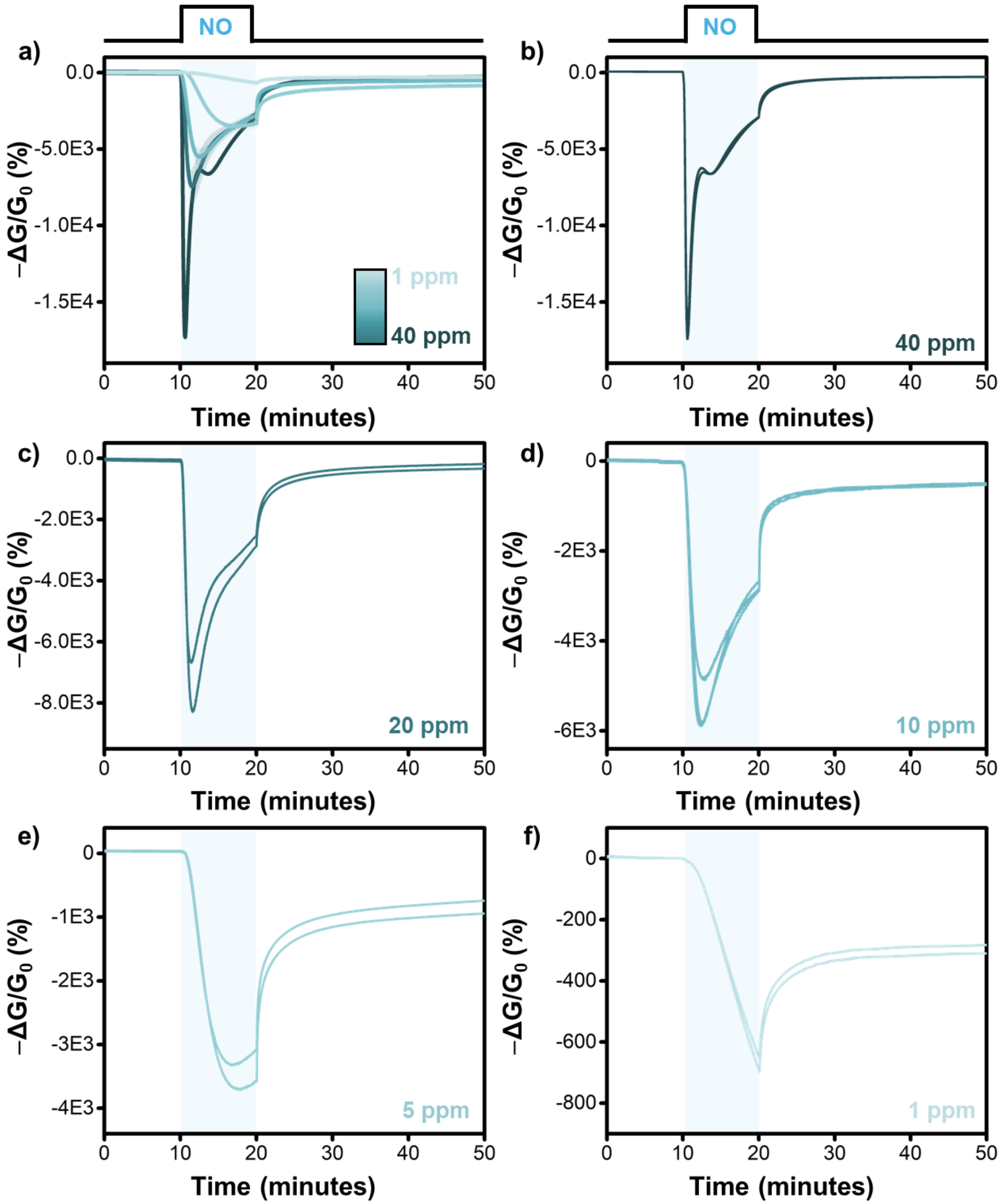

Figure S54. a) Averaged sensing responses of DC-104 towards NO at different concentrations. The teal shaded area represents the standard deviation of sensing responses from 3 devices. Sensing responses of at least 3 devices of DC-104 towards b) 40 ppm, c) 20 ppm, d) 10 ppm, e) 5 ppm, and f) 1 ppm of NO. The blue shaded area represents the time of exposure of the devices to NO.

S5.7. Limits of Detection (LODs)

LODs were determined according to the calculations described by Ammu *et al.*[16]

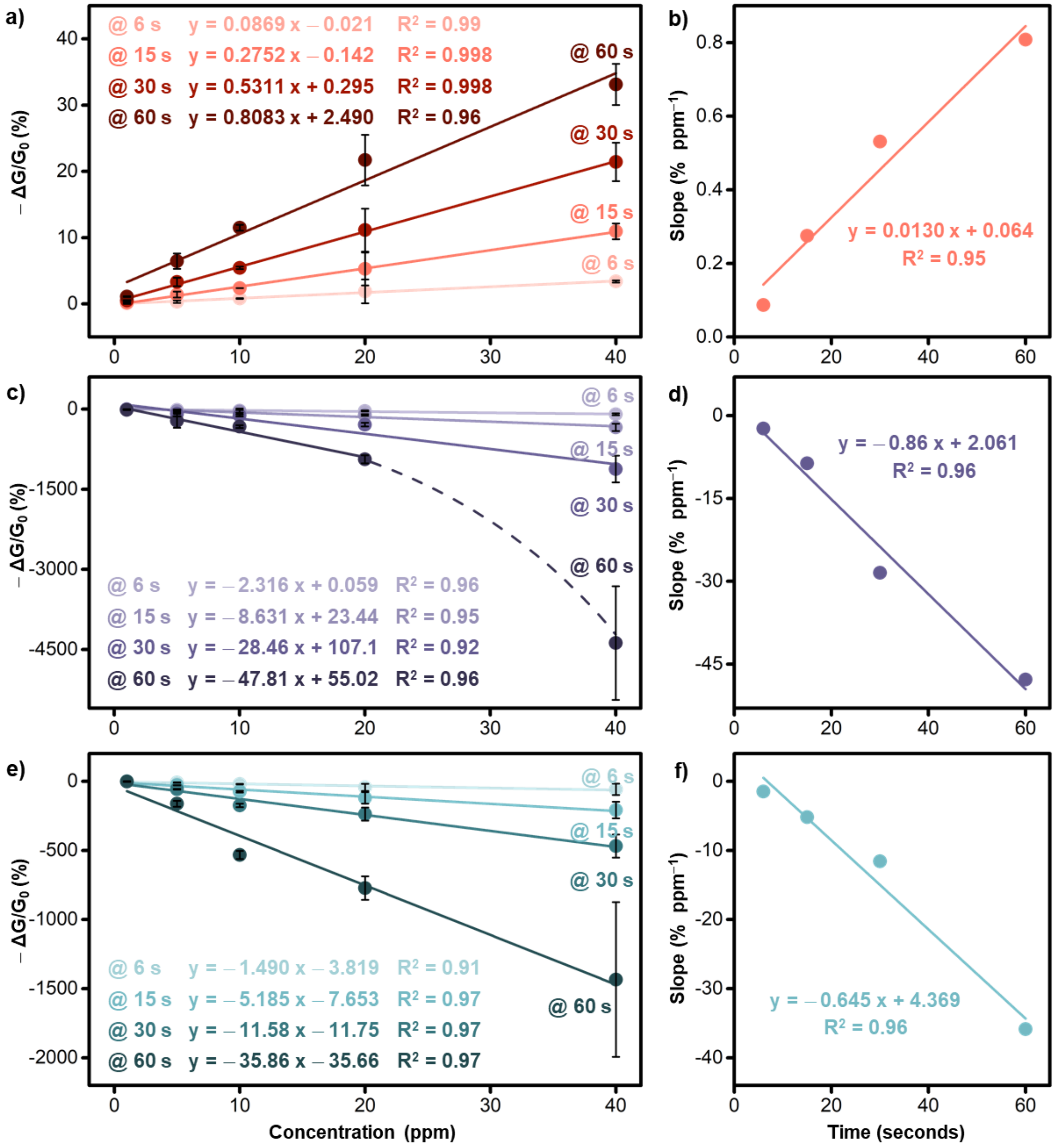

Figure S55. Linear concentration-dependent responses at 6, 15, 30, and 60 seconds of exposure to H_2S in N_2 and their respective slopes vs time by a, b) DC-103, c, d) DC-100, and e, f) DC-104.

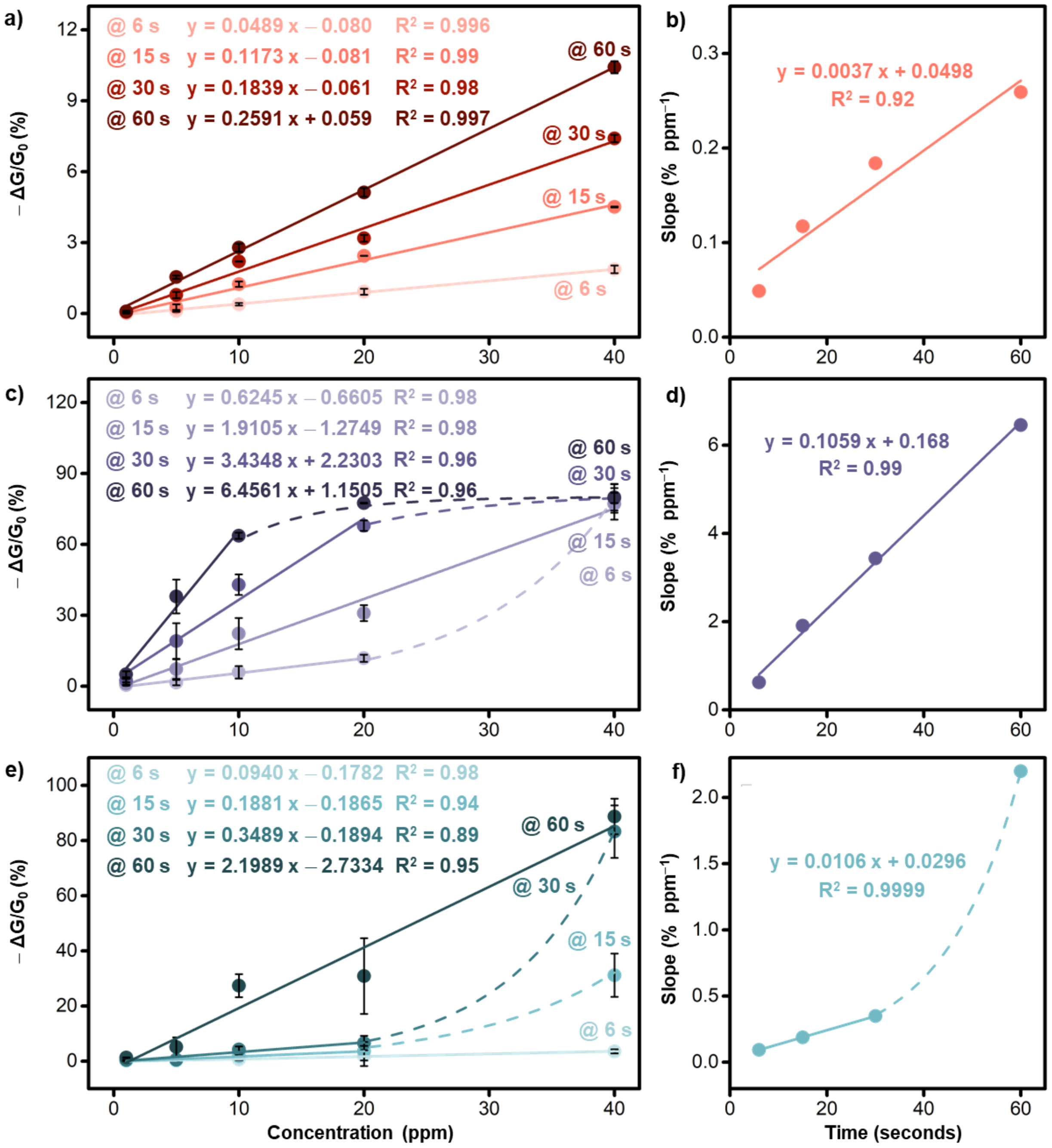

Figure S56. Linear concentration-dependent responses at 6, 15, 30, and 60 seconds of exposure to NH_3 in N_2 and their respective slopes vs time by a, b) DC-103, c, d) DC-100, and e, f) DC-104.

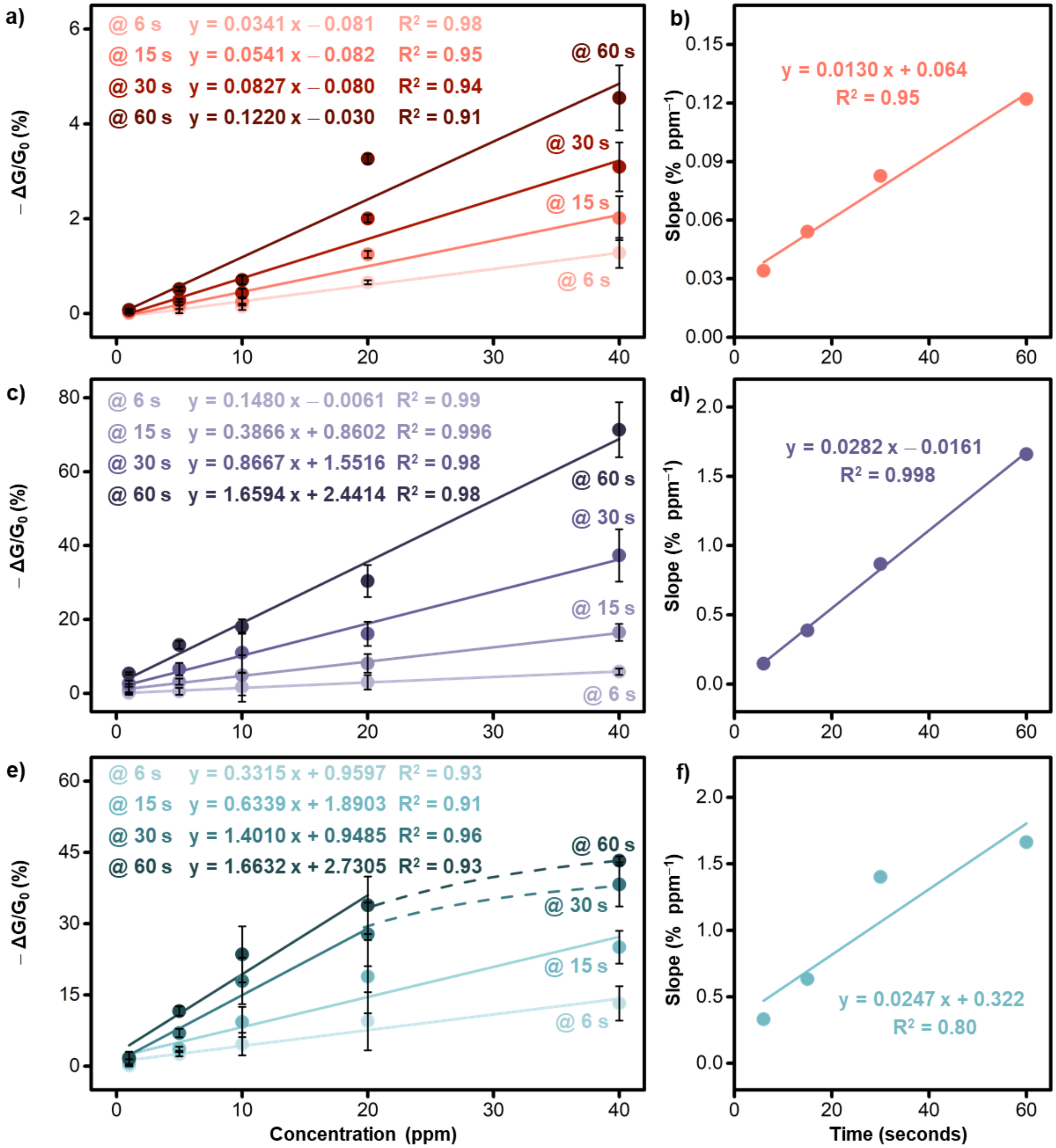

Figure S57. Linear concentration-dependent responses at 6, 15, 30, and 60 seconds of exposure to SO_2 in N_2 and their respective slopes vs time by a, b) DC-103, c, d) DC-100, and e, f) DC-104.

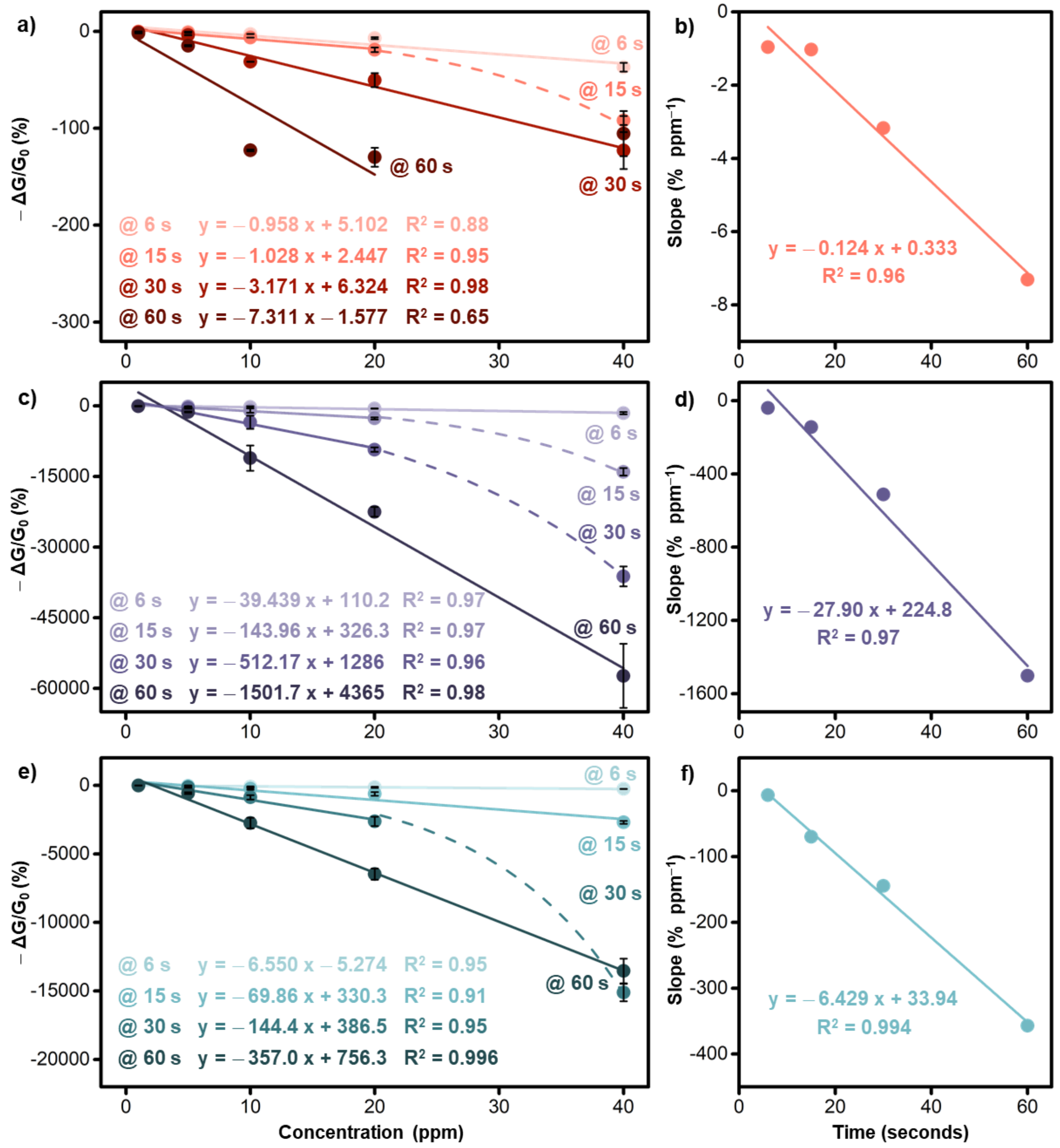

Figure S58. Linear concentration-dependent responses at 6, 15, 30, and 60 seconds of exposure to NO in N_2 and their respective slopes vs time by a, b) DC-103, c, d) DC-100, and e, f) DC-104.

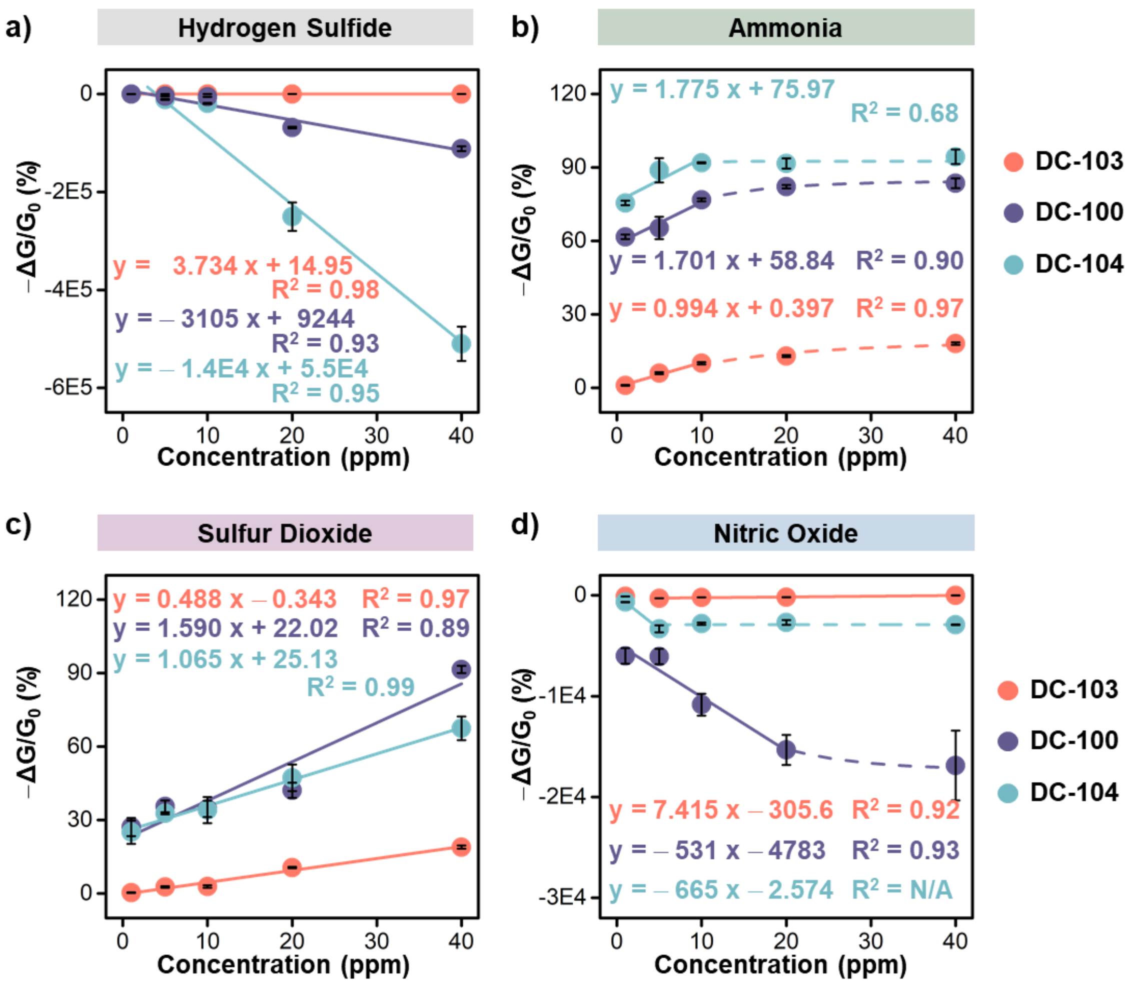

Figure S59. Linear concentration-dependent responses of DC-103, DC-100 and DC-104 at 10 minutes of exposure to a) H_2S, b) NH_3, c) SO_2, and d) NO in N_2.

Table S7. Theoretical LODs of the three MOF analogs at 6, 15, 30, and 60 seconds as well as upon 10 minutes of exposure to H_2S, NH_3, SO_2, and NO in N_2.

Exposure time		6 s	15 s	30 s	60 s	10 min
H_2S LOD (ppb)	DC-103	711	224	116	76	16.5
	DC-100	848	227	69	41	0.63
	DC-104	974	280	125	40	0.10
NH_3 LOD (ppb)	DC-103	610	254	162	115	30.0
	DC-100	702	229	128	68	258
	DC-104	1.2 ppm	596	321	51	63.2
SO_2 LOD (ppb)	DC-103	2.5 ppm	1.6 ppm	1.0 ppm	688	172
	DC-100	5.5 ppm	3.6 ppm	1.6 ppm	840	878
	DC-104	4.6 ppm	3.6 ppm	1.7 ppm	1.4 ppm	2.2 ppm
NO LOD (ppb)	DC-103	130	121	39	17.1	16.8
	DC-100	52	14	4.0	1.4	3.9
	DC-104	31	2.9	1.4	0.57	0.30

S5.8. Initial Rates

Initial rates were determined by plotting the linear fit of the initial response for each MOF towards the different concentrations of gases under study. In most cases, the linear fit is for the first minute of response, unless the change in response is not completely linear across the whole first minute, in which the initial linear portion is taken into consideration.

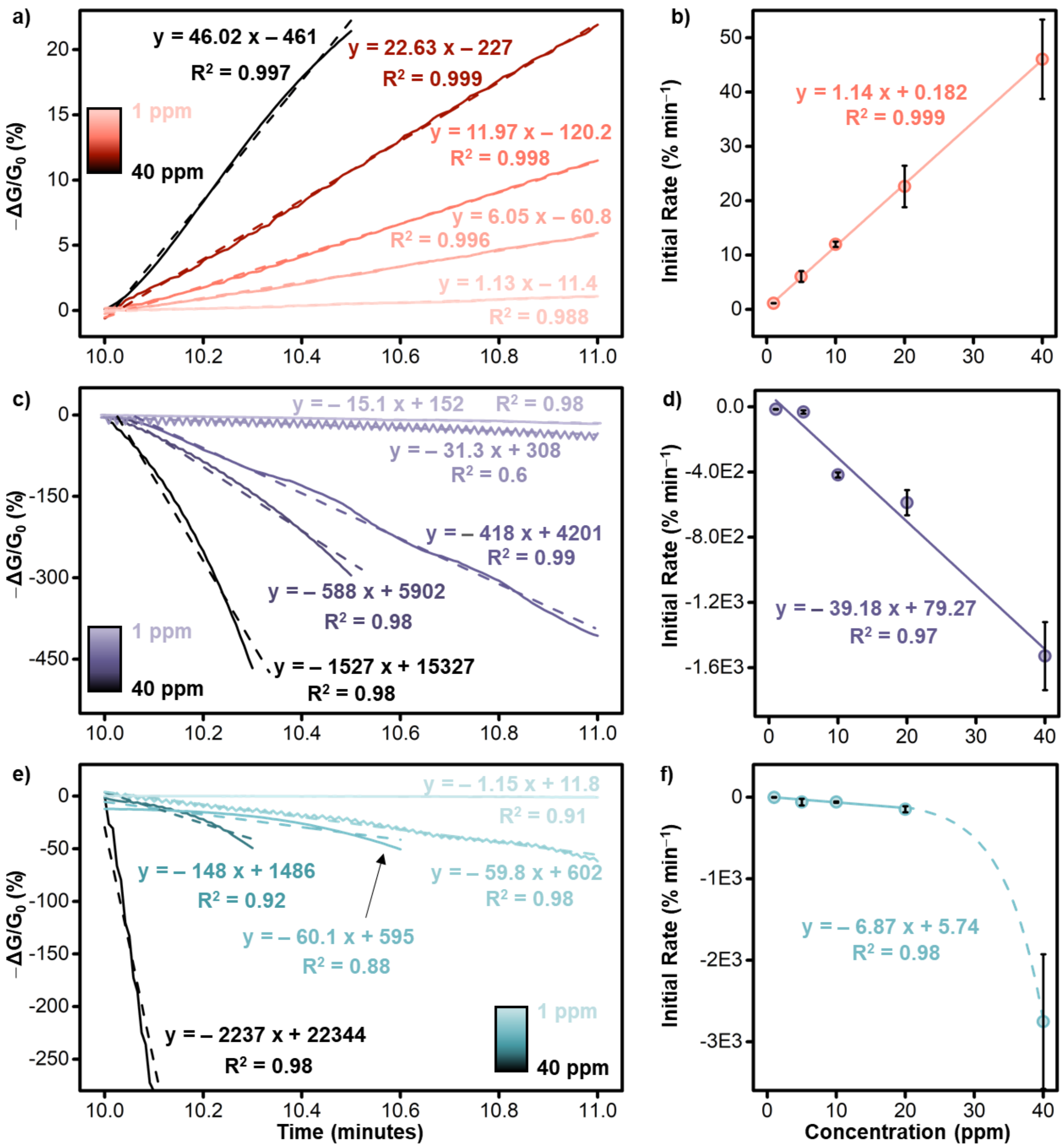

Figure S60. Sensing responses towards different concentrations of H_2S in N_2 during the first minute of exposure and their respective linear fits as well as linear relationships between the slope of the linear fits as a function of concentration of H_2S for a, b) DC-103, c, d) DC-100, and e, f) DC-104.

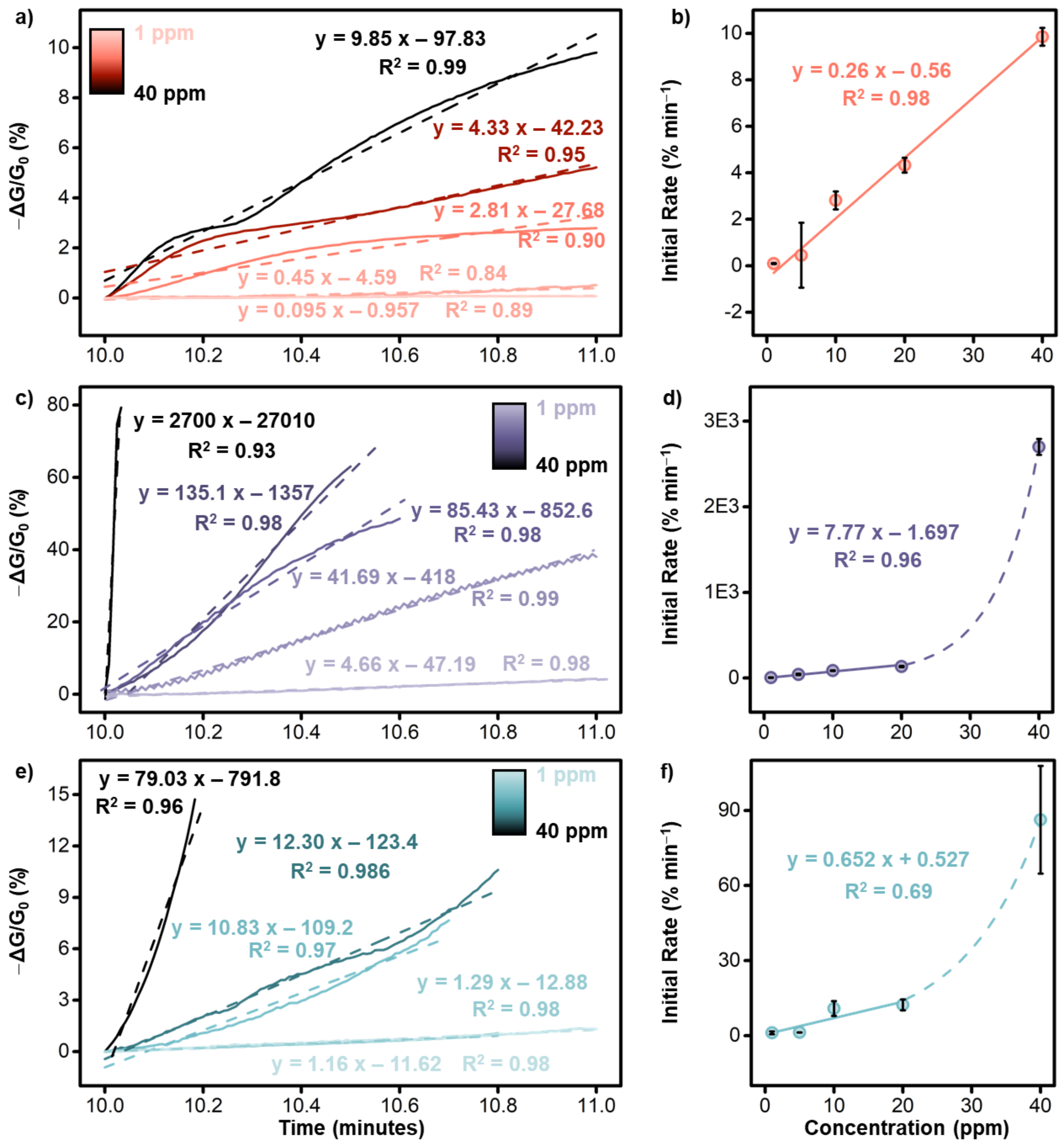

Figure S61. Sensing responses towards different concentrations of NH_3 in N_2 during the first minute of exposure and their respective linear fits as well as linear relationships between the slope of the linear fits as a function of concentration of NH_3 for a, b) DC-103, c, d) DC-100, and e, f) DC-104.

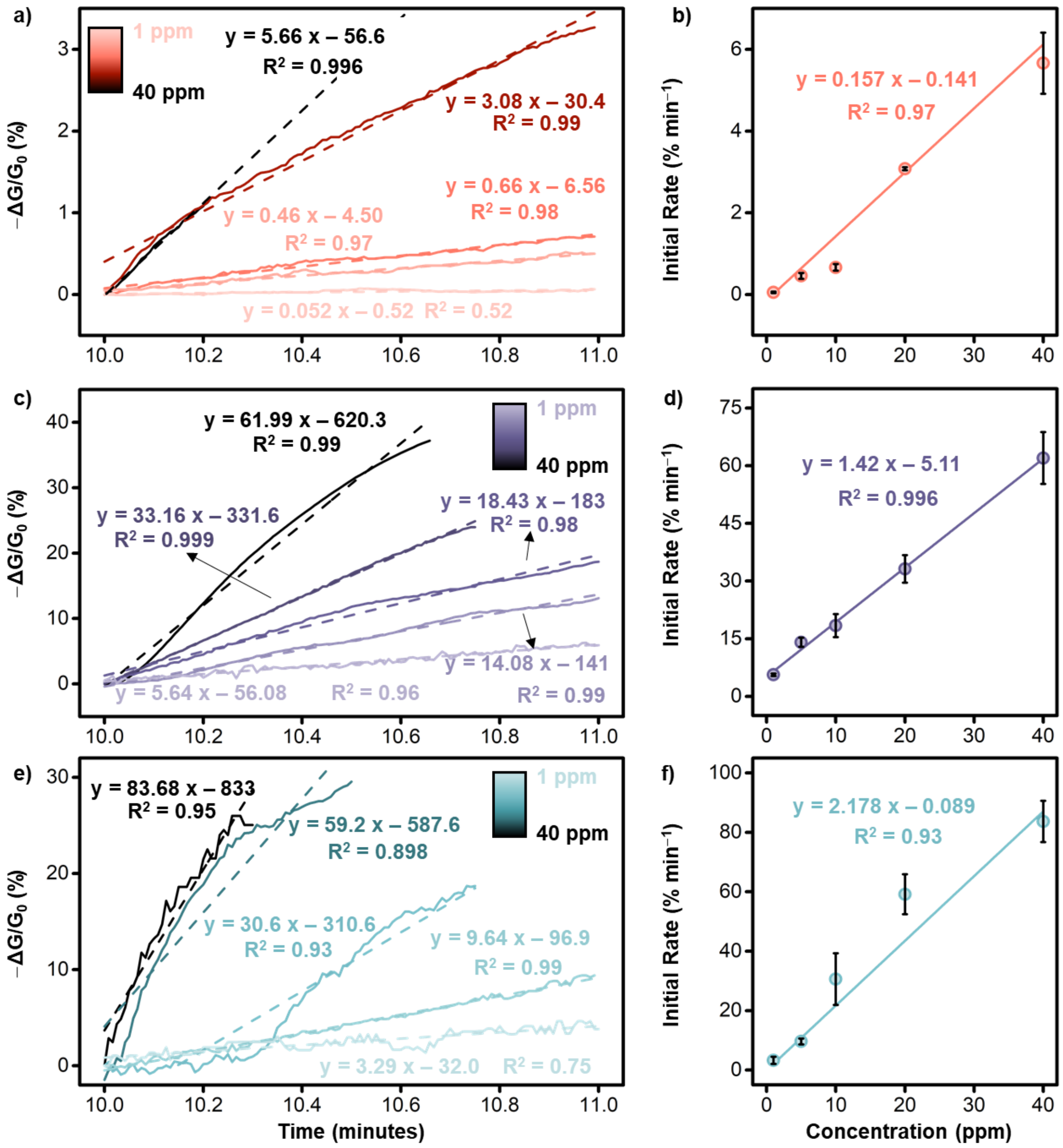

Figure S62. Sensing responses towards different concentrations of SO_2 in N_2 during the first minute of exposure and their respective linear fits as well as linear relationships between the slope of the linear fits as a function of concentration of SO_2 for a, b) DC-103, c, d) DC-100, and e, f) DC-104.

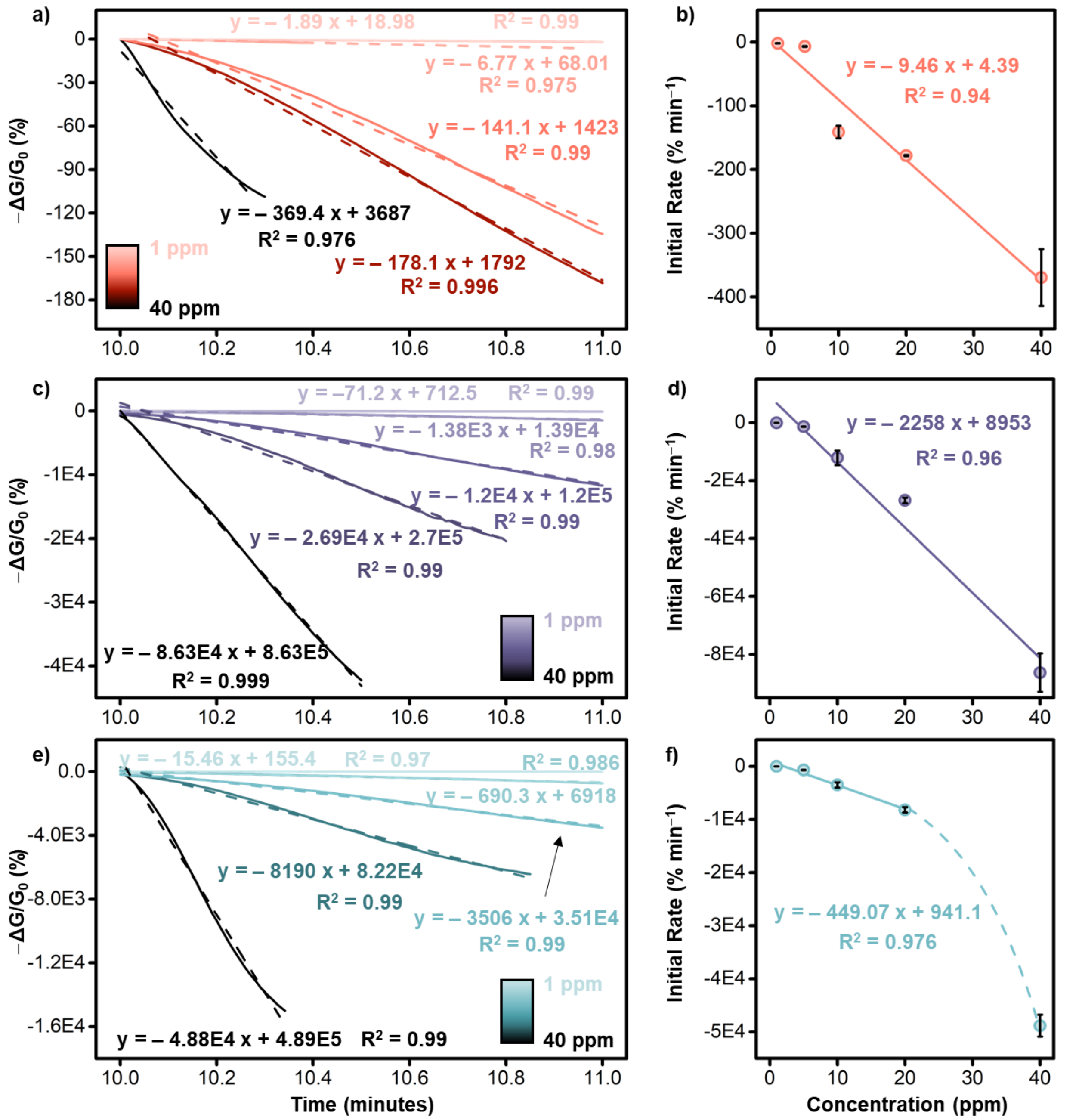

Figure S63. Sensing responses towards different concentrations of NO in N_2 during the first minute of exposure and their respective linear fits as well as linear relationships between the slope of the linear fits as a function of concentration of NO for a, b) DC-103, c, d) DC-100, and e, f) DC-104.

S5.9. Recyclability Tests

Measurements for recyclability tests were composed of 13 cycles with an initial 10 min baseline, followed by 10 min of exposure under 20 ppm of gas analyte then 10 min of recovery per cycle. The graphs are plotted similarly to what was explained in **Section S5.2**.

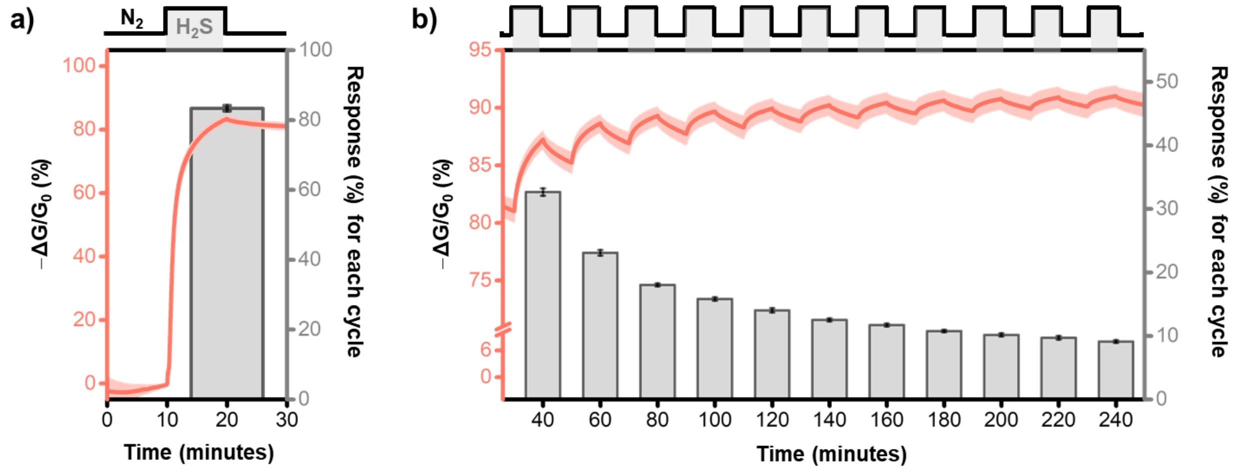

Figure S64. Recyclability experiment of DC-103 towards 20 ppm of H_2S: Averaged sensing response and the percentage response of each individual cycle of a) the 1st exposure and b) the 2nd to 12th exposures of the same electrodes. The orange shaded area represents the standard deviation of sensing responses from 3 devices.

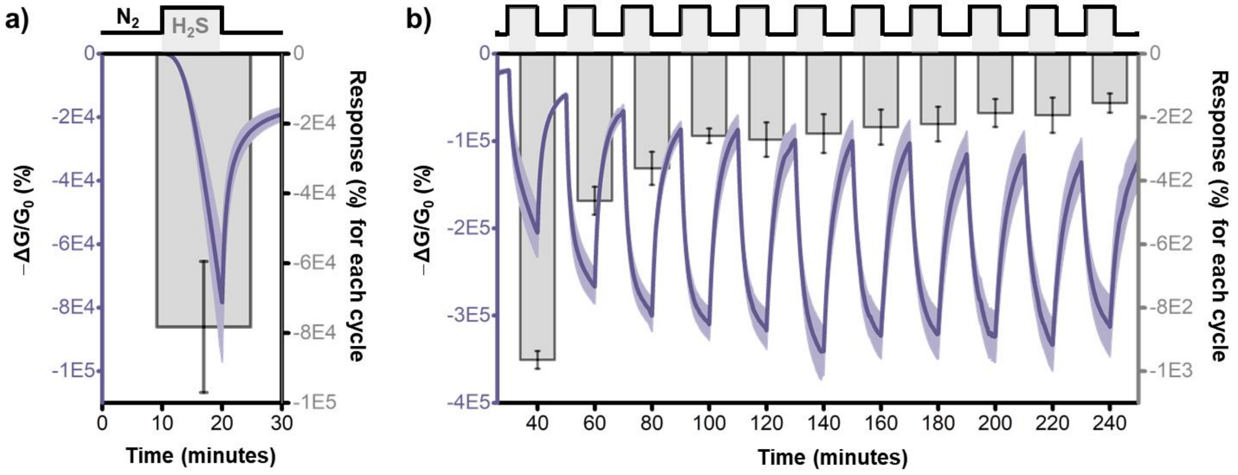

Figure S65. Recyclability experiment of DC-100 towards 20 ppm of H_2S: Averaged sensing response and the percentage response of each individual cycle of a) the 1st exposure and b) the 2nd to 12th exposures of the same electrodes. The purple shaded area represents the standard deviation of sensing responses from 3 devices.

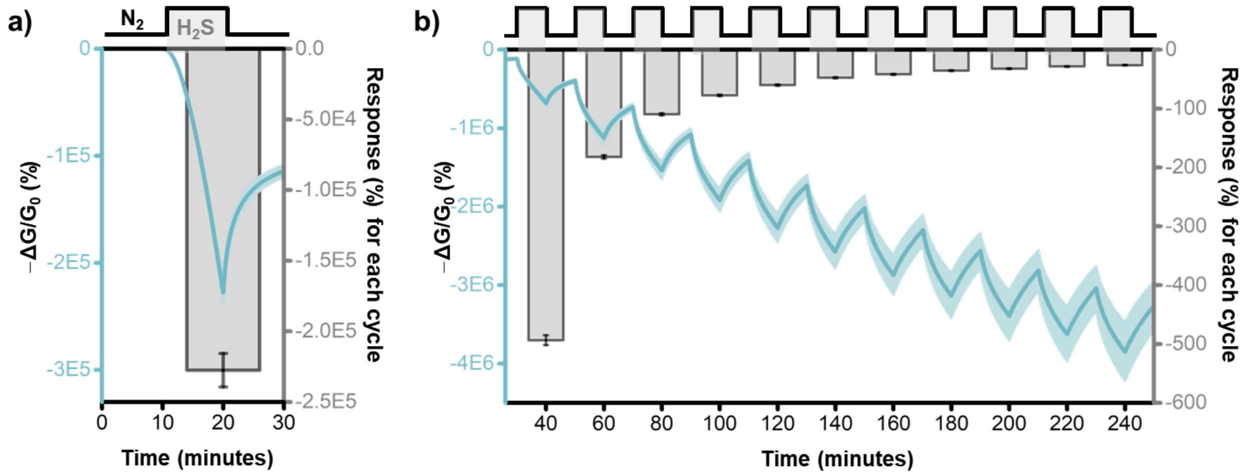

Figure S66. Recyclability experiment of DC-104 towards 20 ppm H_2S: Averaged sensing response and the percentage response of each individual cycle of a) the 1st exposure and b) the 2nd to 12th exposures of the same electrodes. The teal shaded area represents the standard deviation of sensing responses from 3 devices.

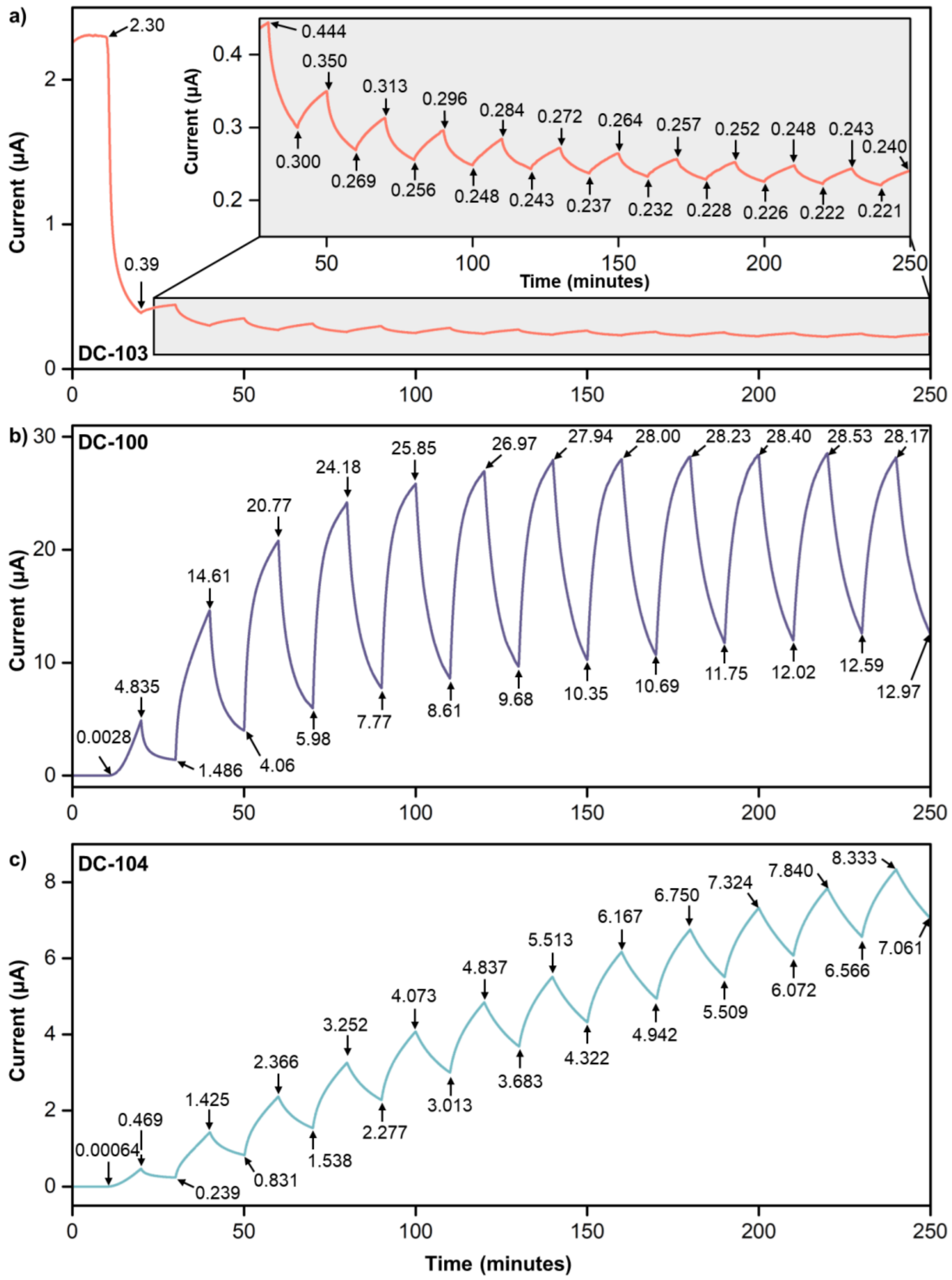

Figure S67. Representative raw current–time traces from **Figures S64-S66** showing the sensing responses of a) DC-103, b) DC-100, and c) DC-104 upon exposure to 20 ppm of H_2S. For clarity, data from a single representative device (out of three measured) are shown. The traces illustrate current changes during repeated exposure and recovery cycles, highlighting partial recovery while maintaining a measurable response to H_2S over multiple cycles.

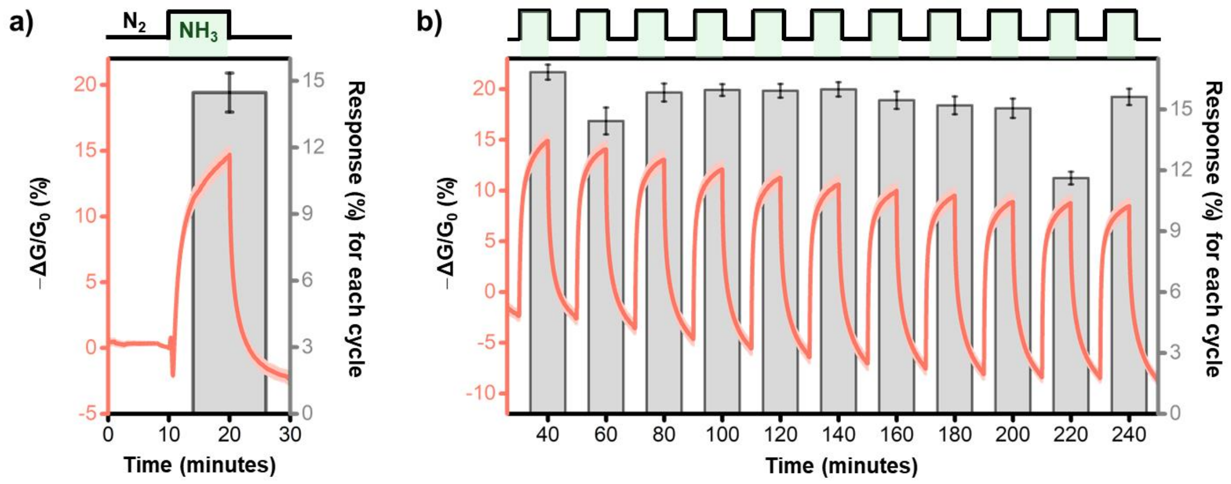

Figure S68. Recyclability experiment of DC-103 towards 20 ppm of NH_3: Averaged sensing response and the percentage response of each individual cycle of a) the 1st exposure and b) the 2nd to 12th exposures of the same electrodes. The orange shaded area represents the standard deviation of sensing responses from 3 devices.

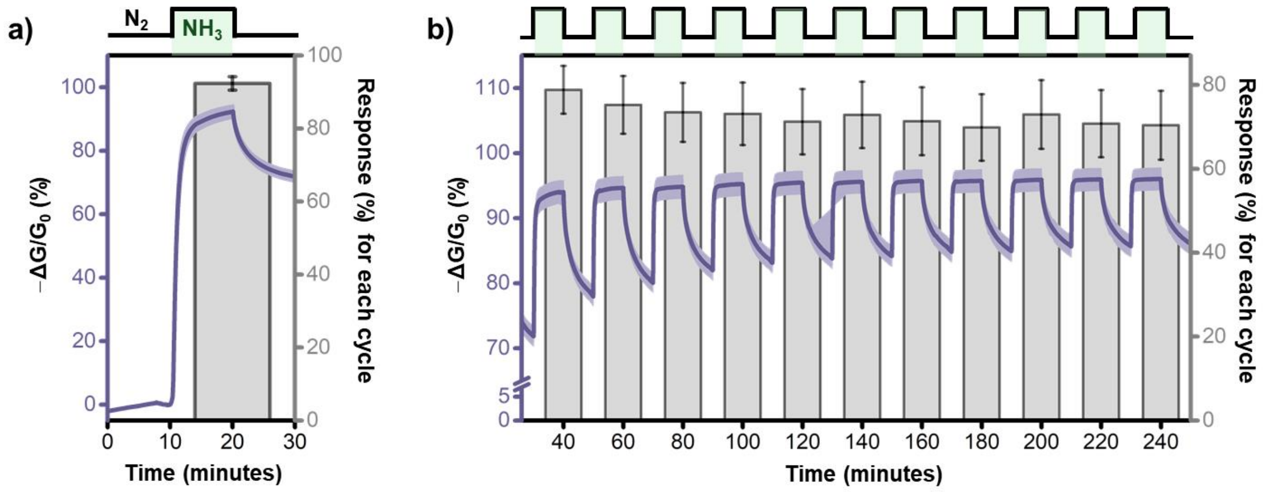

Figure S69. Recyclability experiment of DC-100 towards 20 ppm of NH_3: Averaged sensing response and the percentage response of each individual cycle of a) the 1st exposure and b) the 2nd to 12th exposures of the same electrodes. The purple shaded area represents the standard deviation of sensing responses from 3 devices.

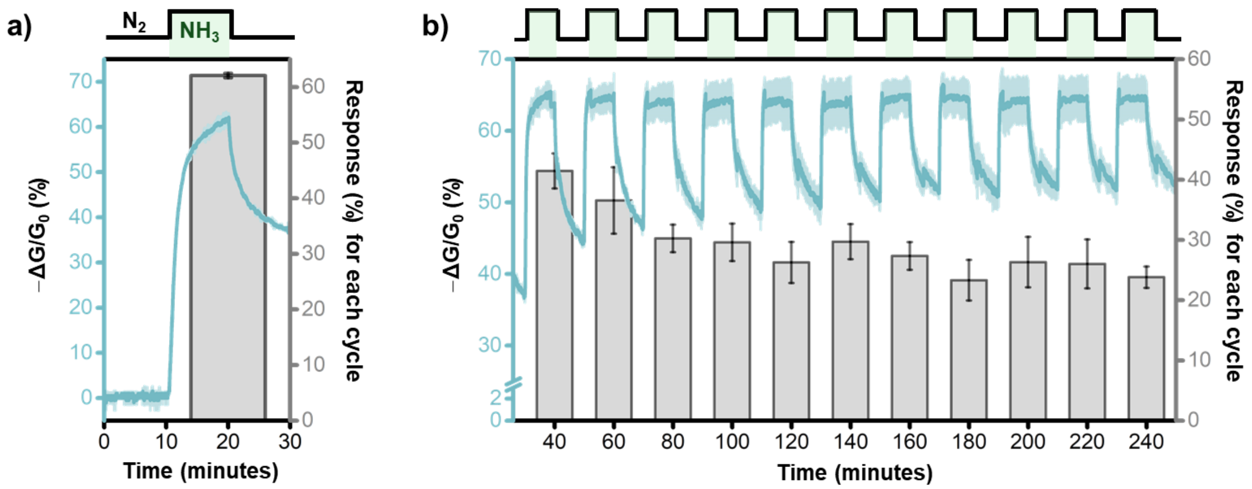

Figure S70. Recyclability experiment of DC-104 towards 20 ppm of NH_3: Averaged sensing response and the percentage response of each individual cycle of a) the 1st exposure and b) the 2nd to 12th exposures of the same electrodes. The teal shaded area represents the standard deviation of sensing responses from 3 devices.

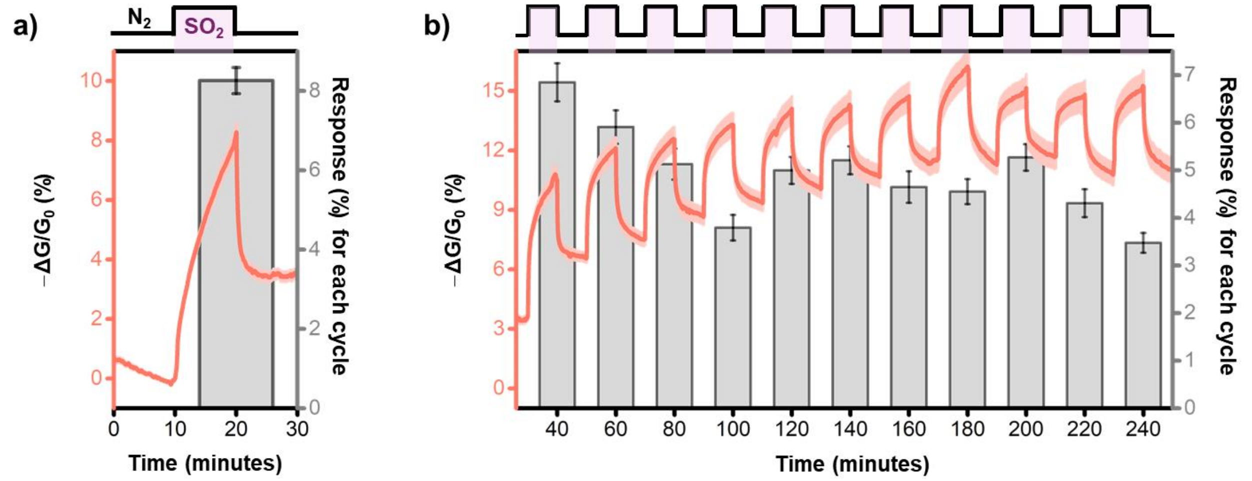

Figure S71. Recyclability experiment of DC-103 towards 20 ppm of SO_2: Averaged sensing response and the percentage response of each individual cycle of a) the 1st exposure and b) the 2nd to 12th exposures of the same electrodes. The orange shaded area represents the standard deviation of sensing responses from 3 devices.

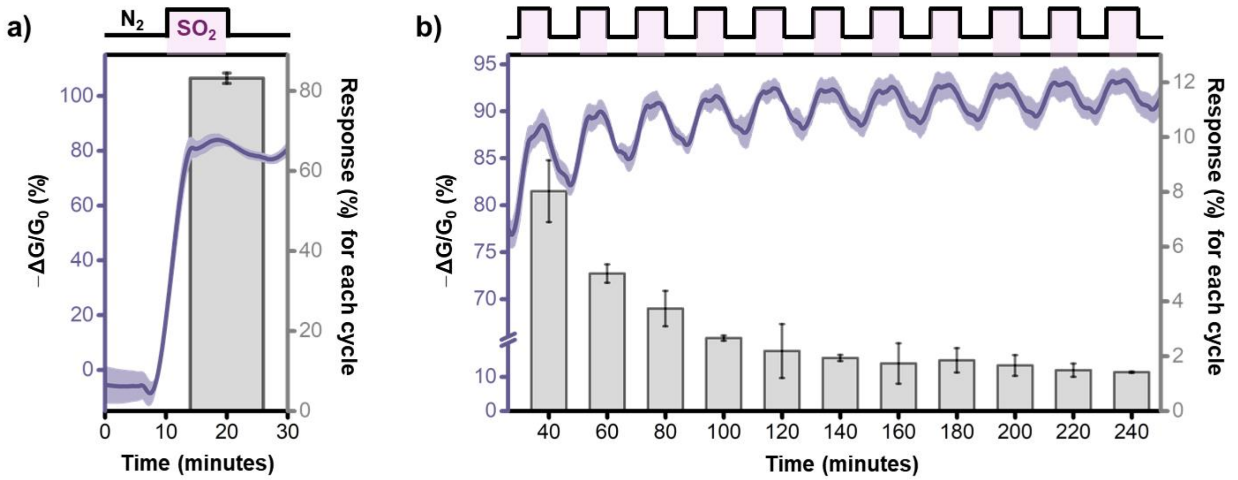

Figure S72. Recyclability experiment of DC-100 towards 20 ppm of SO_2: Averaged sensing response and the percentage response of each individual cycle of a) the 1st exposure and b) the 2nd to 12th exposures of the same electrodes. The purple shaded area represents the standard deviation of sensing responses from 3 devices.

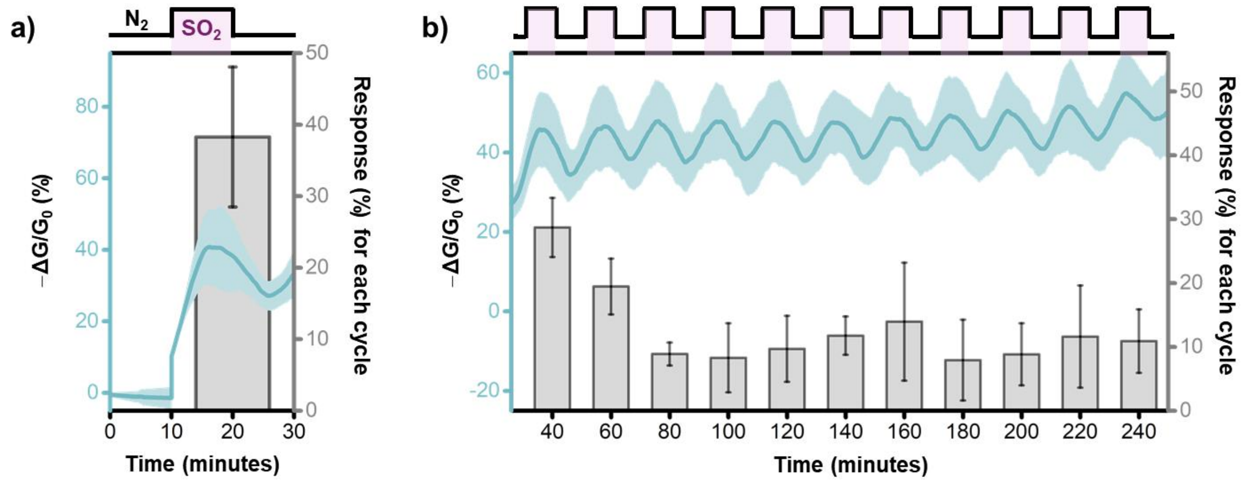

Figure S73. Recyclability experiment of DC-104 towards 20 ppm of SO_2: Averaged sensing response and the percentage response of each individual cycle of a) the 1st exposure and b) the 2nd to 12th exposures of the same electrodes. The teal shaded area represents the standard deviation of sensing responses from 3 devices.

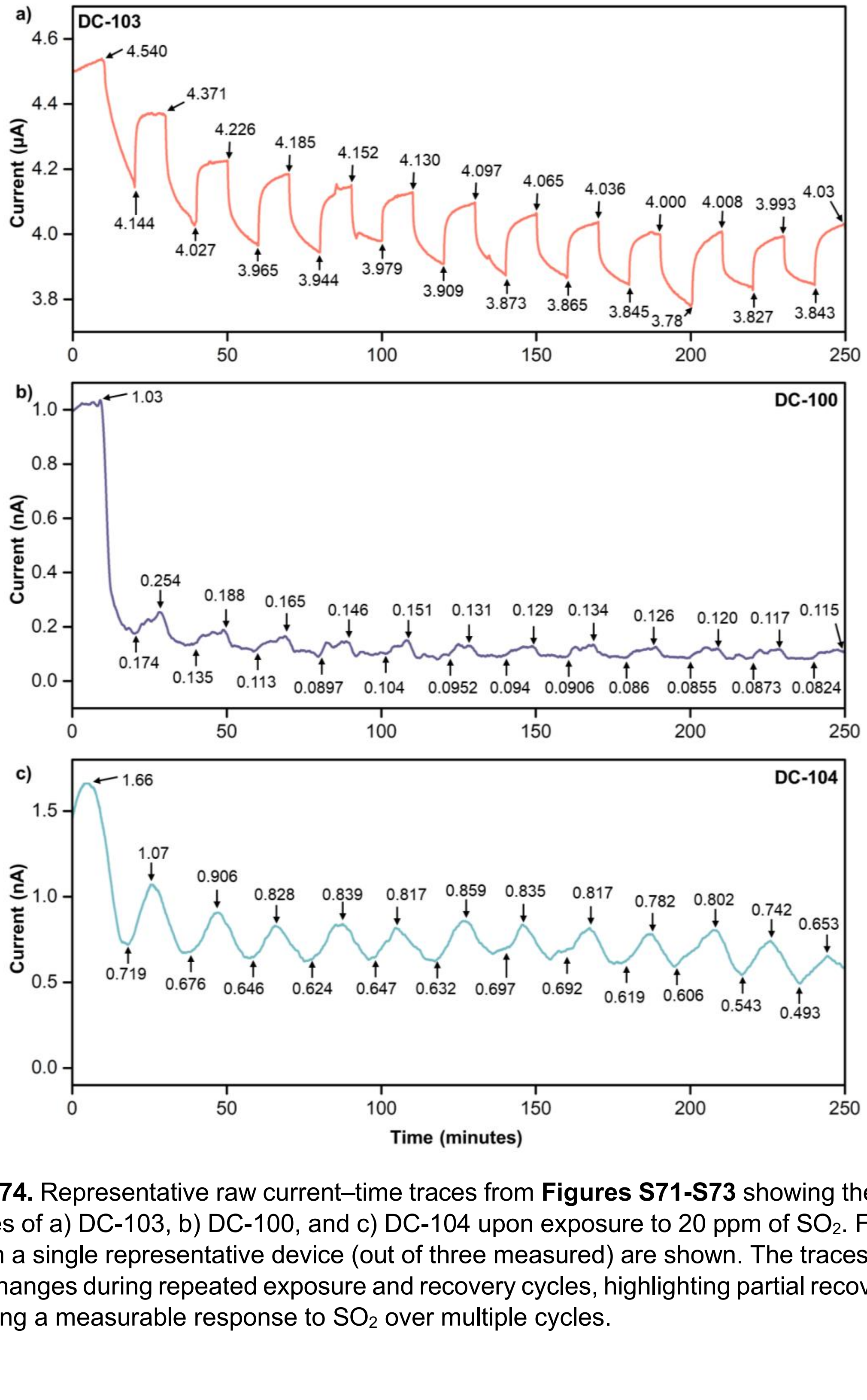

Figure S74. Representative raw current–time traces from **Figures S71-S73** showing the sensing responses of a) DC-103, b) DC-100, and c) DC-104 upon exposure to 20 ppm of SO_2. For clarity, data from a single representative device (out of three measured) are shown. The traces illustrate current changes during repeated exposure and recovery cycles, highlighting partial recovery while maintaining a measurable response to SO_2 over multiple cycles.

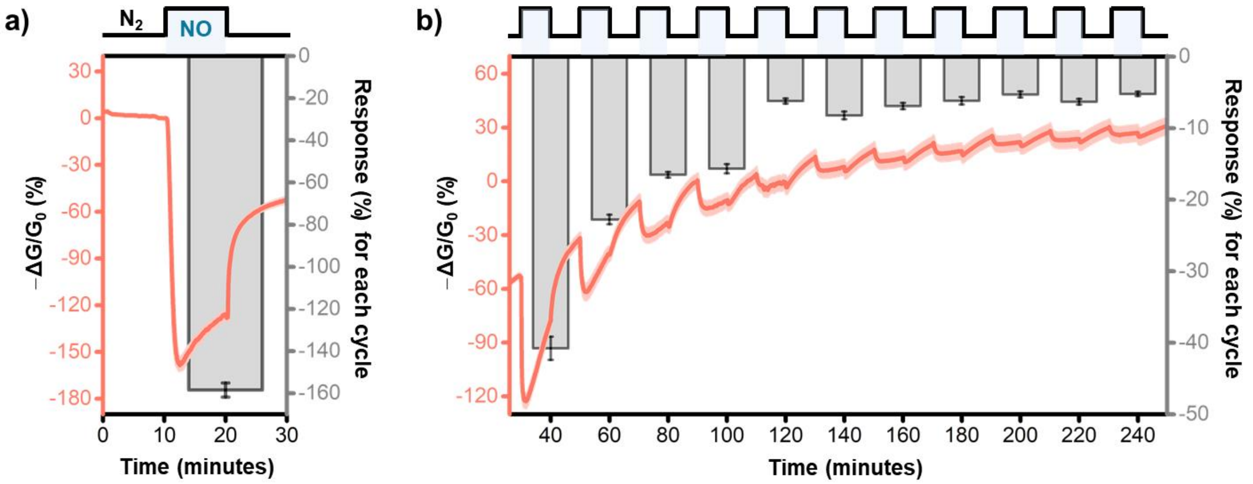

Figure S75. Recyclability experiment of DC-103 towards 20 ppm of NO: Averaged sensing response and the percentage response of each individual cycle of a) the 1st exposure and b) the 2nd to 12th exposures of the same electrodes. The orange shaded area represents the standard deviation of sensing responses from 3 devices.

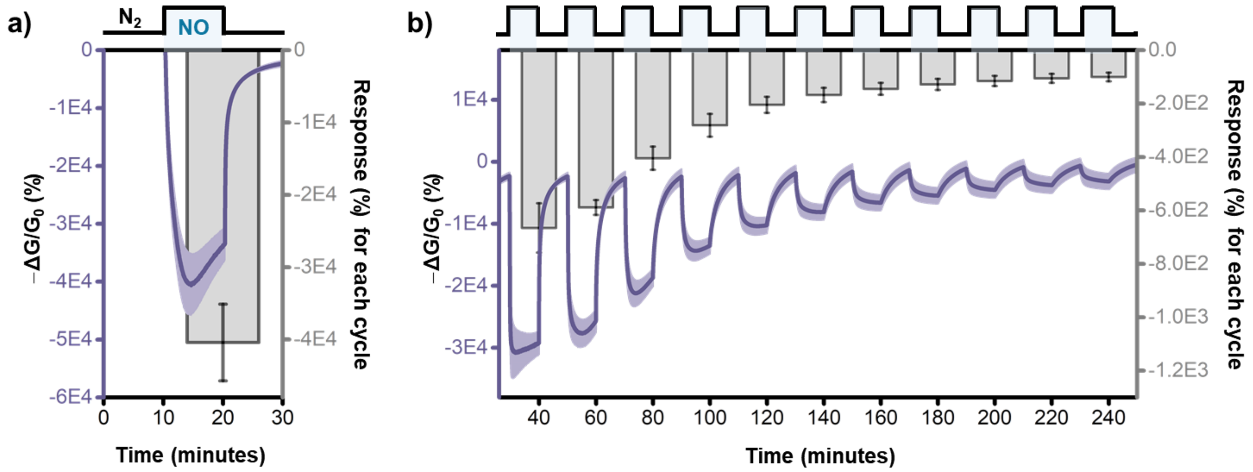

Figure S76. Recyclability experiment of DC-100 towards 20 ppm of NO: Averaged sensing response and the percentage response of each individual cycle of a) the 1st exposure and b) the 2nd to 12th exposures of the same electrodes. The purple shaded area represents the standard deviation of sensing responses from 3 devices.

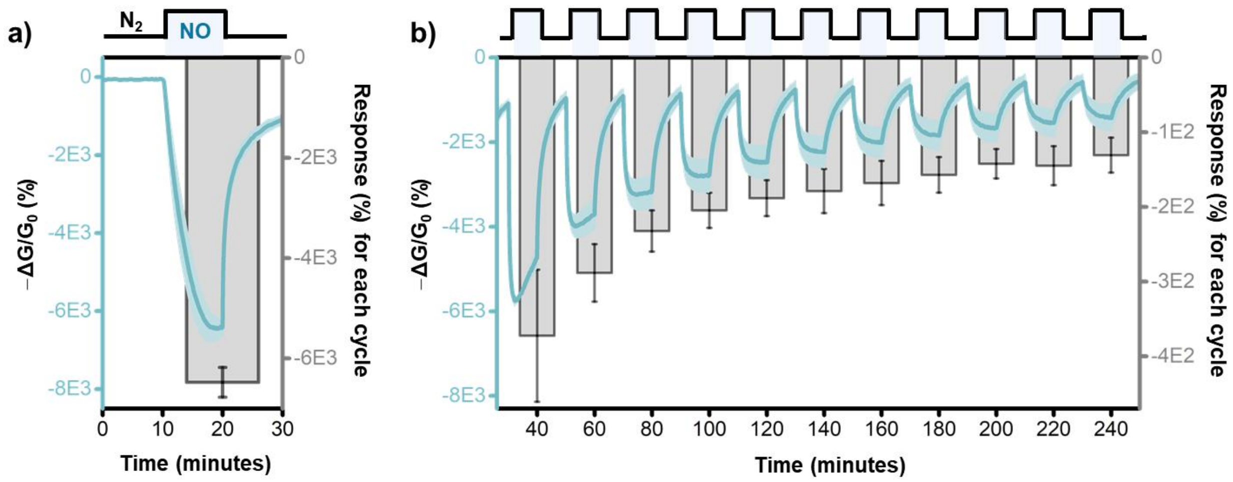

Figure S77. Recyclability experiment of DC-104 towards 20 ppm of NO: Averaged sensing response and the percentage response of each individual cycle of a) the 1st exposure and b) the 2nd to 12th exposures of the same electrodes. The teal shaded area represents the standard deviation of sensing responses from 3 devices.

S5.10. Recovery Calculations

Recovery calculations were performed for each of the MOFs towards the different gases at the tested concentrations according to the following equation:

Equation S5:

$$\%\,Recovery = \frac{I_{max} - I_{after\ recovery}}{I_{after\ recovery}} \times\ 100$$

where I_{max} is the current at maximum response (mainly at 20 minutes, i.e. after 10 minutes of exposure) and $I_{after\ recovery}$ is the current at the end of the recovery period (at 50 minutes, i.e. after 30 minutes of recovery).

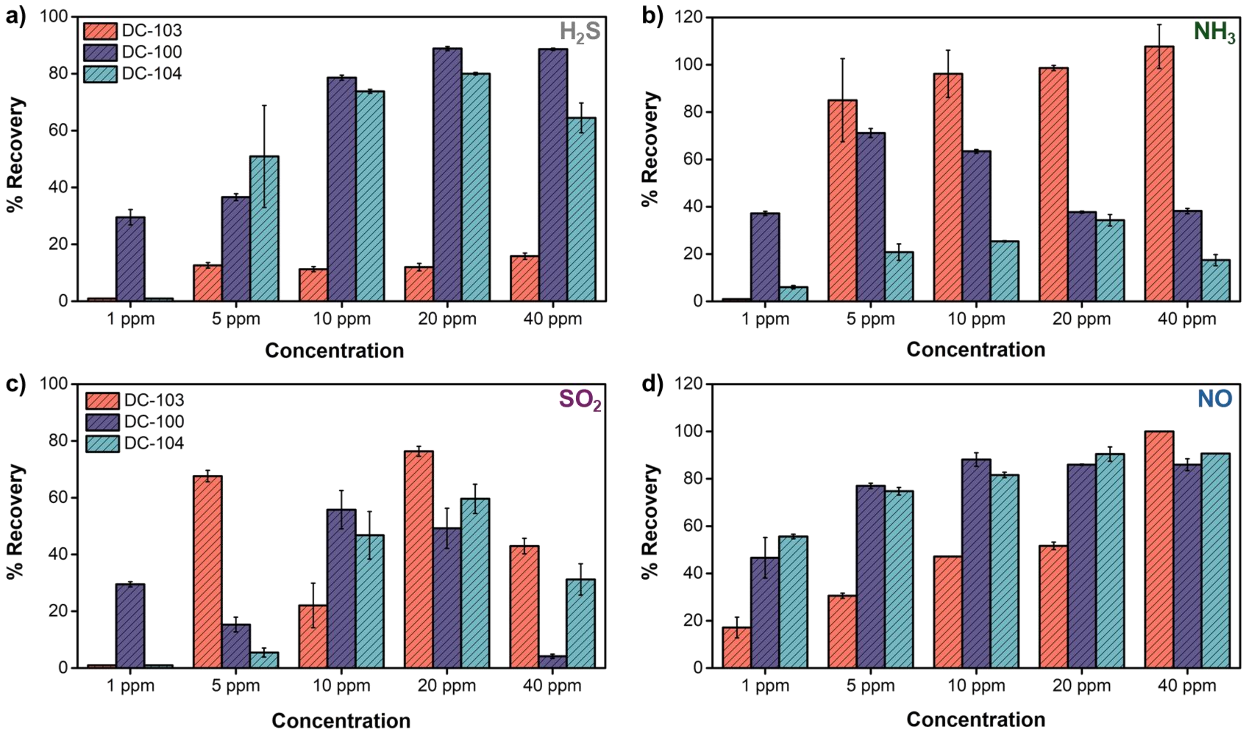

Figure S78. Bar graphs showing the average percent recovery of the three MOFs towards the different concentrations of a) H_2S, b) NH_3, c) SO_2, and d) NO. The error bars represent the standard deviation of sensing responses from at least 3 devices.

S5.11. Comparison to MPz, MPc, and MNPc MOFs

Table S8. Summary of reported sensing response characteristics of MTPz-Cu, MPz-Cu, MPc-Cu, and MNPc-Cu MOFs towards H_2S, NH_3, and NO. No sensing reports for SO_2 using these MOFs have been published to date for comparison with MTPz-Cu-MOFs.

gas	MOF	$-\Delta G/G_0$	concentration			LOD	at	exp time	ref
H_2S	DC-100 (NiTPz-Cu)	-111,350%	at	40 ppm	with	0.63 ppb	at	10 min	This work
	NiPc-O_8-Cu	100%	at	40 ppm	with	1.04 ppb	at	30 min	5
	NiPc-Cu	70%	at	40 ppm	with	175 ppb	at	30 min	17
	NiNPc-Cu	95%	at	40 ppm	with	-	-	-	5
H_2S	DC-104 (CuTPz-Cu)	-510,000%	at	40 ppm	with	0.10 ppb	at	10 min	This work
	CuPc-O-Cu	92%	at	40 ppm	with	69 ppb	at	30 min	17
NH_3	DC-103 (CoTPz-Cu)	18%	at	40 ppm	with	30 ppb	at	10 min	This work
	CoPz-Cu-NH	-10%	at	40 ppm	with	230 ppb	at	5 min	18
NH_3	DC-100 (NiTPz-Cu)	84%	at	40 ppm	with	258 ppb	at	10 min	This work
	NiPz-Cu-NH	18%	at	40 ppm	with	690 ppb	at	5 min	18
	NiPc-O_8-Cu	30%	at	40 ppm	with	160 ppb	at	30 min	5
	NiNPc-Cu	51%	at	40 ppm	with	-	-	-	5
NH_3	DC-104 (CuTPz-Cu)	94%	at	40 ppm	with	63.2 ppb	at	10 min	This work
	CuPz-Cu-NH	26%	at	40 ppm	with	140 ppb	at	5 min	18
NO	DC-103 (CoTPz-Cu)	-123%	at	40 ppm	with	16.8 ppb	at	10 min	This work
	CoPc-O_8-Cu	-170%	at	80 ppm	with	-	-	-	19
NO	DC-100 (NiTPz-Cu)	-58,300	at	40 ppm	with	3.9 ppb	at	10 min	This work
	NiPc-O_8-Cu	-480%	at	80 ppm	with	-	-	-	19
	NiPc-Cu	-150%	at	40 ppm	with	-	-	-	17
	DC-100 (NiTPz-Cu)	-6000%	at	1 ppm	with	3.9 ppb	at	10 min	This work
	NiPc-O_8-Cu	-397%	at	1 ppm	with	0.13 ppb	at	30 min	5
	NiNPc-Cu	-40%	at	1 ppm	with	-	-	-	5
NO	DC-104 (CuTPz-Cu)	-2920%	at	40 ppm	with	0.3 ppb	at	10 min	This work
	CuPc-Cu	-140%	at	40 ppm	with	-	-	-	17

S6. Principal Component Analysis

Principal component analysis (PCA) plots were acquired through Origin Lab 2025 by inputting a normalized matrix of features extracted from the sensing traces. Briefly, we began the PCA analysis by gathering data of the entire 10-minute exposure and 30-minute recovery cycles from three separate devices for each MOF-analyte combination at 1, 5, 10, 20 and 40 ppm concentrations. From those sensing traces, we extracted the following features: i) initial rate of response obtained as the slope of the fitted linear straight line within the first minute of exposure; ii) maximum magnitude of response across the 10 minutes of exposure to gaseous analyte; iii) magnitude of response at 10.8 minutes, or upon 0.8 minutes of exposure to gaseous analyte, as this time shows the best concentration-dependent response prior to saturation; iv) area under the

curve obtained as the integration of the area from minute 10 to minute 20, i.e. during the 10 minutes of exposure to gaseous analytes; and v) % recovery obtained using calculations obtained previously in **Equation S5**.

Across each row containing data features from a single device, the matrix was normalized through the Z-score to center and transform the data into a magnitude and dispersion with an average of zero and a standard deviation of 1, according to the following equation:[20, 21]

Equation S6: $$x_{normalized} = \frac{x_{original} - avg}{stdev}$$

where the normalized data point ($x_{normalized}$) is obtained by subtracting the original data feature ($x_{original}$) by the average (*avg*) of the values across all features from a single device and dividing the difference by the standard deviation (*stdev*) of the latter set of values.

Different combinations of the normalized features allowed us to obtain the plots depicted in **Figure S79** for data from full exposure to all concentrations of analytes. For concrete evidence of differentiation in an environmentally-relevant scenario, we then considered array differentiation at concentrations of analytes that are around or above the Occupational Safety and Health Administration (OSHA) permissible exposure limits (PEL) of each gas (**Figure S80**). For example, for H_2S with an OSHA PEL of 20 ppm, we considered concentrations of 20 and 40 ppm of H_2S in the PCA analysis. Similarly, we considered concentrations of 40 ppm, 5–40 ppm, and 20-40 ppm of NH_3 (PEL = 50 ppm), SO_2 (PEL = 5 ppm), and NO (PEL = 25 ppm), respectively.[22, 23]

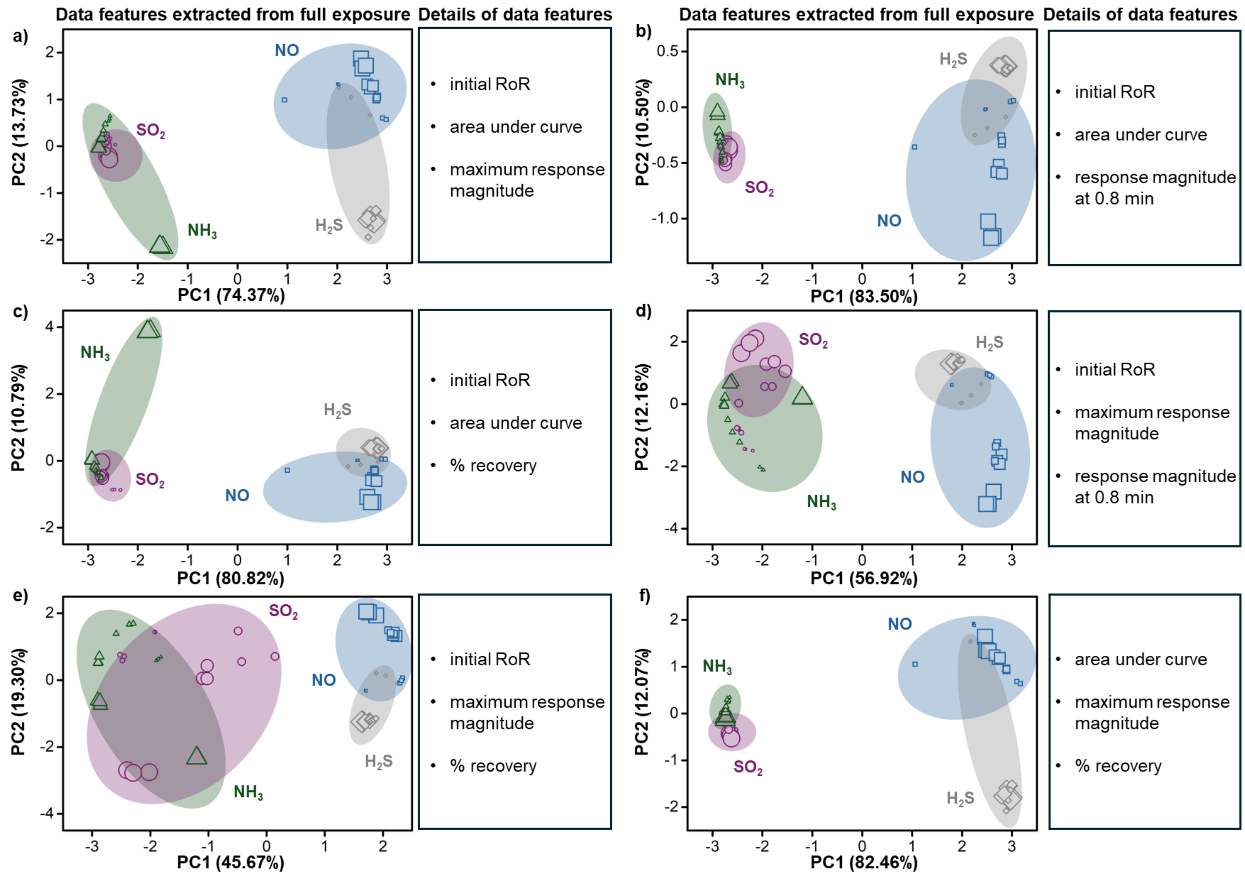

Figure S79. PCA plots upon analysis of the corresponding data features from the full exposure sensing traces of DC-103, DC-100, and DC-104 towards H_2S, NH_3, SO_2, and NO in N_2 at 1, 5, 10, 20, and 40 ppm concentrations of each.

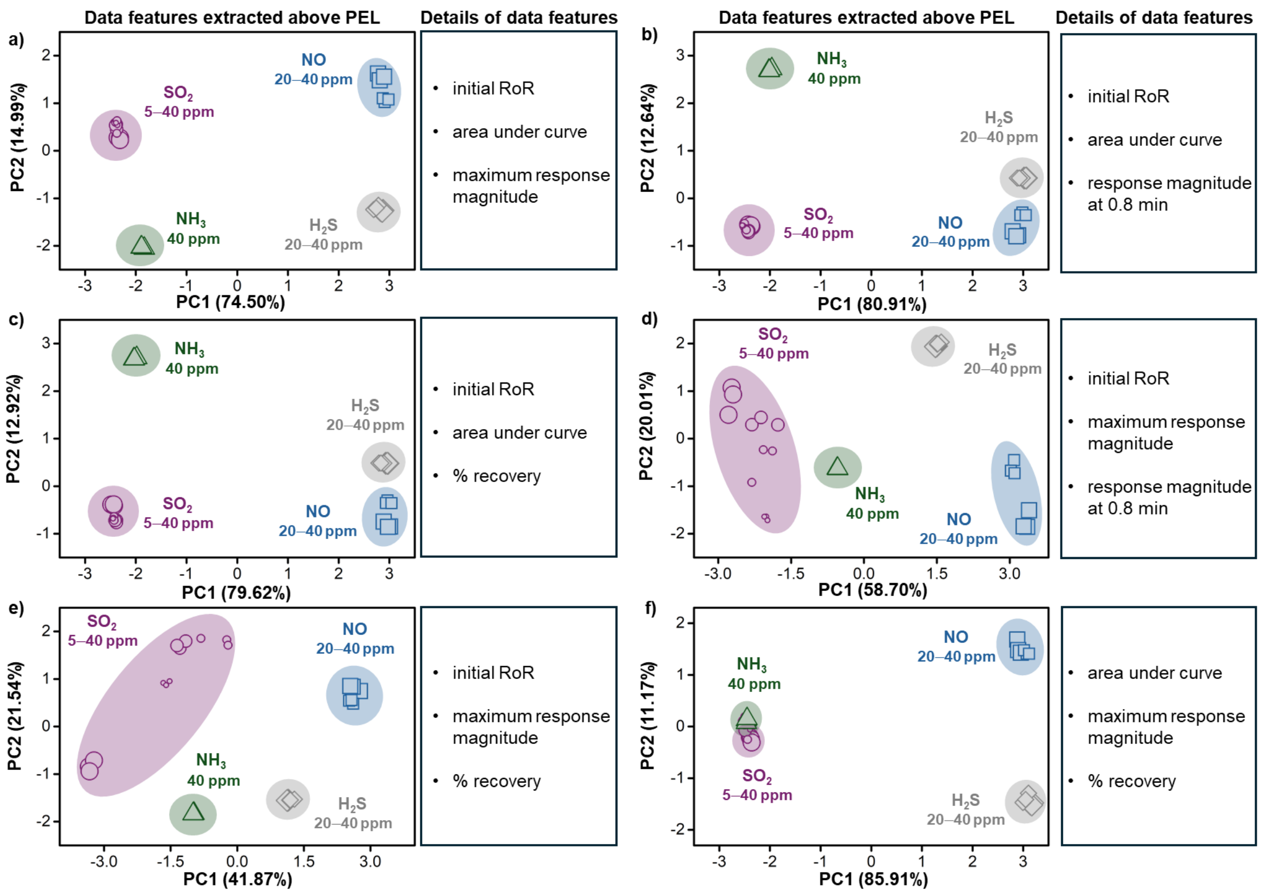

Figure S80. PCA plots upon analysis of the corresponding data features from the full exposure sensing traces of DC-103, DC-100, and DC-104 towards H_2S, NH_3, SO_2, and NO in N_2 at concentrations around or at OSHA PEL limits of each, respectively.

Then, we aimed to study the differentiation within a few seconds of exposure to gas rather than across the entire exposure time. For these shortened exposure times, we performed post-processing PCA for extracted values from the first few seconds of exposure within the full 10-minute exposure. Although PCA is conventionally a post-processing method, previous studies have demonstrated the feasibility of applying PCA and related dimensionality-reduction techniques to real-time data analysis.[24] Additionally, other artificial intelligence (AI)-based approaches for real-time gas differentiation have been reported and could, in principle, be implemented in future sensing platforms with appropriate data acquisition and processing

pipelines.[25] For the purpose of this study, we considered features that are extractable upon that exposure time, hence we focused on: i) initial rate obtained as the slope of the linear fit within the studied exposure time, ii) area under the curve within the studied exposure time, and iii) magnitude of response at the studied exposure time. The obtained results upon exposure to 15 s as well as 6 s of gaseous analytes are shown in **Figure S81**, in addition to ones obtained for similar features across full exposure to 10 minutes for comparison.

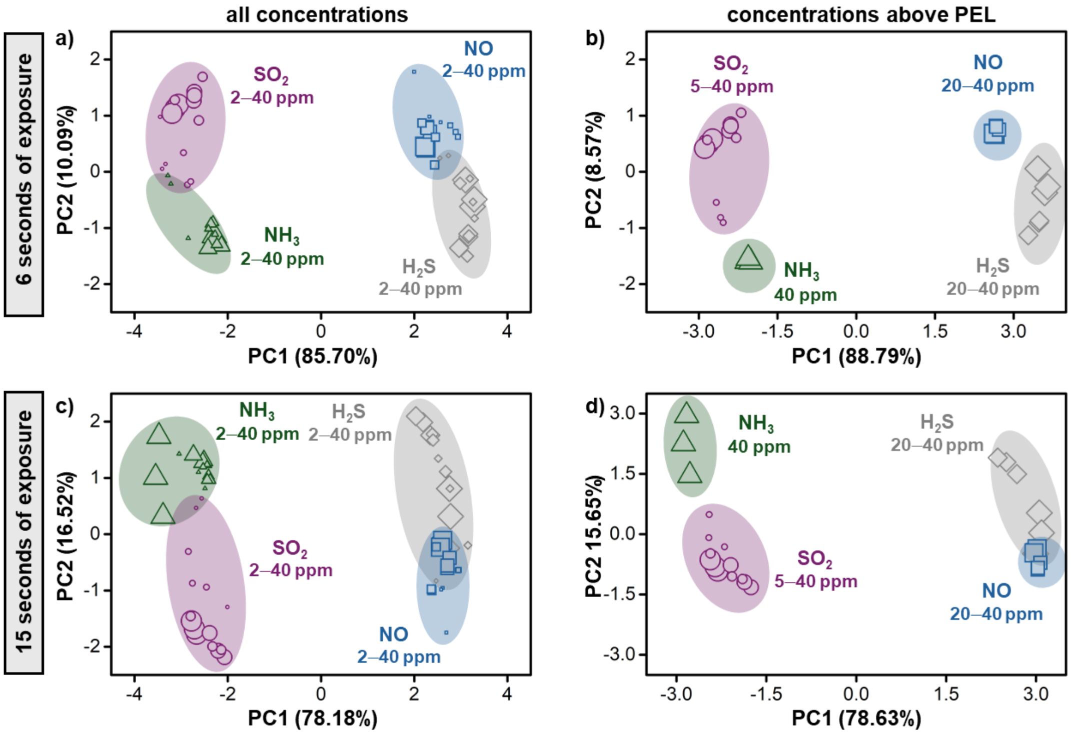

Figure S81. PCA plots upon analysis of the corresponding data features across 2–40 ppm concentrations and concentrations around/above PEL limits of each gas upon exposure to a, b) 6 seconds of exposure and c, d) 15 seconds of exposure to the gases in N_2.

S7. Diffuse Reflectance Infrared Fourier Transform Spectroscopy (DRIFTS)

For data collection, a homogeneous composite of potassium bromide (KBr) with the respective MOF was placed in a sample cup in an air-tight sealed steel chamber with a gas inlet and gas outlet port and with KBr windows to allow IR beam light path. The composite is activated by heating to 110 °C under nitrogen flow for around 1 hour while in the sample cup, followed by its respective cooling. Each MOF/KBr composite is then subjected to a single beam (unsubtracted), which was used as the initial spectrum, while accounting for a background spectrum of dry KBr. Then, difference spectra (Kubelka-Munk) are collected by subtracting spectra collected during exposure from the initial spectrum. Each of these spectra were collected with 32 scans from 400 to 4000 cm^{-1}. The reported spectra are at 0, 2, 4, 6, 8, and 10 min of exposure to gaseous analyte, followed by recovery spectra while purging with nitrogen gas at 1, 2, 4, 6, 8, and 10 min of recovery. Due to low signal of gases at the concentrations at which sensing experiments were performed, we opted to use higher concentrations of gases for the DRIFTS experiments, by which 0.1 % of H_2S and 1% of NH_3, SO_2, and NO in nitrogen were used. For NO experiments, NO gas was passed through a dry tube containing soda lime (activated overnight at 80 °C under nitrogen) was utilized to eliminate/decrease the presence of nitrogen dioxide impurities from the NO gas tank, as they can lead to the formation of undesired potassium nitrate that overlaps with the IR peaks under study. In DRIFTS, we consistently observed the appearance of water-related bands during gas exposure, identified at around 3500 and 1650 cm^{-1}, corresponding to the O–H stretching and bending vibrations of water, respectively (**Figures S82-S93**).[26]

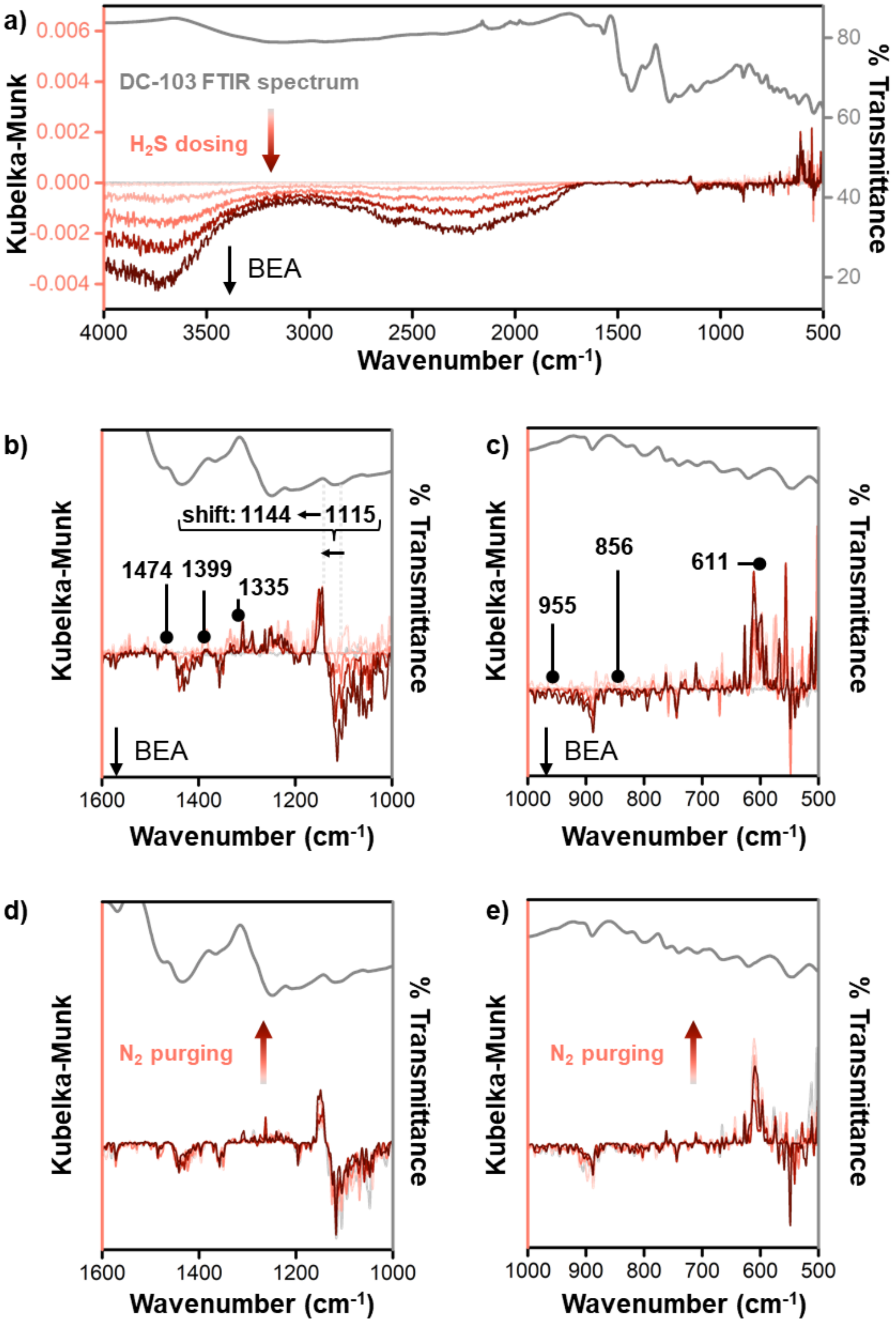

Figure S82. a), b) and c) DRIFTS spectra of DC-103 towards 1000 ppm of H_2S at 0, 2, 4, 6, 8, and 10 minutes of exposure; d), e), and f) subsequent DRIFTS spectra of DC-103 upon purging with N_2 gas after 1, 2, 4, 6, 8, and 10 minutes of recovery.

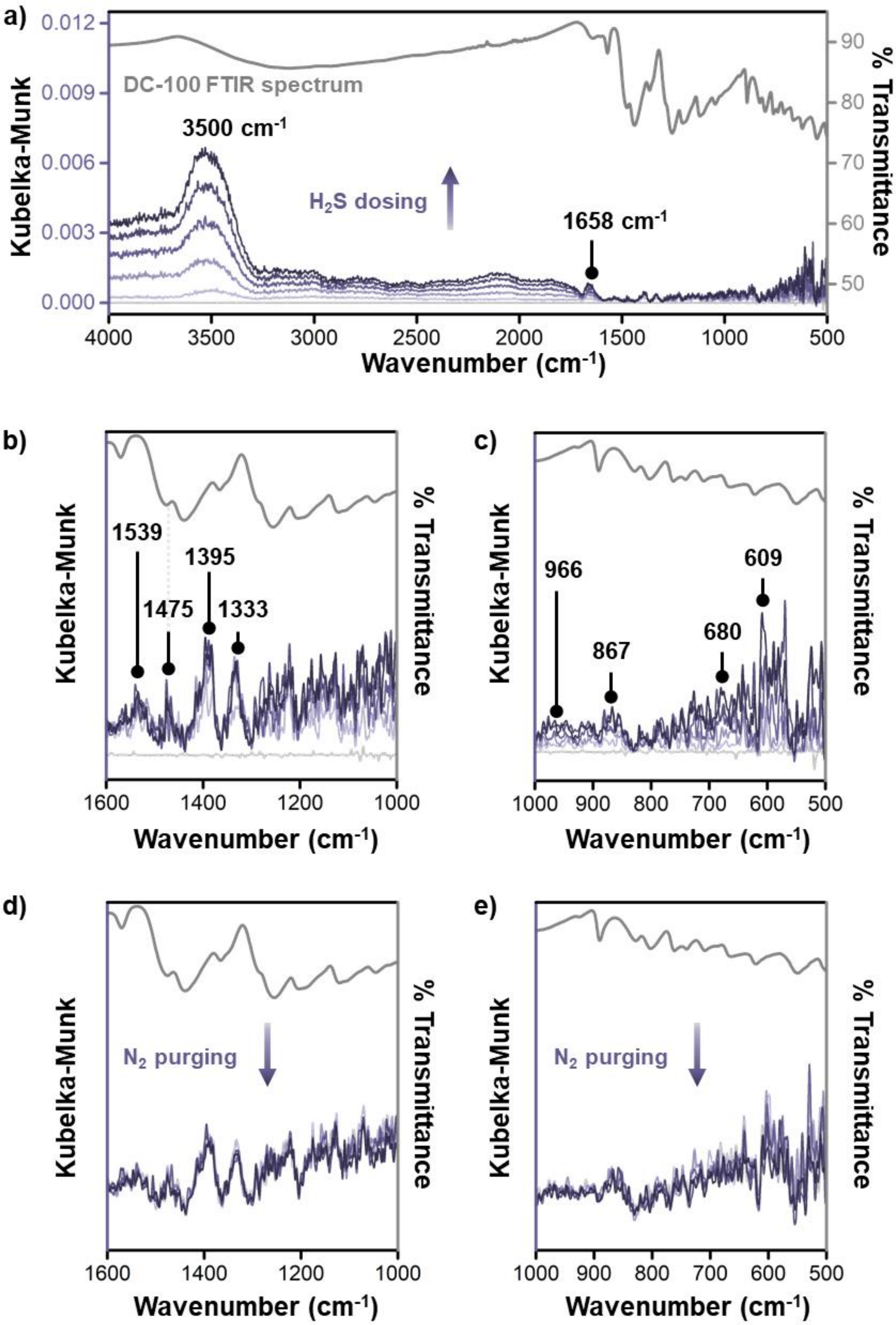

Figure S83. a), b) and c) DRIFTS spectra of DC-100 towards 1000 ppm of H_2S at 0, 2, 4, 6, 8, and 10 minutes of exposure; d), e), and f) subsequent DRIFTS spectra of DC-100 upon purging with N_2 gas after 1, 2, 4, 6, 8, and 10 minutes of recovery.

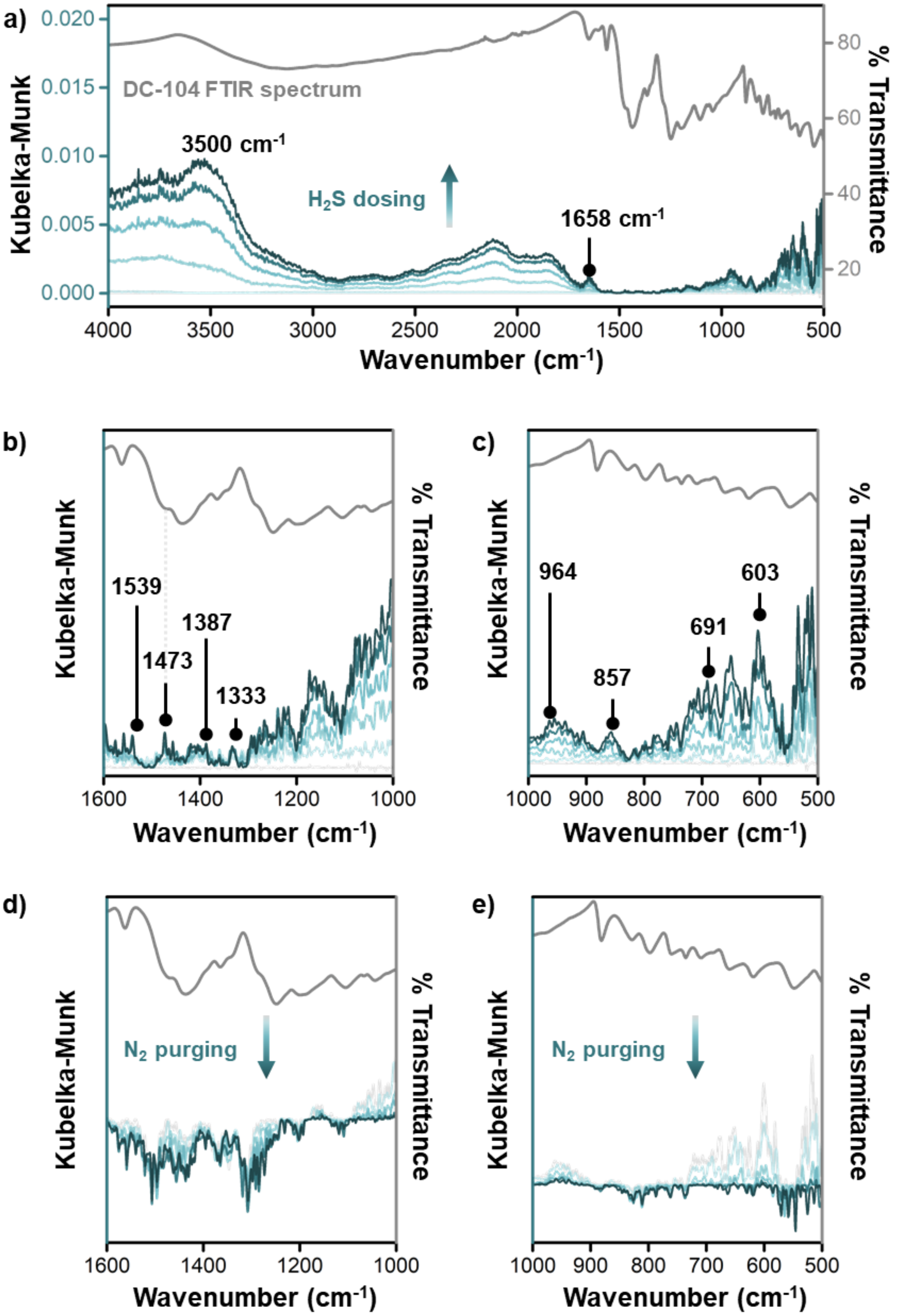

Figure S84. a), b) and c) DRIFTS spectra of DC-104 towards 1000 ppm of H_2S at 0, 2, 4, 6, 8, and 10 minutes of exposure; d), e), and f) subsequent DRIFTS spectra of DC-104 upon purging with N_2 gas after 1, 2, 4, 6, 8, and 10 minutes of recovery.

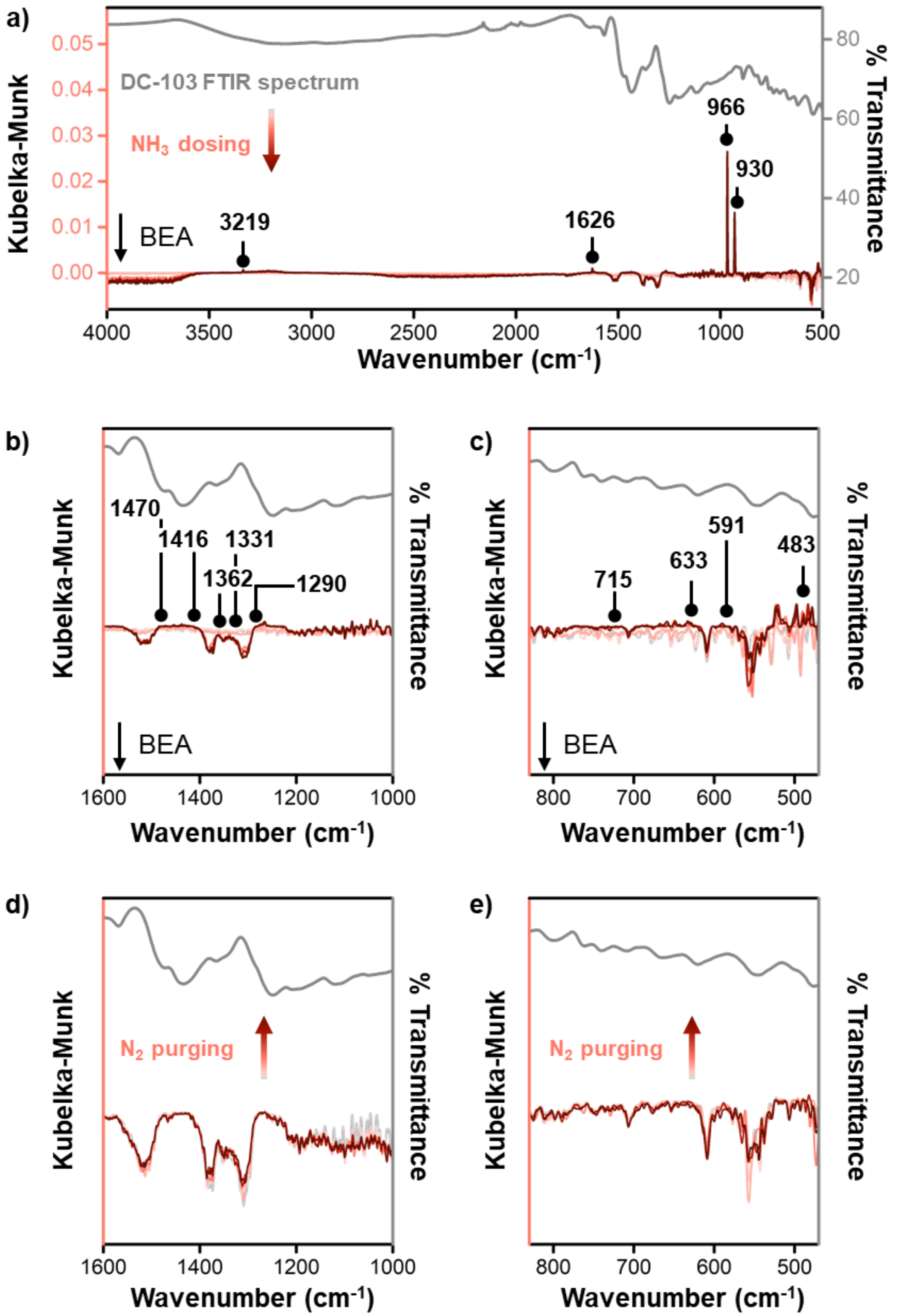

Figure S85. a), b) and c) DRIFTS spectra of DC-103 towards 1% of NH_3 at 0, 2, 4, 6, 8, and 10 minutes of exposure; d), e), and f) subsequent DRIFTS spectra of DC-103 upon purging with N_2 gas after 1, 2, 4, 6, 8, and 10 minutes of recovery.

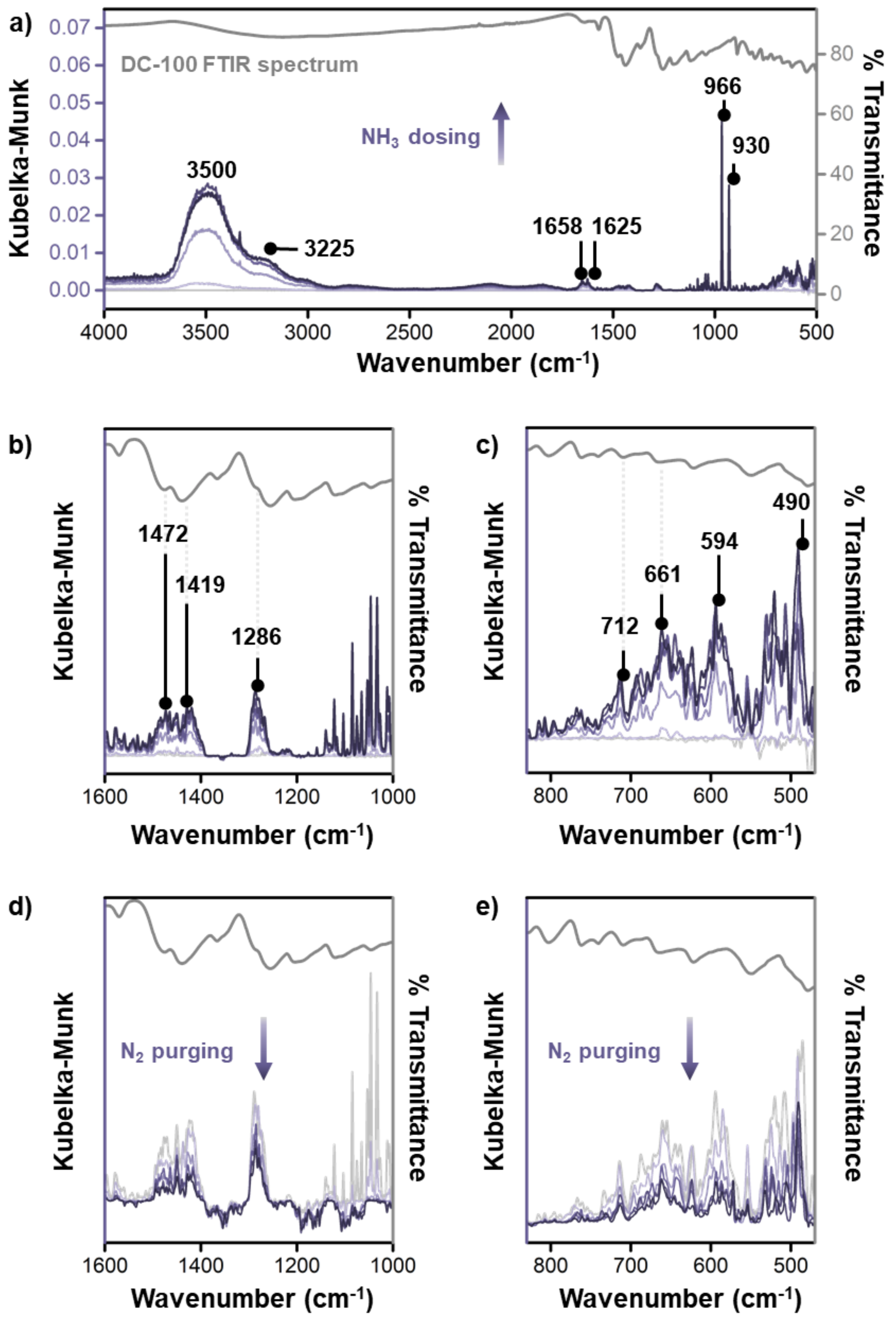

Figure S86. a), b) and c) DRIFTS spectra of DC-100 towards 1% NH_3 at 0, 2, 4, 6, 8, and 10 minutes of exposure; d), e), and f) subsequent DRIFTS spectra of DC-100 upon purging with N_2 gas after 1, 2, 4, 6, 8, and 10 minutes of recovery.

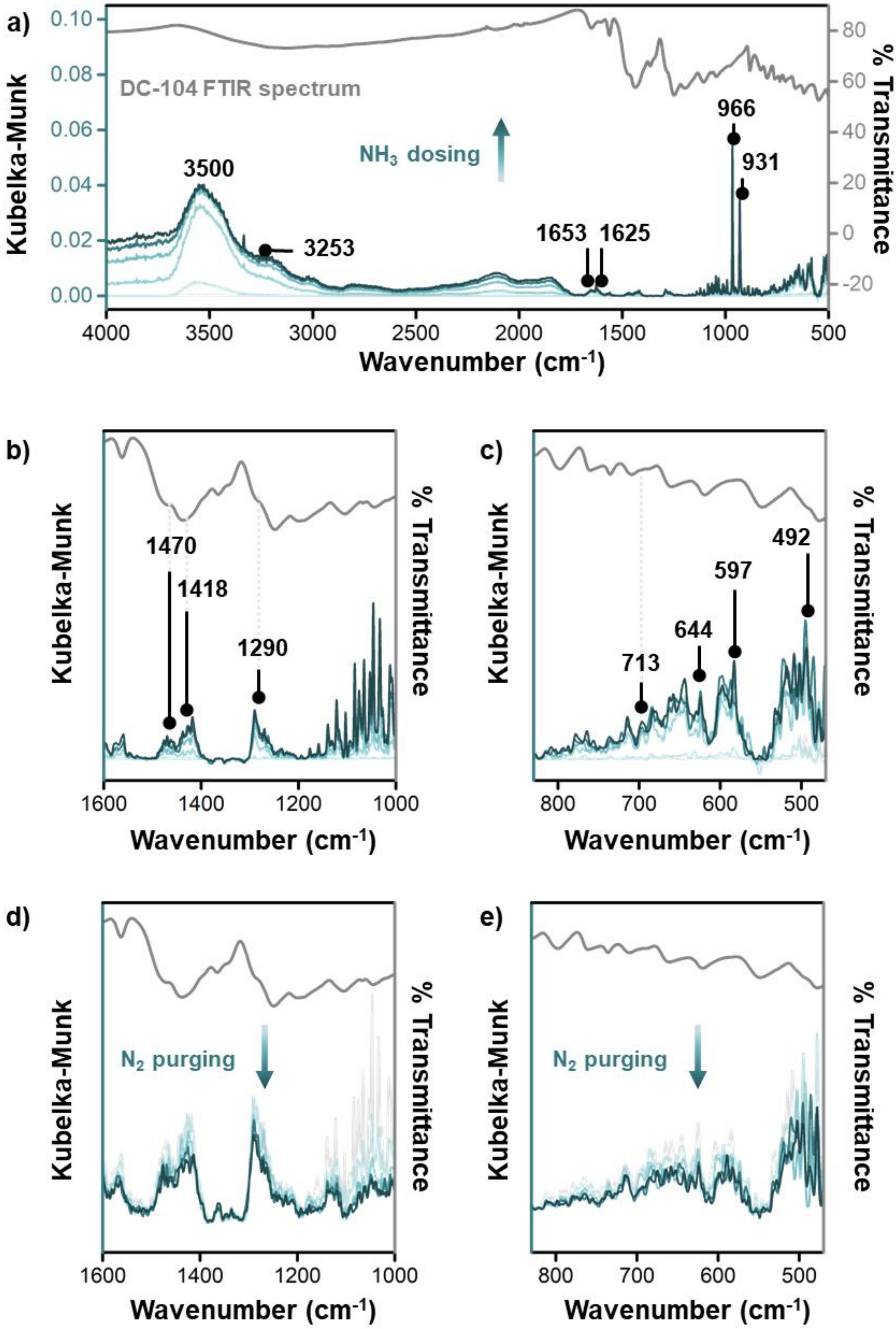

Figure S87. a), b) and c) DRIFTS spectra of DC-104 towards 1% of NH_3 at 0, 2, 4, 6, 8, and 10 minutes of exposure; d), e), and f) subsequent DRIFTS spectra of DC-104 upon purging with N_2 gas after 1, 2, 4, 6, 8, and 10 minutes of recovery.

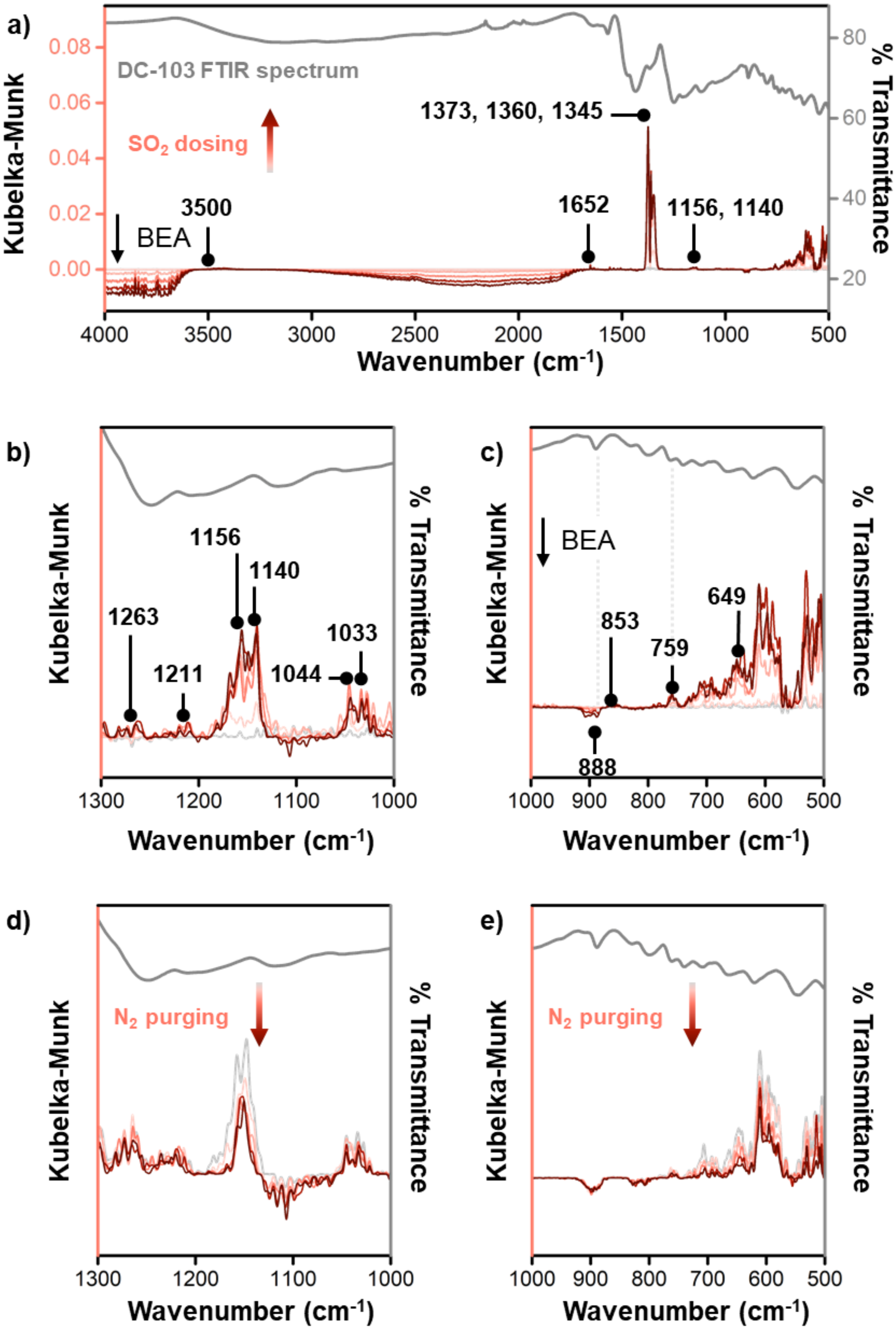

Figure S88. a), b) and c) DRIFTS spectra of DC-103 towards 1% of SO_2 at 0, 2, 4, 6, 8, and 10 minutes of exposure; d), e), and f) subsequent DRIFTS spectra of DC-103 upon purging with N_2 gas after 1, 2, 4, 6, 8, and 10 minutes of recovery.

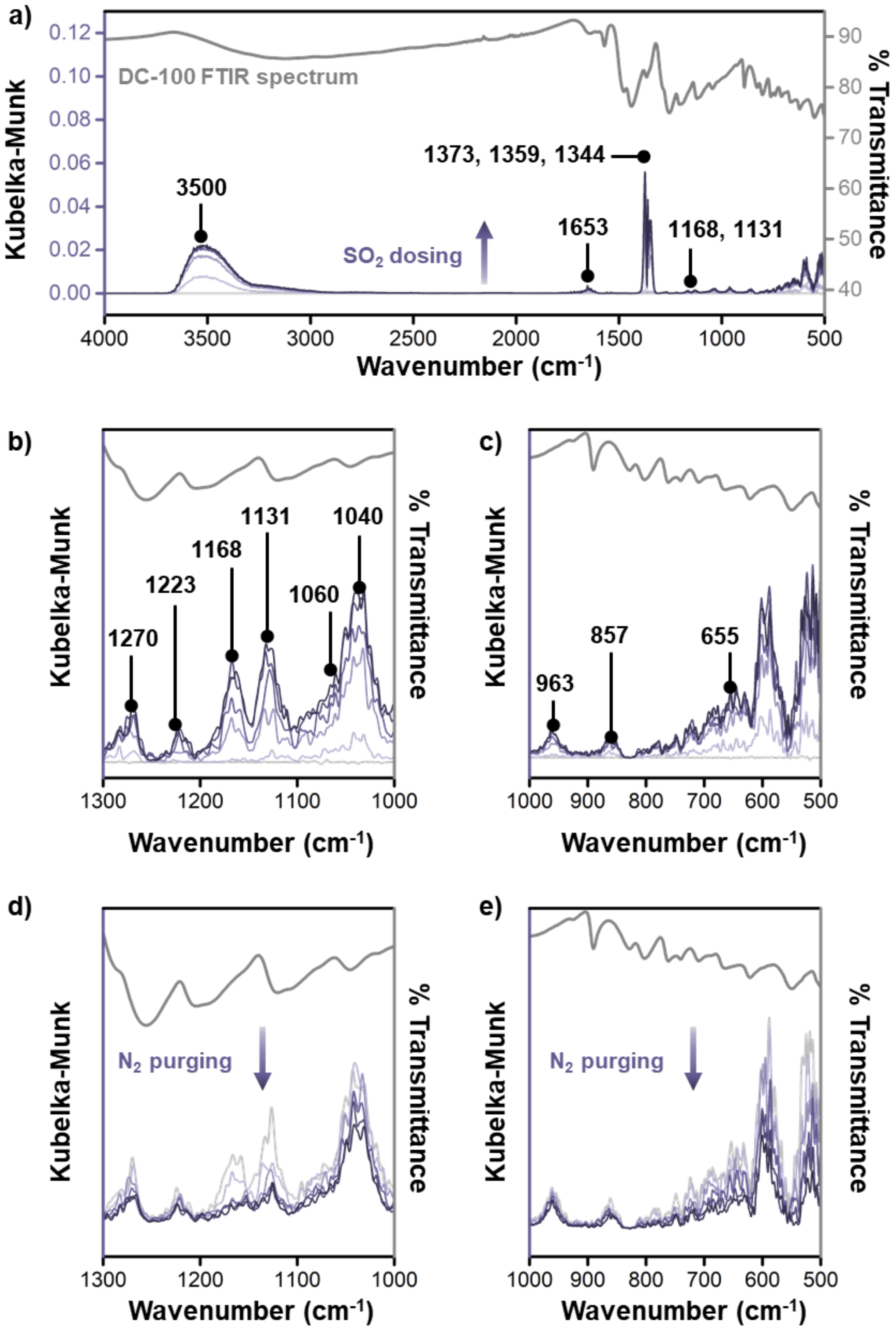

Figure S89. a), b) and c) DRIFTS spectra of DC-100 towards 1% of SO_2 at 0, 2, 4, 6, 8, and 10 minutes of exposure; d), e), and f) subsequent DRIFTS spectra of DC-100 upon purging with N_2 gas after 1, 2, 4, 6, 8, and 10 minutes of recovery.

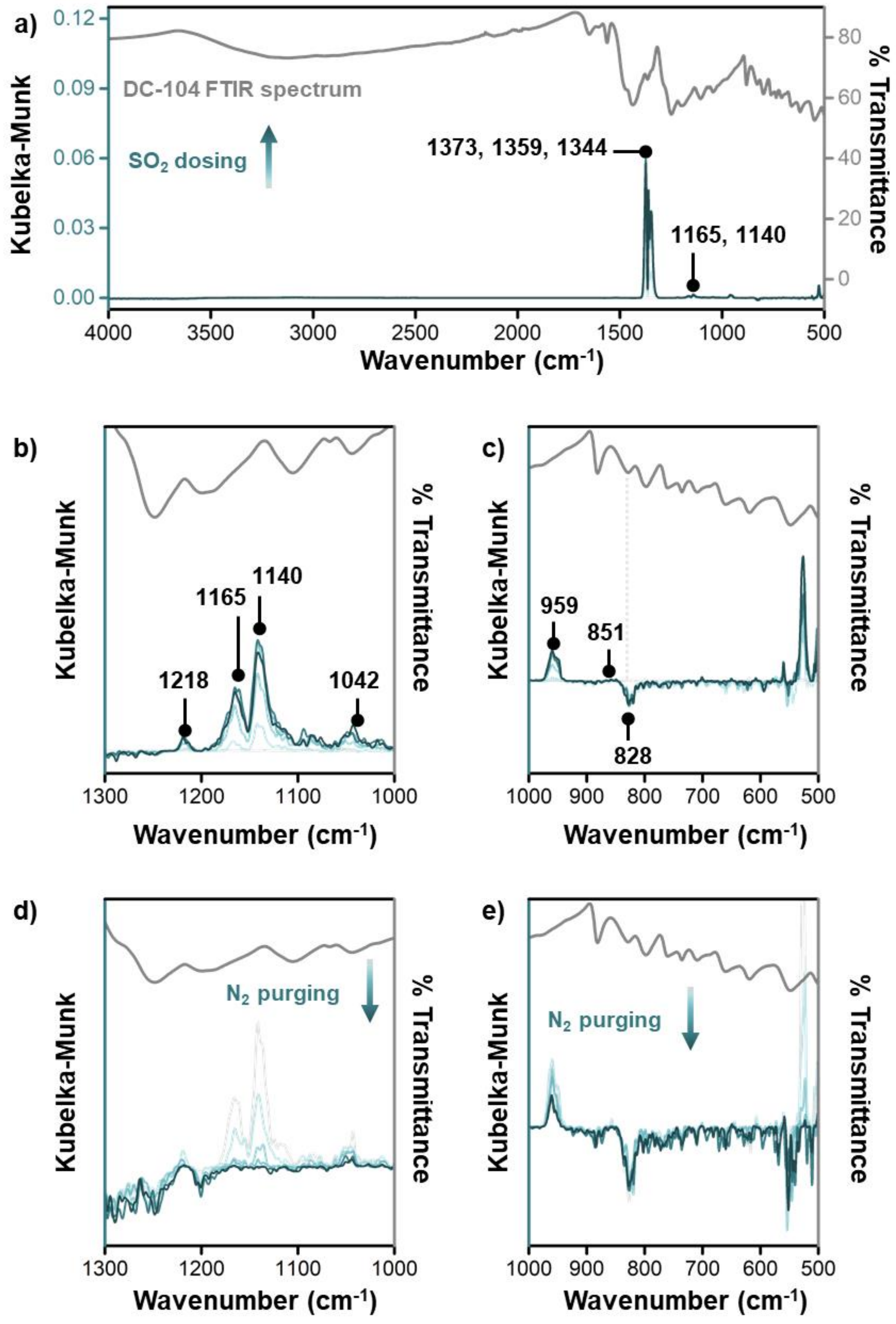

Figure S90. a), b) and c) DRIFTS spectra of DC-104 towards 1% of SO_2 at 0, 2, 4, 6, 8, and 10 minutes of exposure; d), e), and f) subsequent DRIFTS spectra of DC-104 upon purging with N_2 gas after 1, 2, 4, 6, 8, and 10 minutes of recovery.

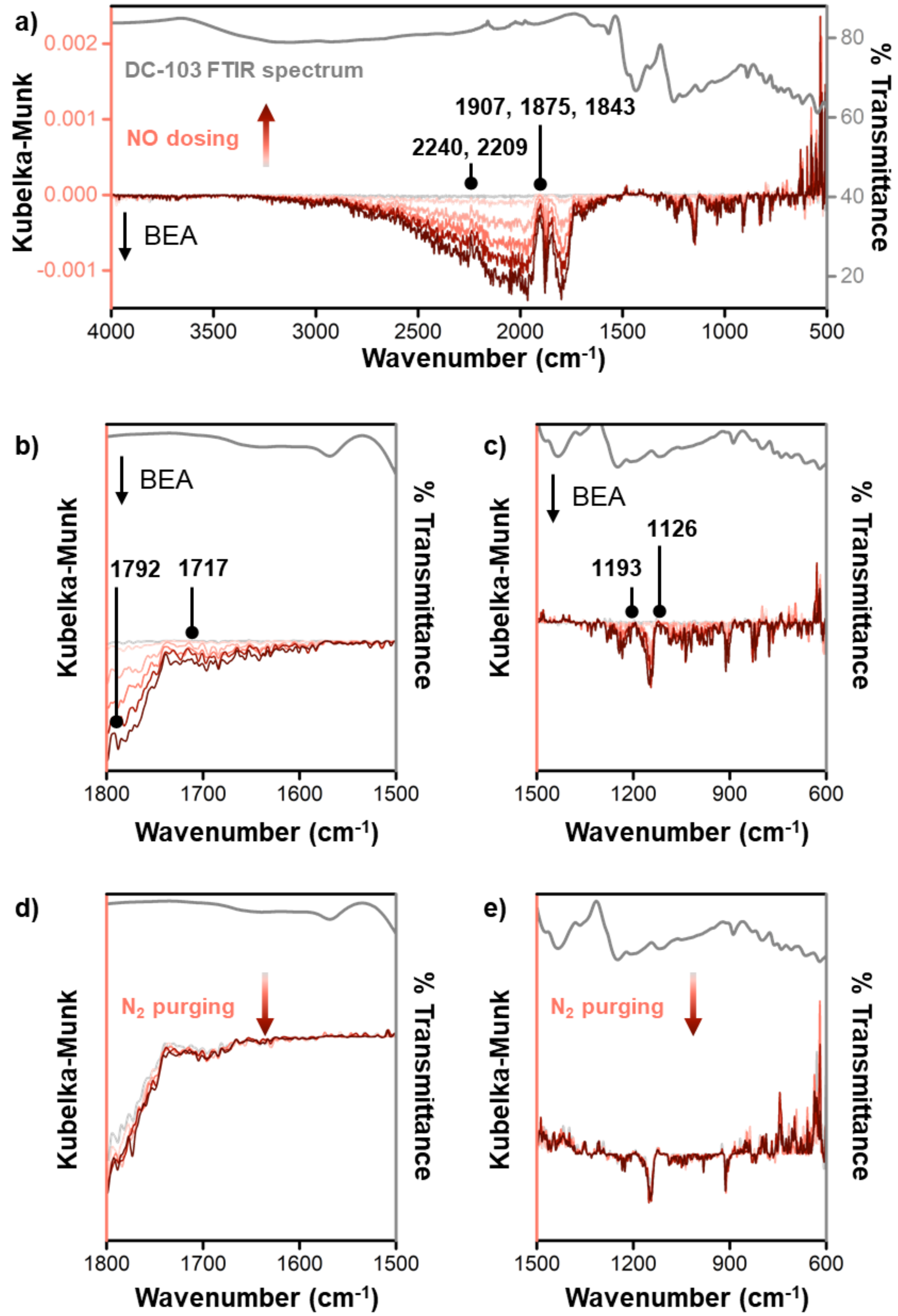

Figure S91. a), b) and c) DRIFTS spectra of DC-103 towards 1% of NO at 0, 2, 4, 6, 8, and 10 minutes of exposure; d), e), and f) subsequent DRIFTS spectra of DC-103 upon purging with N_2 gas after 1, 2, 4, 6, 8, and 10 minutes of recovery.

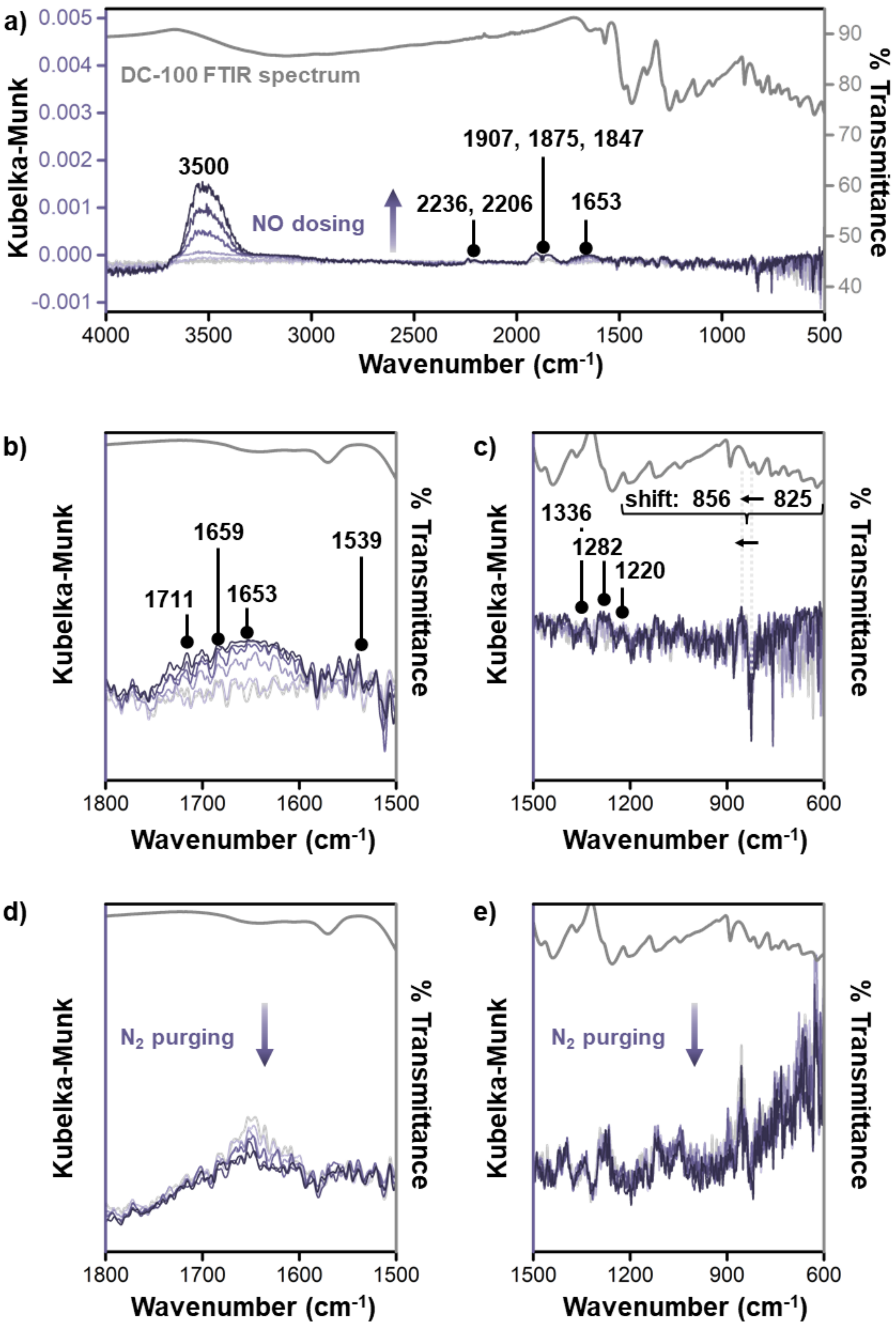

Figure S92. a), b) and c) DRIFTS spectra of DC-100 towards 1% of NO at 0, 2, 4, 6, 8, and 10 minutes of exposure; d), e), and f) subsequent DRIFTS spectra of DC-100 upon purging with N_2 gas after 1, 2, 4, 6, 8, and 10 minutes of recovery.

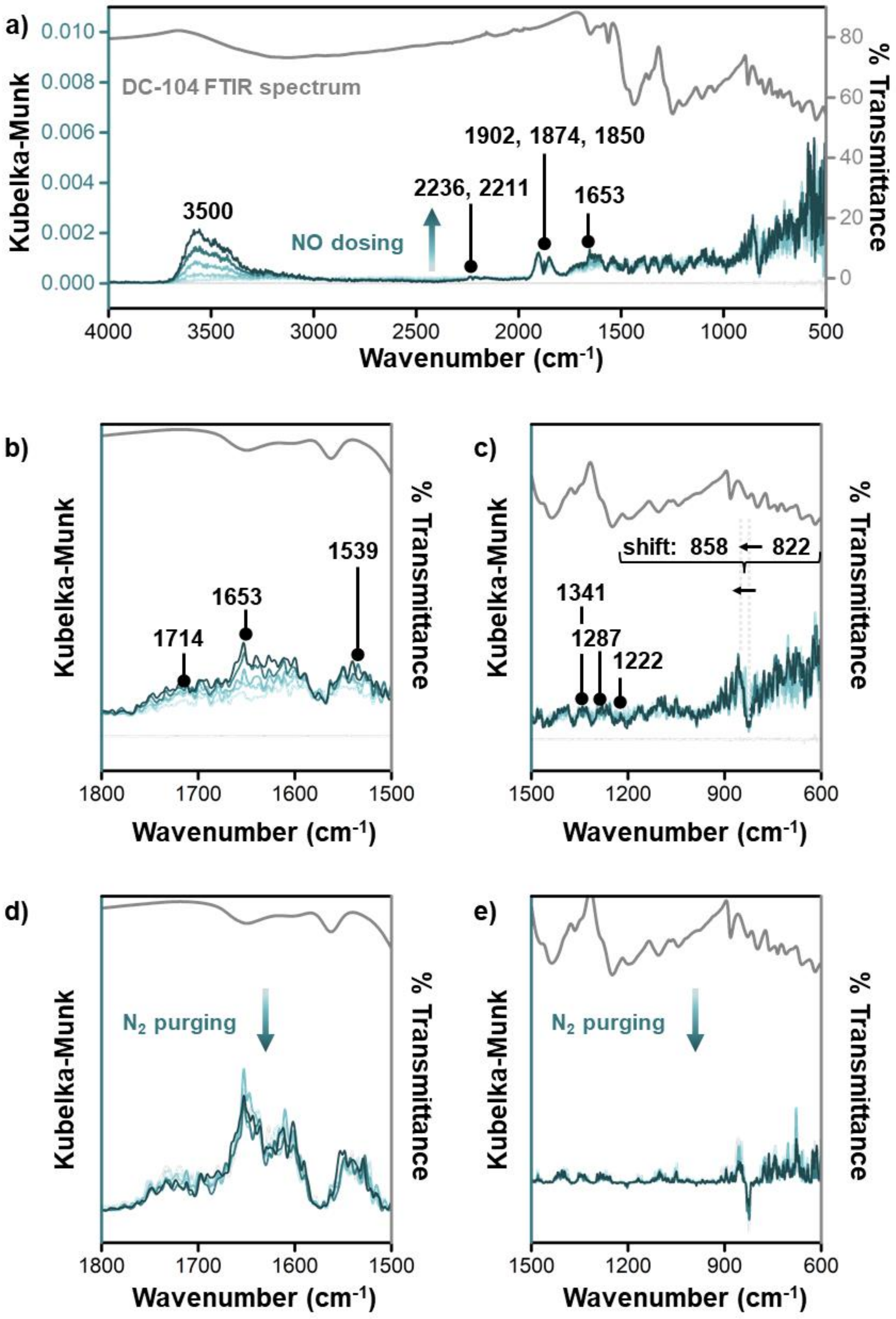

Figure S93. a), b) and c) DRIFTS spectra of DC-104 towards 1% of NO at 0, 2, 4, 6, 8, and 10 minutes of exposure; d), e), and f) subsequent DRIFTS spectra of DC-104 upon purging with N_2 gas after 1, 2, 4, 6, 8, and 10 minutes of recovery.

S8. Characterization after exposure to gases

For XPS and EPR spectra collected after exposure, the MOF powders were placed in a sealed container with an inlet of the gas analyte (at 1% concentration in nitrogen) and an outlet for 2 hours before analysis.

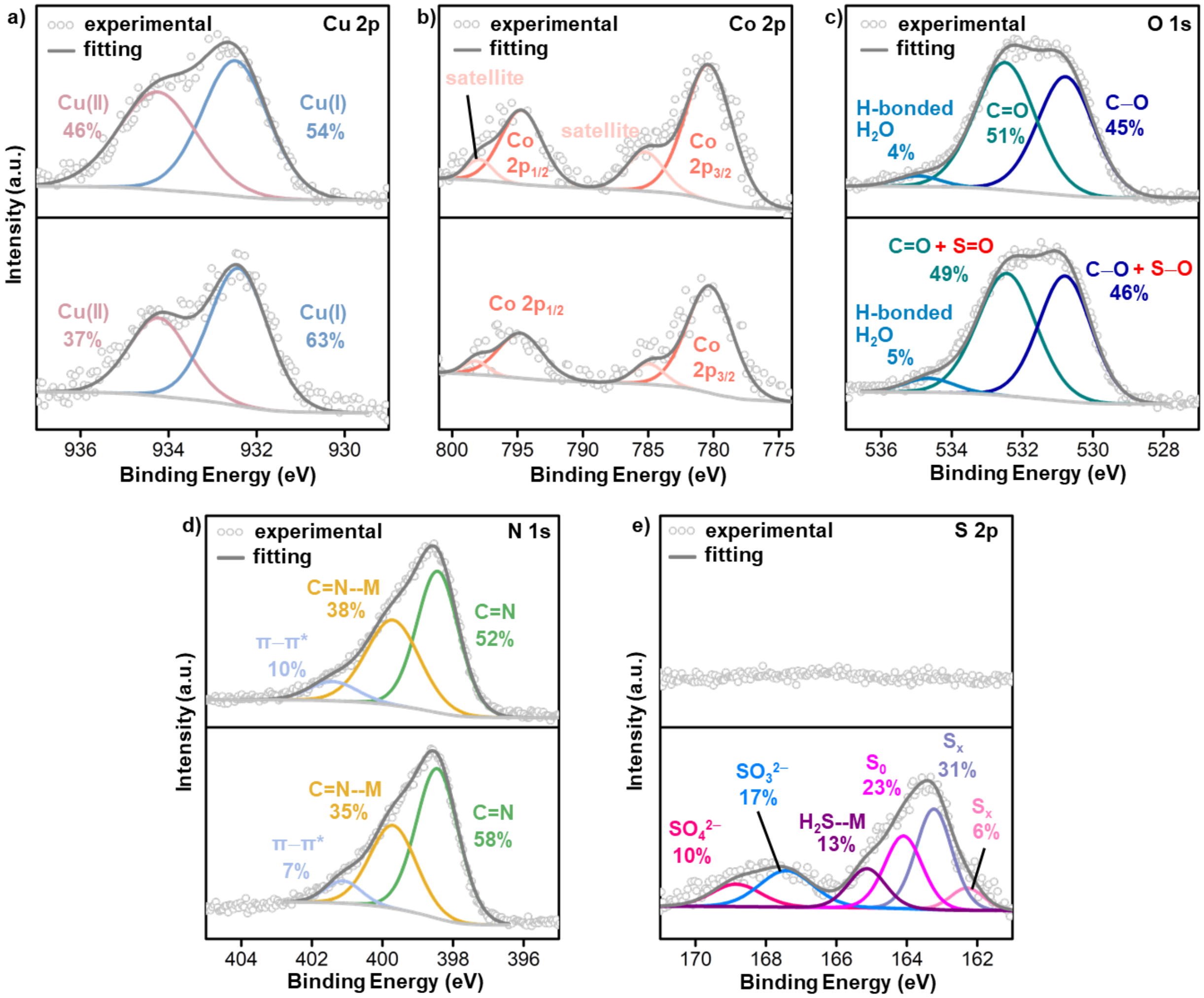

Figure S94. High-resolution XPS spectra of DC-103 after 2 hours of exposure to 1% H_2S in N_2 at the binding energies of a) Cu 2p, b) Co 2p, c) O 1s, d) N 1s, and e) S 2p.

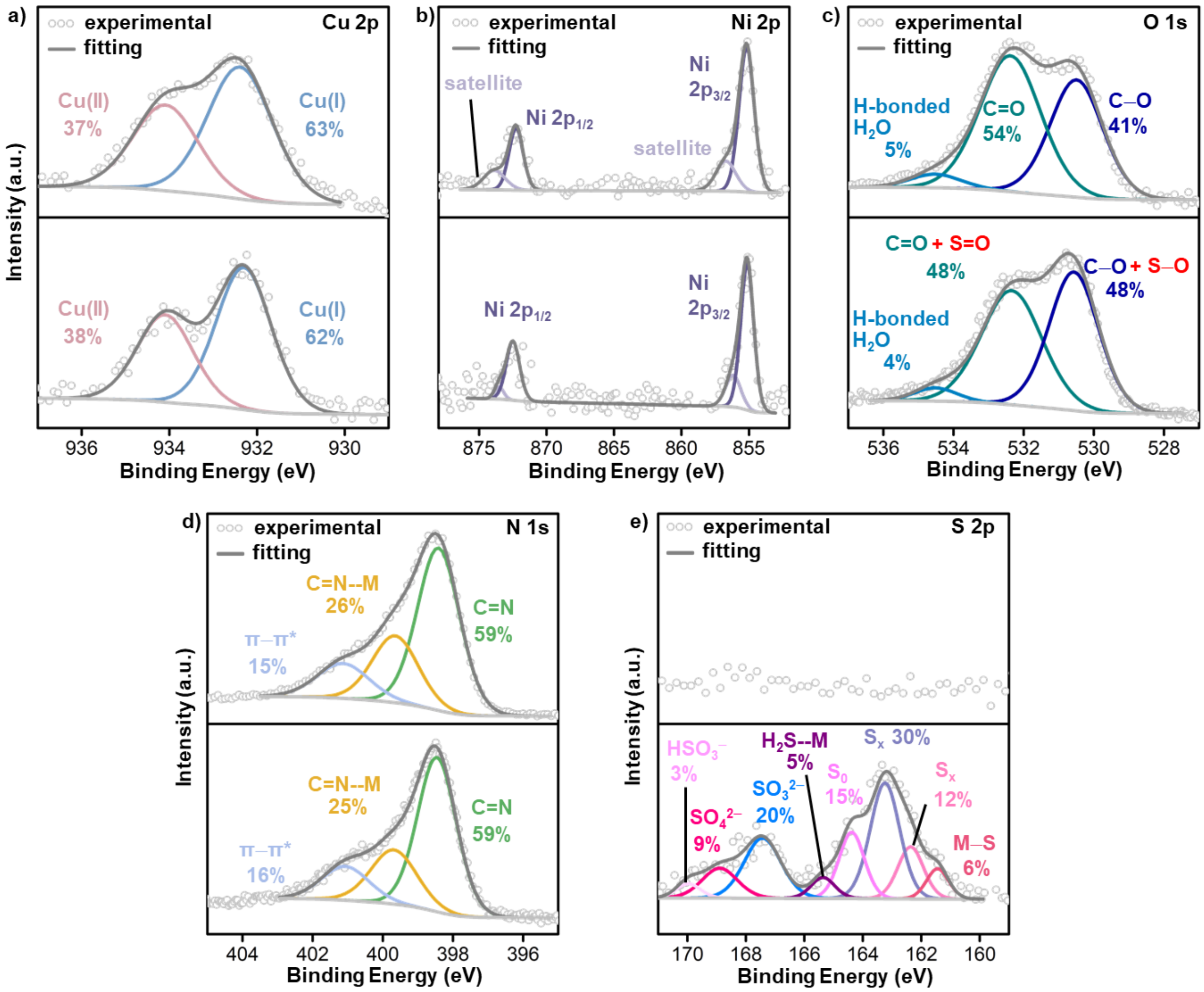

Figure S95. High-resolution XPS spectra of DC-100 after 2 hours of exposure to 1% H_2S in N_2 at the binding energies of a) Cu 2p, b) Ni 2p, c) O 1s, d) N 1s, and e) S 2p.

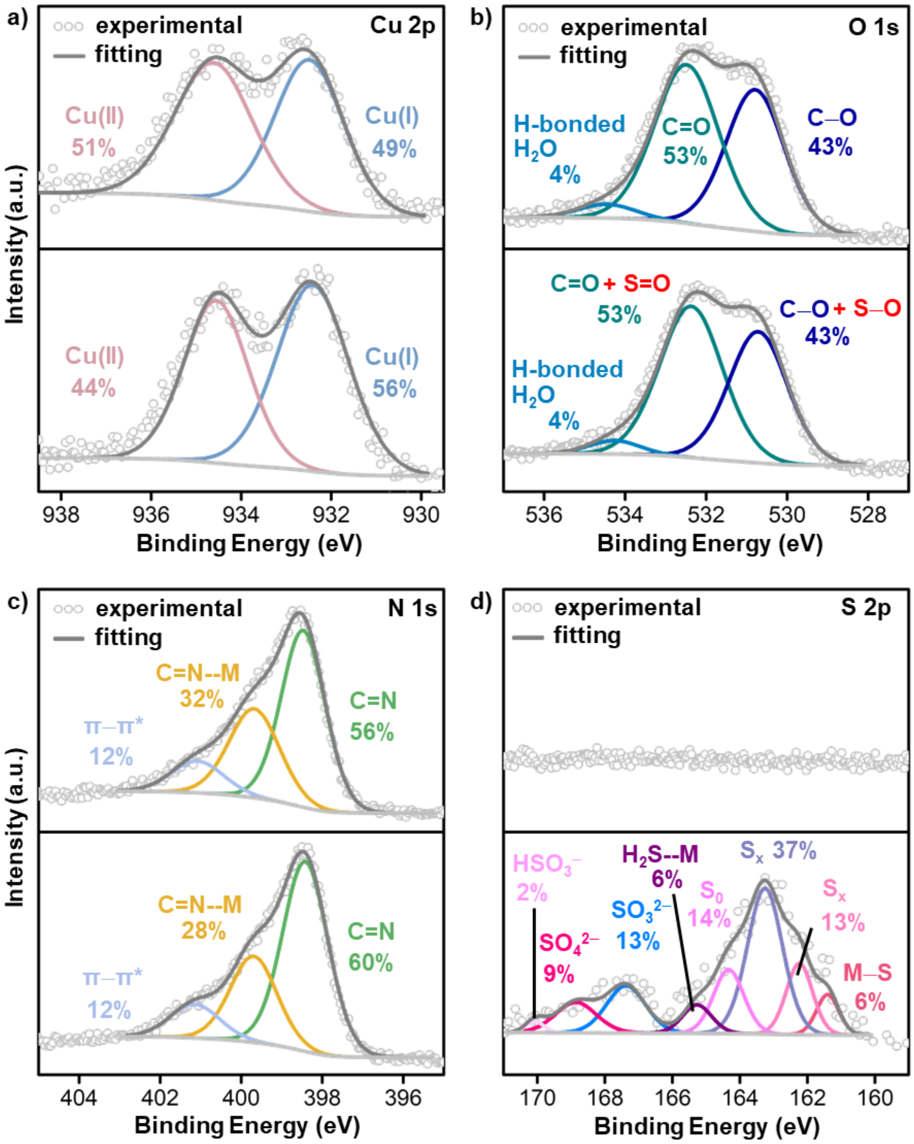

Figure S96. High-resolution XPS spectra of DC-104 after 2 hours of exposure to 1% H_2S in N_2 at the binding energies of a) Cu 2p, b) O 1s, c) N 1s, and d) S 2p.

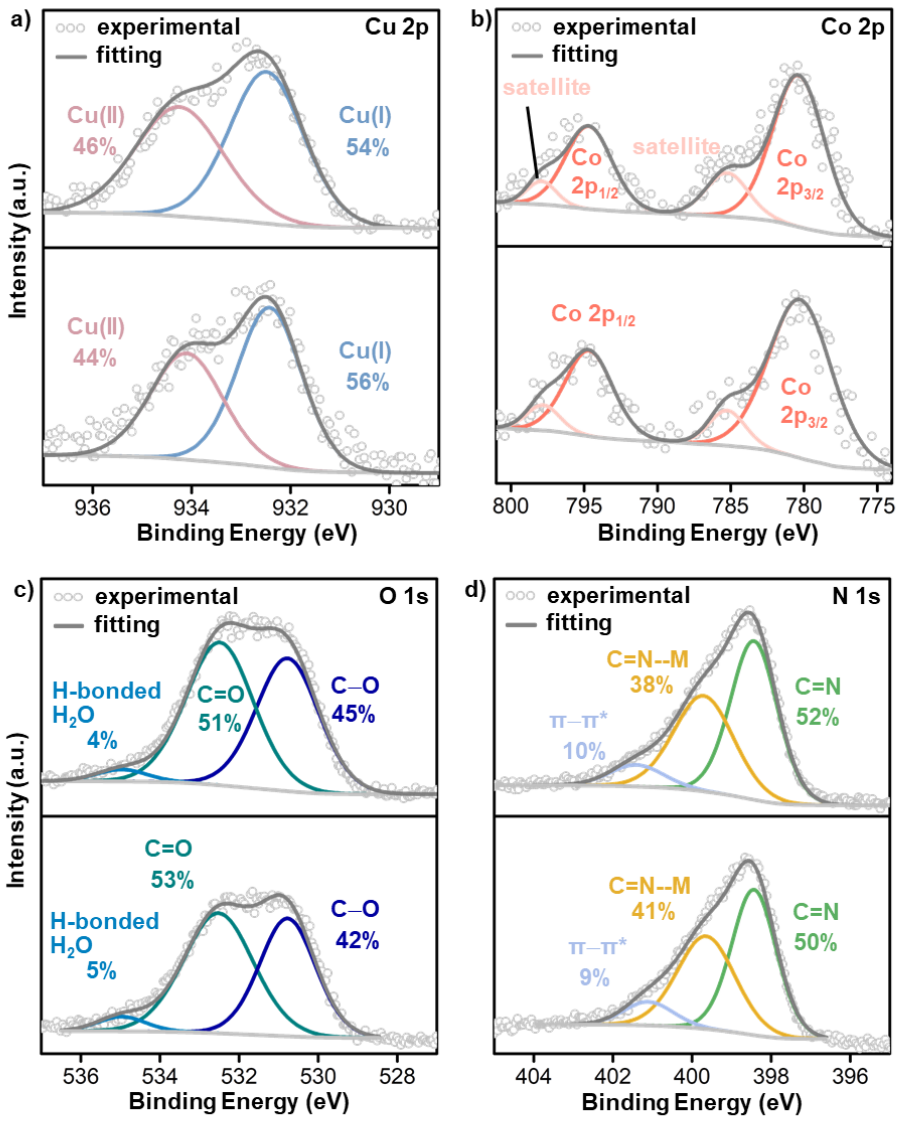

Figure S97. High-resolution XPS spectra of DC-103 after 2 hours of exposure to 1% NH_3 in N_2 at the binding energies of a) Cu 2p, b) Co 2p, c) O 1s, and d) N 1s.

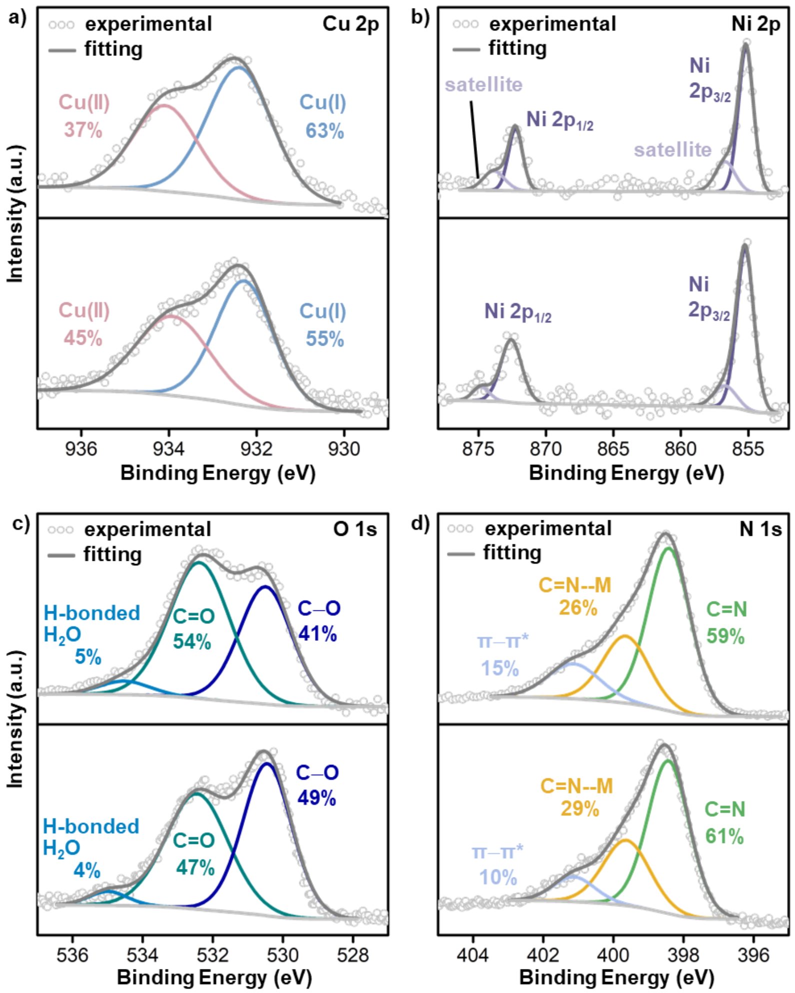

Figure S98. High-resolution XPS spectra of DC-100 after 2 hours of exposure to 1% NH_3 in N_2 at the binding energies of a) Cu 2p, b) Ni 2p, c) O 1s, and d) N 1s.

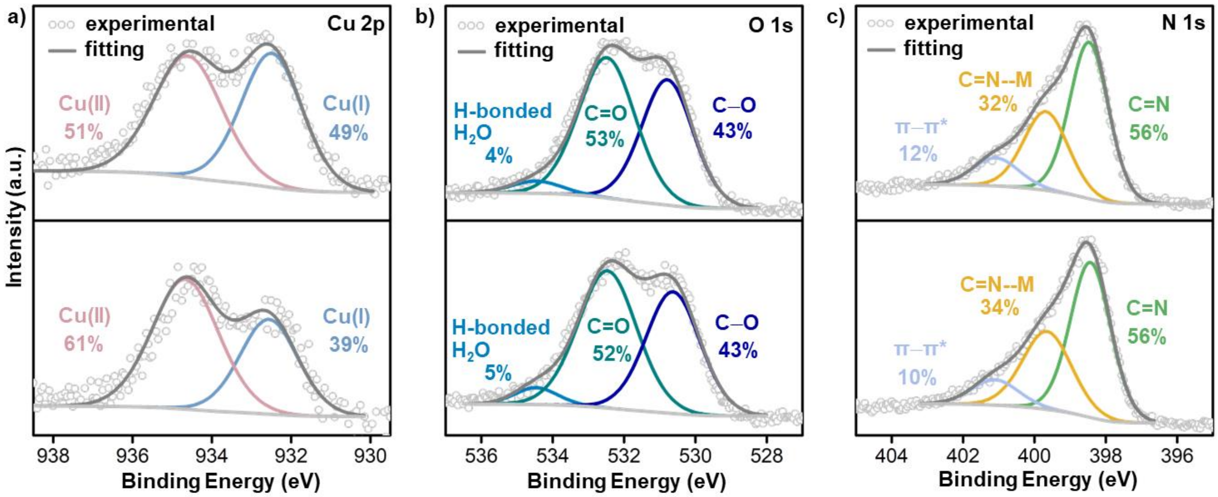

Figure S99. High-resolution XPS spectra of DC-104 after 2 hours of exposure to 1% NH_3 in N_2 at the binding energies of a) Cu 2p, b) O 1s, and c) N 1s.

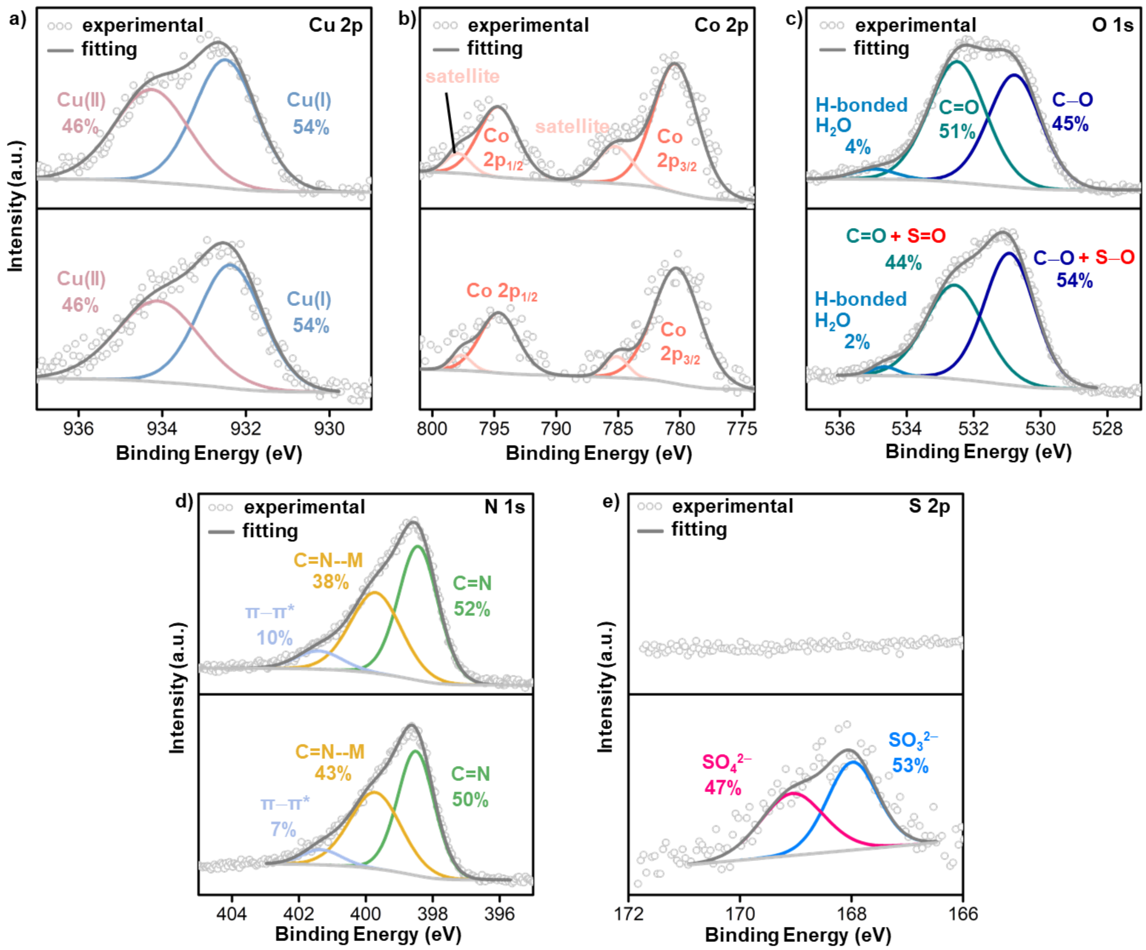

Figure S100. High-resolution XPS spectra of DC-103 after 2 hours of exposure to 1% SO_2 in N_2 at the binding energies of a) Cu 2p, b) Co 2p, c) O 1s, d) N 1s, and e) S 2p.

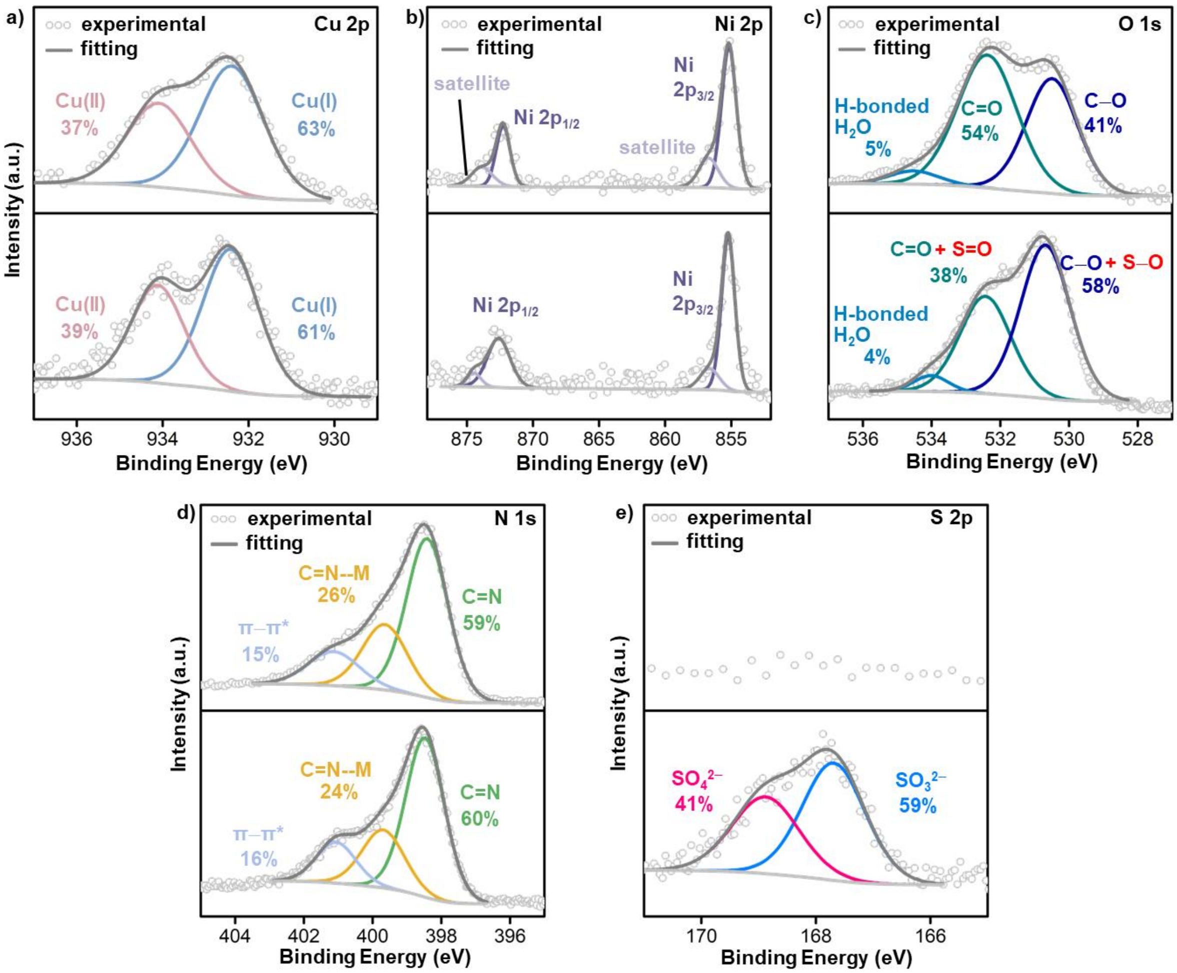

Figure S101. High-resolution XPS spectra of DC-100 after 2 hours of exposure to 1% SO_2 in N_2 at the binding energies of a) Cu 2p, b) Ni 2p, c) O 1s, d) N 1s, and e) S 2p.

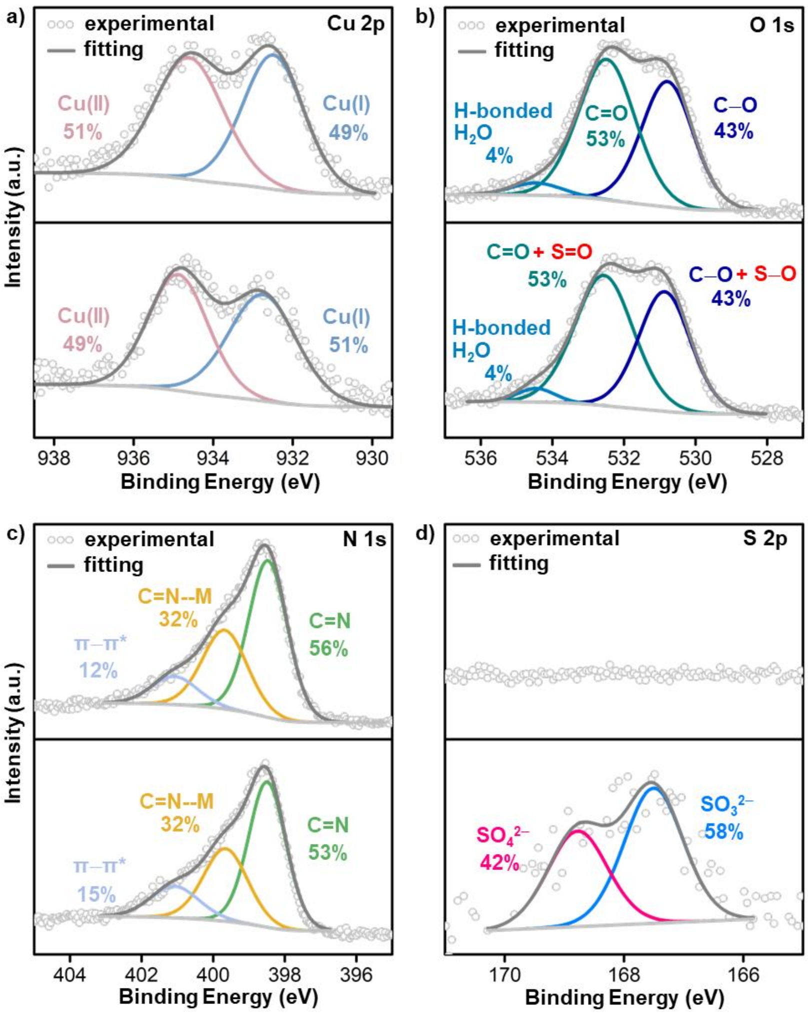

Figure S102. High-resolution XPS spectra of DC-104 after 2 hours of exposure to 1% SO_2 in N_2 at the binding energies of a) Cu 2p, b) O 1s, c) N 1s, and d) S 2p.

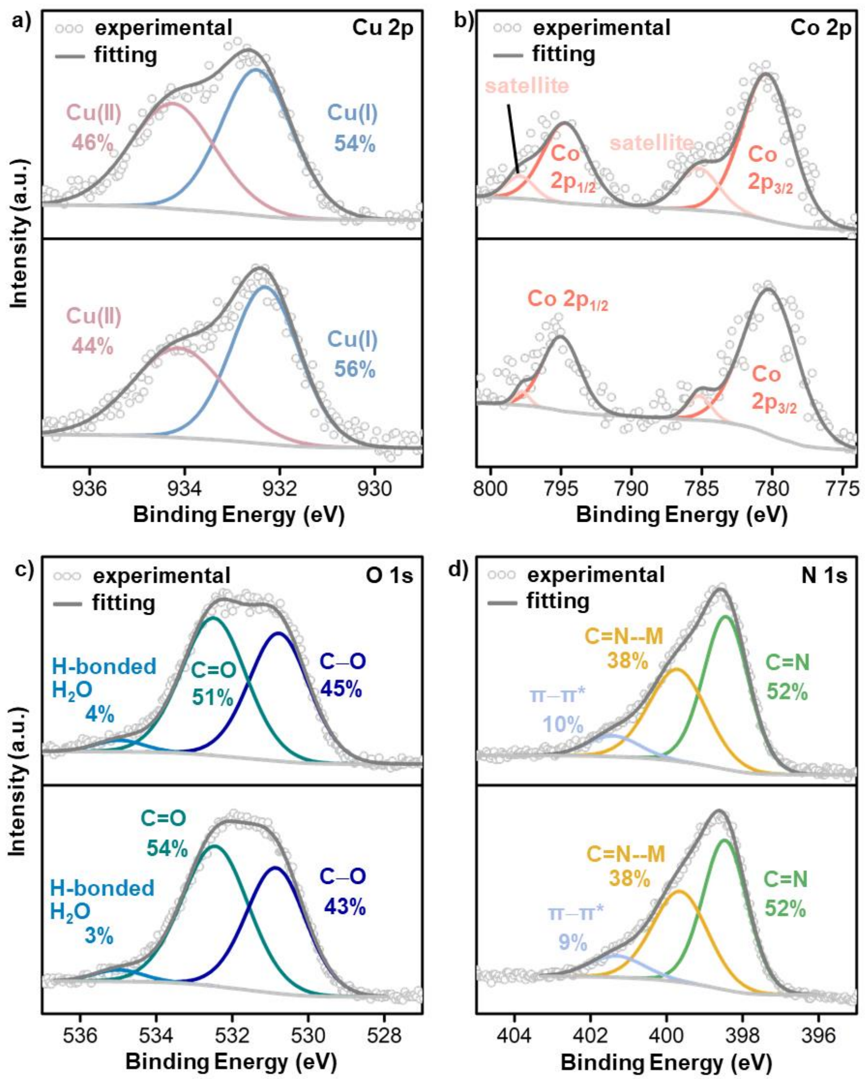

Figure S103. High-resolution XPS spectra of DC-103 after 2 hours of exposure to 1% NO in N_2 at the binding energies of a) Cu 2p, b) Co 2p, c) O 1s, and d) N 1s.

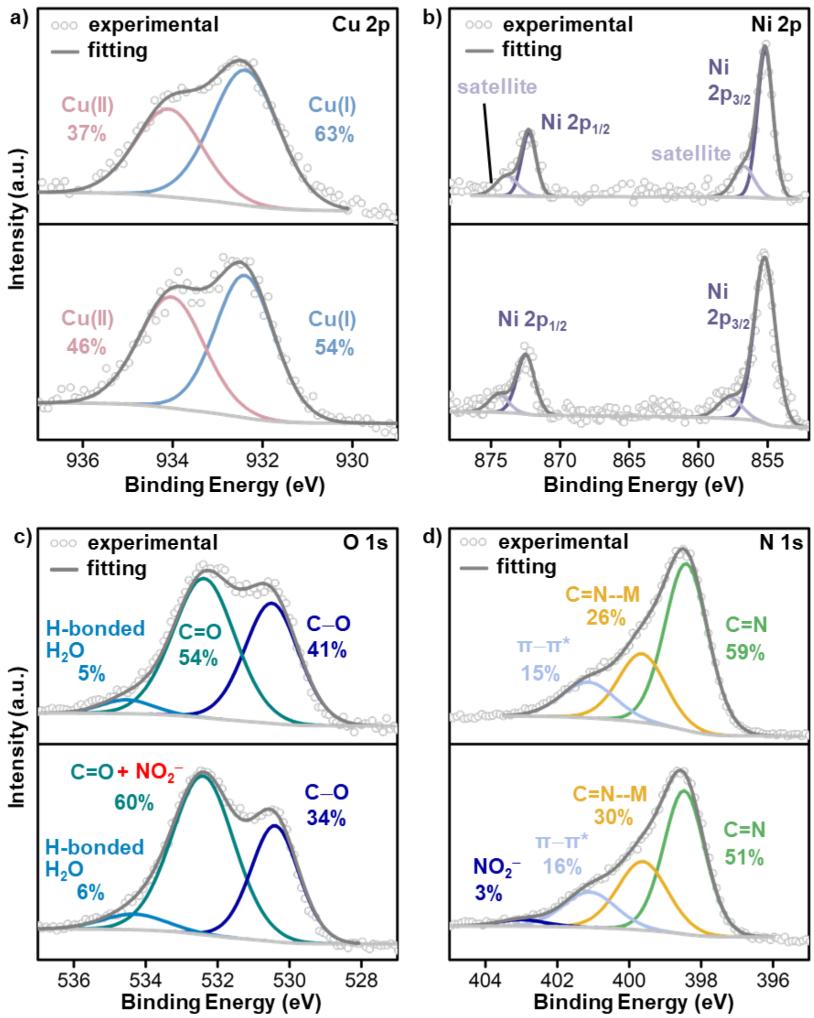

Figure S104. High-resolution XPS spectra of DC-100 after 2 hours of exposure to 1% NO in N_2 at the binding energies of a) Cu 2p, b) Ni 2p, c) O 1s, and d) N 1s.

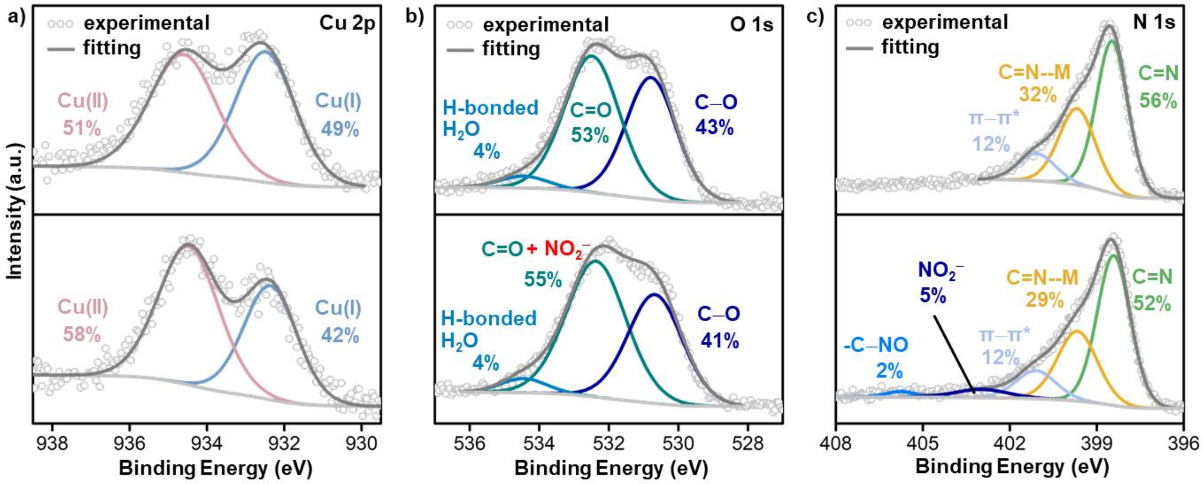

Figure S105. High-resolution XPS spectra of DC-103 after 2 hours of exposure to 1% NO in N_2 at the binding energies of a) Cu 2p, b) O 1s, and c) N 1s.

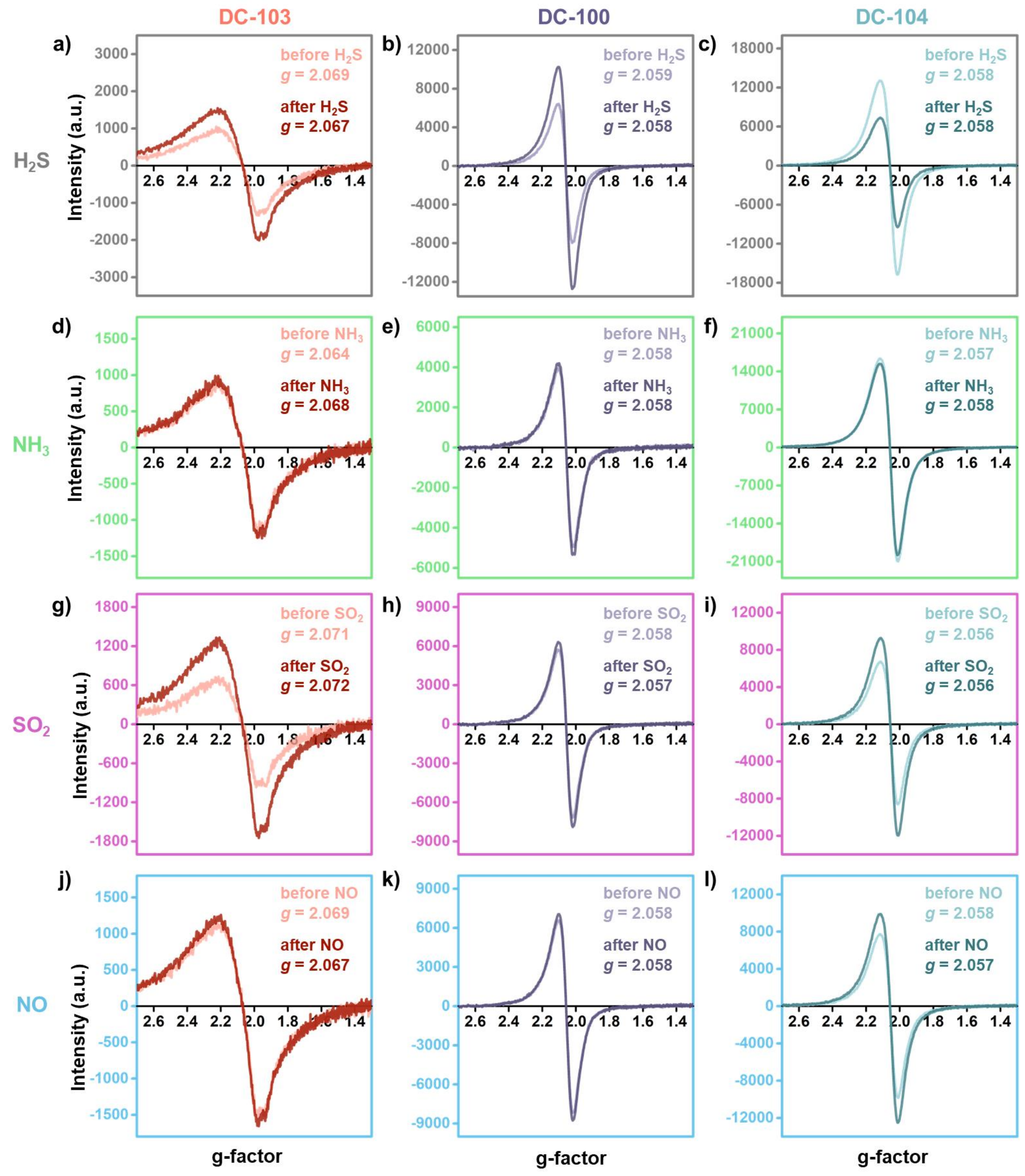

Figure S106. EPR plots taken at room temperature of MTPz-Cu-MOFs before and after exposure to a), b), c) H_2S, d), e), f) NH_3, g), h), i) SO_2, and j), k), l) NO.

S9. Optical Band Gaps Upon Gas Exposure

We performed optical band gap measurements upon gas exposure to 1% of each reducing gas in dry N_2 atmosphere for 2 hours. The results represented in **Figures S106-S108**, are obtained from the same quartz slide before and after exposure to analyte gas. The dropcasted slides and experimental procedures were similar as described in **Section S3.8**.

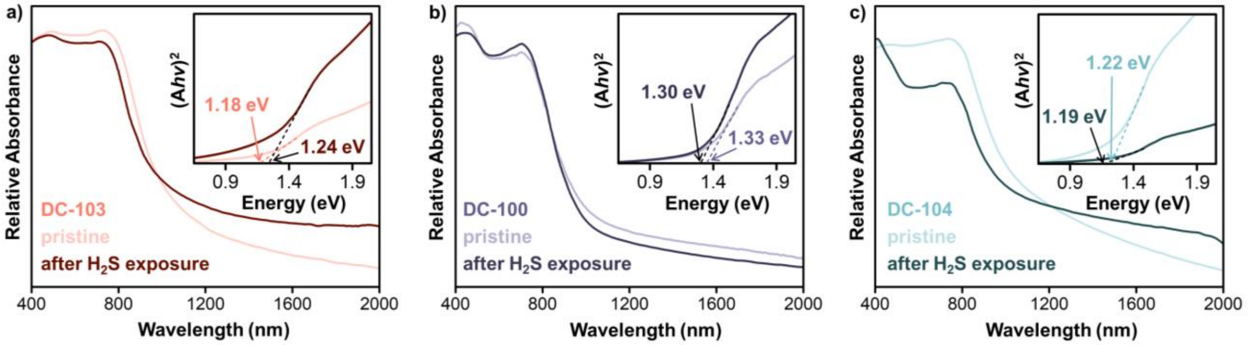

Figure S107. UV-Vis-NIR absorption spectra and inset graphs of the Tauc plots with the determined optical band gaps of the same thin film sample of pristine a) DC-103, b) DC-100, and c) DC-104 and after exposure to 1% H_2S in N_2 for 2 hours.

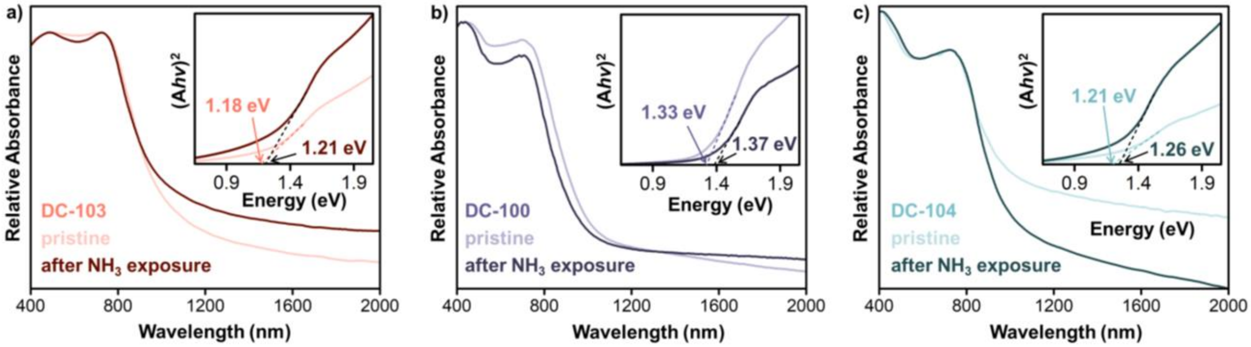

Figure S108. UV-Vis-NIR absorption spectra and inset graphs of the Tauc plots with the determined optical band gaps of the same thin film sample of pristine a) DC-103, b) DC-100, and c) DC-104 and after exposure to 1% NH_3 in N_2 for 2 hours.

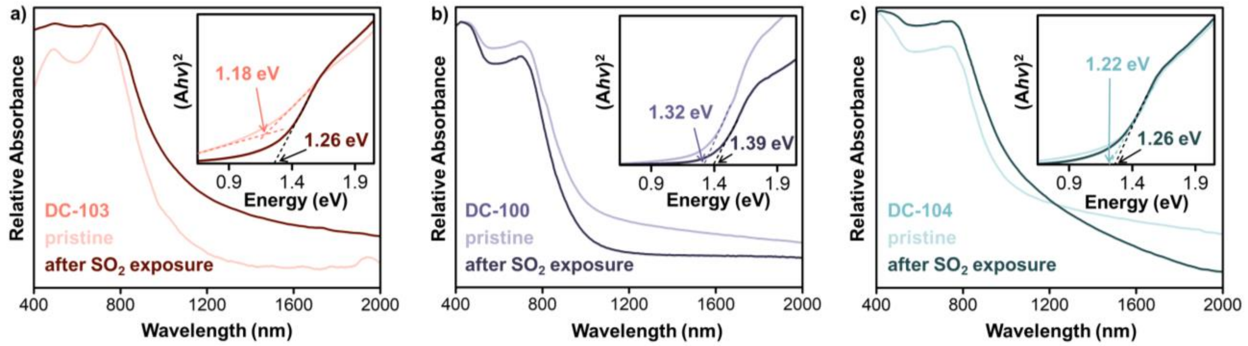

Figure S109. UV-Vis-NIR absorption spectra and inset graphs of the Tauc plots with the determined optical band gaps of the same thin film sample of pristine a) DC-103, b) DC-100, and c) DC-104 and after exposure to 1% SO_2 in N_2 for 2 hours.

S10. Electrochemical impedance spectroscopy

We performed electrochemical impedance spectroscopy (EIS) measurements for the three MOF analogs upon exposure to 40 ppm of H_2S or SO_2 in dry N_2 over 10 minutes. Measurements were acquired over a frequency range of 0.1 Hz to 2 MHz using 3 points per decade, an AC amplitude of 100 mV rms, and 0 V DC bias. These parameters enabled a total data acquisition time of around 2 minutes per spectrum, allowing time-resolved measurements during toxic gas exposure. Measurements were collected after around 15 minutes of purging with background gas (equivalent to the equilibration and baseline performed in the chemiresistive sensing experiments). Five consecutive spectra were collected at 2-minute intervals for a total exposure time of 10 minutes, consistent with the experimental procedures followed in the chemiresistive sensing experiments. EIS data were fitted using the equivalent electrical circuit shown in **Figure S110**, selected based on minimizing the goodness-of-fit parameter (χ^2), incorporating resistors (R), and a constant phase element (CPE).

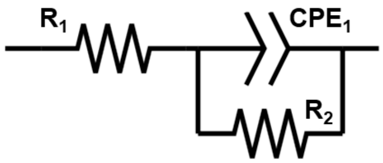

Figure S110. Equivalent electrical circuit used for fitting experimental EIS measurements in this study.

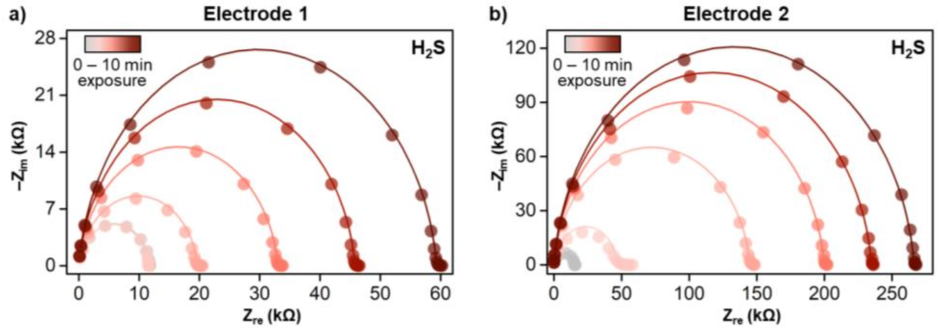

Figure S111. a,b) Nyquist plots of two independent DC-103 electrodes collected at 2-minute intervals during exposure to 40 ppm of H_2S in dry N_2. Dots represent experimental data, while lines represent fittings according to equivalent circuits shown in **Figure S110**.

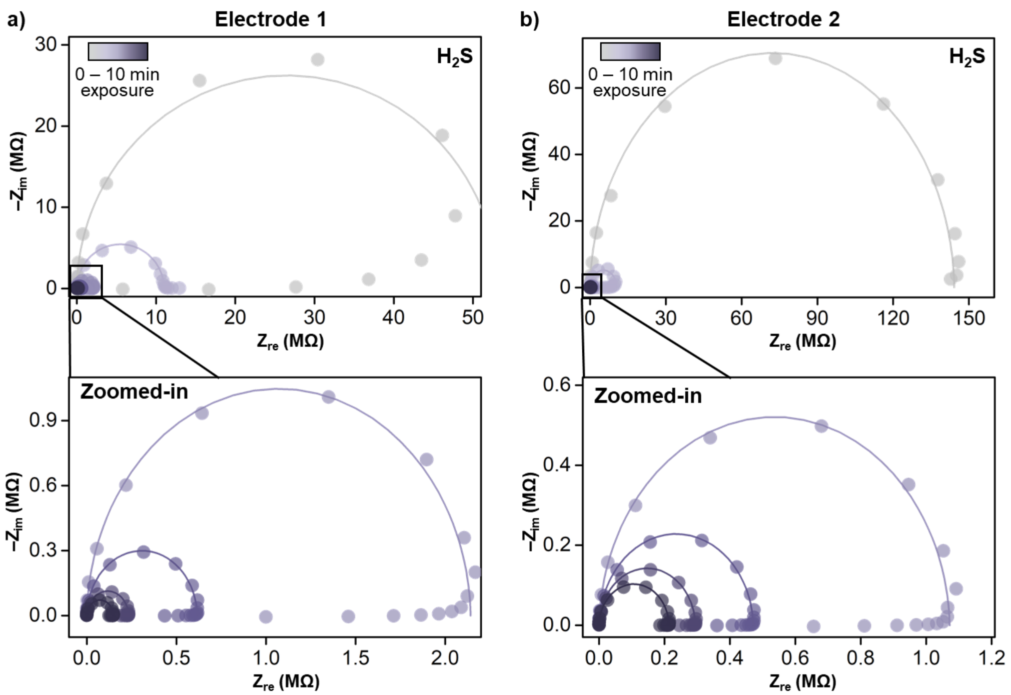

Figure S112. a,b) Nyquist plots of two independent DC-100 electrodes collected at 2-minute intervals during exposure to 40 ppm of H_2S in dry N_2. Dots represent experimental data, while lines represent fittings according to equivalent circuits shown in **Figure S110**.

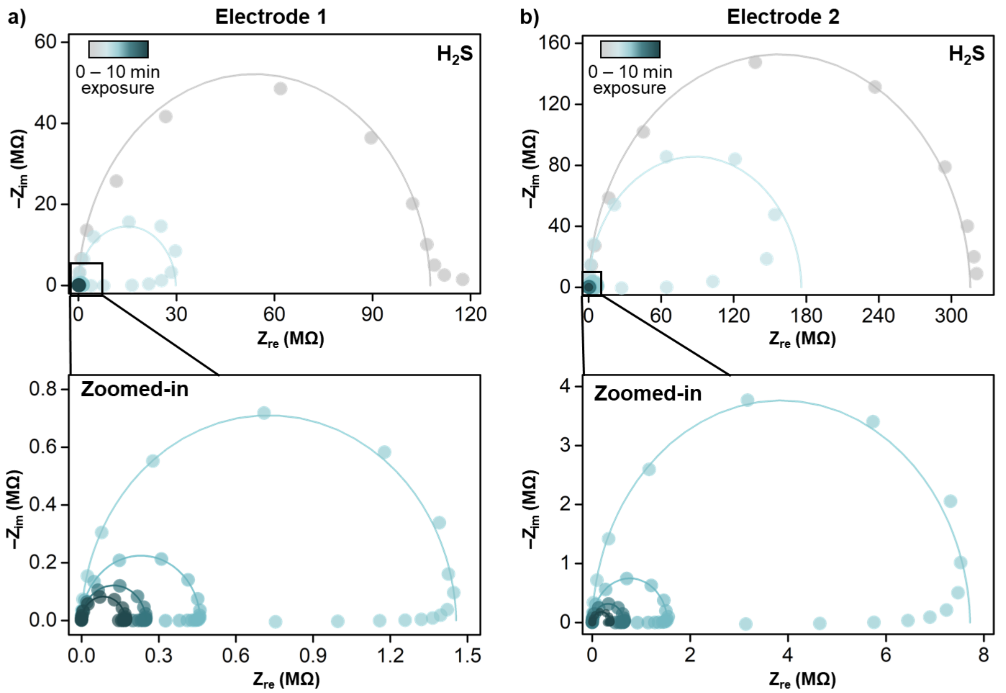

Figure S113. a,b) Nyquist plots of two independent DC-104 electrodes collected at 2-minute intervals during exposure to 40 ppm of H_2S in dry N_2. Dots represent experimental data, while lines represent fittings according to equivalent circuits shown in **Figure S110**.

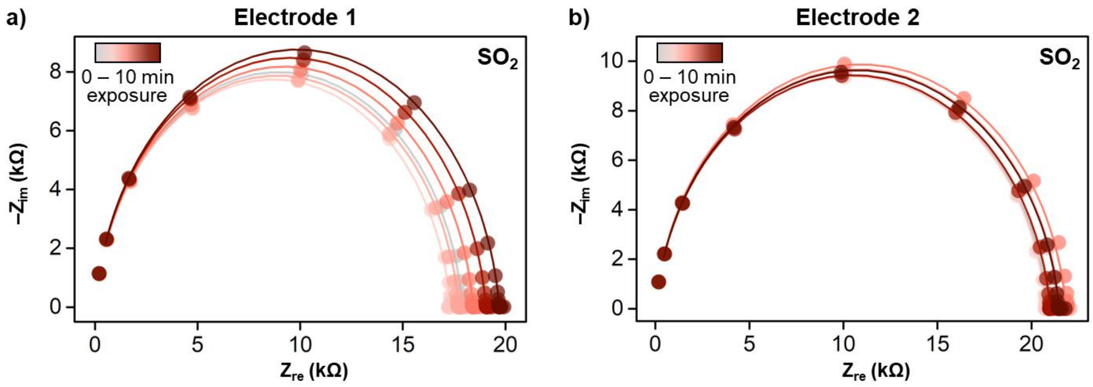

Figure S114. a,b) Nyquist plots of two independent DC-103 electrodes collected at 2-minute intervals during exposure to 40 ppm of SO_2 in dry N_2. Dots represent experimental data, while lines represent fittings according to equivalent circuits shown in **Figure S110**.

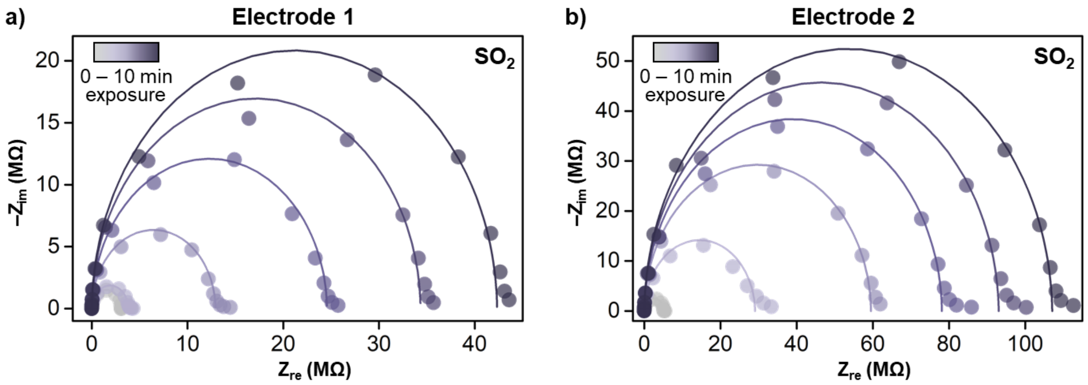

Figure S115. a,b) Nyquist plots of two independent DC-100 electrodes collected at 2-minute intervals during exposure to 40 ppm of SO_2 in dry N_2. Dots represent experimental data, while lines represent fittings according to equivalent circuits shown in **Figure S110**.

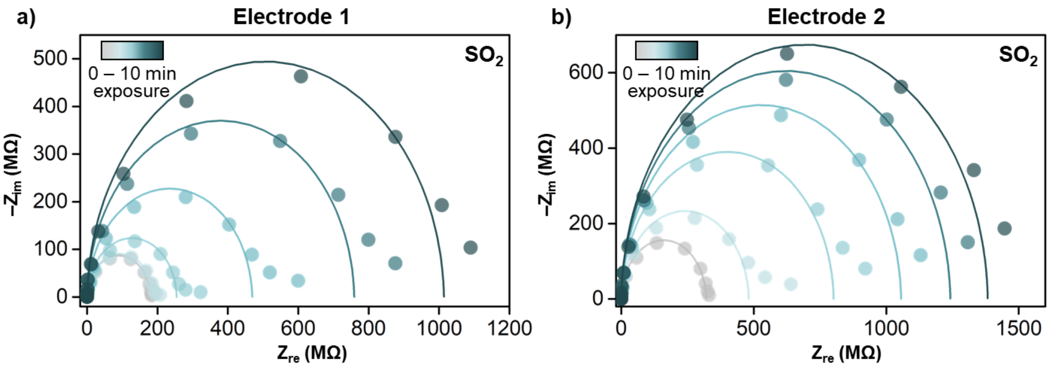

Figure S116. a,b) Nyquist plots of two independent DC-104 electrodes collected at 2-minute intervals during exposure to 40 ppm of SO_2 in dry N_2. Dots represent experimental data, while lines represent fittings according to equivalent circuits shown in **Figure S110**.

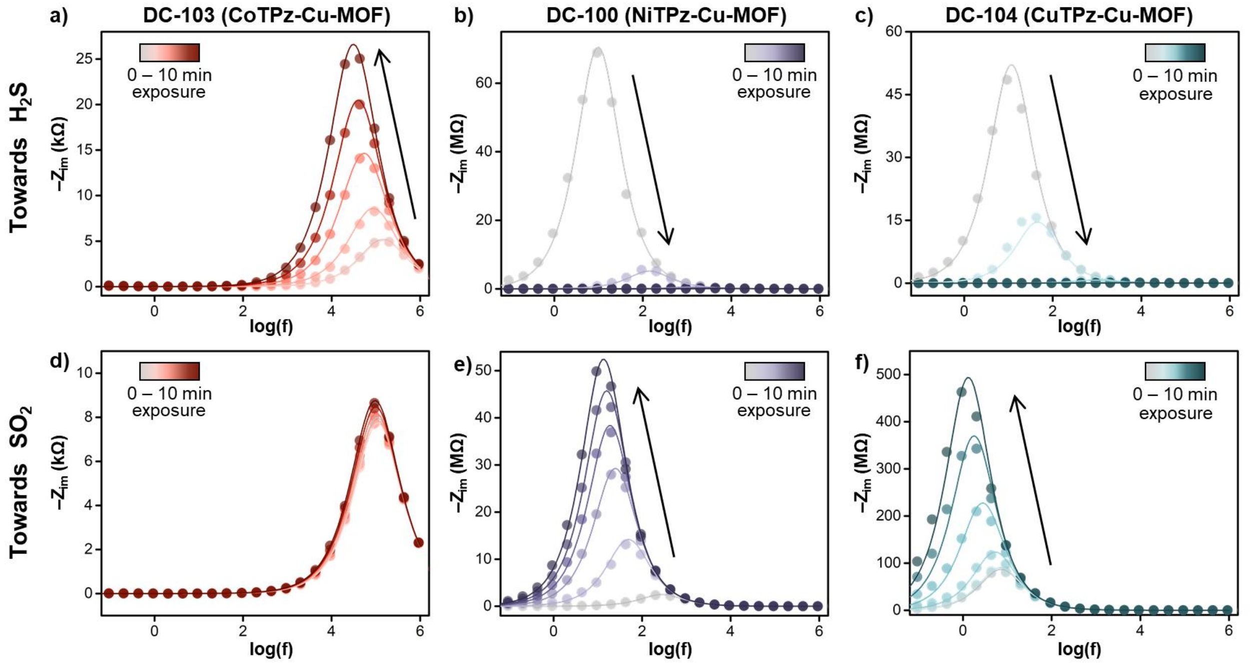

Figure S117. Representative plots of the negative imaginary impedance component as a function of logarithmic frequency for a) DC-103, b) DC-100, c) DC-104 upon exposure to 40 ppm of H_2S in dry N_2 and for e) DC-103, f) DC-100, g) DC-104 upon exposure to 40 ppm of SO_2 in dry N_2. Dots represent experimental data, while lines represent fittings according to equivalent circuits shown in **Figure S110**.

S11. Suspension characterization

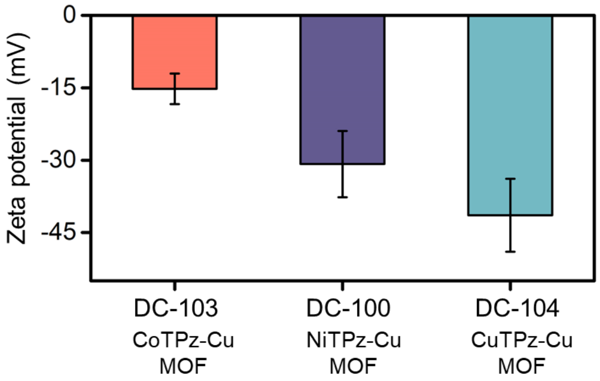

Figure S118. Bar graph showing average values of zeta potential for DC-103, DC-100, and DC-104, extracted from plots of **Figure S119**.

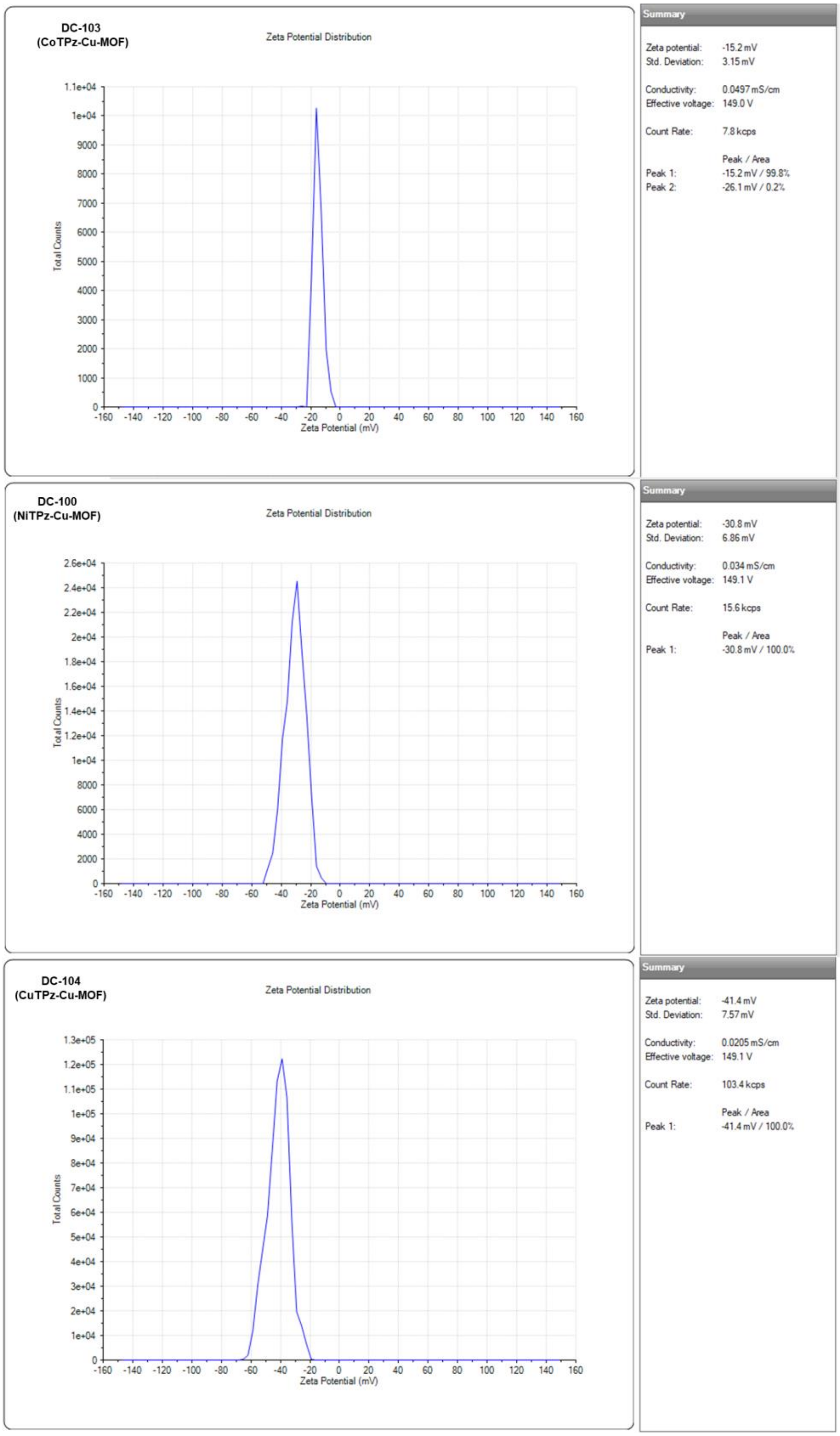

Figure S119. Raw data of zeta potential measurements of fresh DC-103, DC-100, and DC-104 MOF suspensions.

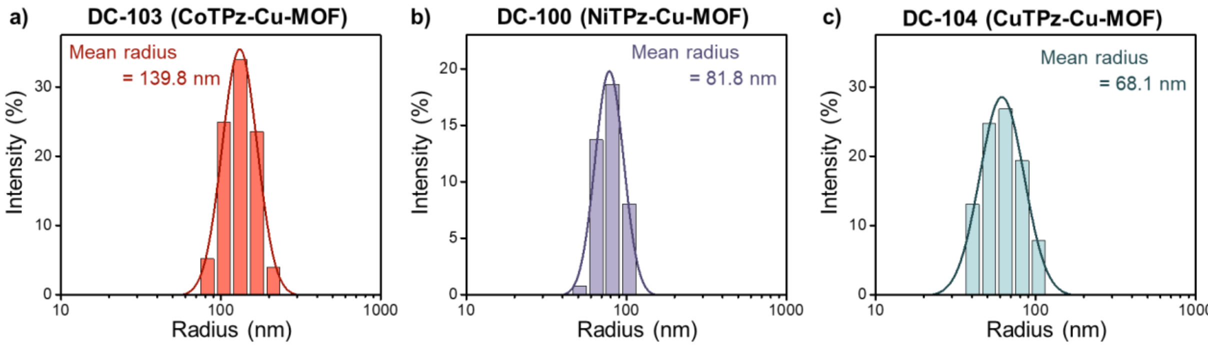

Figure S120. Dynamic light scattering (DLS) plots of a) DC-103, b) DC-100, and c) DC-104 MOF suspensions.

S12. References

1. Chan, J. Y. M.; Shehayeb, E. O.; Pennington, D. L.; Hendon, C. H.; Mirica, K. A., Molecular Engineering of a Conductive Metal–Organic Framework for Ultrasensitive, Rapid, Selective, and Reversible Sensing of Nitric Oxide. *J. Am. Chem. Soc.* **2025,** *147* (32), 29003-29012.
2. Fan, K. W.; Peterson, M. B.; Ellersdorfer, P.; Granville, A. M., Expanding the aqueous-based redox-facilitated self-polymerization chemistry of catecholamines to 5, 6-dihydroxy-1 H-benzimidazole and its 2-substituted derivatives. *RSC Adv.* **2016,** *6* (30), 25203-25214.
3. Zhou, Z.-L.; Weber, E.; Keana, J. F., Acetoxylation of 6, 7-dialkoxy-substituted 1, 4-dihydroquinoxaline-2, 3-diones (Qxs) using fuming nitric acid in acetic acid: A facile synthesis of 5-acyloxy-6, 7-dialkoxy QXs. *Tetrahedron Lett.* **1995,** *36* (42), 7583-7586.
4. Ding, J.; Wei, Z.; Li, F.; Zhang, J.; Zhang, Q.; Zhou, J.; Wang, W.; Liu, Y.; Zhang, Z.; Su, X.; Yang, R.; Liu, W.; Su, C.; Yang, H. B.; Huang, Y.; Zhai, Y.; Liu, B., Atomic high-spin cobalt (II) center for highly selective electrochemical CO reduction to CH_3OH. *Nat. Commun.* **2023,** *14* (1), 6550.
5. Meng, Z.; Aykanat, A.; Mirica, K. A., Welding Metallophthalocyanines into Bimetallic Molecular Meshes for Ultrasensitive, Low-Power Chemiresistive Detection of Gases. *J. Am. Chem. Soc.* **2018,** *141* (5), 2046-2053.
6. Zhong, H.; Ly, K. H.; Wang, M.; Krupskaya, Y.; Han, X.; Zhang, J.; Zhang, J.; Kataev, V.; Büchner, B.; Weidinger, I. M.; Kaskel, S.; Liu, P.; Chen, M.; Dong, R.; Feng, X., A phthalocyanine-based layered two-dimensional conjugated metal–organic framework as a highly efficient electrocatalyst for the oxygen reduction reaction. *Angew. Chem. Int. Ed.* **2019,** *58* (31), 10677-10682.
7. Smits, F., Measurement of sheet resistivities with the four-point probe. *Bell Syst. Tech. J.* **1958,** *37* (3), 711-718.
8. Makuła, P.; Pacia, M.; Macyk, W., How To Correctly Determine the Band Gap Energy of Modified Semiconductor Photocatalysts Based on UV–Vis Spectra. *J. Phys. Chem. Lett.* **2018,** *9* (23), 6814-6817.
9. Kresse, G.; Furthmüller, J., Efficient iterative schemes for ab initio total-energy calculations using a plane-wave basis set. *Phys. Rev. B* **1996,** *54* (16), 11169-11186.
10. Kresse, G.; Furthmüller, J., Efficiency of ab-initio total energy calculations for metals and semiconductors using a plane-wave basis set. *Comput. Mater. Sci.* **1996,** *6* (1), 15-50.

11. Kresse, G.; Joubert, D., From ultrasoft pseudopotentials to the projector augmented-wave method. *Phys. Rev. B* **1999,** *59* (3), 1758-1775.
12. Perdew, J. P.; Ruzsinszky, A.; Csonka, G. I.; Vydrov, O. A.; Scuseria, G. E.; Constantin, L. A.; Zhou, X.; Burke, K., Restoring the Density-Gradient Expansion for Exchange in Solids and Surfaces. *Phys. Rev. Lett.* **2008,** *100* (13), 136406.
13. Grimme, S.; Antony, J.; Ehrlich, S.; Krieg, H., A consistent and accurate ab initio parametrization of density functional dispersion correction (DFT-D) for the 94 elements H-Pu. *J. Chem. Phys.* **2010,** *132* (15).
14. Dudarev, S. L.; Botton, G. A.; Savrasov, S. Y.; Humphreys, C. J.; Sutton, A. P., Electron-energy-loss spectra and the structural stability of nickel oxide: An LSDA+U study. *Phys. Rev. B* **1998,** *57* (3), 1505-1509.
15. Wang, L.; Maxisch, T.; Ceder, G., Oxidation energies of transition metal oxides within the GGA+U framework. *Phys. Rev. B* **2006,** *73* (19), 195107.
16. Ammu, S.; Dua, V.; Agnihotra, S. R.; Surwade, S. P.; Phulgirkar, A.; Patel, S.; Manohar, S. K., Flexible, all-organic chemiresistor for detecting chemically aggressive vapors. *J. Am. Chem. Soc.* **2012,** *134* (10), 4553-4556.
17. Cline, E. L.; Noh, H.-J.; Benedetto, G.; Fabusola, G.; Hawkins, A.; Simon, C. M.; Mirica, K. A., Array of bimetallic metallophthalocyanine-based metal-organic frameworks for chemiresistive detection and differentiation of toxic gases. *Chem* **2026**, 103037.
18. Jiao, J.; Yang, M.; Ye, X.; Zhang, Y.; Jiang, Y.; Meng, Z., A Trimming-π Strategy for Constructing Functional Conductive Metal–Organic Frameworks Using Metalloporphyrazine Units. *Angew. Chem.* **2025,** *137* (20), e202502066.
19. Aykanat, A.; Meng, Z.; Stolz, R. M.; Morrell, C. T.; Mirica, K. A., Bimetallic Two-Dimensional Metal–Organic Frameworks for the Chemiresistive Detection of Carbon Monoxide. *Angew. Chem. Int. Ed.* **2022,** *61* (6), e202113665.
20. Kammoun, A.; Ravier, P.; Buttelli, O., Impact of PCA pre-normalization methods on ground reaction force estimation accuracy. *Sensors* **2024,** *24* (4), 1137.
21. Martins, A.; Fonseca, I.; Farinha, J. T.; Reis, J.; Cardoso, A. J. M., Online monitoring of sensor calibration status to support condition-based maintenance. *Sensors* **2023,** *23* (5), 2402.
22. Feng, L.; Musto, C. J.; Kemling, J. W.; Lim, S. H.; Suslick, K. S., A colorimetric sensor array for identification of toxic gases below permissible exposure limits. *Chem. Commun.* **2010,** *46* (12), 2037-2039.
23. Phillips, M. L.; Hall, T. A.; Sekar, K.; Tomey, J. L., Assessment of medical personnel exposure to nitrogen oxides during inhaled nitric oxide treatment of neonatal and pediatric patients. *Pediatrics* **1999,** *104* (5), 1095-1100.
24. Chowdhury, R. R.; Adnan, M. A.; Gupta, R. K., Real-Time Principal Component Analysis. *ACM/IMS Trans. Data Sci.* **2020,** *1* (2), Article 10.
25. Chowdhury, M. A. Z.; Oehlschlaeger, M. A., Artificial Intelligence in Gas Sensing: A Review. *ACS Sens.* **2025,** *10* (3), 1538-1563.
26. Jo, Y.-M.; Kim, D.-H.; Wang, J.; Oppenheim, J. J.; Dincă, M., Humidity-mediated dual ionic–electronic conductivity enables high sensitivity in MOF chemiresistors. *J. Am. Chem. Soc.* **2024,** *146* (29), 20213-20220.
